# AI-driven electrocatalysis: reinforcement learning-based control and theoretical modeling of the glycerol oxidation reaction

**DOI:** 10.1039/d6ee02239a

**Published:** 2026-10-05

**Authors:** Vladislav A. Mints, Maria-Magdalena Titirici, Ifan E. L. Stephens

**Affiliations:** a Department of Chemical Engineering, Imperial College London South Kensington Campus London SW7 2AZ UK vladislav.mints@empa.ch; b Advanced Institute for Materials Research (WPI-AIMR), Tohoku University Sendai 980-8577 Japan; c Department of Materials, Imperial College London South Kensington Campus London SW7 2AZ UK

## Abstract

In this work, we present the full development process of a reinforcement-learning-based controller deployed in a closed-loop experimental setup to optimize the glycerol electrooxidation reaction. The conversion of glycerol to valuable products on platinum has attracted attention as a substitute for the oxygen evolution reaction in hydrogen-producing electrolyzers, motivated by the availability of glycerol as an inexpensive byproduct of biodiesel production. However, the glycerol oxidation reaction is known to poison platinum, requiring an intelligent operation procedure to sustain high conversion rates. The employed AI controller autonomously discovered a non-periodic pulsing strategy, which achieved on average a 30% higher charge transfer rate over the human-engineered pulsing protocol. In addition, we analyzed its decision-making using first-order Markov-chain modelling. This analysis revealed that the AI employs two distinct surface reactivation procedures: most of the time, it uses a short cycle, pulsing from the glycerol-oxidation potential to a relaxation potential of 0.3 V *vs.* RHE, while it periodically performs a longer cycle by pulsing first to 1.0 V *vs.* RHE and then to 0.3 V *vs.* RHE to revitalize the system. To further support these findings, we developed an interpretable physics-inspired AI model that simulates surface dynamics during glycerol oxidation and captures the evolving distribution of active and poisoned sites. Although demonstrated for glycerol oxidation, both the AI-control framework and the physics-inspired AI model are broadly applicable to other electrochemical systems, offering a general pathway for integrating AI methods into experimental electrocatalytic research.

## Abstract 2

In this work, we present the full development process of a reinforcement-learning-based controller deployed in a closed-loop experimental setup to optimize the glycerol electrooxidation reaction.

## Graphical abstract

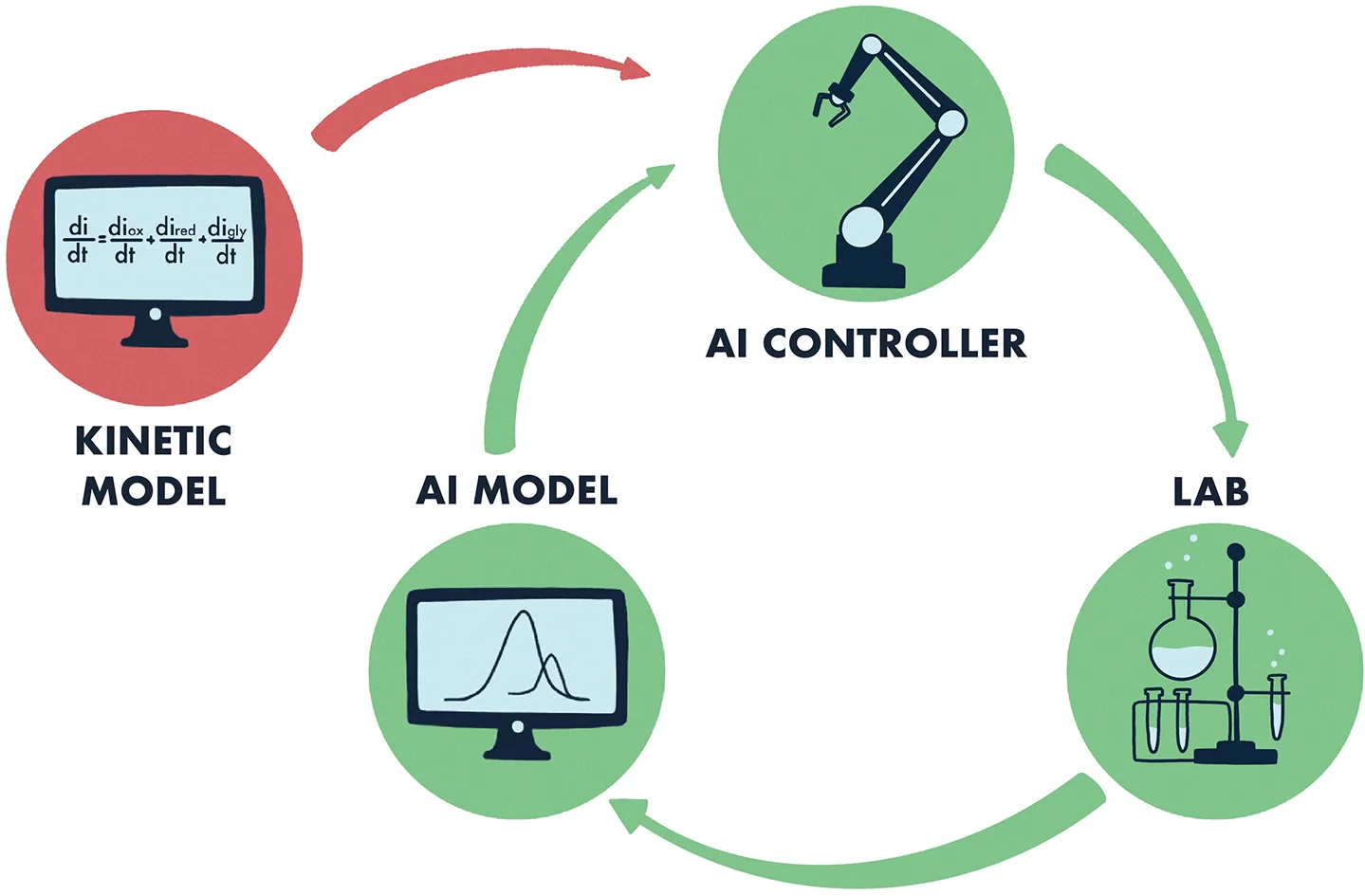

**Broader context**

The decarbonization of the chemical industry is one of the most pressing challenges societies are facing. Electrifying chemical manufacturing with renewable energy offers a promising path forward, and electrocatalytic processes are central to this transition. Much of today's research focuses on discovering new catalyst materials, often accelerated by artificial intelligence (AI). However, the way an electrochemical system is operated also strongly influences the performance, prompting increasing interest in operating protocols. Most protocols are manually designed and remain fixed throughout the system's operation, even though the system continually evolves. Reinforcement learning offers an alternative way to control the system: it can learn online how to steer the process and adapt to changing conditions to maintain high performance. In this work, we present a workflow for building such an AI controller and apply it to glycerol oxidation. The resulting controller improves the conversion rate by 30% compared to a human-designed protocol. This demonstrates that AI can optimize not just catalyst materials but also their exploitation, opening a path toward AI-augmented electrolyzers, providing a sustainable synthesis route toward our most important chemicals.

## Introduction

In recent years, artificial intelligence (AI) has entered the field of electrocatalysis primarily as a tool to accelerate electrocatalyst discovery and optimization.^1^ This gave rise to the use of optimization algorithms to more efficiently screen for novel catalyst materials in laboratories^2–4^ and self-driving laboratories.^5,6^ It also contributed to AI augmented optimization workflows by using AI algorithms combined with experimental datasets and theoretical calculations to accelerate the discovery of novel catalysts.^7–9^ Beyond discovering new catalysts, however, AI can also be applied to operate existing catalysts more effectively by learning how to control reactions in real time through reinforcement learning, an area that remains comparatively understudied. A key contribution in this direction, by Xu *et al.*, demonstrated that AI-guided operation can improve the energy efficiency of a direct methanol fuel cell.^10^

Besides optimizing fuel-cell operation, AI also has strong potential to optimize electrosynthesis and electrolysis reactions. For example, in CO_2_ reduction, pulsed electrolysis can influence the product distribution, system stability and energy efficiency.^11–13^ An ideal pulsing policy for such systems must balance these competing performance metrics. However, the search for such policies is mostly manual, resulting in rigid pulse sequences that lack the flexibility to intrinsically adapt to changes occurring in the system. Reinforcement learning, on the other hand, has the potential not only to autonomously identify an optimal control policy but also to generate adaptive policies that respond to evolving system conditions. Beyond CO_2_ reduction, essentially all electrolysis systems that benefit from pulsed operation^14–17^ could be further improved by reinforcement-learning-derived control policies.

In this work, we used reinforcement learning to improve the glycerol electrooxidation reaction rate on platinum.^18^ Glycerol has attracted significant attention as an inexpensive byproduct of biodiesel production that can be upconverted into value-added chemicals *via* electrooxidation, while simultaneously substituting the oxygen evolution reaction in hydrogen-producing electrolyzers.^19–21^ However, like many carbon-based electrooxidation reactions,^22,23^ the glycerol oxidation reaction generates strongly adsorbed intermediates that deactivate the platinum surface,^24^ necessitating pulsed operation for efficient conversion.^25–27^ However, unlike the oxidation of smaller molecules such as formic acid,^28^ methanol,^29^ and ethanol,^30^ glycerol electrooxidation branches out and produces uncharacterized poisoning compounds that are particularly resistant to oxidative removal.^31,32^ As a consequence, there is no clear electrochemical pathway to remove these compounds from the platinum surface, making it challenging to manually design an optimal pulsing protocol.

Chen *et al.*^26^ proposed a two-step pulsing policy alternating between 0.3 V and 0.7 V *vs.* the reversible hydrogen electrode (RHE) at 2 Hz. They hypothesized that the short pulse at 0.7 V *vs.* RHE prevents the overoxidation of glycerol and the formation of the strongly adsorbed species, while the pulse at 0.3 V *vs.* RHE assists in their desorption. Plub-in *et al.*^27^ extended this concept by introducing a third potential step at 1.0 V *vs.* RHE.^25^ At 1.0 V *vs.* RHE, the platinum surface will be converted into platinum oxide, which is inactive for glycerol electrooxidation but may oxidize and remove strongly adsorbed poisoning species such as CO.^33^ The subsequent step to 0.3 V *vs.* RHE reduces the oxide back to metallic platinum, leading to partial platinum dissolution and redeposition,^34,35^ which roughens the surface and further decreases the coverage of poisoning species. However, both pulsing strategies were derived empirically, leaving open whether more optimal operating policies can be identified using reinforcement learning.

To enable the use of reinforcement learning in electrocatalysis, time-resolved electrochemical experiments must be reformulated as a series of state–action–state transitions (Fig. 1A). A system state at timestep *t* (state(*t*)) represents the full physical description of the electrochemical setup at that moment, including atomistically resolved surface, double layer structure, temperature, pressure, and other relevant parameters. When an action is applied, typically the imposition of a potential or current, the system evolves into the next state (state(*t* + 1)). However, in practice, the true state of the electrochemical setup is not experimentally accessible. Instead, we only observe a projection of this state, yielding a partially observed state or state-representation (state′(*t*)) (Fig. 1B). In most electrochemical experiments, this representation corresponds to the measured current–voltage pair (*E*(*t*), *i*(*t*)), although it may be augmented with additional *in situ* techniques. Consequently, any time-resolved electrochemical experiment can be written as a trajectory of state-representations and actions, laying a general foundation for AI-based analysis and control.

**Fig. 1:**
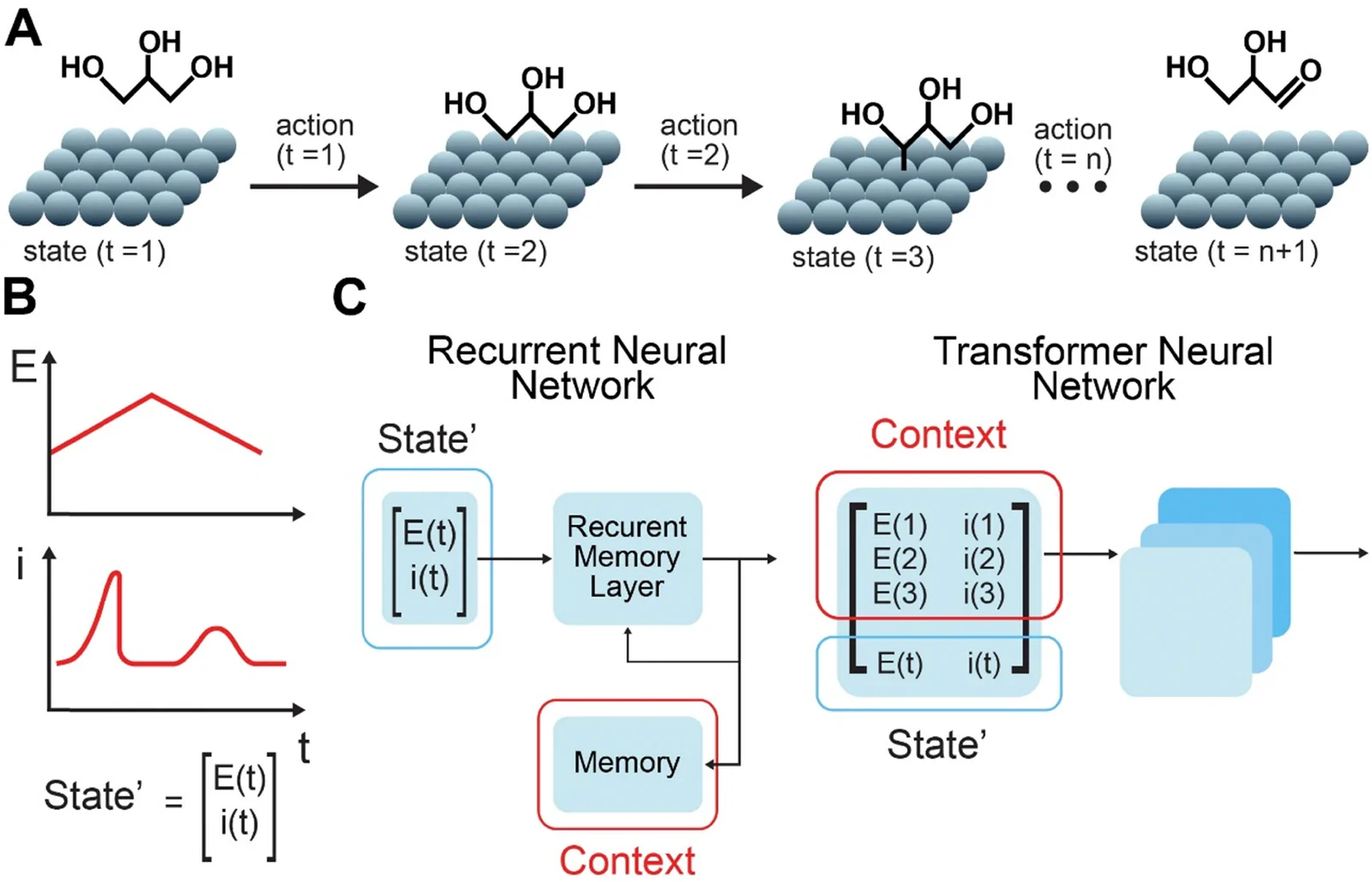
(A) An illustrative visualization of the glycerol electrooxidation reaction system as a sequence of state–action–state transitions. (B) An unfolded cyclic voltammogram of the glycerol electrooxidation reaction showing that the current voltage pair is only a representation of the underlying state. (C) An illustration of different strategies to create context when handling the partially observable states.

The concept of partially observed states and how to treat these is well-established in AI literature. The general approach is to introduce context, similar to how a human anticipates the next frame in a movie based on preceding frames. There are two major strategies for incorporating such context into neural networks. The first relies on a recurrent neural network (RNN), in which a recurrent memory layer infers a latent state representation that is carried forward to the next timestep, thereby providing context for future predictions (Fig. 1C).^36,37^ However, because this memory must be updated sequentially, RNNs train slowly, particularly when the dataset is large (>TB). The second approach is to provide context by supplying a sequence of preceding state-representations as part of the input, which is then processed by a transformer model.^38^ Transformers use an attention mechanism that mathematically encodes the relative importance of each element in the sequence prior to inference. As they are non-recurrent, transformers process data in parallel and therefore train significantly faster than RNNs. This transformer architecture forms the foundation of today's large-language models, explaining why transformers have become so widely adopted.

In this work, anticipating a relatively small dataset (<GB), we used an RNN to both control and model the glycerol oxidation reaction. Inspired by DeepMind's Agent57^39^ success in mastering classic Atari games, the first objective was to optimize the pulsing policy using a deep recurrent Q-network,^40^ following the development cycle outlined in Fig. 2. At this stage, the goal was to maximize the overall charge transfer rate rather than optimizing product distribution. Accordingly, the optimal control policy requires balancing the active and recovery phases. Targeting a specific product distribution would require a different reward function, as well as a time-resolved product analysis method, and thus represents a continuation of this work. This workflow began with a simple kinetic model as an entry point and then proceeded through iterative cycles of laboratory deployment, analysis, and refinement, ultimately yielding a controller that outperformed the engineered three-step policy by approximately 30%. The second objective was to construct an interpretable, physics-inspired AI model capable of capturing the dynamic evolution of the platinum surface during glycerol electrooxidation. This was achieved by developing a custom fully trainable Butler–Volmer type neural layer, which was incorporated into an architecture designed to simulate an evolving active site distribution. Combined, this work provides a general pathway for integrating AI into electrocatalysis research.

**Fig. 2:**
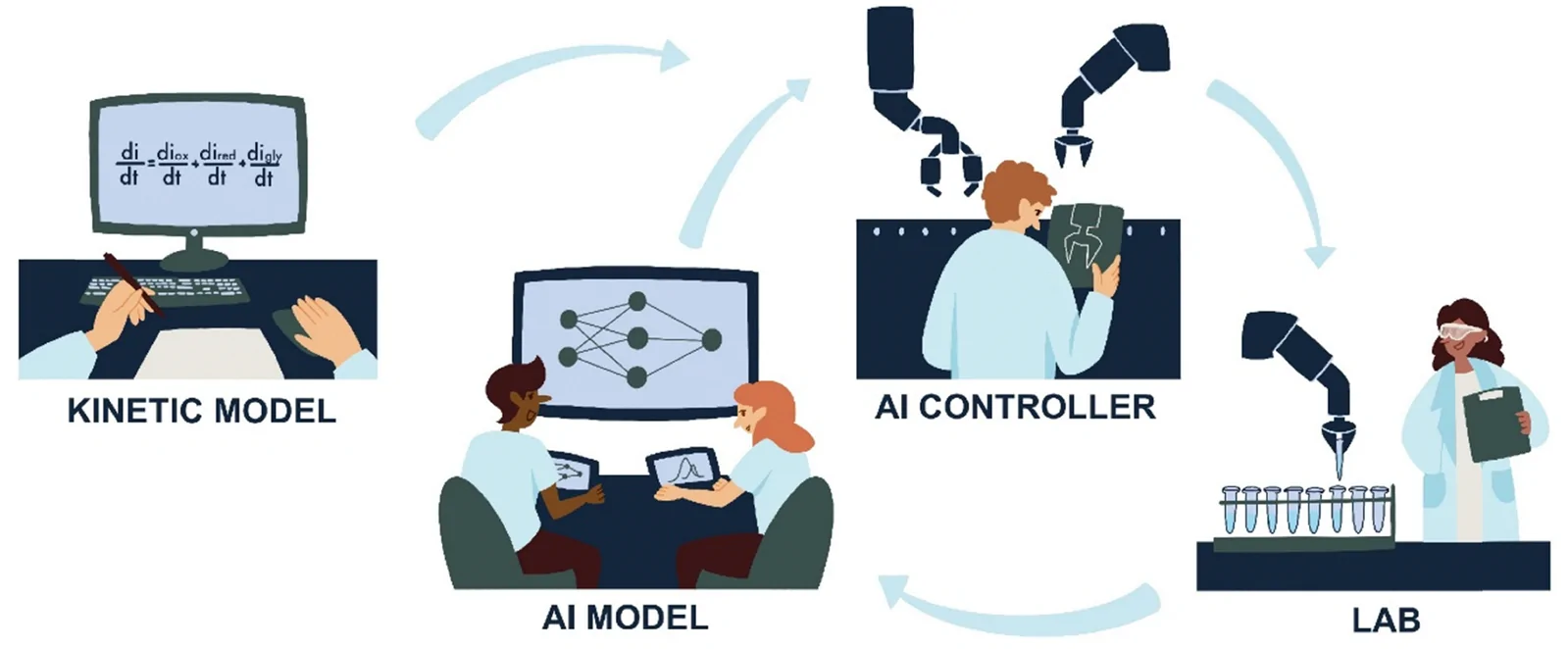
Development cycle of the AI-controllers and AI-models in this work.

## Results

### Development of AI Controller 1

As the first stage of the AI development cycle (Fig. 2), we constructed a simplified kinetic model (Section II.2, SI) to train and benchmark an initial AI controller before deploying it in the laboratory. The purpose of this model was to create a controlled environment that favors the emergence of the proposed three-step policy (π_3-step_), which applies a repeating cycle of 0.3–0.8–1.0 V *vs.* RHE.^27^ Consequently, the resulting model, outlined in eqn (S1)–(S8), does not provide mechanistic insight into the glycerol electrooxidation reaction. A notable modelling choice was the artificial separation of the oxide formation and reduction windows: oxide formation was restricted to potentials above 0.81 V *vs.* RHE, whereas oxide reduction was constrained to potentials below 0.79 V *vs.* RHE. These non-overlapping windows generate the sharp features observed in the simulated cyclic voltammograms (CVs) shown in Fig. 3A.

**Fig. 3:**
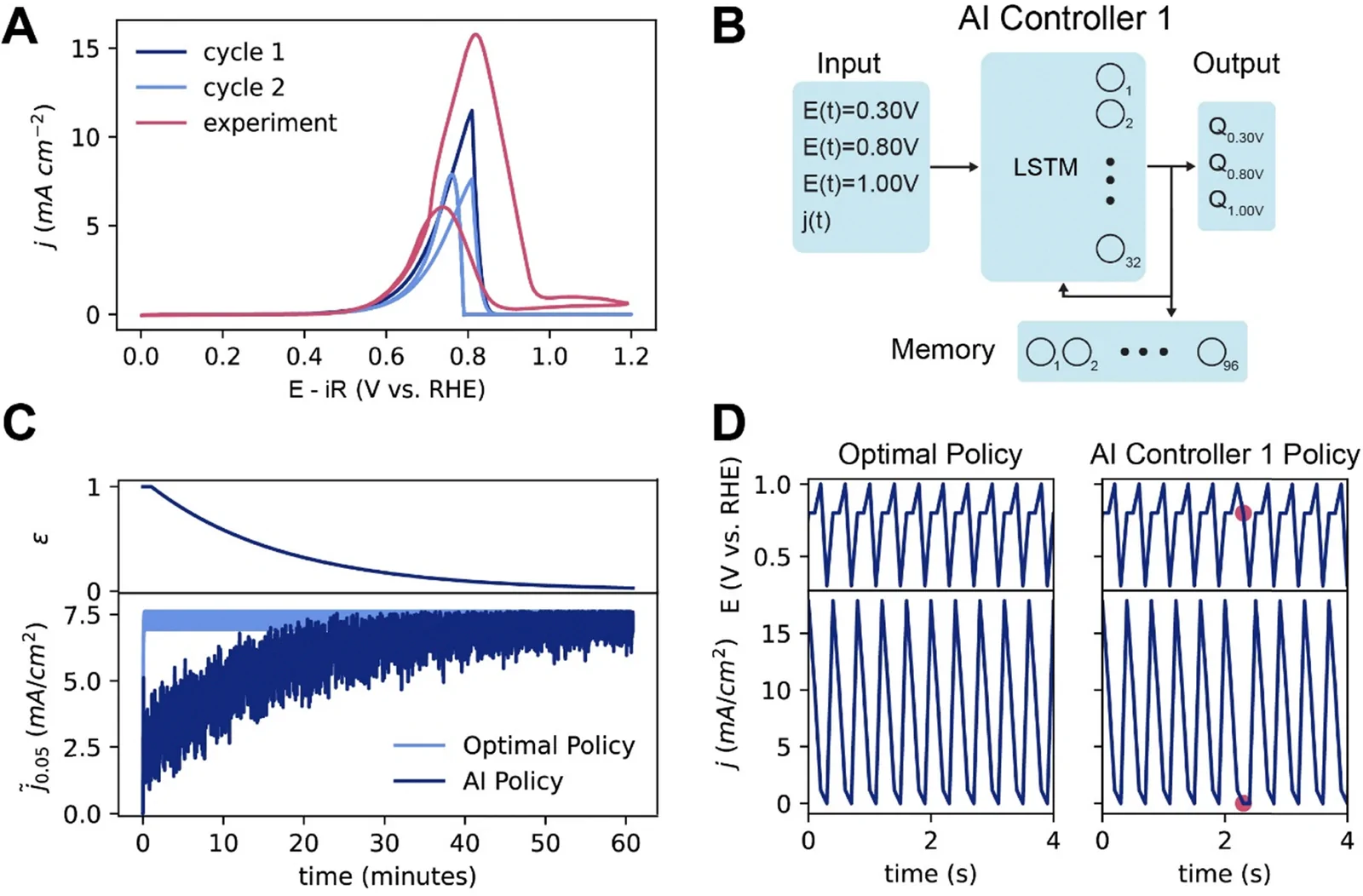
(A) Measured and simulated CVs for glycerol electrooxidation on Pt in 0.1 M KOH and 0.1 M glycerol (Ar saturated) at 50 mV s^−1^ and 200 rpm. The measured experiment was conducted with an Ag/AgCl reference electrode and a platinum mesh counter electrode in the same compartment. (B) Architecture of AI Controller 1. (C) Simulation of the training process of AI Controller 1 on the kinetic model, showing *ε* decay over time, the exponentially weighted moving average of the current density (*j̃*_0.05_) during training (blue), and the corresponding average under the optimal heuristic policy (light blue). (D) Optimal policies as identified numerically and using AI Controller 1; the red marker indicates an exploratory action selected under the *ε*-greedy policy.

This kinetic model was used to evaluate and benchmark the first AI Controller, based on a deep recurrent Q-network,^39,40^ whose structure is shown in Fig. 3B (SI Section II.1.3). The objective of this controller was to autonomously learn to maximize the glycerol electrooxidation rate. The model input consisted of current–voltage pairs, which serve as state representations rather than the true system states. To compensate for this partial observability, the controller was equipped with a long short-term memory (LSTM) layer.^41,42^ Thus, prior to each decision, a sequence of preceding state representations was supplied to initialize the memory and provide temporal context. Since the system operated at three discrete potentials, 0.3, 0.8, and 1 V *vs.* RHE, each associated with distinct electrochemical behavior, the applied potential was one-hot encoded to eliminate artificial numerical correlations in the input space. The model output corresponded to *Q*-values for each possible action, where each action consisted of applying one of the three potentials. These *Q*-values represent the cumulative reward obtained by selecting a given action and following the optimal policy thereafter.

The reward for the AI controller had to reflect the objective of maximizing the overall glycerol electrooxidation rate without targeting a specific product distribution. As the capacitive, oxide-formation and reduction charges cancel each other out, the average current can be attributed solely to the glycerol electrooxidation rate. Therefore, the reward was defined as the observed current with a small additional bonus proportional to the average current (eqn (1)). To calculate the average current at each time step, a sliding-window average could be used, but this would introduce computational overhead. Instead, the average current was approximated using an exponentially weighted moving average, as defined in eqn (2), with a smoothing factor *λ* of 0.05, corresponding approximately to a sliding window of 20 steps. Throughout this manuscript, the exponentially weighted moving average is denoted with a tilde (*j̃*_*λ*_), whereas the true average current is denoted with a bar (*j̄*) (Fig. S3). Future rewards were scaled using a discount factor *γ*, which determines the relative weight of delayed *vs.* immediate rewards; here, *γ* was set to 0.9, corresponding to an effective planning horizon of approximately ten steps.

1 reward = *j*(*t*) + 0.2*j̃*_0.05_(*t*)

2 *j̃*_*λ*_(*t*) = *λj*(*t*) + (1 − *λ*)*j̃*_*λ*_(*t* − 1)

To enable the AI controller to freely explore different strategies, an *ε*-greedy policy was employed.^43^ At each timestep, prior to action selection, a random value between 0 and 1 was drawn. When this value was below the *ε*-threshold, the controller followed a uniform random policy π_random_, which applies one of the three predefined potentials with equal probability, irrespective of the current system state. Conversely, when the sampled value exceeded *ε*, the controller selected the action associated with the highest *Q*-value, corresponding to the learned AI policy (π_AI_).

The real-time simulation of the training process of AI Controller 1 is shown in Fig. 3C. Over the course of one hour, the ε-threshold decreases and the controller increasingly selects actions based on π_AI_. As this policy consistently outperforms the random exploratory policy π_random_, *j̃*_0.05_ rises accordingly. By the end of the simulation, the controller converges to the same optimal pulsing strategy as identified by the numerical trial-and-error solver (Fig. 3D). However, because *ε* does not decay to zero, the controller retains a small degree of exploration, which slightly disrupts the execution of the optimal policy.

### Deployment of AI Controller 1

Next, AI Controller 1 was deployed in a real laboratory environment. This setup consisted of a platinum rotating disk electrode (RDE), rotating at 200 rpm, in 0.1 M KOH and 0.1 M glycerol (Ar-saturated). The measurements were acquired using a Biologic SP-300 potentiostat interfaced with a custom Python script through the Biologic API (SI Section II.1). In this setup, the system applied a potential for 100 ms after which the corresponding current–voltage pair was recorded and passed to the AI controller for the next decision. Because the controller's decision-making process was not instantaneous, slight computational delays introduced variability in the true timestep, which ranged between 100 and 200 ms. All figures in this manuscript account for these delays and display the corrected time series. Additionally, due to a solution resistance of approximately 40 Ω, the applied potential of 0.8 V *vs.* RHE was adjusted to 0.85 V to compensate for the ohmic drop.

Because extended runs risked memory crashes on the control workstation, the experiment was executed as a sequence of automatic restarts with one-hour sub-experiments. This yielded 18 sequential runs (Fig. S29), with sub-experiment 4 shown in Fig. 4A as a representative. In every sub-experiment, AI Controller 1 achieves peak performance with *ε* ≈ 0.35. More specifically, across sub-experiments 4–18, the controller achieves an average current density of 6.3 ± 0.4 mA cm^−2^ within the *ε* range of 0.3–0.4. As *ε* decreases further, the performance correspondingly declines reaching an average current density of 4.8 ± 0.3 mA cm^−2^ for *ε* < 0.05. This indicates that the controller delivers high performance only when supported by ∼35% random exploration and cannot sustain the high performance on its own. Consequently, the next step in developing a robust AI controller was to analyze and understand the shortcomings of AI Controller 1.

**Fig. 4:**
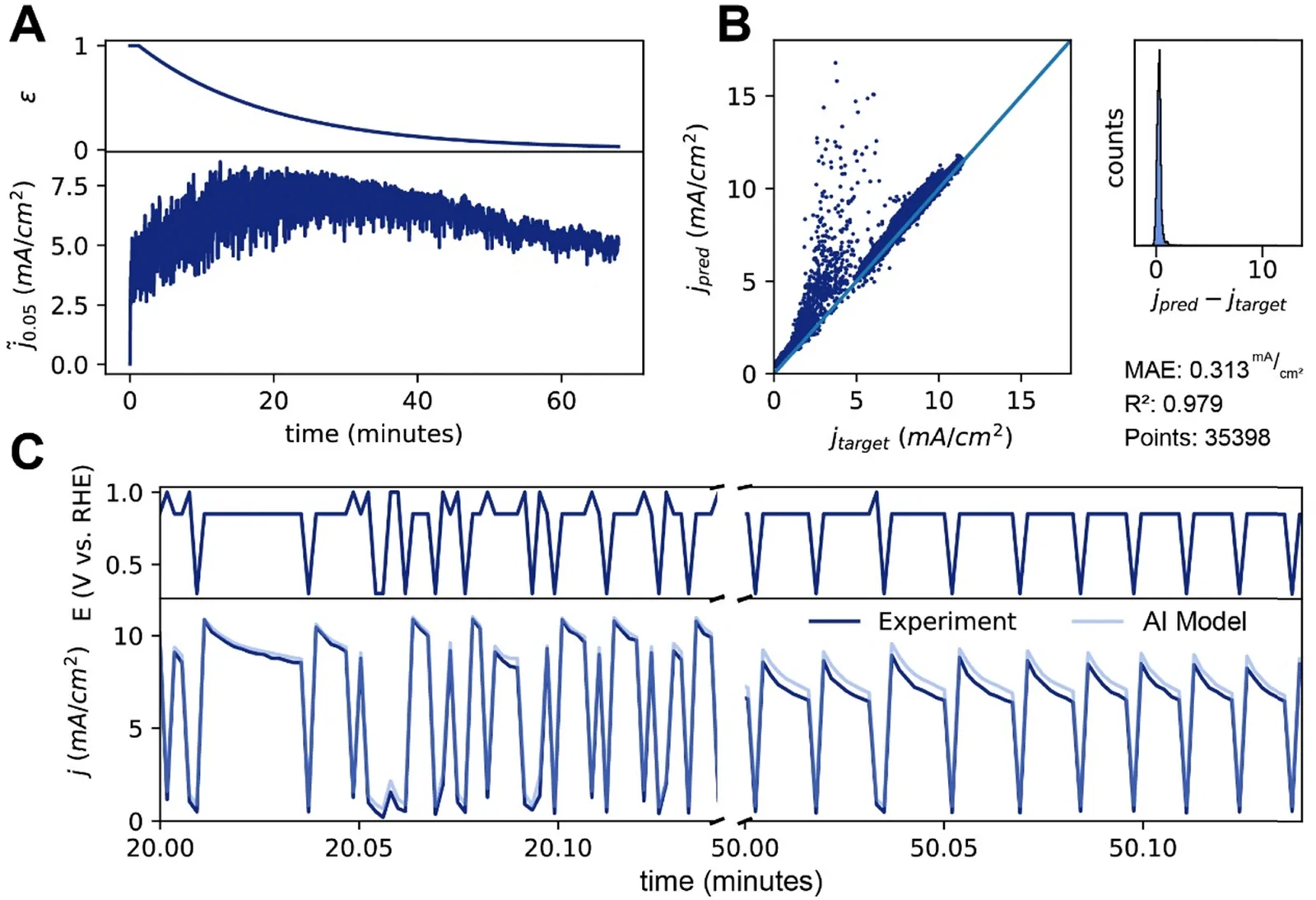
(A) Experiment controlled by AI Controller 1, showing the evolution of the exponentially weighted moving average current density (*j̃*_0.05_) and *ε*, which determines the fraction of AI-guided *versus* random actions. The data correspond to experiment 10.4, conducted in 0.1 M KOH and 0.1 M glycerol (Ar-saturated) at 200 rpm using a platinum disk working electrode, a platinum mesh counter electrode in the same compartment, and an Ag/AgCl reference electrode. (B) Parity plot of AI Model 1 comparing predicted (*j*_pred_) and measured current densities (*j*_target_). Predictions were obtained from a free-running simulation of the full time series of experiment 10.4; the first 600 samples were used to initialize the hidden state. The inset shows the distribution of prediction errors. (C) Applied potential and the corresponding current density during experiment 10.4 under AI control, shown at 20 min (*ε* = 0.36) and 50 min (*ε* = 0.074).

To perform this investigation, we used the data collected during these runs to train AI model 1. This AI model, based on a recurrent neural network, was trained using a teacher forcing approach using sub-experiments 1–3 (SI Section III.1) and validated using sub-experiments 4 and 5. The model replicated the three training experiments in a free-running simulation with a mean absolute error (MAE) of 0.51 mA cm^−2^. On the validation sub-experiment 4, AI Model 1 achieved an MAE of 0.31 mA cm^−2^, as shown in Fig. 4B. Fig. 4C illustrates a segment of this free-running simulation and its close resemblance to the experimental measurement. Therefore, these results establish AI Model 1 as an accurate and realistic virtual testing environment, making it an effective offline diagnostic tool for AI Controller 1.

AI Model 1 was used to simulate an experiment operated fully under the learned policy π_AI_ of AI Controller 1 (Fig. 5A). In this simulation, the controller rapidly pulses between 0.3 and 0.85 V *vs.* RHE without ever selecting 1.0 V, which results in a smooth and stable average current density of approximately 5.5 mA cm^−2^. The same simulation was repeated with the introduction of a single artificial pulse to 1.0 V at 10 s (Fig. 5B). This oxidative pulse causes a brief drop in average current to 5.1 mA cm^−2^, followed by recovery to a slightly higher value of 5.6 mA cm^−2^. These results demonstrate that occasional oxide forming pulses are essential to increase the conversion rate of the system, albeit at the cost of a short-lived decrease in activity. After this pulse, the controller resumes its two-step pulsing behavior, leading to a gradual decay from 5.6 to 5.5 mA cm^−2^ over approximately 5 s. This decay spans roughly 50 decision steps, far exceeding the effective planning horizon of ten steps used by Controller 1. Therefore, these simulations indicate that the controller must be modified to capture longer-range temporal dependencies.

**Fig. 5:**
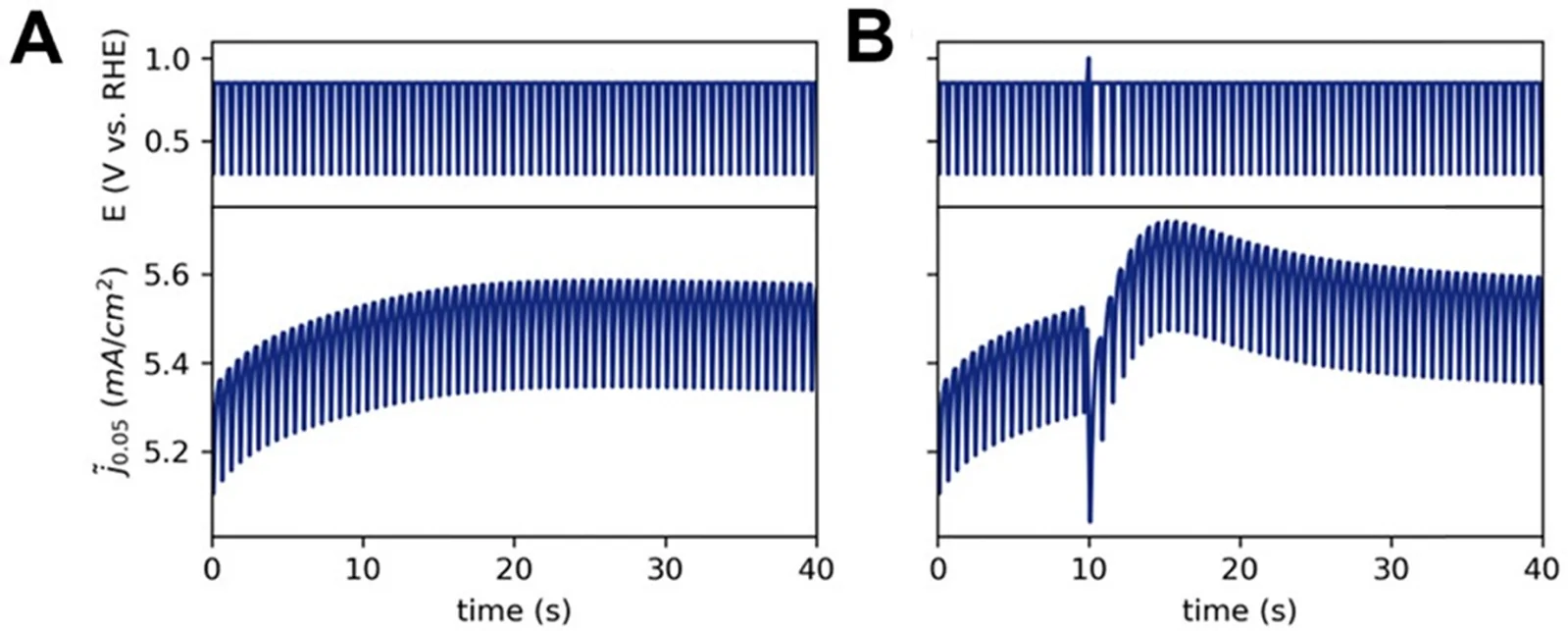
Evaluation of AI Controller 1 using AI Model 1, showing the simulated evolution of the exponentially weighted moving average current density (*j̃*_0.05_) in response to applied potentials. The hidden state of AI Model 1 was initialized using 600 points from experiment 10.4 in the 57–58 min interval. (A) Simulation of the average current density obtained when AI Controller 1 governs the potential sequence in the AI-modelled environment. (B) Same simulation as in panel A, but with a single 1.0 V pulse introduced at 10 s.

### AI Controller 2

These insights led to the development of the second controller, AI Controller 2, which addressed the shortcomings of AI Controller 1. AI Controller 2 employs a deeper network architecture (Fig. 6A), enabling it to capture long-range temporal dependencies that Controller 1 could not resolve. In addition, the discount factor was increased from 0.9 to 0.995, extending the effective planning horizon from approximately 10 to 200 timesteps and allowing the controller to pursue longer-sighted strategies. To further support this extended planning, *j̃*_0.05_ was included as part of the input, as it is a proxy for the ‘health’ of the platinum surface. The reward function was also reshaped to use *j̃*_0.05_ directly as the reward signal; to stimulate high average currents, it was defined according to eqn (3) so that, below 5 mA cm^−2^, the reward equals *j̃*_0.05_ whereas above, the reward is tripled, encouraging operation in the higher-performance regime. Because prolonged operation at 0.85 V *vs.* RHE is expected to promote poisoning, a penalty was introduced for repeating the same action more than ten consecutive times to encourage rapid pulsing. Finally, AI Controller 2 was pretrained on the dataset obtained from the first experiment, providing a more effective starting point for online learning.

3 reward = *j̃*_0.05_ + max(0,2(*j̃*_0.05_ − 5))

**Fig. 6:**
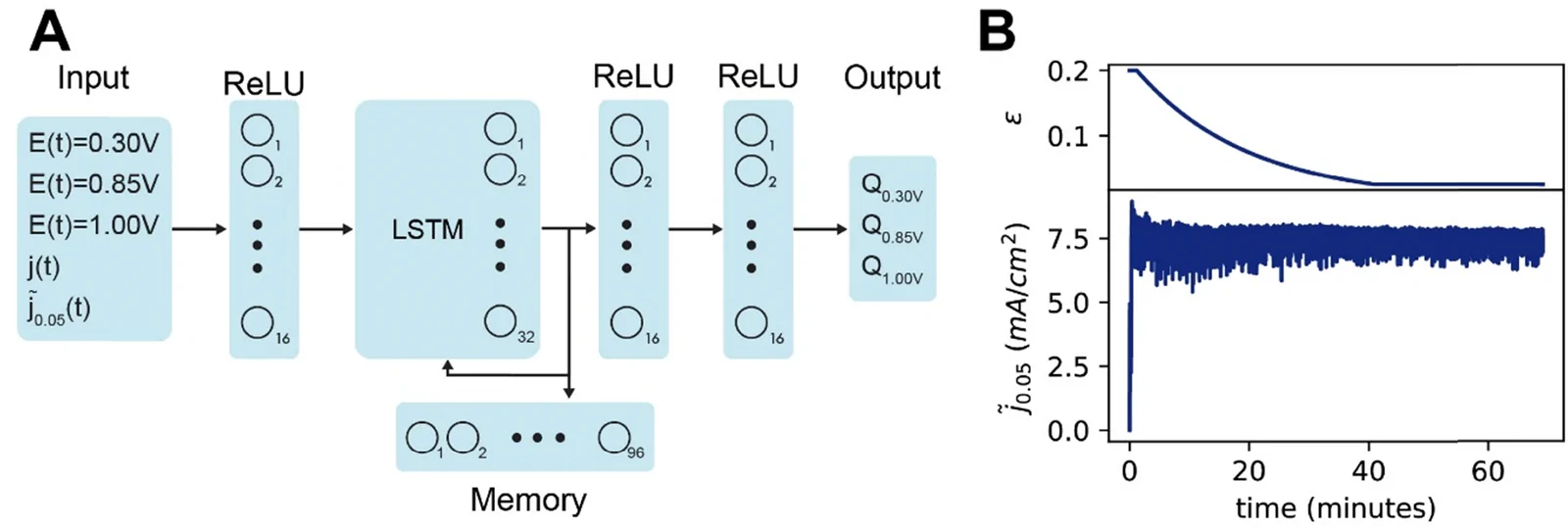
(A) Architecture of AI Controller 2. (B) Experiment controlled by AI Controller 2, showing the evolution of the exponentially weighted moving average current density (*j̃*_0.05_) and *ε*, which determines the fraction of AI-guided *versus* random actions. The data correspond to experiment 11.2.8, conducted in 0.1 M KOH and 0.1 M glycerol (Ar-saturated) at 200 rpm using a platinum disk working electrode, a platinum mesh counter electrode in the same compartment, and an Ag/AgCl reference electrode.

AI Controller 2 was deployed in the laboratory using the same procedure as that of AI Controller 1, with the experiment automatically restarting every hour and producing a total of 13 sub-experiments. Because the model was pretrained, the *ε* value started at 0.2 and decayed to 0.025, reaching a regime approximately 20 min long in which only 2.5% of the actions were exploratory. Unlike AI Controller 1, AI Controller 2 maintains a stable *j̃*_0.05_ of approximately 7.5 mA cm^−2^ throughout the full duration of the experiment (Fig. 6B). Notably, this value closely corresponds to the peak *j̃*_0.05_ achieved by AI Controller 1 at *ε* ≈ 0.36. The sustained, high *j̃*_0.05_ demonstrates that AI Controller 2 has converged to a high-performing policy π_AI_.

The converged AI Controller 2 was compared to the proposed 3-step policy π_3-step_.^27^ This 3-step policy consists of a repeating sequence of 0.3–0.85–1 V *vs.* RHE, with each potential applied for 100 ms. To ensure that both policies operated under identical conditions, they were alternated within the same experiment, each for a 15-minute interval. AI Controller 2 achieves on average a current density *j̄* of 7.86 mA cm^−2^, representing a 29% improvement over the 3-step policy, which delivers 6.06 mA cm^−2^ on average (Fig. 7A).

**Fig. 7:**
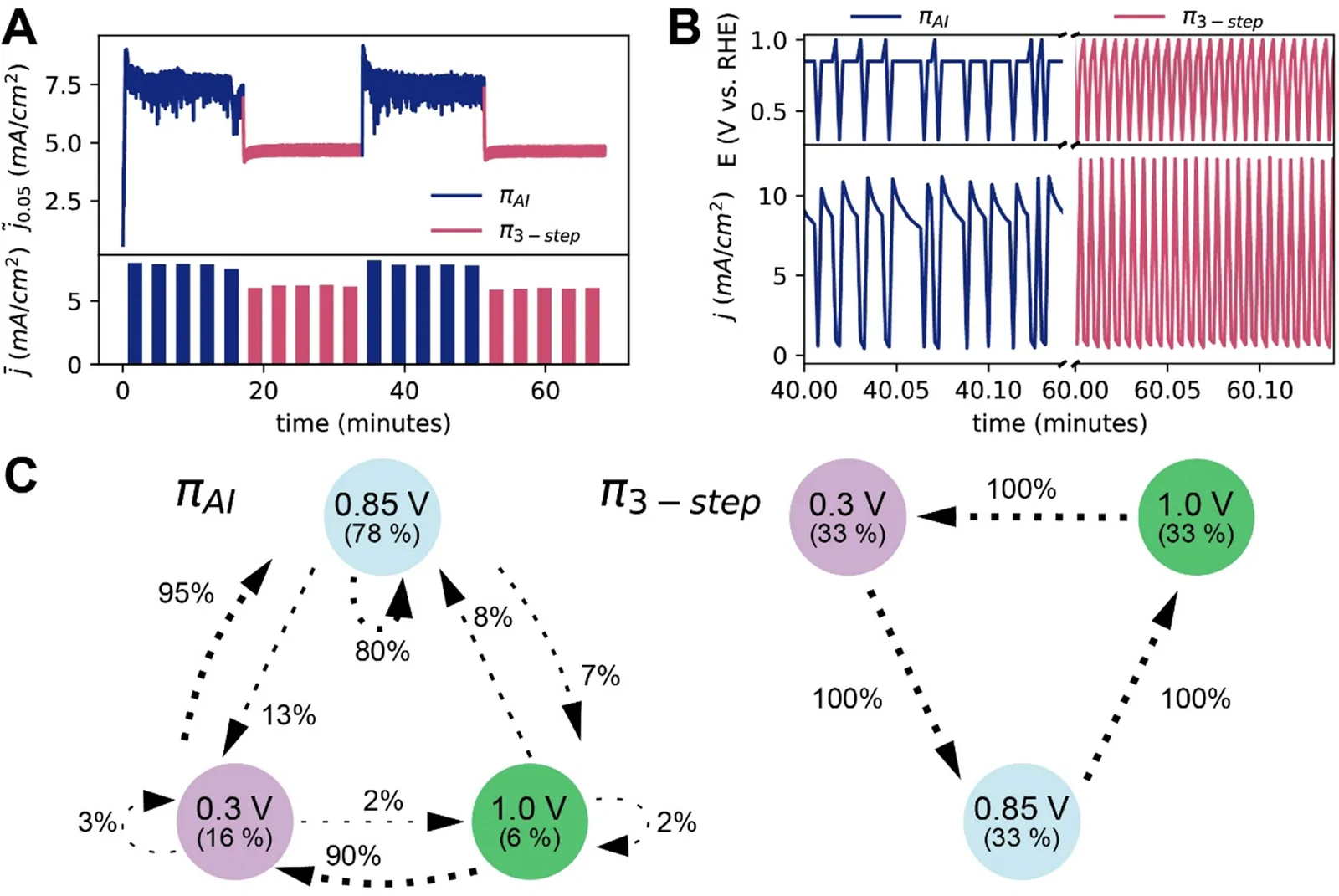
(A) Comparison of AI Controller 2 with the proposed three-step policy during experiment 11.3.1 in 0.1 M KOH and 0.1 M glycerol (Ar-saturated) at 200 rpm. The working electrode was a platinum disk, with a platinum mesh counter electrode in the same compartment and an Ag/AgCl reference electrode. The policy of AI Controller 2 was evaluated at *ε* = 0.05, corresponding to 5% random exploration. The panel shows the controller's exponentially weighted moving average current density (*j̃*_0.05_) alongside the true average current density (*j̄*), computed from the raw data stream using bins of 1800 decision steps. (B) Zoom-in on the operating regimes under the AI-derived policy and the three-step policy. (C) Markov-chain analysis of the AI-controlled regime (34–51 min) and the three-step pulsing regime (51–68 min). The numbers above the arrows indicate transition probabilities between potentials, and the numbers in parentheses within each node indicate the frequency with which that potential was visited.

To visualize the differences between the AI-derived policy and the 3-step policy, a first-order Markov-chain analysis was performed.^44^ This analysis quantifies how frequently the system occupies each potential and how likely it is to transition between them. Under AI control, the system resides at 0.85 V for 78% of the time, at 0.3 V for 16% of the time, and at 1.0 V for 6% of the time. From 0.85 V, the system remains at this potential with an 80% probability, transitions to 0.3 V with a 13% probability, and transitions to 1.0 V with a 7% probability. When 1.0 V is applied, a subsequent transition to 0.3 V occurs in 90% of the cases, after which the system returns to 0.85 V in 95% of the cases. In contrast, the three-step policy occupies each potential exactly 33% of the time and transitions deterministically in a fixed cycle.

These observations indicate that the AI controller has learned two distinct surface reactivation procedures to maintain an active surface and selects between them depending on the instantaneous state of the electrode. Moreover, these reactivation procedures closely resemble the empirically derived human policies.^25–27^ The first is the short cycle between 0.85 and 0.3 V *vs.* RHE, previously documented by Chen *et al.*^26^ The authors hypothesized that this cycle may suppress excessive formation and accumulation of PtO_*x*_ and assist the desorption of strongly adsorbed intermediates or products that are otherwise resistant to oxidation.^31,32,45,46^ According to the Markov-chain analysis, this short relaxation cycle is used most frequently, indicating that it serves as the primary surface-maintenance mechanism. The second is a longer cycle that steps from 0.85 to 1.0 V and then to 0.3 V *vs.* RHE.^25,27^ We hypothesize that the excursion to 1.0 V briefly forms an oxide layer capable of oxidizing small molecules (*e.g.*, CO),^47^ thereby accelerating their removal from the surface. The subsequent step to 0.3 V regenerates and restructures the metallic Pt surface, leading to a harsher reactivation cycle. However, because glycerol oxidation remains active at 0.3 V *vs.* RHE,^31^ the PtO_*x*_ reduction current is likely largely compensated, resulting in a small net oxidative current at this potential.^27^ This cycle is used less frequently, suggesting that the AI uses it only to complement the short reactivation cycle. Verification of this hypothesis would require *operando* spectroscopic measurements, such as rapid-scan *in situ* FTIR, synchronized with the AI-controller to monitor the evolution of surface-bound intermediates during the proposed reactivation cycles.

### AI Controller 3

Having established a robust three-potential policy with Controller 2, we next examined whether expanding the action space could deliver additional gains or reveal alternative operating regimes. This led to the development of AI Controller 3, which operated on a 19-potential action space spanning 0.3 to 1.2 in 50 mV increments. Controller 3 was trained and developed using the same workflow outlined in Fig. 2. To accommodate the substantially larger action space, the network was made deeper to capture more complex dynamics. Also, it was equipped with a dueling head, which has been shown to improve the performance of Deep Q-Network algorithms, leading to the final architecture shown in Fig. 8.^48^

**Fig. 8:**
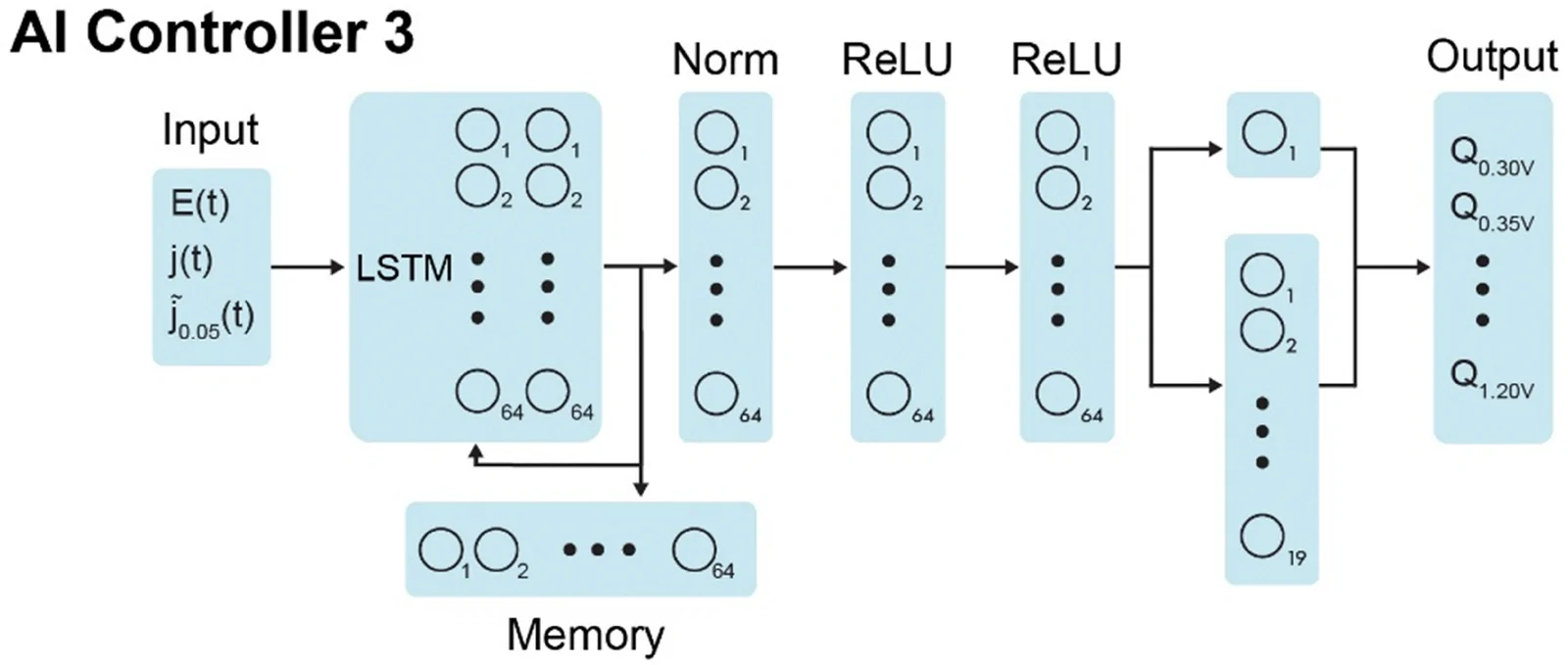
Architecture of AI Controller 3.

However, despite these modifications, the controller consistently overestimated *Q*-values of unseen observations. To mitigate this, it was trained offline using the Conservative Q-Network algorithm,^49^ which places additional emphasis on observed sequences relative to unseen ones. Furthermore, during deployment, online learning had to be disabled because it triggered catastrophic forgetting. Preventing such forgetting would have required access to the full historical dataset during operation, which was not feasible given the available hardware.

To assess the performance of the offline-trained AI Controller 3, we benchmarked its learned policy at *ε* = 0.05 against the three-step policy π_3-step_, a uniform random policy π_random_, and a center-biased random policy π_random-2_. This center-biased random policy had 64% chance of applying a voltage between 0.7 and 0.85 V *vs.* RHE, whereas the uniform random policy had only a 21% chance. This alternation of control policies also introduced random perturbations into the experimental design, which the AI controller successfully compensated for by consistently recovering its activity to a stable value (Fig. 9A). Thus, the AI controller successfully learned to adapt to random perturbations during experimental operations. Like in the previous benchmark experiment, this experiment alternated among the four policies under otherwise identical conditions and consisted of 14 sequential 2 hour long sub-experiments.

**Fig. 9:**
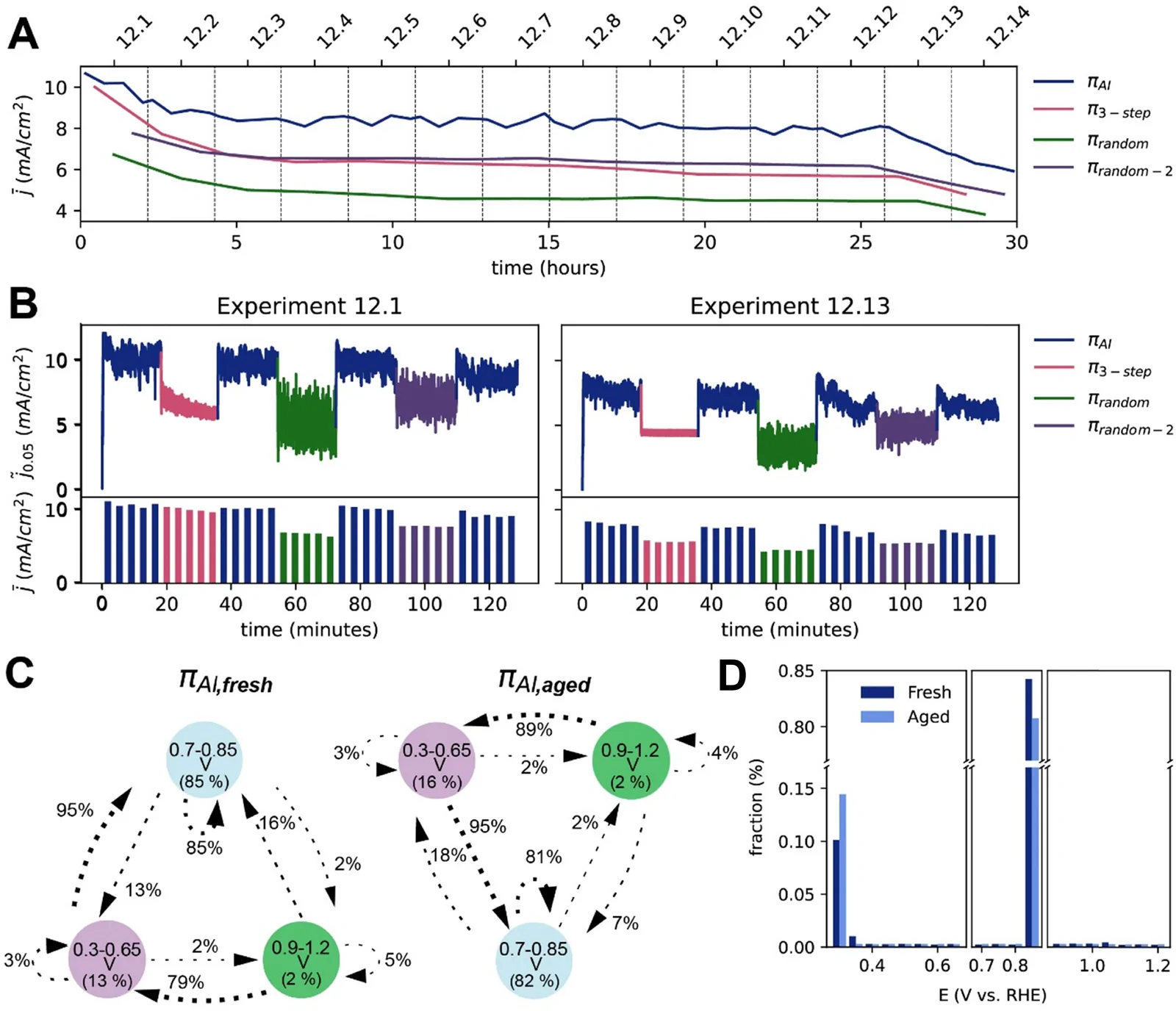
(A) Comparison of the performance of AI Controller 3 expressed as the true average current density (*j̄*) which was evaluated at *ε* = 0.05, with the three-step policy, a uniform random policy π_random_, and a center-biased random policy π_random-2_ in 0.1 M KOH and 0.1 M glycerol (Ar-saturated) at 200 rpm. The working electrode was a platinum disk, with a platinum mesh counter electrode in the same compartment and an Ag/AgCl reference electrode. The experiment is segmented into sub-experiments that were used to stabilize the electrolysis. (B) A zoom-in on experiments 12.1 and 12.13. The panel shows the controller's exponentially weighted moving average current density (*j̃*_0.05_) alongside the true average current density (*j̄*) computed from the raw data stream using bins of 1800 decision steps. (C) Markov-chain analysis of the AI-controlled regimes under fresh cell conditions (experiment 12.1) and aged cell conditions (experiments 12.2–12.13). The potentials are grouped according to their electrochemical response: relaxation potentials (0.3–0.65 V), glycerol-oxidation potentials (0.7–0.85 V), and oxide-formation potentials (0.9–1.2 V). (D) Potential distributions of AI control under fresh and aged cell conditions.

Experiment 12.1 marks the first two hours in a fresh cell (Fig. 9B). During the first hour, the AI Controlled regime reaches an average *j̃*, calculated from the data observed using the AI controller, of 10.3 mA cm^−2^, whereas the three-step policy yields an average *j̃* of 6.4 mA cm^−2^. However, when the true average *j̄* is calculated from the raw data stream, the values converge to 10.4 and 10.0 mA cm^−2^, respectively. This discrepancy arises from a combination of fresh-cell dynamics and solution resistance effects (Fig. S8). Under the three-step protocol, a pulse from 0.85 V to 1.0 V is applied to a metallic Pt surface, producing a brief current spike of ∼5 mA. With a solution resistance of ∼40 Ω, this transient current causes the effective electrode potential to be 0.8 V, a potential for which glycerol oxidation on metallic platinum remains highly active. Over the next ∼50 ms, the current rapidly decays to <1 mA as the surface becomes poisoned, shifting the effective potential into the oxide-formation regime and eventually passivating the surface. Because this process unfolds within ∼50 ms, it does not influence *j̃*, which samples only the currents at the end of the 100 ms control intervals. In contrast, the potentiostat does sample every 25 ms and captures these transients, which elevate *j̄* for the three-step policy reducing the difference between the two policies.

After the first two hours, the performance of all policies decreases markedly before reaching a stable regime that persists for the subsequent 26 h of operation (experiments 12.2 to 12.13), herein referred to as aged-cell conditions. Under these conditions, the true average *j̄* achieved by the AI policy decreases by 20%, from the initial 10.4 mA cm^−2^ to 8.2 ± 0.42 mA cm^−2^, where the uncertainty represents the standard deviation of the mean values obtained from consecutive 15 min operating intervals. In contrast, the 3-step policy loses 38% of its initial activity, decreasing from 10.0 to 6.2 ± 0.55 mA cm^−2^. Throughout this stable aged-cell regime, the AI policy maintains a 32 ± 6% higher average current density than the 3-step policy, consistent with the improvement observed using AI Controller 2. This value was determined by comparing each 3-step-policy interval with the direct neighboring AI-controlled intervals.

Experiment 12.13 marked the end of the stable aged-cell conditions and occurred after approximately 26 hours of the electrolysis time (Fig. 9B). Here, during the first hour, the AI controlled regime reached an average *j̃* of 7.5 mA cm^−2^ and a corresponding *j̄* of 7.8 mA cm^−2^. In comparison, the three-step policy achieved *j̃* and *j̄* values of 4.4 and 5.6 mA cm^−2^, respectively. Due to the prolonged electrolysis time, the system was partially deactivated, leading to lower overall currents. Under these aged-cell conditions, the pulse from 0.85 to 1.0 V in the three-step policy produced only a modest transient of 2–3 mA, which decayed within ∼25 ms. As a result, the difference between *j̃* and *j̄* is substantially smaller compared to fresh-cell conditions, justifying the use of *j̃* as an online measure for the average activity.

The decision-making behavior of AI Controller 3 was also analyzed using a first-order Markov-chain model. Because a full 19-node network would obscure the underlying structure, the potentials were grouped according to their electrochemical response: relaxation potentials (0.3–0.65 V *vs.* RHE), glycerol-oxidation potentials (0.7–0.85 V *vs.* RHE), and oxide-formation potentials (0.9–1.2 V *vs.* RHE). The resulting Markov-chain diagrams (Fig. 9B) exhibit patterns closely resembling those of AI Controller 2. In the fresh-cell experiment, the controller remains in the glycerol-oxidation regime for 85% of the time and pulses to a relaxation potential with a probability of 13%. When entering the oxide-formation regime, the system transitions back to a glycerol-oxidation potential with a probability of 16% and to a relaxation potential with a probability of 79%. In the aged--cell experiment, the controller spends 82% of the time in the glycerol oxidation regime and pulses more frequently to relaxation potentials (18%). Transitions from oxide-formation potentials in the aged cell show a strong resemblance to AI Controller 2, with an 89% probability of transitioning to a relaxation potential, which subsequently transitions with 95% probability to a glycerol oxidation potential. This suggests that AI Controller 3 learned a highly similar policy to AI Controller 2.

To further elucidate the controller's decision-making process, the Markov-chain results were complemented with an unrolled potential-distribution diagram (Fig. 9C). This diagram reports how frequently each potential is selected within the three electrochemical groups. In both the fresh- and aged-cell experiments, AI Controller 3 primarily operates at 0.3 and 0.85 V *vs.* RHE, effectively converging to the same action space identified by AI Controller 2. Within the oxide-formation regime, however, the controller exhibits no clear preference for a specific potential, suggesting that any potential between 0.9 and 1.2 V *vs.* RHE provides a comparable result. Nevertheless, the inclusion of an oxide-formation step remains essential, as eliminating this regime altogether leads to catalyst deactivation.^27^ In particular, convergence to a purely two-potential policy is responsible for the poor performance of AI Controller 1 (Fig. 4 and 5). A minor difference arises between the fresh- and aged-cell conditions in the relative use of 0.3 and 0.85 V: in the fresh cell experiment, the system is more active, and the controller spends less time reactivating the surface at 0.3 V, whereas in the aged cell it resorts to this relaxation potential more frequently. This suggests that the controller performs only modest adjustments to its strategy in response to the overall state of the system.

These results indicate that effective control of the glycerol electrooxidation reaction requires only three potentials: a recovery potential around 0.3 V, a high-activity potential around 0.85 V and an oxide-formation potential within approximately 0.9–1.2 V. Although expanding the action space to 19 potentials could, in principle, enable finer control, it demands substantially more data due to the ‘curse of dimensionality’ and introduces stability issues associated with catastrophic forgetting. While these issues could, in principle, be mitigated by introducing a sufficiently large replay buffer during online training, the available hardware posed a limitation. Consequently, AI Controller 3 is more complex yet does not provide a substantial performance benefit over AI Controller 2, achieving the same average 30% improvement (Fig. 9A). Moreover, because Controller 2 does not exhibit catastrophic forgetting during online learning and reliably maintains high activity, it represents a more stable and practical solution for this system when using conventional laboratory equipment.

The AI controller can further be extended to target a specific product distribution, potentially resulting in a different control policy. However, establishing the required feedback loop would necessitate online product analysis, as well as a modified reward function. We hypothesize that the presented set of three potentials forms the minimal action space for effective control of the glycerol electrooxidation reaction. To target a specific product distribution, finer control may become necessary. Successfully implementing such control would require equipping the laboratory with a dedicated AI workstation.

### Physics-inspired AI model

Throughout the development of the AI controllers, AI models played an essential role by providing an offline environment in which virtual experiments could be conducted and pretrained models could be evaluated before their costly deployment in the laboratory. However, these AI models were based on a simple recurrent-neural-network architecture that, while accurate, offers limited interpretability and provides little mechanistic insight.^50,51^ To address this limitation, we developed a fully trainable physics-inspired AI model that embeds classical electrochemical equations directly into its structure.

This physics-inspired AI model incorporates a custom trainable layer that models surface kinetics through site-specific activity functions. This design is inspired by previous work on heterogeneous alloy catalysts, where the overall activity can be represented as a linear combination of the activities of individual active sites, as expressed in eqn (4).^52^ In this equation, *f*_*n*_ denotes the fractional coverage of site *n*, *j*_*n*,0_ is the exchange current density of site *n*, and *α*, *z*, *F*, *R*, and *T* retain their conventional electrochemical meanings within the Butler–Volmer equation. In alloy systems, active sites typically correspond to fixed elemental configurations, yielding a static site distribution. However, the same concept can be generalized by including adsorbates in the active-site definition. Unlike static elemental arrangements, adsorbates evolve dynamically during operation, making the active-site distribution a time-dependent property. Importantly, this generalized formulation can be applied to a monometallic platinum surface, enabling the glycerol electrooxidation reaction to be formulated as a linear combination of dynamically evolving active site contributions.4
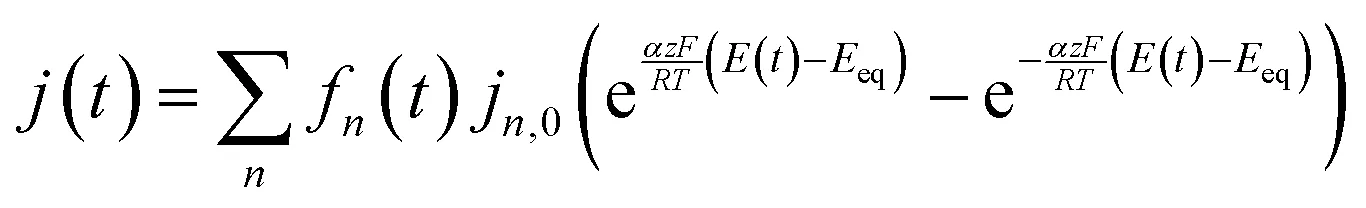


To implement this concept into a neural network, eqn (11) was reformulated into a fully differentiable Butler–Volmer layer described using eqn (5)–(12). This layer takes as an input the time-dependent active-site distribution and the applied potential and converts these into the resulting current density. Eqn (5) parameterizes the site-specific Butler–Volmer Equation using four learnable kinetic coefficients *β*_1_–*β*_4_. Here, *β*_1_ approximates the exchange current density, *β*_2_ corresponds to the inverse Tafel slope, *β*_3_ represents the equilibrium potential, and *β*_4_ governs the oxide-formation gate *σ*(*E*) defined in eqn (6). The oxide-formation gate deactivates glycerol-oxidation sites at high potentials where platinum oxide is formed, in line with experimental observations. To constrain these parameters to physically meaningful values while maintaining differentiability, tanh-based transformations (eqn (7)–(10)) were employed. These transformations restrict *β*_1_ to the e^−20^ to e^20^ range, *β*_2_ to values corresponding to Tafel slopes between 36 mV dec^−1^ and 2.3 V dec^−1^, *β*_3_ to 0.3–1.0 V *vs.* RHE, and *β*_4_ to 0.75–2.0 V *vs.* RHE. In addition, to stabilize the training and prevent runaway losses, a Koutecký–Levich-type expression was introduced (eqn (11)). This equation limited the oxidative currents to 51 mA cm^−2^ and the reductive limiting current to 10 mA cm^−2^, thereby preventing the model from exploiting non-existent reductive currents. Finally, using these expressions, the total current density is calculated as a linear combination of all the site-specific partial currents (eqn (12)).5
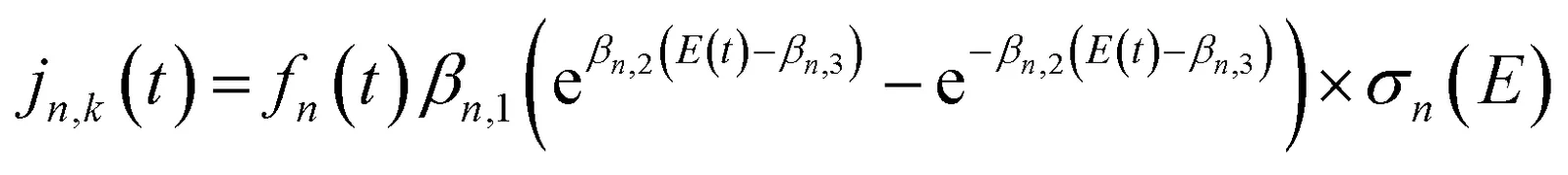
6
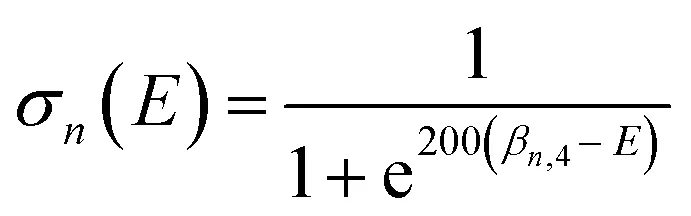
7
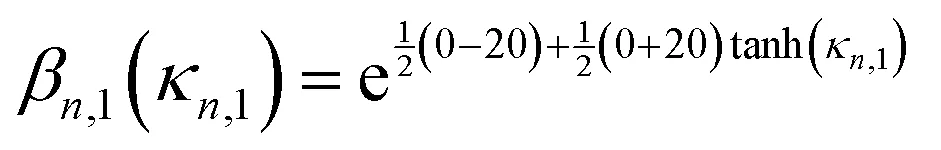
8
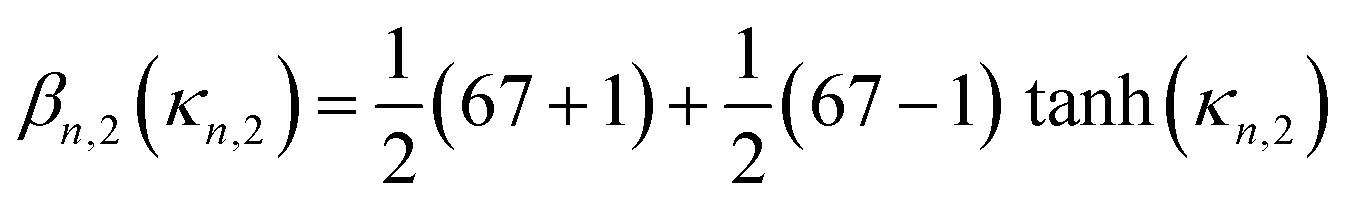
9
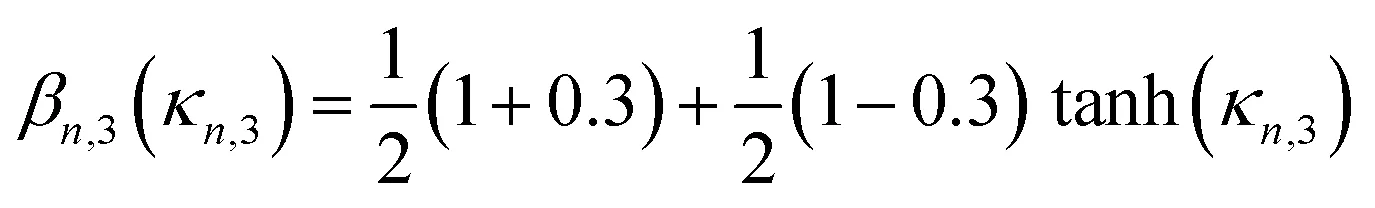
10
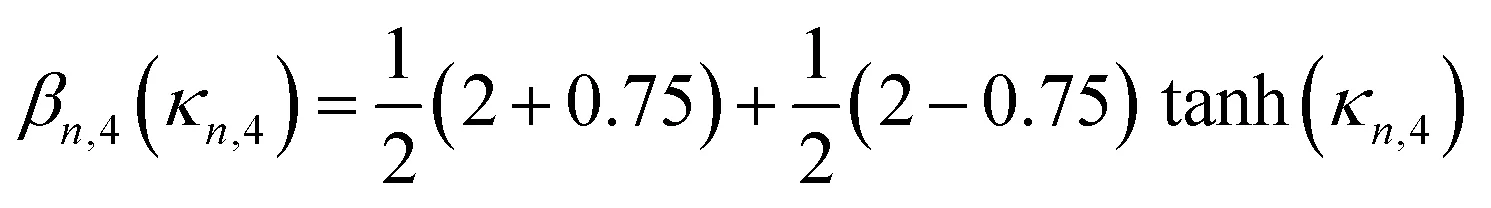
11
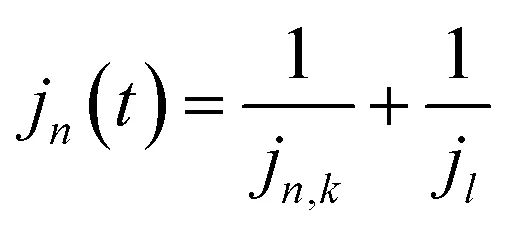
12
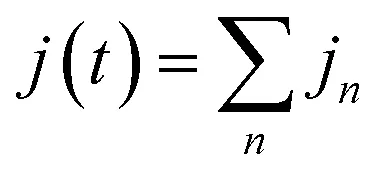


This Butler–Volmer neural layer was incorporated into an encoder–decoder architecture, as illustrated in Fig. 10, to create AI Model 2. At each timestep, the network receives the voltage, current, and hidden state (*E*(*t*), *j*(*t*), *h*(*t*)) which are passed through the encoder to produce the updated hidden state *h*(*t* + 1). This hidden state encodes the system's latent dynamics, including the evolving active-site distribution. The decoder then maps *h*(*t* + 1) onto an explicit virtual active-site distribution *F*(*t* + 1). In this work, the number of virtual active sites to describe the glycerol electrooxidation was arbitrarily set to eight. The predicted distribution *F*(*t* + 1) together with the potential applied at the next timestep *E*(*t* + 1), is subsequently passed through the Butler–Volmer layer to compute the next current value *j*(*t* + 1). The model then proceeds autoregressively, using (*E*(*t* + 1), *j*(*t* + 1), and *h*(*t* + 1)) as the input for the subsequent prediction with *E*(*t* + 2).

**Fig. 10:**
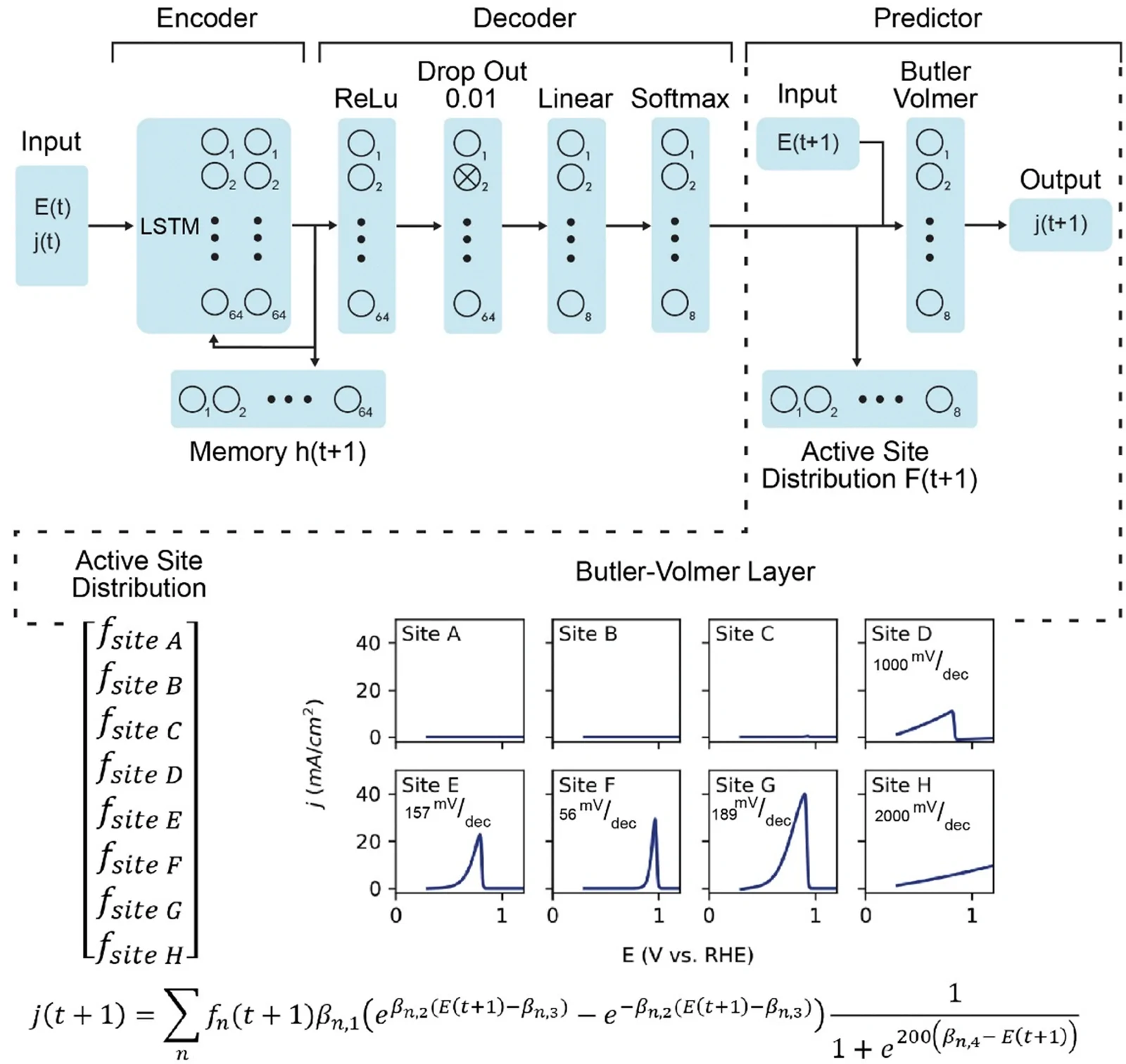
The architecture of the physics-inspired AI Model 2. The model was trained on experiments 1–11 and validated on experiment 12. The inset shows the site-specific activity functions learned for the eight virtual active sites with their corresponding Tafel slopes.

AI Model 2 was trained using the rich dataset generated during the development of the AI controllers, with experiments 1–11 serving as the training set. Prior to training, the entire dataset was corrected for solution resistance, which substantially reduced the inference error (Fig. S53 and S58). Experiment 12 was used for validation and achieved an MAE of 0.689 mA cm^−2^ for the entire dataset. As the training data are dominated by aged-cell dynamics, the validation errors differ between fresh-cell and aged-cell conditions. Experiment 12.1 in the fresh cell was simulated with an MAE of 1.6 mA cm^−2^, whereas experiment 12.13 in the aged cell was simulated with a substantially lower error of 0.516 mA cm^−2^ (Fig. 11). Considering additional sources of noise, such as variations in solution resistance, initial glycerol concentration, and timing uncertainties in the control script, an average MAE of 0.689 mA cm^−2^ represents a remarkably low inference error for this system.

**Fig. 11:**
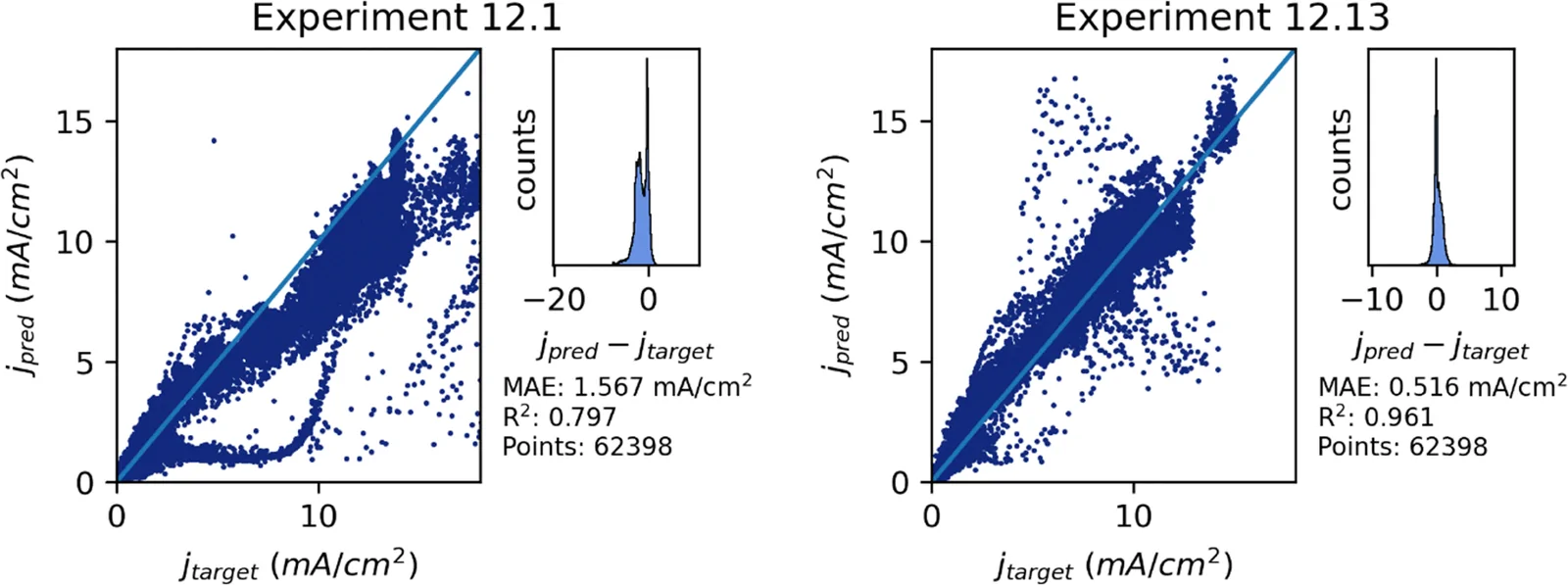
Parity plot of AI Model 2 comparing predicted and measured current densities obtained from free-running simulations of experiments 12.1 and 12.13. For each experiment, the first 600 data points were used to initialize the hidden state, after which the remaining 62 398 points were predicted autonomously. The inset shows the distribution of prediction errors.

With the trained and validated AI Model 2, the next step was to analyze the eight site-specific activity functions, which are shown in Fig. 10 (Table S1). These virtual active sites originate purely from mathematical fitting and are presently not calibrated to physical processes, although this could be achieved by incorporating additional data streams into the training. However, they provide an interpretable representation of the internal dynamics of AI Model 2, resulting in a grey-box model. The first 3 virtual active sites (A–C) exhibit effectively no current across the operating potential window, indicating that they function as inactive or mathematically auxiliary sites. In contrast, virtual sites D–G display clear activity functions that operate only at potentials below 1.0 V *vs.* RHE, resembling glycerol oxidation CVs^53,54^ (Fig. 3A), and are thus assigned to glycerol oxidation sites. Finally, virtual site H is the only site showing significant activity above 1.0 V *vs.* RHE, suggesting that it represents a bifunctional contribution: modelling glycerol oxidation at less positive potentials and platinum-oxide formation^55^ at more positive potentials.

As the model incorporates a physics-inspired Butler–Volmer layer, each virtual active site is associated with a corresponding Tafel slope, a parameter that is often used to infer reaction mechanisms. Subsequent analysis revealed that the model relies primarily on virtual glycerol-oxidation sites D and E, allowing sites F and G to be removed without a substantial loss in predictive performance (SI Section III.3). Virtual site D exhibits a large Tafel slope of 1000 mV dec^−1^, indicating that it primarily contributes a broad baseline current over a wide potential range. In contrast, virtual site E exhibits a Tafel slope of 157 mV dec^−1^, which is comparable to experimentally reported values for glycerol oxidation. Kwon *et al.* reported a Tafel slope of approximately 120 mV dec^−1^ in the low-overpotential regime of glycerol electrooxidation on platinum,^18^ whereas Habibi *et al.* reported a value of 177 mV dec^−1^ for Pt nanoparticles.^56^ In this work, we observed an apparent Tafel slope of 148 mV dec^−1^ under fresh-cell conditions (Fig. S4) and values of 115 and 146 mV dec^−1^ under aged-cell conditions (Fig. 12).

**Fig. 12:**
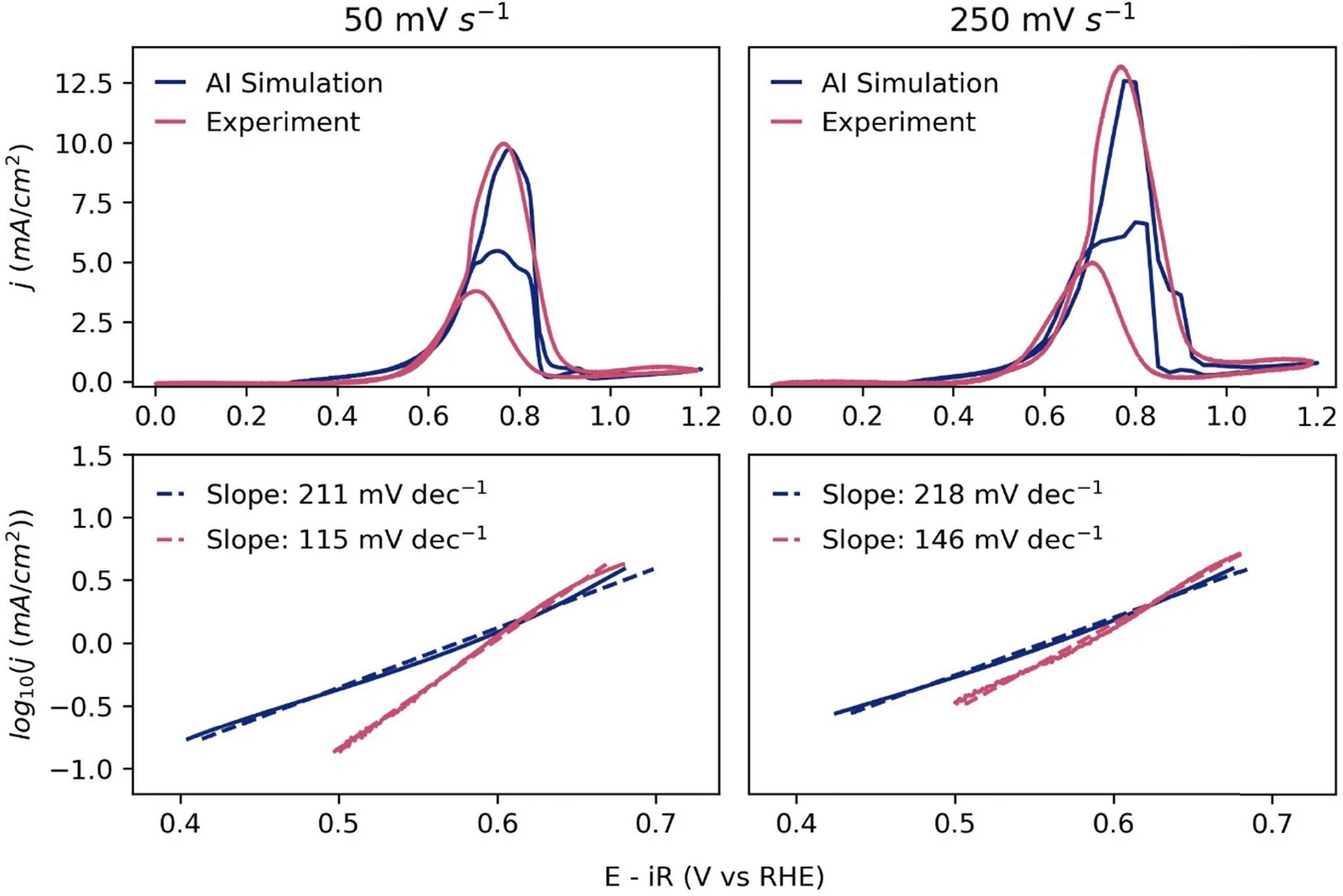
Simulated and experimental CVs obtained using AI Model 2 at scan rates of 50 and 250 mV s^−1^. The experimental CVs correspond to aged-cell conditions and were recorded at the end of experiment 3 in 0.1 M KOH and 0.1 M glycerol (Ar-saturated) at 200 rpm. The working electrode was a platinum disk, with a platinum mesh counter electrode in the same compartment and an Ag/AgCl reference electrode. The lower panel shows the corresponding Tafel analysis of the forward scan, with the solid line representing the data and the dashed line the linear fit.

The wide range of apparent Tafel slopes reported for glycerol electrooxidation reflects the inherent complexity of the reaction. Specifically, glycerol electrooxidation proceeds through a complex network of competing reaction pathways and poisoning mechanisms. Consequently, the reaction does not exhibit a well-defined Tafel regime that would permit the assignment of a specific reaction mechanism to an observed apparent Tafel slope. Moreover, the activity functions identified by AI emerged purely from mathematical fitting and were not calibrated against product distributions. As such, the modeled virtual active sites cannot be assigned to specific reaction mechanisms beyond a general association with the glycerol electrooxidation reaction.

A major test of AI Model 2 was its ability to simulate CVs of the glycerol oxidation reaction at scan rates of 50 and 250 mV s^−1^ (Fig. 12). This represents a challenging out-of-domain task because neither the training nor validation datasets contained CV measurements. Moreover, the model was trained exclusively on experiments with voltage steps of multiples of 50 mV each lasting for at least 100 ms. In contrast, CVs are typically implemented as staircase waveforms with potential increments of approximately 1 mV, where each step can persist for only a few milliseconds. Consequently, CV simulation requires the AI model to operate on both voltage values and timescales that were absent from the training dataset.

The AI model simulated the forward scan with a high degree of similarity to the experimental CVs, correctly predicting the height, width and location of the oxidation peak. The reverse scan proved more challenging, with the model predicting an artificial feature near 0.8 V *vs.* RHE that is absent in the experimental data. We hypothesize that this artifact arises from insufficient training data at highly oxidizing potentials. During autonomous operation, the AI controllers generally avoided extensive oxidation of the platinum surface, resulting in a limited representation of these dynamics in the training set. Consequently, this discrepancy could likely be mitigated by incorporating additional data collected at positive potentials into the training procedure.

To further evaluate the AI model, a Tafel-slope analysis was performed on both the simulated and experimental voltammograms. The simulated CVs exhibit apparent Tafel slopes of approximately 210 mV dec^−1^. This result highlights that, although individual active sites within the model are characterized by discrete fixed Tafel slopes, the evolving active-site distribution can give rise to a different apparent Tafel slope during simulations. In comparison, the experimental CVs exhibit apparent Tafel slopes of 115 and 146 mV dec^−1^ at scan rates of 50 and 250 mV^−1^, respectively. This difference is primarily caused by a background current predicted by the AI in the potential window of 0.4–0.5 V *vs.* RHE. We similarly hypothesize that this artifact is caused by insufficient sampling of transitions within the relaxation-potential regime.

Overall, despite these discrepancies, AI Model 2 demonstrates the ability to generalize beyond its training domain. This finding indicates that the model has extracted fundamental kinetic information from experiments conducted under arbitrary and non-periodic pulsing conditions. These kinetics are currently encoded within virtual active sites and require additional research and data streams for physical interpretation. Nevertheless, these results highlight the strong potential of the physics-inspired AI framework to further advance our understanding of kinetics under complex, dynamic, and non-periodic operating conditions.

The interpretable structure of AI Model 2 was further used to investigate the dynamics of the platinum surface under AI operation and under the three-step policy (Fig. 13). Under AI control, the system is primarily pulsed between 0.3 and 0.85 V, with occasional pulses to 1.0 V. The virtual inactive sites A–C oscillate between approximately 30 and 40% in response to these pulses. The virtual active sites D–G reveal that the AI model and controller rely predominantly on sites D and E, while sites F and G contribute only minor adjustments that fine-tune the predicted current. Thereby, site E exhibits a higher intrinsic activity than site D, as reflected in its Butler–Volmer parameters. When the potential is stepped to 0.85 V, the fraction of site E rapidly decreases and is replaced by site D and inactive sites A–C. The subsequent relaxation pulse to 0.3 V reverses this trend, converting a fraction of site D back to site E. Notably, the controller tends to apply this relaxation pulse when the coverage of site E approaches low values, avoiding extended operation with site E fully depleted. Over time, the recovery of site E becomes slower, prompting the controller to apply a pulse to an oxidative potential. This oxidative pulse temporarily restores site E and enhances the efficacy of the subsequent relaxation steps at 0.3 V, making these pulses essential for extended operation. Virtual site H remains relatively stable throughout the simulation but decreases in response to oxidative pulses. This is consistent with its assignment as the oxide-formation site, whose contribution diminishes when an oxide layer is formed.

**Fig. 13:**
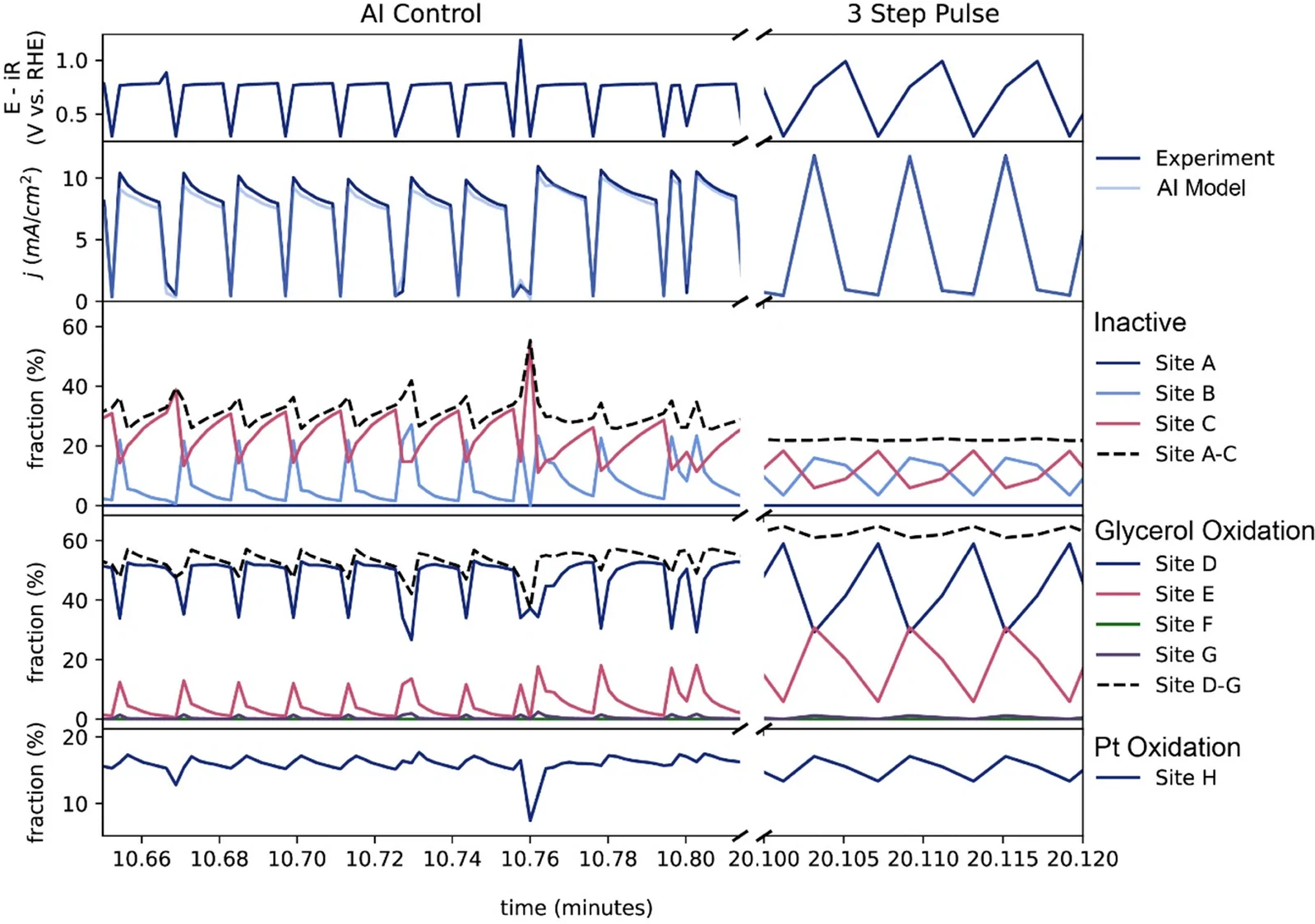
Zoomed-in view of the simulation of experiment 12.13, showing the experimental and simulated current density alongside the model-predicted active-site distributions at the corresponding timesteps. Virtual sites A–C are inactive or auxiliary sites. Virtual sites D–G represent glycerol-oxidation sites. Virtual site H contributes to glycerol oxidation at low potentials and to platinum oxide formation at higher potentials.

In comparison, the three-step policy is performing excessive reactivation of the surface. Consequently, the combined fraction of virtual inactive sites A–C is lower and more stable than that under AI control. At the same time, excessive reactivation ensures that a large fraction of virtual site E is available during the glycerol-oxidation step at 0.85 V. As a result, the three-step policy preferentially exploits virtual site E, whereas the AI controller relies more strongly on virtual site D. Under the unverified assumption that virtual sites D and E correspond to different reaction pathways, this divergence suggests that the two policies may yield different product distributions. Verifying this hypothesis, however, remains a subject for future investigation.

In its present form, the identified active sites are model-derived constructs and have not been verified to correspond to specific physical properties of the system. Nevertheless, the physics-inspired network introduced here provides a flexible framework for analyzing complex electrochemical reactions under non-periodic pulsing conditions, beyond the glycerol electrooxidation reaction. For example, with appropriate calibration, the decoder could be constrained to reproduce active-site distribution consistent with adsorbate coverages measured using *in situ* spectroscopy^31,32,57^ or theoretically computed.^58,59^ Likewise, when supplied with time-resolved product-distribution data,^60–62^ the Butler–Volmer layer could be adapted to predict partial current densities associated with individual reaction pathways. Taking together, these capabilities offer a route toward integrating mechanistic insight, spectroscopy, theoretical modelling, and reaction-product analysis within a single interpretable AI modelling framework.

## Conclusion

Summarizing, this work demonstrated a complete development cycle for a reinforcement-learning-based controller capable of autonomously performing electrochemical experiments. Beginning with a simple kinetic model as an entry point, the workflow progressively incorporated experimental data and AI model feedback to refine and improve the AI controller. Applied to glycerol electrooxidation, this approach led to the discovery of a control strategy that outperformed the engineered three-step policy by approximately 30%. The AI-derived policy and its accompanying analysis revealed that three potentials constitute a minimal action space for the effective control of the glycerol electrooxidation reaction. While demonstrated here for glycerol electrooxidation, AI-based dynamic control can be extended to a broad range of electrochemical systems to identify non-periodic operating regimes that enhance energy efficiency, conversion rate, selectivity, and operational stability.

Moreover, the rich datasets collected during controller training enabled the construction of a physics-inspired AI model that combines an encoder–decoder architecture with a trainable Butler–Volmer layer. This interpretable model provides, at each timestep, a distribution of active sites with known activity functions, offering mechanistic insights into the system's dynamics. In this study, it was used exclusively in combination with electrochemical data to elucidate the behavior of the glycerol electrooxidation reaction under AI-derived and engineered policies. Importantly, this AI modelling framework also offers a general and extensible platform for analyzing complex non-periodic pulsing behavior, enabling the systematic study of dynamic regimes in fuel cells and electrolyzers that are difficult to resolve using conventional analysis approaches. Beyond electrochemical data alone, the AI framework can integrate additional data streams from theoretical calculations, product-distribution measurements, and spectroscopic techniques, thereby unifying complementary sources of information within a single model. The resulting AI model forms an interpretable bridge between idealized model systems and experimentally noisy laboratory environments, supporting not only mechanistic understanding but also practical applications such as digital twinning and diagnostics of electrolyzer health. Together with the AI controller, this work provides a pathway for AI to accelerate the discovery, mechanistic understanding, and optimization of electrochemical processes.

## Conflicts of interest

There are no conflicts of interest to declare.

## Supplementary Material

EE-019-D6EE02239A-s001

EE-019-D6EE02239A-s002

## Data Availability

All data generated, as well as scripts used in this work are available at: https://zenodo.org/records/21035955. All scripts are also available at: https://github.com/vamints/Scripts_AI_Driven_Electrocatalysis_RL_Glycerol. A ready-to-use Butler–Volmer Layer in PyTorch is available at: https://github.com/vamints/electrochem-ml-layers. Supplementary information (SI): experimental methods, AI controller and model development, supplementary figures, supporting datasets, and model validation results. See DOI: https://doi.org/10.1039/d6ee02239a.
