# Supplementary material for: AI-driven electrocatalysis: reinforcement learning-based control and theoretical modeling of the glycerol oxidation reaction
Source: Energy Environ Sci. 2026 Oct 5;19(21):7377–93. doi: 10.1039/d6ee02239a (PMC13635933; doi:10.1039/d6ee02239a)
Supplement: Supplementary file 3 [file EE-019-D6EE02239A-s001.pdf]

Supplementary Information (SI) for Energy & Environmental Science.
This journal is © The Royal Society of Chemistry 2026
Supplementary Information (SI) for Energy & Environmental Science.
This journal is © The Royal Society of Chemistry

Supporting Information to:

AI-Driven Electrocatalysis: Reinforcement Learning-Based Control and Theoretical Modeling of the Glycerol Oxidation Reaction

Vladislav A. Mints*, Maria-Magdalena Titirici, Ifan E. L. Stephens

Corresponding author: vladislav.mints@empa.ch

Data Availability

All data generated, as well as scripts used in this work are available at:
https://zenodo.org/records/21035955

All scripts are also available at:
https://github.com/vamints/Scripts_AI_Driven_Electrocatalysis_RL_Glycerol

A ready-to-use Butler-Volmer Layer in Pytorch is available at:
https://github.com/vamints/electrochem-ml-layers

I. Experimental

All electrochemical measurements were conducted at room temperature in a three-electrode, a in house-made Teflon cell with a Pine WaveVortex rotating disk electrode (RDE) and controlled using a Biologic SP-300 potentiostat. Before each experiment, the Teflon cell and all plastic volumetric flasks and bottles were thoroughly cleaned by performing at least ten rinses with ultrapure water (18.2 MΩ·cm, Sartorius Arium Pro), followed by 30 minutes of sonication.

A platinum disk (⌀ 0.5 cm, 0.195 cm^2) was used as the working electrode and polished with 0.05 µm alumina slurry (BAS Inc.) before each experiment. A flame-annealed platinum mesh was used as the counter electrode in the same compartment. The reference electrode was a leakless Ag/AgCl electrode (LF-2-100, Innovative Instruments) calibrated against the hydrogen oxidation/evolution reaction (HOR/HER) on platinum. The initial calibration yielded a value of –0.917 V vs reversible hydrogen electrode (RHE).

Prior to the final experiment, the Ag/AgCl electrode was recalibrated in the 0.1 M KOH saturated with H_2 (110971-F, BOC) by purging for 10 minutes, followed by cyclic voltammograms (CVs) recorded at 900 rpm with a scan rate of 5 mV s^{-1} and no iR-compensation. The recalibration exhibited a drift of 7 mV from the initial value. Because this deviation was negligible relative to the ohmic-drop induced shifts, the initial calibration was used throughout all experiments.

The electrolyte consisted of 0.1 M KOH (99.995%, Merck Suprapur). Before each experiment, the solution was purged with Ar (270050-L, BOC) while the platinum working electrode was electropolished by cycling between 0 and 1.2 V vs RHE at 250 mV s^{-1} in 200 mL of electrolyte at 1600 rpm. After electropolishing, 5-10 CVs were recorded between 0 and 1.2 V vs RHE at 50 mV s^{-1} and 0 rpm to characterize the Pt surface. Once a representative and stable CV was obtained, 1.5 mL of glycerol (99.5%, Sigma-Aldrich) was added, producing a final volume of ~201.5 mL and a concentration of 0.1 M glycerol. The solution was stirred at 2500 rpm for 10 minutes using the RDE to ensure complete dissolution.

Electrochemical impedance spectroscopy (EIS) was then performed at open-circuit potential, between 10 kHz to 10 Hz and an amplitude of 5 mV. Due to the high resistivity of the Ag/AgCl electrode, the high frequency data points contained artifacts. Therefore, the value at minimum imaginary resistance was taken as the solution resistance. An additional set of 10 characterization CVs between 0 and 1.2 V vs RHE at 50 mV s^{-1} were then collected at 200 rpm. All characterization CVs, together with the Nyquist plots, are provided in Section IV of the Supporting Information.

After the characterization stage, the Python-based AI experiment was performed at a fixed rotation rate of 200 rpm with a small Ar flow of 5 mL min^{-1} into the solution. All python scripts, AI controller and AI models, in this work used raw currents *i* is inputs and outputs.

These were corrected by the electrode geometry 0.195 cm^2 to current densities j in the manuscript.

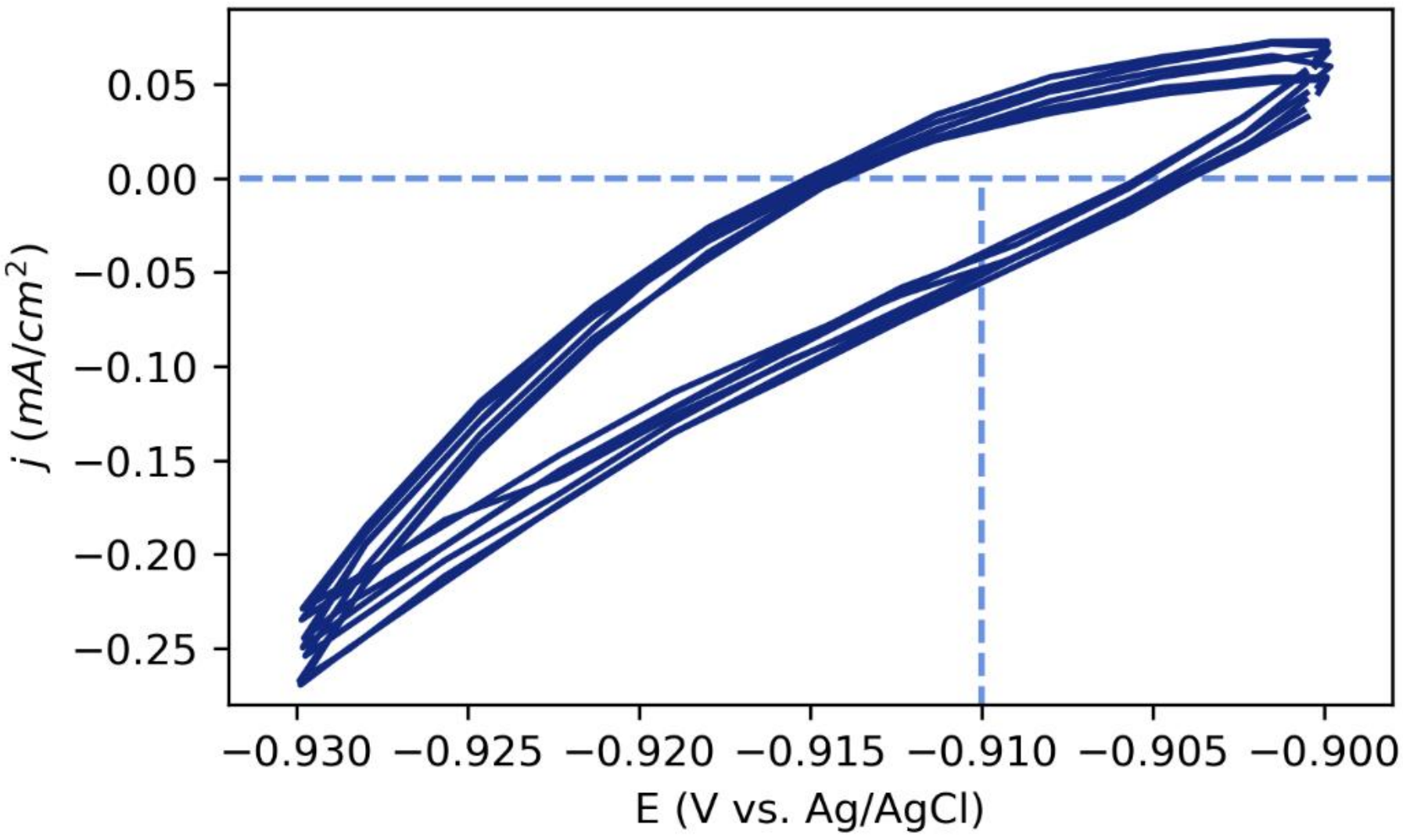

Figure S1: *Calibration of the Ag/AgCl electrode after experiments. Pt in 0.1 KOH, H_2 saturated, at 5 mV s^{-1} and 900 rpm.*

II. AI controller Training

II.1 AI Controller Software

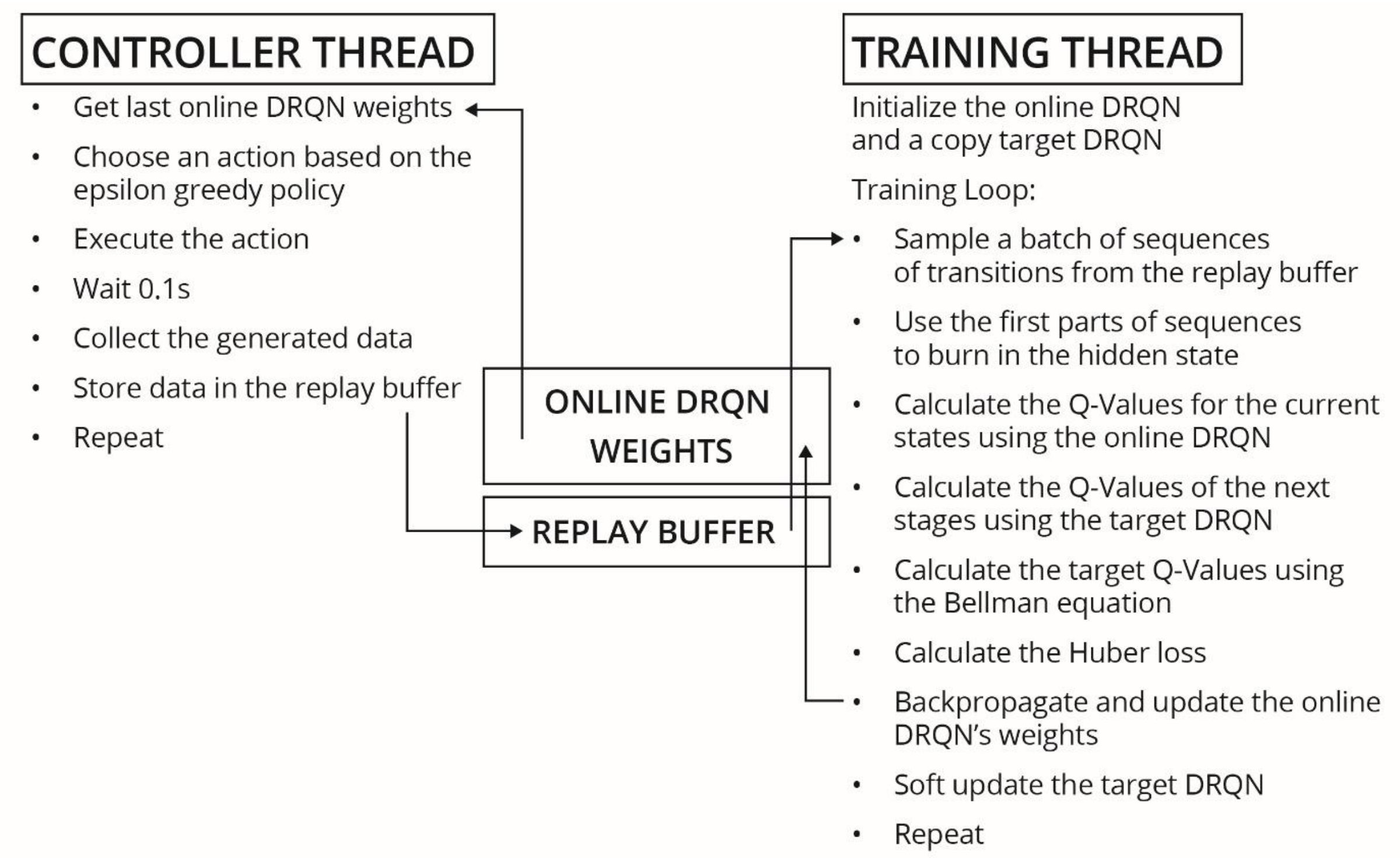

Figure S2: *A schematic describing the software behind the AI controller.*

The AI-controllers used in this work were implemented as variants of deep recurrent Q-networks (DRQN) equipped with long short-term memory (LSTM) layers.[1] The input to these networks is a sequence of observations preceding the moment of decision and predict Q-values for the available actions. The Q-values correspond to the immediate reward and expected return when following the optimal policy thereafter. All AI-based experiments were executed through a custom multithreaded Python program using PyTorch 2.1[2], illustrated schematically in Figure S2.

The program operates using two concurrently running threads that continuously exchange information. The first thread handles real-time control and data acquisition. Every 100 ms, it synchronizes the active DRQN with the version currently being trained and selects the next action using an ε-greedy policy. A random number between 0 and 1 is drawn; if the value is less than ε, the controller executes an exploratory action prescribed by the uniformly random policy (π_{random}); otherwise, above ε, it follows the DRQN's earned policy (π_{AI}). To do so, the DRQN hidden state is reset and reinitialized using the latest sequence of observations, with the sequence length matching the training configuration (Note, it uses the sequence length and not the burn in length). The controller then evaluates the Q-values of the final state in the sequence and applies the potential with the highest Q-value to the electrochemical cell. After the 100 ms control

interval, the thread collects the instrument data sampled at 25 ms intervals, writes the raw data to the disk and stores the final point of this interval in the replay buffer for training, which collects all observations of the ongoing experiments (Figure S3). Finally, the thread updates the ε value according to the scheduling ruleset, which differed per experiment. The exact ε trajectories can be found in section IV of the Supplementary Information.

The second thread trains the DRQN continuously during the experiment. To improve training stability, the implementation employs a Double Q-learning framework in which an online network and target network operate together.[3] Each training step begins by sampling a batch of sequences from the replay buffer using uniform random sampling. Due to hardware limitations of the laboratory workstation (HP EliteDesk 800, Intel i9-9700, 8 GB DDR4 RAM), these real-time experiments used a batch size of one sequence. The initial portion of each sequence is used to burn in the hidden state of the DRQN, while the remaining observations are used to update the network parameters. The Huber loss serves as the objective function and the training is performed using the Adam optimizer with default PyTorch parameters ($\beta_1 = 0.9$, $\beta_2 = 0.999$), a learning rate of 10^{-3}, and gradient clipping at a maximum norm of 1 to prevent runaway gradients. To further stabilize learning, the target network is updated using τ-soft updates with τ between 0.005 and 0.01.[4] The exact neural network architecture used at each experiment, along with the hyperparameters, sequence length, burn in window, gamma, reward function, are listed in section IV of the Supporting Information.

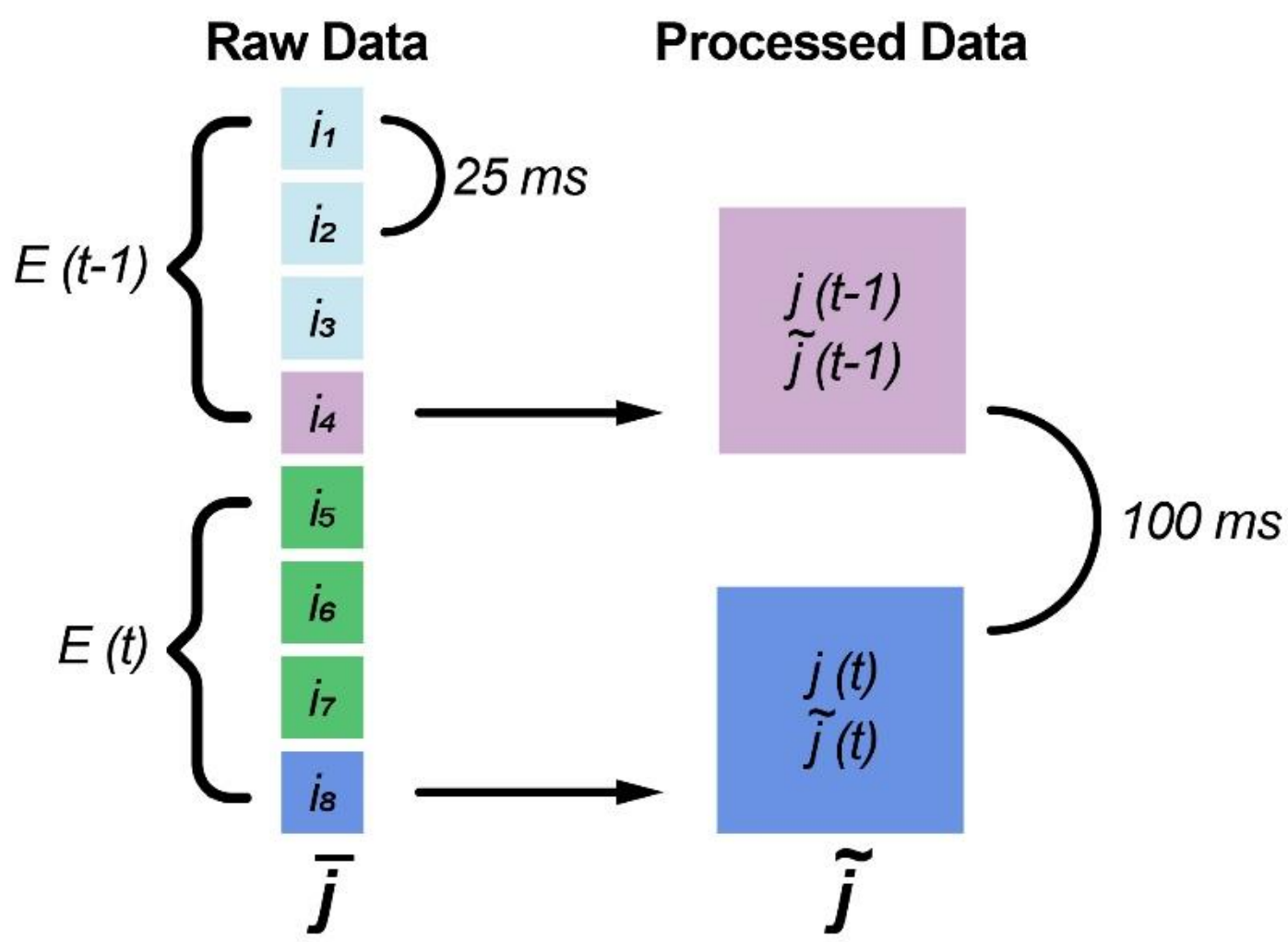

Figure S3. *Data processing flow. The raw data was sampled at 25 ms intervals and used to calculate the true average. The processed data was used to control the electrolysis, train the AI controller, AI models and calculate the exponentially moving average.*

II.2 Kinetic Model

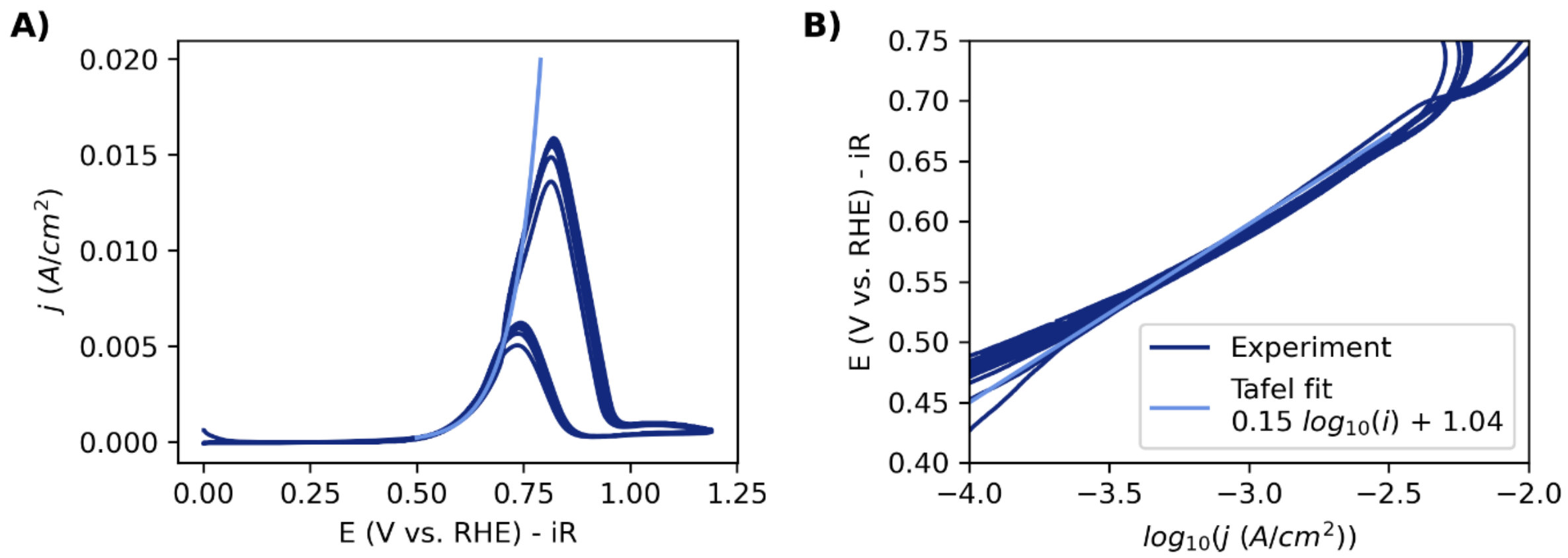

***Figure S4**. **A)** Glycerol oxidation on Pt in 0.1 M KOH, 0.1 M Glycerol at 50 mV s^{-1} and 200 rpm. **B)** Tafel fit of the glycerol oxidation.*

Before deploying the AI controller in the laboratory, its performance was first evaluated and benchmarked using a simple kinetic model defined by Eq. S1-S8. In this model, the platinum surface is represented by three surface fractions, θ_{oxide}, $\theta_{poisoned}$, θ_{active} which sum to unity. It's starting conditions assumed that the surface is fully active with θ_{active}=1.

Eq. S1 and S2 describe the rates of oxide formation and reduction. In these equations, k_{Ox} and k_{red} are both set to 4•10^{-9} mol s^{-1}, while k_b and T denote to the Boltzmann constant and a temperature of 298 K, respectively.[5] *E* is the applied potential in V vs. RHE. A site density of 2.18×10^{-9} mol cm^{-2} was used to convert molar rates into fractional coverages. The time-dependent change in the oxide coverage is given by Eq. S3, while Eq. S4 converts the oxide formation and reduction rates into a current density using the Faraday constant F.

The glycerol oxidation current density is described by Eq. S5. Here, the exchange current density j_0, equal to e$^{-16.2}$, and the Tafel slope of 148 mV per decade were obtained by fitting the Pt characterization CV from Experiment 1. This CV was recorded in 0.1 M KOH and 0.1 M glycerol at 200 rpm and a scan rate of 50 mV s^{-1} (Figure S3). Equation S6 describes surface poisoning, assuming that 0.5 % of the glycerol oxidation current leads to irreversibly adsorbed species that can be oxidatively stripped. Equation S7 governs the change in θ_{active}, and Eq. S8 provides the total current density as the sum of all partial current densities.

The system was integrated using a fixed-time-step solver with a time step of 1 ms. During pulse simulation, each 100 ms control interval was represented by 100 sub-steps of 1 ms.

$$v_{ox} = \begin{cases} k_{Ox}(1-\theta_{oxide})e^{\frac{0.5(E-0.79)}{k_bT}} & if\ E > 0.81\ V \\ 0 & else \end{cases} \qquad \text{(S1)}$$

$$v_{red} = \begin{cases} k_{red}\theta_{oxide}e^{\frac{0.5(E-0.79)}{k_bT}} & if\ E < 0.79\ V \\ 0 & else \end{cases} \quad \text{(S2)}$$

$$\frac{d\theta_{oxide}}{dt} = \frac{v_{ox}-v_{red}}{2.18*10^{-9}} \quad \text{(S3)}$$

$$j_{ox} = d\theta_{oxide} * 2 * F \quad \text{(S4)}$$

$$j_k = j_0\theta_{active}\, e^{15.5E} \quad \text{(S5)}$$

$$\frac{d\theta_{poisoned}}{dt} = \frac{0.005 j_k}{F} \quad \text{(S6)}$$

$$\frac{d\theta_{active}}{dt} = -\frac{d\theta oxide}{dt} - \frac{d\theta_{poisoned}}{dt} \quad \text{(S7)}$$

$$j = j_{ox} + j_k \quad \text{(S8)}$$

Oxide formation and reductions were restricted to non-overlapping potential windows. Oxide formation occurs only above 0.81 V, while oxide reduction occurs only below 0.79 V. These constraints were introduced so that the model mathematically favours the proposed three-step pulsing protocol.[6]

For example, if oxide formation is allowed at 0.8 V during glycerol oxidation, both the active and poisoned surface fractions would be consumed by oxide growth, which could then be fully removed by stepping to 0.3 V. Under these conditions, a simple two-step pulse between 0.8 and 0.3 V would become optimal. Likewise, if oxide reduction occurred at 0.8 V, a single oxidative pulse would suffice to reactivate the system Therefore, by restricting oxide formation and reduction to potentials outside the probed glycerol oxidation potential, the model will produce a three-step pulsing protocol.

II.3 AI Controller V1, three step pulsing

AI controller V1 was used to optimize the three-step pulsing proposed in earlier work.[6] This protocol involves applying a potential for glycerol oxidation, followed by an oxidative pulse at 1.0 V vs. RHE and concluding with a relaxation pulse at 0.3 V vs. RHE. From the characterization CV the model is based on (Figure S3), the optimal iR-corrected potential for glycerol oxidation was determined to be 0.8 V vs. RHE, which produced absolute currents of approximately 2-3 mA. In the experimental setup, however, the solution resistance was around 40 Ω, requiring a higher uncompensated potential to reach it. Therefore, during deployment the glycerol oxidation potential was set to 0.85 V vs. RHE, and the discrete action space consisted of the three potentials 0.3, 0.85 and 1.0 V vs. RHE.

The input space of the AI Controller V1 included the applied potential, the measured current, and an exponentially moving average of the current. The latter is denoted by a tilde (~) and calculated according to Eq. S9. Because the three discrete potentials lead to distinct electrochemical responses, the applied potential was one-hot encoded to avoid unintended numerical correlations, which resulted in a five-element input vector.

$$\tilde{i}_\lambda(t) = \lambda\, i(t) + (1 - \lambda)\, \tilde{i}_\lambda(t - 1) \qquad \text{(S9)}$$

II.4 AI Controller V2

AI Controller V2 was developed to operate over a more continuous action space ranging from 0.3 to 1.2 V vs. RHE. This range was discretized in 50 mV increments, resulting in a 19-element action space. Because these potentials are uniformly spaced, the applied potential was treated as a numerical input, thus was not one-hot encoded. In addition, the random policy was modified to introduce a bias toward glycerol oxidation potentials, yielding a center biased random policy ($\pi_{random-2}$). In the three-element action space of Controller V1, the probability of selecting the glycerol-oxidation potential under a uniform random policy is 1/3. In contrast, within the expanded 19-element space, only four potentials between 0.7 and 0.85 V correspond to active glycerol-oxidation conditions, giving an initial uniform selection probability of 4/19. To compensate for this reduction and steer the controller toward catalytically relevant potentials, each of these four actions was assigned an increased probability of 0.17. This resulted in a combined probability of 0.64 for selecting a potential within the 0.7-0.85 V region. The residual 15 actions had a uniform probability distribution which summed to 0.36.

Several experiments were conducted with online iR compensation enabled. However, the feedback loop associated with the iR compensation introduced additional noise, producing oscillatory currents even under nominally constant potential. These current oscillations were large enough to be detected by the DRQN and were likely to interfere with the learning process. Consequently, online iR compensation was disabled in the final and control experiments, and the AI controller was instead trained to operate in presence of this noise source.

II.5 AI Controller Offline Training

Between laboratory experiments, the AI controllers were further trained offline using all previously collected data sets. This offline procedure employed the exact same training loop as the deployed online controller; however, instead of receiving data from the live controller thread, the replay buffer was initialized with the entire offline experimental data set. Offline training was carried out on an ASUS TUF A15 laptop equipped with an RTX 4060, CUDA 13.0, a Ryzen 7 7735HS and 16 GB of DDR5 RAM. Unlike the laboratory hardware, this setup could use batch sizes of up to 128, sequence lengths of 1800 and burn-in lengths of 600. Throughout training, checkpoints were saved manually and evaluated within the AI-model environments until a satisfactory policy emerged. Moreover, based on checkpoint performance, the learning rate and batch sizes were adjusted manually.

During offline training, AI Controller V2 exhibited consistent tendency to overestimate Q-values for observations not encountered during live-training. An expected consequence of operating within a substantially large action space. To mitigate this issue and improve the performance, a Conservative Q-Learning (CQL) scheme was incorporated into the offline training protocol, using α value of 0.2.[7]

III. AI Model Training

III.1 AI Model V1 – RNN based

The datasets obtained during the operation of AI controllers were also used to train AI models of the electrochemical system. The first-generation of AI models employed recurrent neural-network architectures equipped with an LSTM layer. At each timestep *t*, the network received the measured current *i(t)*, the applied potential *E(t)* and the potential to be applied in the next timestep *E(t+1)*, and was trained to predict the corresponding next-step current *i(t+1)*. For AI models constructed for the three-element action space, the potentials were one-hot encoded in the same manner used for training AI Controller V1. The final architectures of these models and their performance metrics are provided in Section V of the Supporting Information.

Because the AI model must execute inference autoregressively, using its own predictions as inputs at future timesteps, it was trained using a teacher-forcing approach. In this teacher-forcing method, the model is periodically guided by replacing its own predicted currents with the true experimental values. The frequency of these replacements is governed by a teacher-forcing ratio, where 1 corresponds to training entirely only on true data and 0 corresponds to a fully free-running simulation.

Each training step consisted of sampling a batch of sequences. The initial portion of each sequence was used to initialize the hidden state of the LSTM layer. After the remaining observations were used to update the model parameters under the teacher-forcing regime. The objective function was the mean-squared error (MSE), and optimization was performed using the Adam optimizer with default PyTorch parameters ($\beta_1 = 0.9$, $\beta_2 = 0.999$) and a learning rate of 10^{-3} to 10^{-5}.

The training process followed an overarching scheduler in which the teacher-forcing ratio was gradually decreased from one to zero, while the sequence length was increased to ensure that the AI model learned to maintain a stable simulation over long trajectories. Checkpoints were saved every 500 training steps and cross-validated with a test set. The teacher-forcing scheduler, sequence length and learning rate were adjusted manually based on cross validation scores to direct the training process. Therefore, no exact scheduler is available for these trainings. In the end, the checkpoint with the best cross-validation performance was selected for use in simulations and in the manuscript.

III.2 AI Model V2 – Physics Inspired

Inspired by previous work on alloy catalysts[8], a second generation of AI models was developed using neural layers specifically designed to mimic electrochemical behavior. The underlying idea is that an electrocatalytic surface can be represented as a sum of distinct active sites, each characterized by its own activity function. This activity can be approximated by a Butler-Volmer-type expression, given in Eq S10, where f_n is the fraction of active site n, $j_{n,0}$ is the exchange current density of site n, and α, z, F, R, T retain their conventional electrochemical meanings. The potential applied at timestep t is denoted $E(t)$ and E_{eq} represents the equilibrium potential.

$$j(t) = \sum_n f_n(t) j_{n,0} \left(e^{\frac{\alpha z F}{RT}(E(t)-E_{eq})} - e^{-\frac{\alpha z F}{RT}(E(t)-E_{eq})} \right) \quad \text{(S10)}$$

To incorporate this behavior into a neural-network architecture, the Butler-Volmer expression was reformulated into a trainable layer, shown in Eq. S11. The coefficients β_1, β_2, β_3, and β_4 are learnable parameters: β_1 approximates the exchange current density, β_2 corresponds to the inverse Tafel slope, β_3 represents the equilibrium potential, and β_4 governs the oxide-formation gate $\sigma(E)$ defined in Eq. S12. This gating function is intended to deactivate sites at potentials where oxide formation becomes significant, reflecting the experimental observation that sites active for glycerol oxidation become inactive once the oxide layer starts to form.

$$j_{n,k}(t) = f_n(t)\beta_{n,1}\left(e^{\beta_{n,2}(E(t)-\beta_{n,3})} - e^{-\beta_{n,2}(E(t)-\beta_{n,3})}\right) * \sigma_n(E) \quad \text{(S11)}$$

$$\sigma_n(E) = \frac{1}{1+e^{200(\beta_{n,4}-E)}} \quad \text{(S12)}$$

The coefficients β_1 to β_4 must remain within physically meaningful domains. However, directly clipping them would remove gradients and make the layer untrainable. Therefore, their domains were imposed by using tanh-based transformation (Eqs. S13–S16) in which κ_1- κ_4 are the real trainable parameters. Through these transformations, β_1 was restricted to the range e^{-20} to e^{20}, β_2 to values corresponding to Tafel slopes between 36 mV dec^{-1} and 2.3 V dec^{-1}, β_3 to 0.3–1.0 V vs RHE, and β_4 to 0.75–2.0 V vs RHE. However, the Butler–Volmer expression introduces an exponential dependence that can easily generate runaway gradients. Therefore, a Koutecký–Levich-type expression (Eq. S17) was introduced to cap the oxidative limiting current at 51 mA cm^{-2} and the reductive limiting current at 10 mA cm^{-2}, preventing the model from relying on non-existent reductive currents. The total current density is then obtained by summing all partial currents as shown in Eq. S18.

$$\beta_{n,1}(\kappa_{n,1}) = e^{\frac{1}{2}(0-20)+\frac{1}{2}(0+20)\tanh(\kappa_{n,1})} \quad \text{(S13)}$$

$$\beta_{n,2}(\kappa_{n,2}) = \frac{1}{2}(67+1) + \frac{1}{2}(67-1)\tanh(\kappa_{n,2}) \quad \text{(S14)}$$

$$\beta_{n,3}(\kappa_{n,3}) = \frac{1}{2}(1 + 0.3) + \frac{1}{2}(1 - 0.3)\tanh(\kappa_{n,3}) \quad \text{(S15)}$$

$$\beta_{n,4}(\kappa_{n,4}) = \frac{1}{2}(2 + 0.75) + \frac{1}{2}(2 - 0.75)\tanh(\kappa_{n,4}) \quad \text{(S16)}$$

$$j_n(t) = \frac{1}{j_{n,k}} + \frac{1}{j_l} \quad \text{(S17)}$$

$$j(t) = \sum_n j_n \quad \text{(S18)}$$

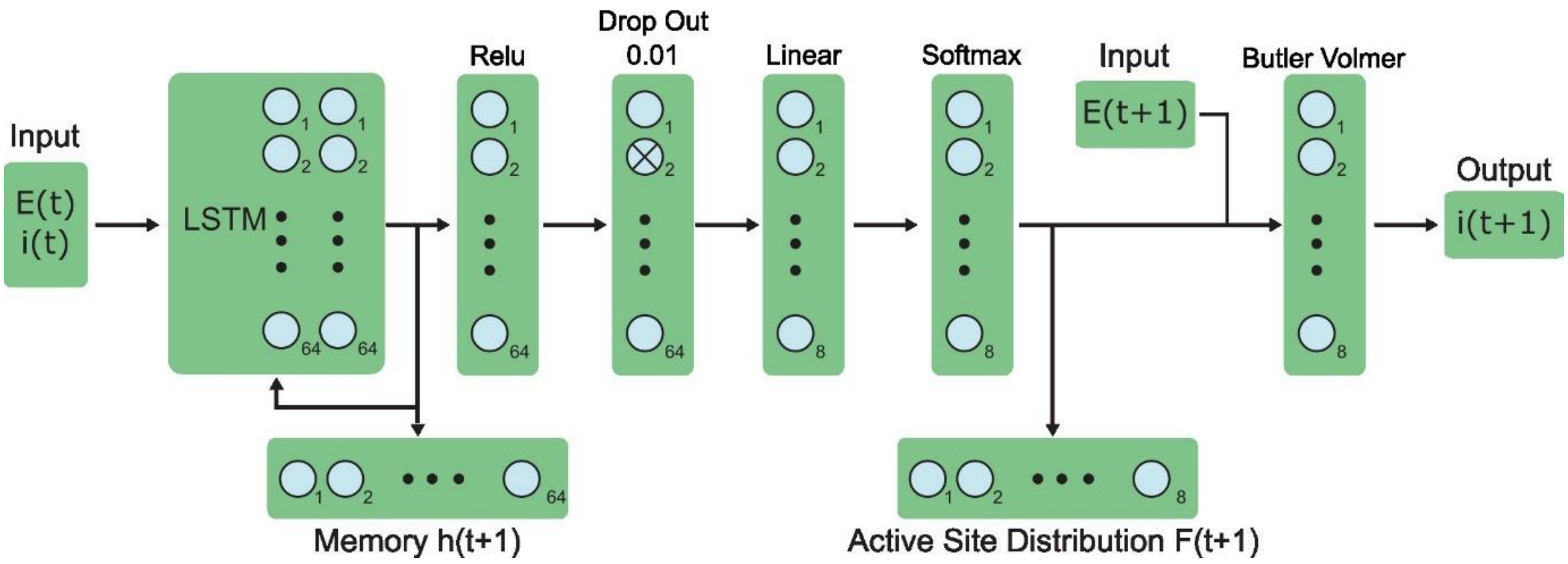

Figure S5. *The structure of the physics inspired AI model with the custom Butler-Volmer layer implemented. The output layer is just a summing layer.*

This custom Butler-Volmer layer was embedded into a recurrent-neural-network architecture to create the physics-inspired AI model illustrated in Figure S4. The number of active sites was arbitrarily set to eight, which was sufficient to produce high performing AI models. At each timestep, the pair *E(t)* and *i(t)* was passed through an LSTM layer to produce a latent state *h(t+1)* describing the evolution of the active-site distribution. This representation was then mapped through a transformer layer into the eight-element vector corresponding to the active-site fractions. Together with the next applied potential *E(t+1),* this vector served as the input to the Butler-Volmer layer, which predicted the next-step current *i(t+1)*.

Training of this physics inspired neural network proceeded in several stages. The $\kappa_{1\text{-}4}$ parameters were initially set randomly but close to zero, ensuring that the corresponding Butler-Volmer equations showed non-zero gradients across the 0-1.2 V domain. The entire set of equations was visualized and inspected, and the network was reinitialized whenever an equation exhibited a flat or non-physical response. After initialization, the model was pretrained for several thousand steps using a constant batch (batch size 4, sequence length 600, burn-in length 550) to shape the Butler-Volmer curves into physically meaningful forms. The equations were again inspected for spurious behavior once pretraining was complete.

Following this pretraining stage, long-range training was performed using the same teacher-forcing strategy described in Section III.1. The sequence length was gradually

increase from 800 to 5000, while the batch size was reduced from 64 to 4 in inverse proportion to maintain computational efficiency. The teacher-forcing ratio was decreased from 1 to 0 over the course of training. Similar to the training of the first generation of AI models, this training was also subject to manual intervention resulting in a non-documented training trajectory.

In the end, two physics inspired AI models were created. The first physics inspired model, AI Model 2.1, was trained on Experiments 1-9 and used the potential as applied during training. However, it displayed validation errors above the acceptable threshold (MSE < 2.5 (mA cm^{-2})^2). Because of this, the dataset was corrected for varying ohmic drops using Eq. 19, where R_{sol} is the solution resistance determined by EIS and the model was retrained starting from the final checkpoint of AI Model 2.1. This retraining produced AI Model 2.2, which exhibited substantially improved accuracy, achieving an MSE = 1.3 (mA cm^{-2})^2) on the test set. The fitting coefficients of AI Model 2.2 are listed in Table S1. Cross-validation scores for both models are provided in Section V of the Supporting Information.

$$E_{corr} = E(t) - i(t)R_{sol} \tag{S19}$$

Table S1. *Parameters of the activity function of the individual active sites as identified by AI model 2.2. In comparison, the Glycerol oxidation CV exhibited a Tafel Slope of 147 mV dec^{-1}. The sites that were preserved during ablation are highlighted in grey.*

Site	j_0	Tafel Slope	E_{eq}	E_{gate}
A	$10^{-7.30}$ mA cm^{-2}	506 mV dec^{-1}	0.577 V	1.342 V
B	$10^{-7.20}$ mA cm^{-2}	331 mV dec^{-1}	0.730 V	1.130 V
C	$10^{-7.54}$ mA cm^{-2}	77 mV dec^{-1}	0.361 V	0.937 V
D	$10^{0.71}$ mA cm^{-2}	1000 mV dec^{-1}	0.300 V	0.834 V
E	$10^{-1.26}$ mA cm^{-2}	157 mV dec^{-1}	0.332 V	0.803 V
F	$10^{-6.17}$ mA cm^{-2}	56 mV dec^{-1}	0.508 V	0.973 V
G	$10^{-0.56}$ mA cm^{-2}	189 mV dec^{-1}	0.359 V	0.914 V
H	$10^{0.71}$ mA cm^{-2}	2000 mV dec^{-1}	0.300 V	1.823 V

III.3 AI Model V2 Ablated – Physics Inspired

The Butler-Volmer Layer of AI controller V2 takes the number of active sites as a hyperparameter. In practice, this hyperparameter governs the bias–variance trade-off. If the model uses only a few active sites, it becomes overly rigid and unable to capture fine dynamics. Conversely, if the number of active sites is set too large, the model may fit all the data but lose interpretability and risk overfitting. To prevent overfitting, the model may employ regularization through dropout, early stopping, as well as an inherently noisy training set.

According to the analysis in Figure 12, the majority of weights are attributed to Sites D, E, H as well as an inactive site. This suggests that the minimum number of active sites to model this system is four. To examine this, we performed an ablation analysis in which a binary mask was introduced to selectively set the activities of sites A, B, C, F, and G to zero, effectively consolidating them into a single inactive site. Subsequently, the ablated model was fine-tuned over 3600 training epochs, with checkpoints stored every 100 epochs. The optimal checkpoint achieved a mean absolute error (MAE) of 0.641 mA cm^{-2} on the entire validation set, representing a reduction of 0.047 mA cm^{-2} compared to the MAE of 0.689 mA cm^{-2} prior to ablation. Despite the slight improvement in the MAE score, the parity plot shows a distinct feature indicating that the ablated model is unable to capture certain dynamics with the same level of precision (Figure S6). Analysis of the active-sites contributions (Figure S7) further reveals that the ablated model exhibits slightly larger deviations in current prediction following the 1.0 V reactivation pulse. However, the conclusions on the dynamics of sites D and E remain unchanged.

Table S2. *Parameters of the activity function of the individual active sites as identified by the ablated AI model 2.2.*

Site	j_0	Tafel Slope	E_{eq}	E_{gate}
**A**	N/A	N/A	N/A	N/A
**B**	N/A	N/A	N/A	N/A
**C**	N/A	N/A	N/A	N/A
**D**	$10^{0.71}$ mA cm^{-2}	993 mV dec^{-1}	0.300 V	0.834 V
**E**	$10^{-1.23}$ mA cm^{-2}	154 mV dec^{-1}	0.332 V	0.819 V
**F**	N/A	N/A	N/A	N/A
**G**	N/A	N/A	N/A	N/A
**H**	$10^{0.71}$ mA cm^{-2}	2049 mV dec^{-1}	0.300 V	1.823 V

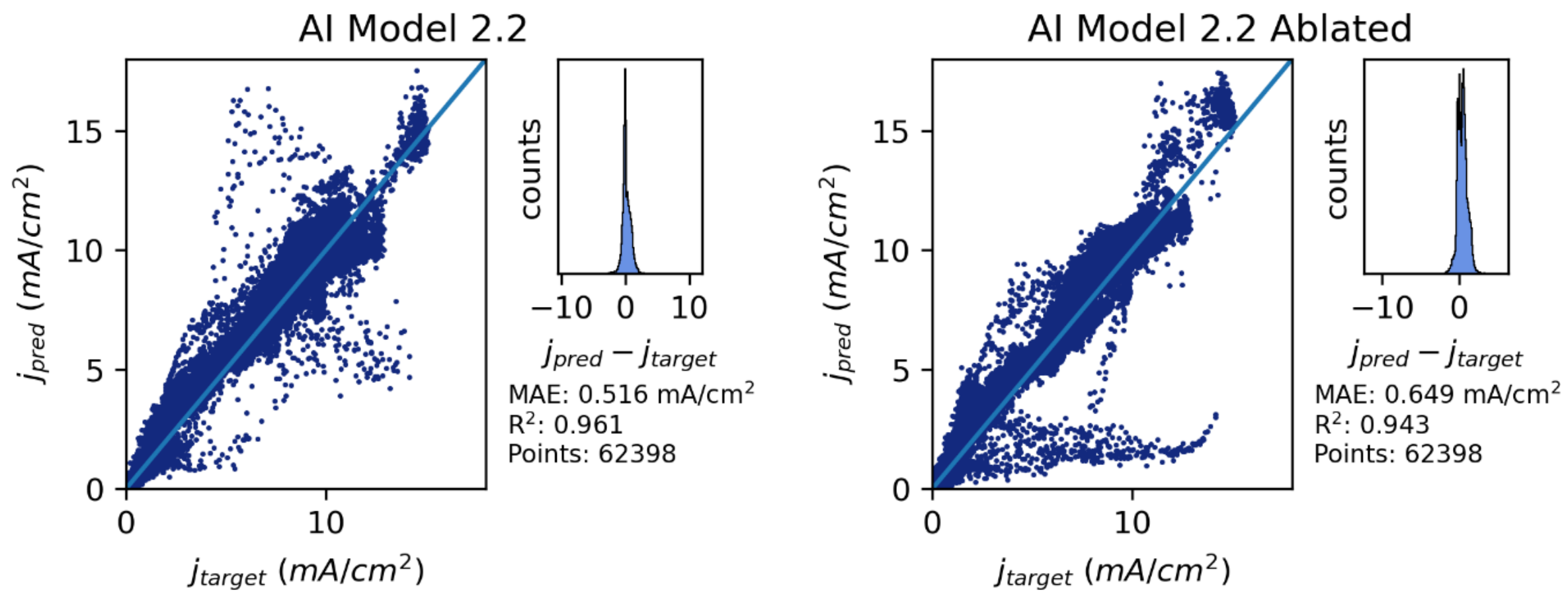

Figure S6. *Parity plots of Experiment 12.13 as validated by the AI model 2.2 and the ablated version 2.2. The ablation results in a set of values that are not correctly predicted.*

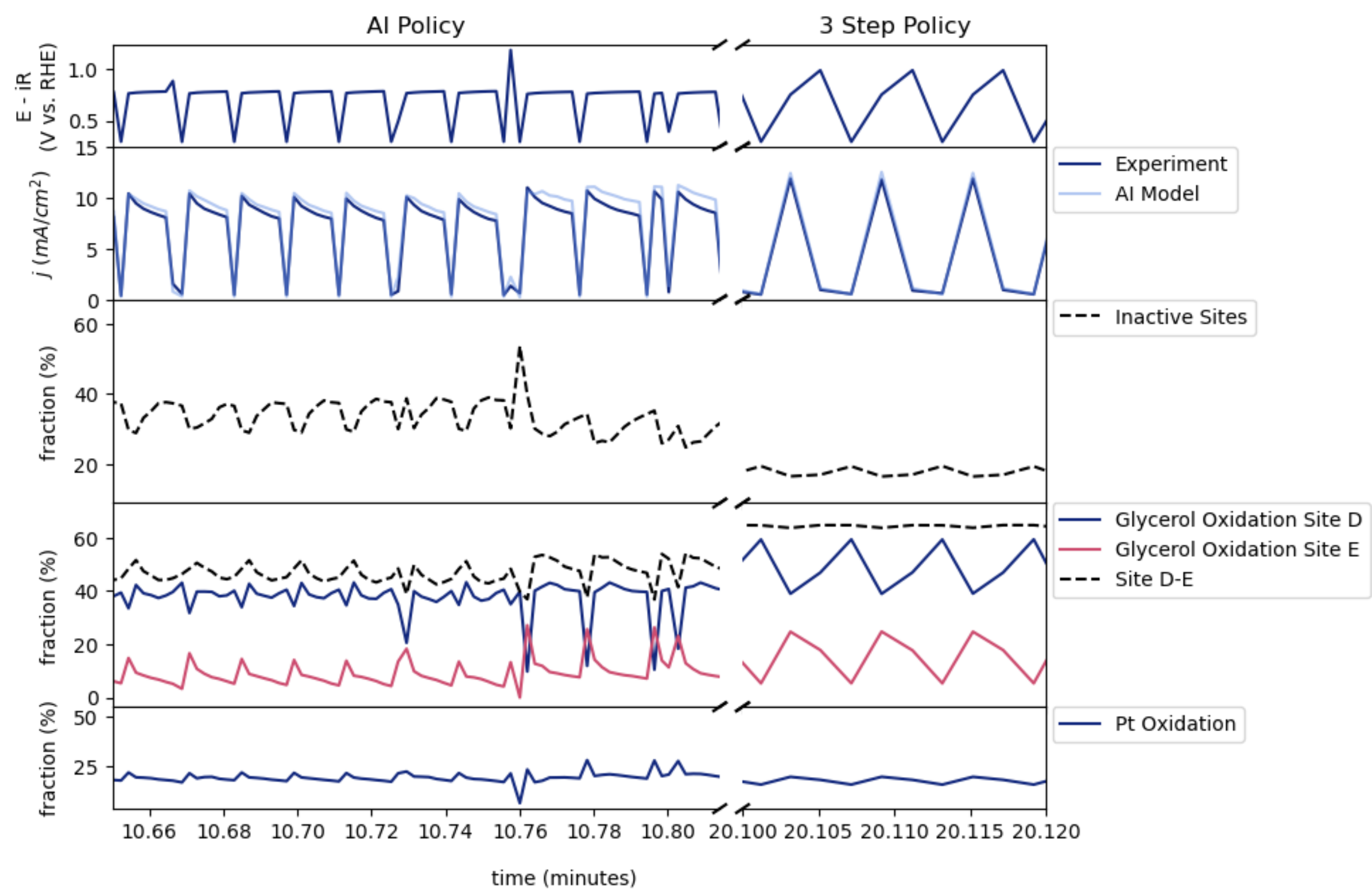

Figure S7. *The same figure as Figure 12, but the active sites have been analyzed using the ablated AI model 2.2.*

IV. Supporting Figures Experiment 12

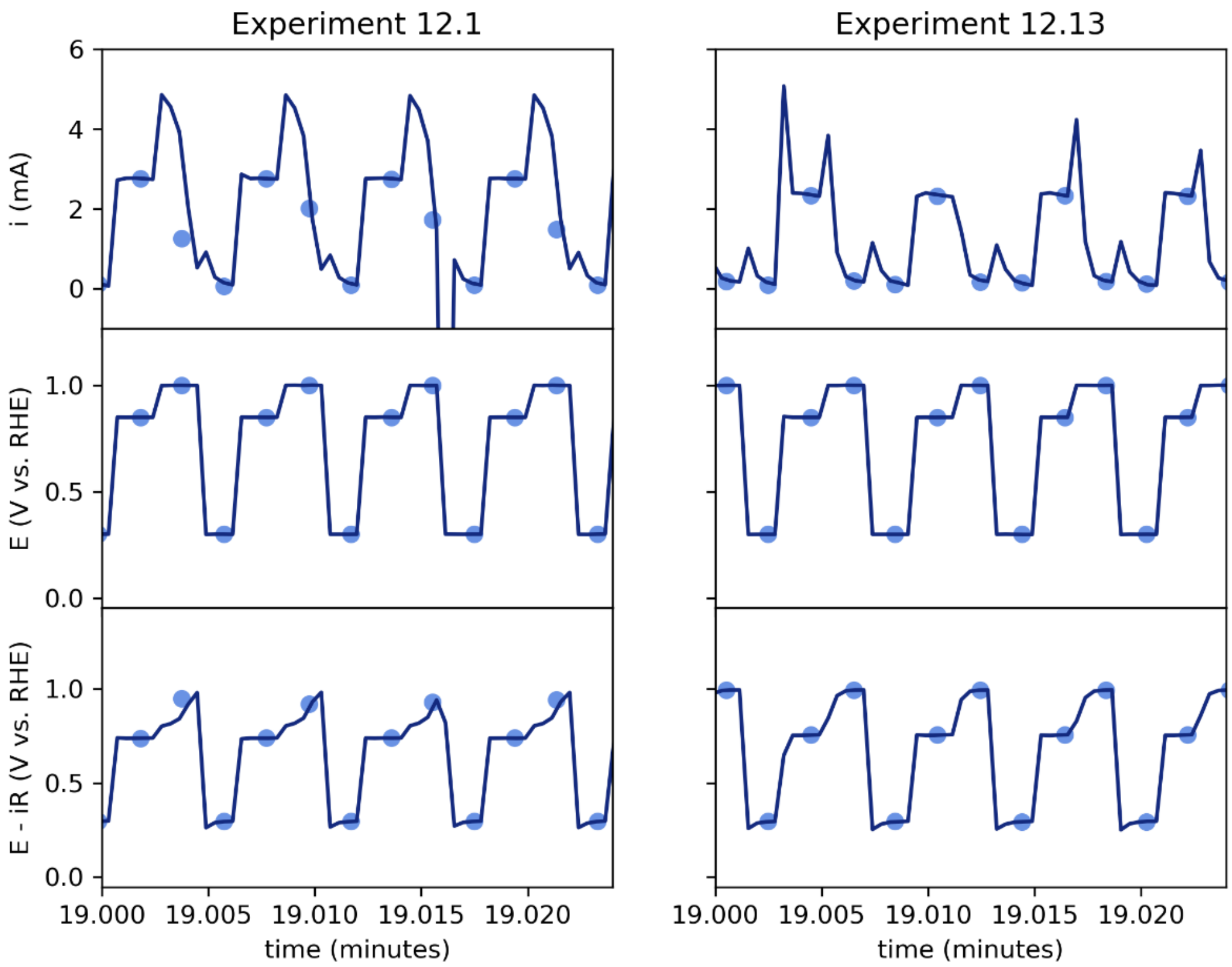

Figure S8. *Raw experimental data in experiment 12 under the three-step policy. Markers correspond to data points observed by the AI controller, whereas the line corresponds to the true transients. The experiments were recorded in 0.1 M KOH and 0.1 M glycerol (Ar saturated) at 200 rpm. The solution resistance was obtained from the EIS and corrected according to Eq. S19.*

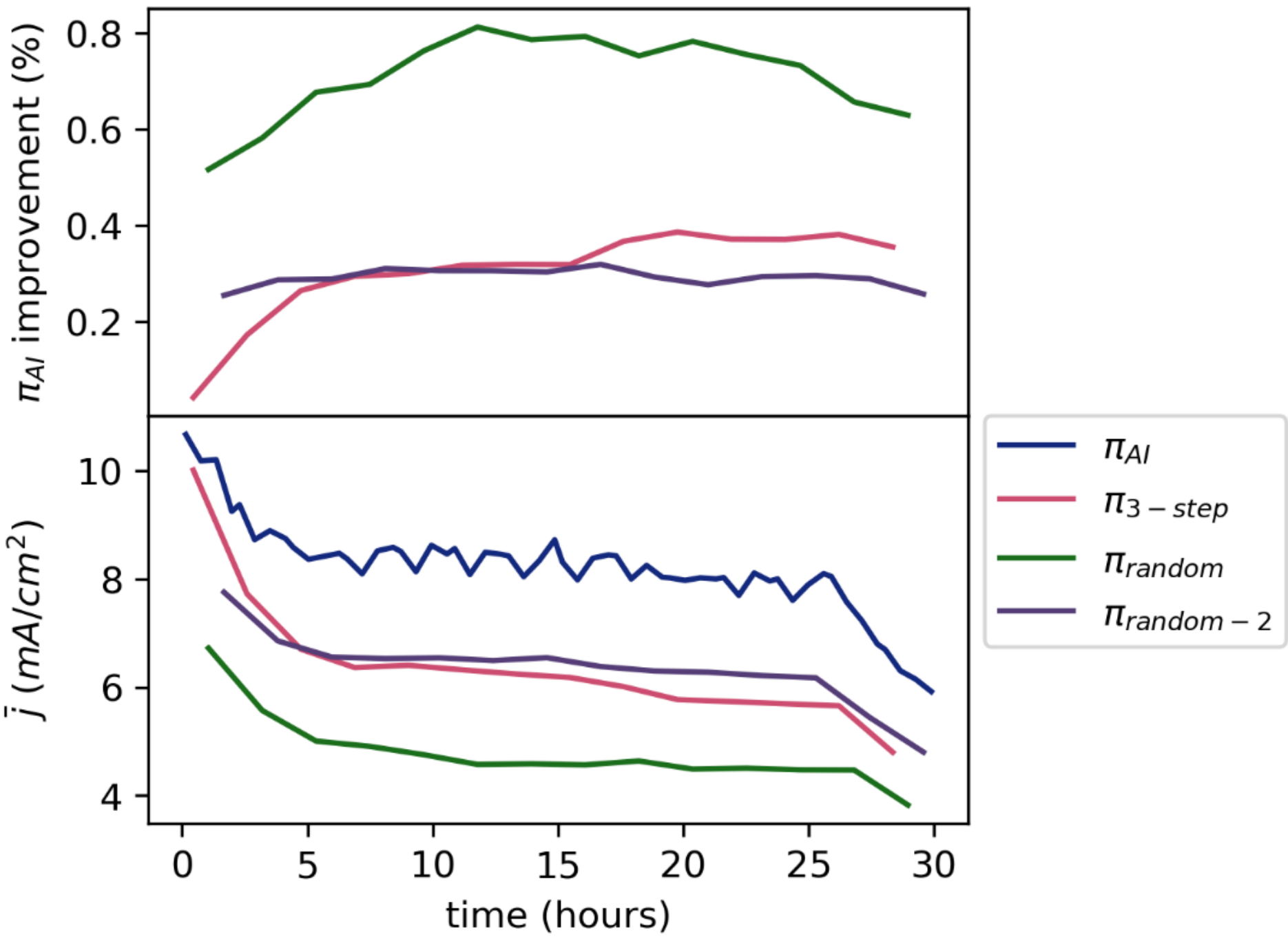

Figure S9. *The summary of AI Controller 3 improvement over the three-step policy, a uniform random policy π_{random}, and a center-biased random policy $\pi_{random\text{-}2}$. The data is the combined data set collected during experiment 12, joined together under the assumption that no time delays occurred during the restarts. The average currents are calculated using bins of 15 minutes. The relative improvement is calculated using the AI performances of the 2 adjacent time bins to the policy bin under evaluation. On average, the AI outperforms the 3-step policy by 30%, π_{random} by 71% and $\pi_{random\text{-}2}$ by 29%.*

V Experimental Data Sets

In this project a total of 12 experiments were performed. This section shows a summary of the data per experiment.

Experiment 1

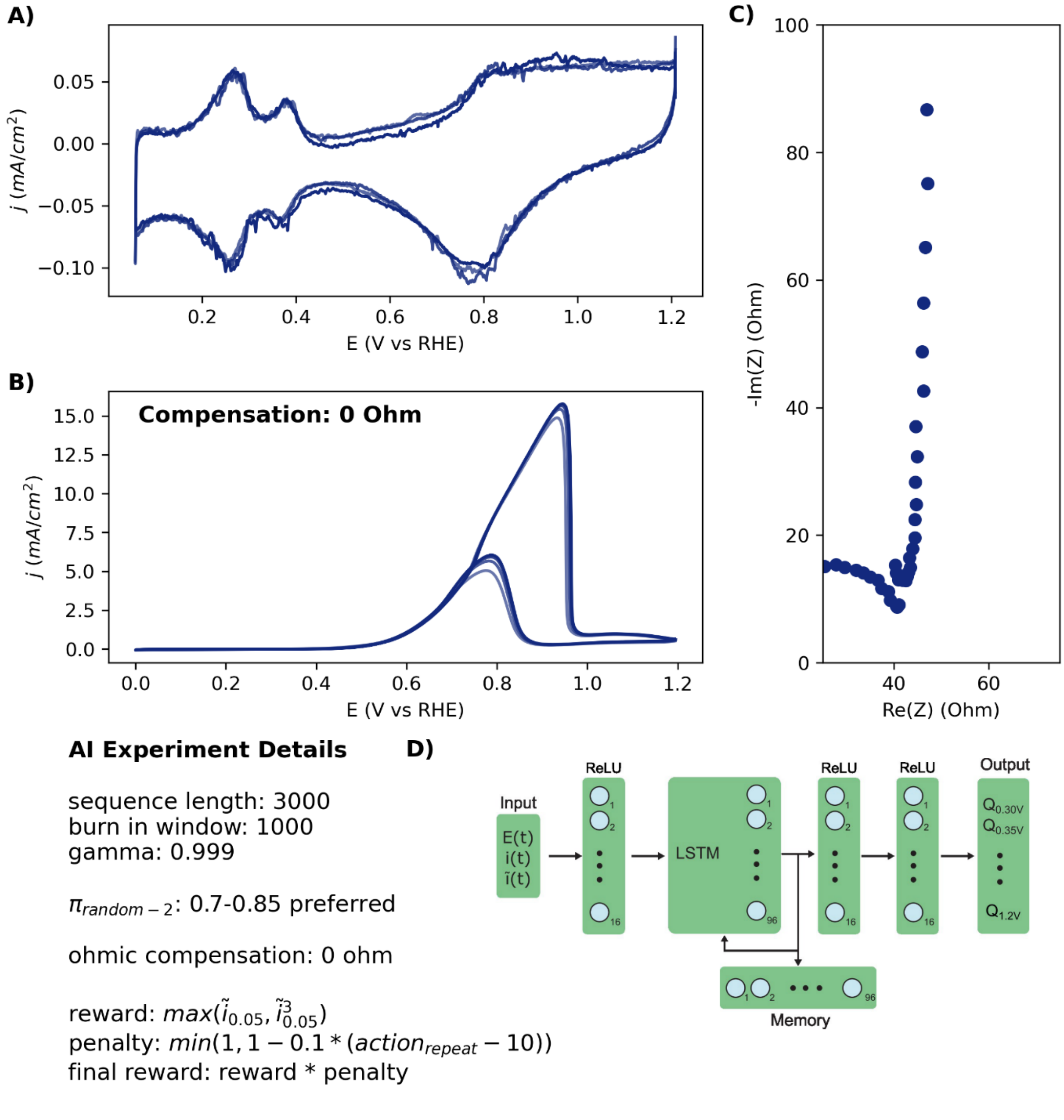

Figure S10. *Summary of Experiment 1.* ***A)*** *Pt characterization CV at 0 rpm, 50 mV s*$^{-1}$ *in 0.1 M KOH Ar*$_{sat}$*.* ***B)*** *Pt characterization CV at 200 rpm, 50 mV s*$^{-1}$ *in 0.1 M KOH, 0.1 M glycerol Ar*$_{sat}$*.* ***C)*** *Nyquist plot of Pt at OCV in 0.1 M KOH, 0.1 M glycerol.* ***D)*** *AI-controller architecture used in this experiment. The output layer is a linear unit.*

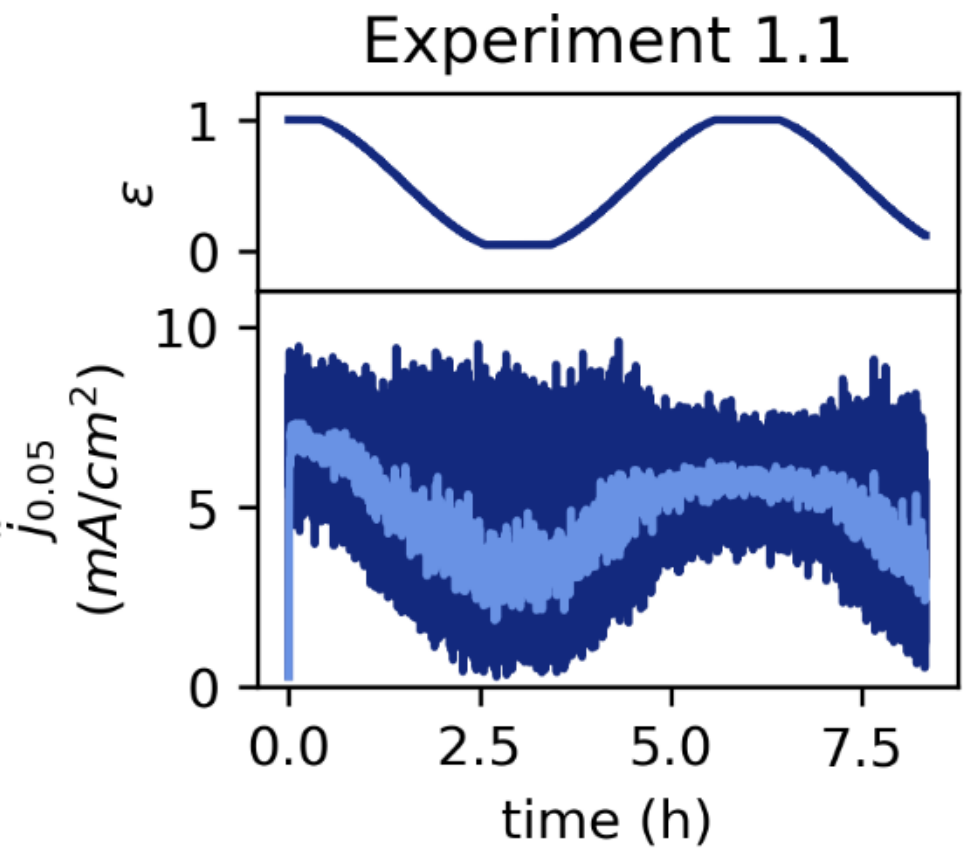

Figure S11. *Summary of data acquired under AI control of Experiment 1.*

Experiment 2

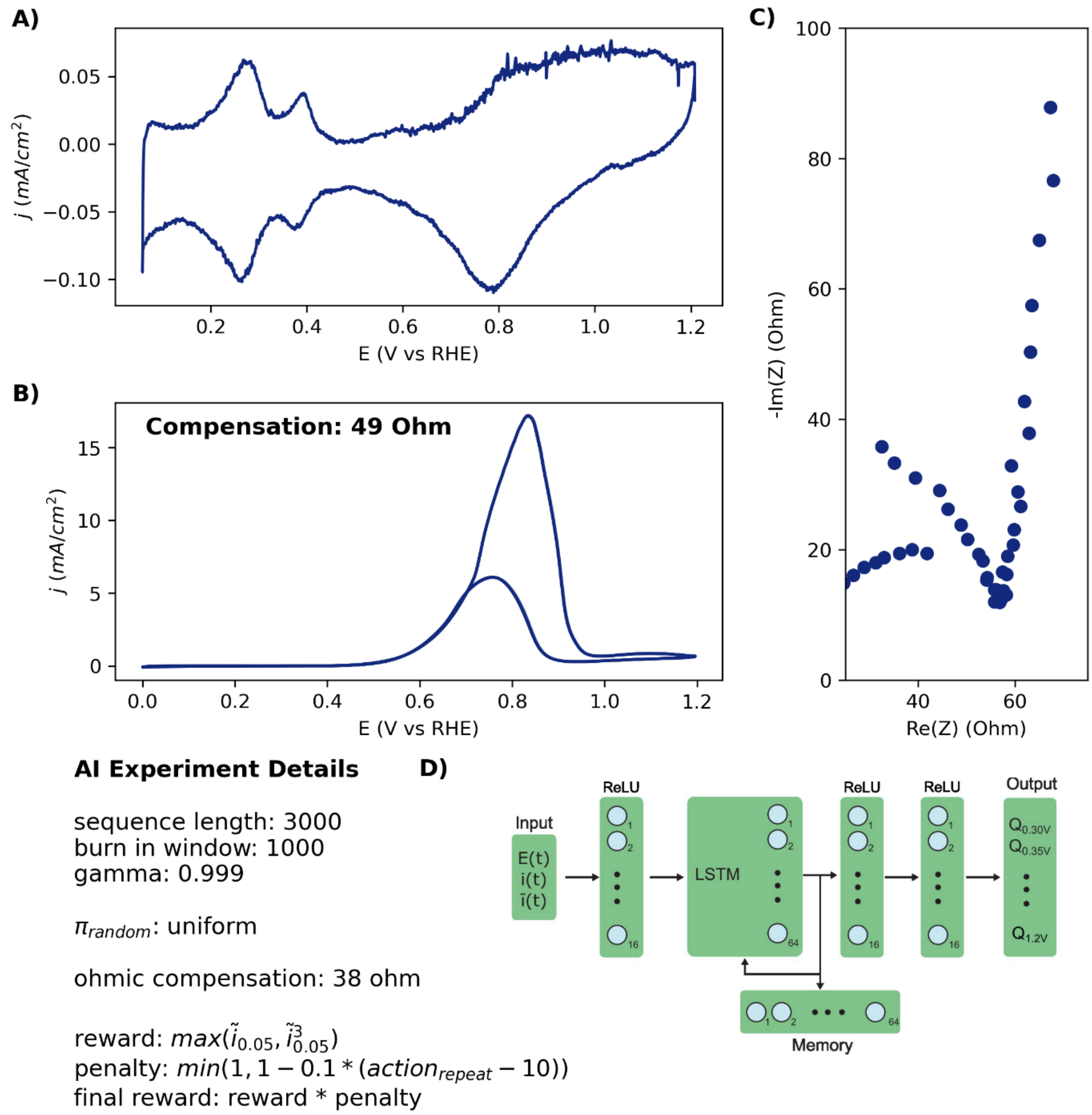

***Figure S12**. Summary of Experiment 2. **A)** Pt characterization CV at 0 rpm, 50 mV s^{-1} in 0.1 M KOH Ar_{sat}. **B)** Pt characterization CV at 200 rpm, 50 mV s^{-1} in 0.1 M KOH, 0.1 M glycerol Ar_{sat}. **C)** Nyquist plot of Pt at OCV in 0.1 M KOH, 0.1 M glycerol. **D)** AI-controller architecture used in this experiment. The output layer is a linear unit.*

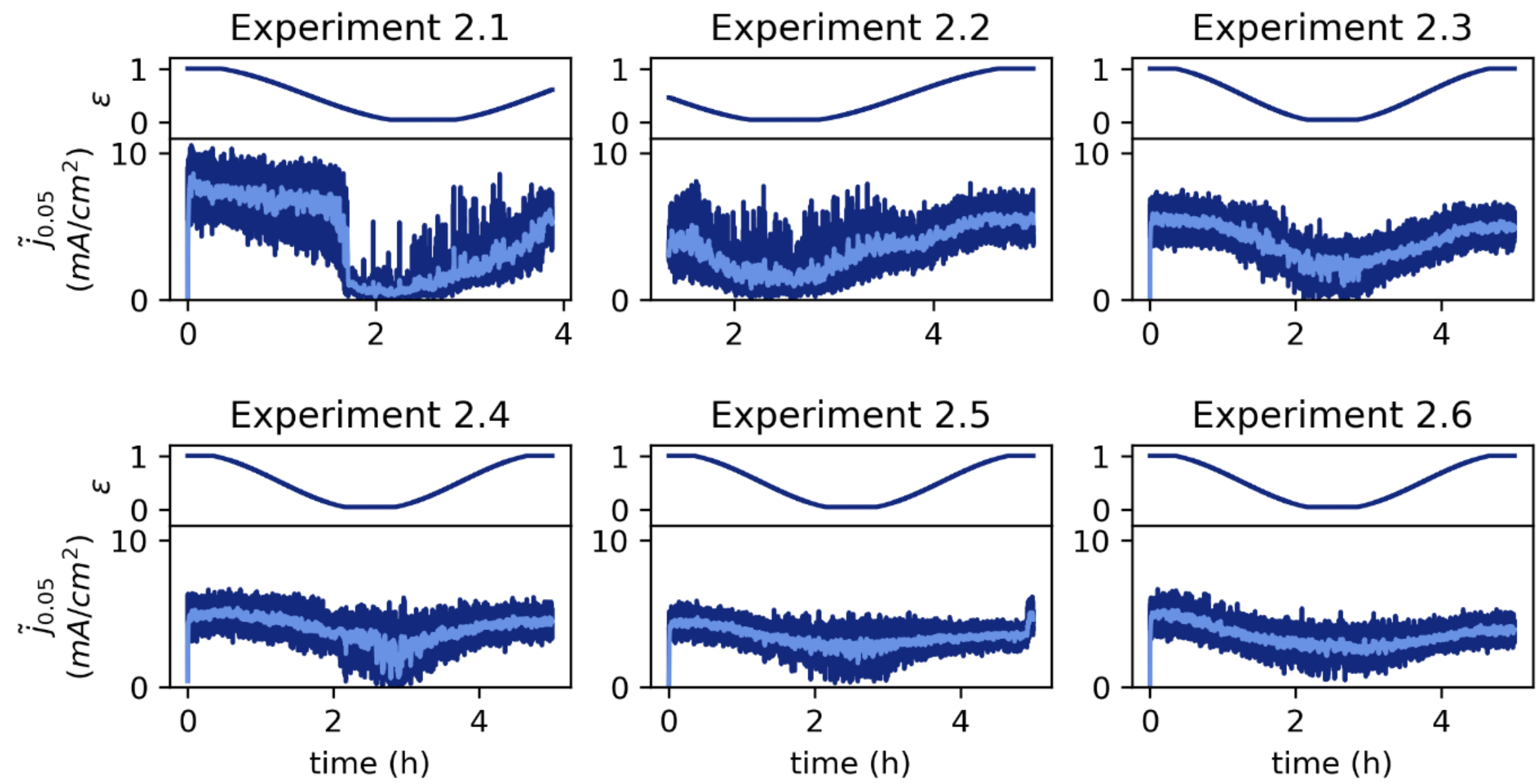

Figure S13. *Summary of data acquired under AI control of Experiment 2.*

Experiment 3

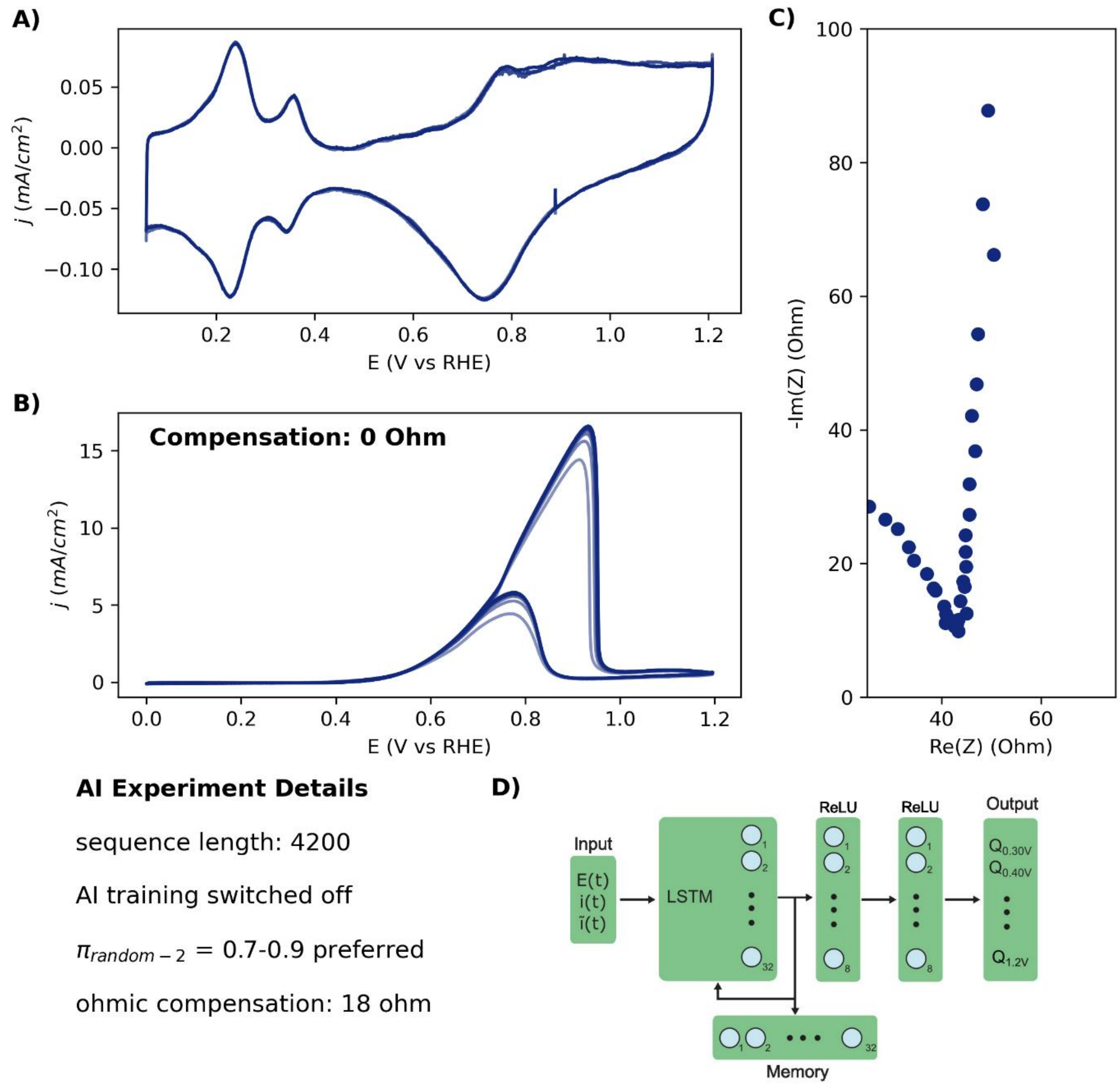

Figure S14. *Summary of Experiment 3.* ***A)*** *Pt characterization CV at 0 rpm, 50 mV s^{-1} in 0.1 M KOH Ar_{sat}.* ***B)*** *Pt characterization CV at 200 rpm, 50 mV s^{-1} in 0.1 M KOH, 0.1 M glycerol Ar_{sat}*. ***C)*** *Nyquist plot of Pt at OCV in 0.1 M KOH, 0.1 M glycerol.* ***D)*** *AI-controller architecture used in this experiment. The output layer is a linear unit.*

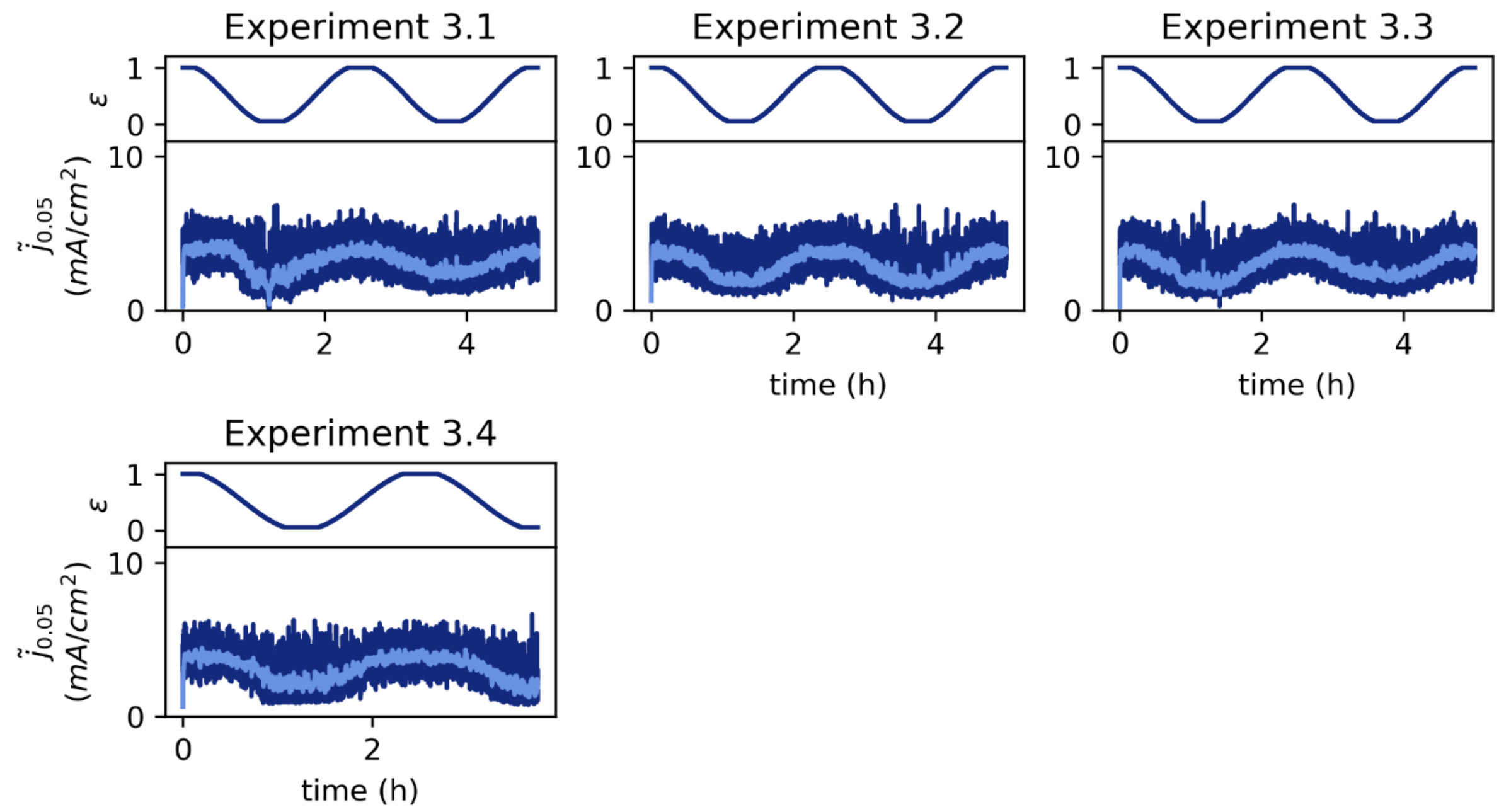

Figure S15. *Summary of data acquired under AI control of Experiment 3.*

Experiment 4

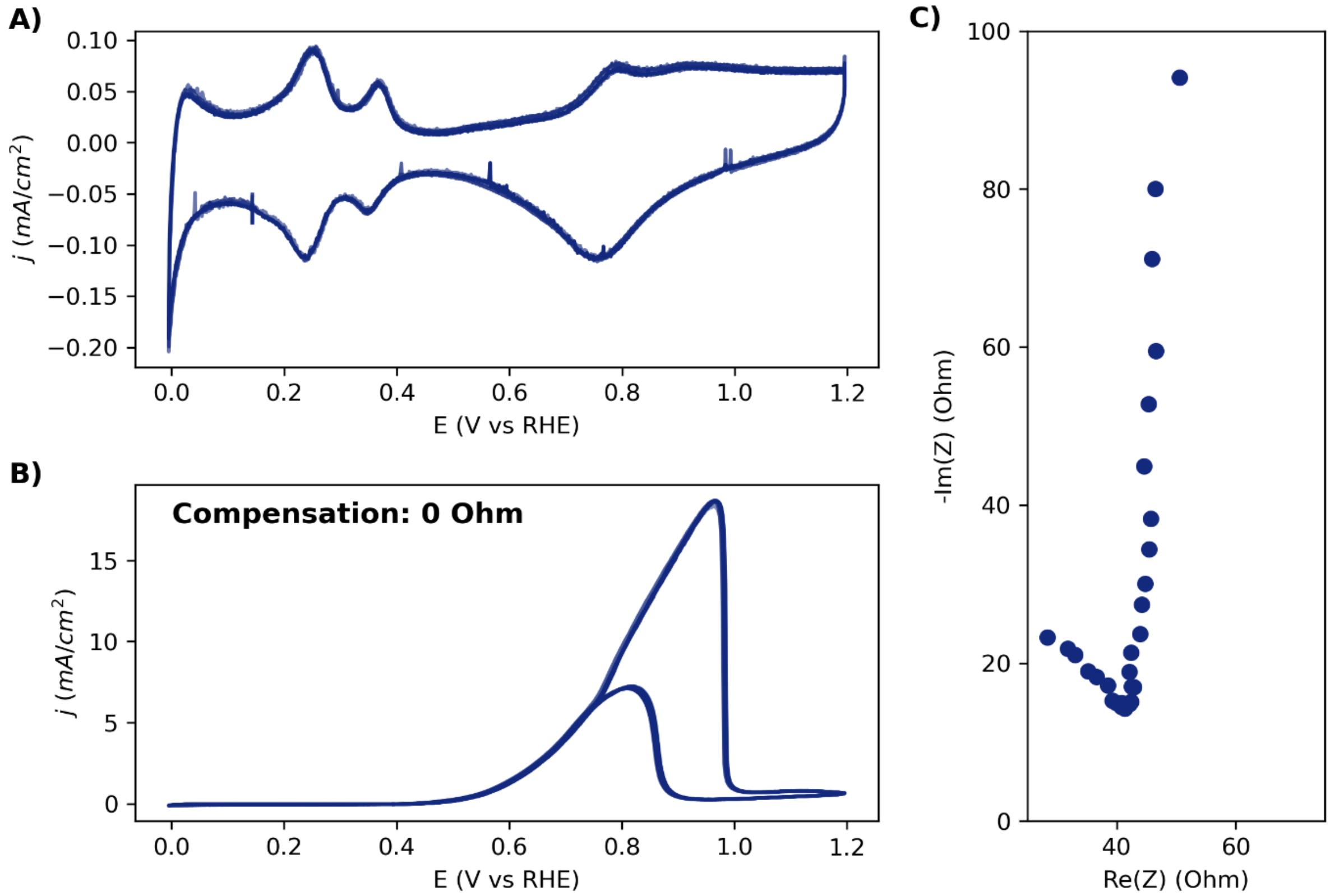

AI Experiment Details

sequence length: 4200
burn in window: 1200
gamma: 0.995

$\pi_{random-2}$: 0.7-0.85 preferred

ohmic compensation: 16 ohm

reward: $max(\tilde{i}_{0.05}, \tilde{i}^3_{0.05})$
penalty: $min(1, 1 - 0.1 * (action_{repeat} - 10))$
final reward: reward * penalty

D)

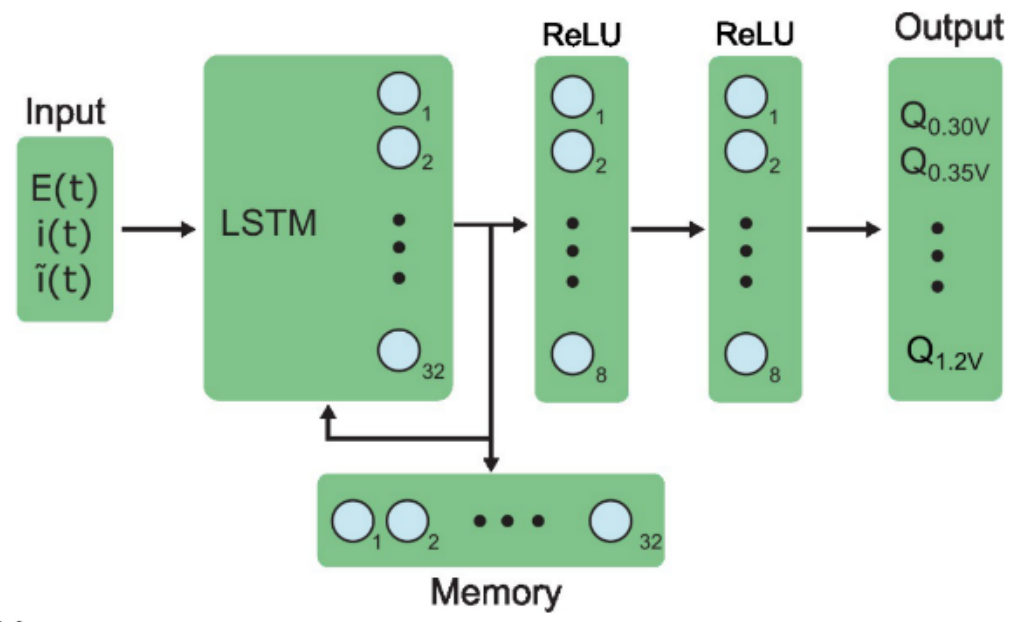

Figure S16. *Summary of Experiment 4.* ***A)*** *Pt characterization CV at 0 rpm, 50 mV s^{-1} in 0.1 M KOH Ar$_{sat}$.* ***B)*** *Pt characterization CV at 200 rpm, 50 mV s^{-1} in 0.1 M KOH, 0.1 M glycerol Ar$_{sat}$.* ***C)*** *Nyquist plot of Pt at OCV in 0.1 M KOH, 0.1 M glycerol.* ***D)*** *AI-controller architecture used in this experiment. The output layer is a linear unit.*

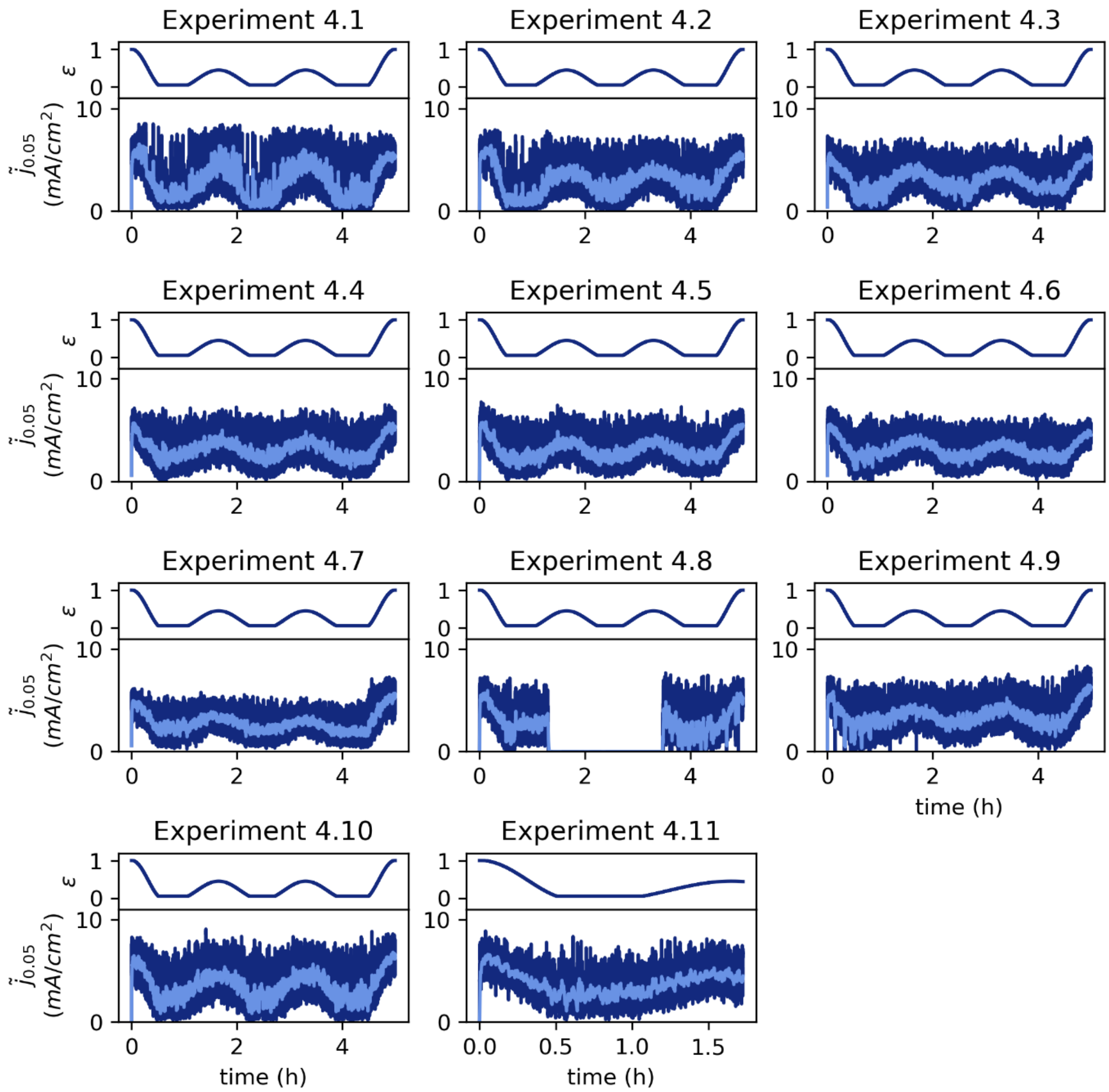

Figure S17. *Summary of data acquired under AI control of Experiment 4. Experiment 4.8 lost contact and therefore was not used in offline training schemes.*

Experiment 5.1

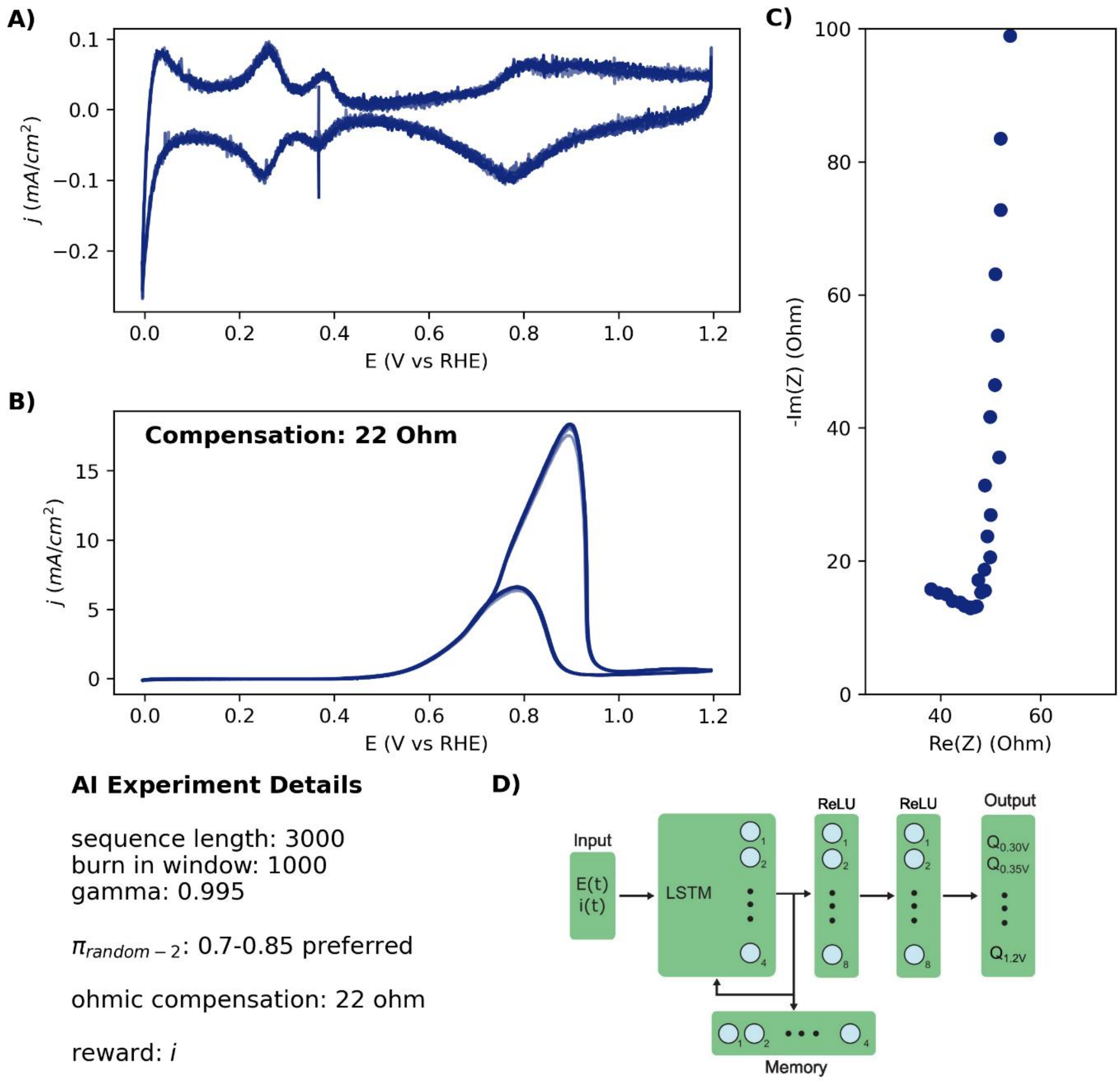

Figure S18. *Summary of Experiment 5.* ***A)*** *Pt characterization CV at 0 rpm, 50 mV s*$^{-1}$ *in 0.1 M KOH Ar*$_{sat}$. ***B)*** *Pt characterization CV at 200 rpm, 50 mV s*$^{-1}$ *in 0.1 M KOH, 0.1 M glycerol Ar*$_{sat}$***.*** ***C)*** *Nyquist plot of Pt at OCV in 0.1 M KOH, 0.1 M glycerol.* ***D)*** *AI-controller architecture used in this experiment. The output layer is a linear unit.*

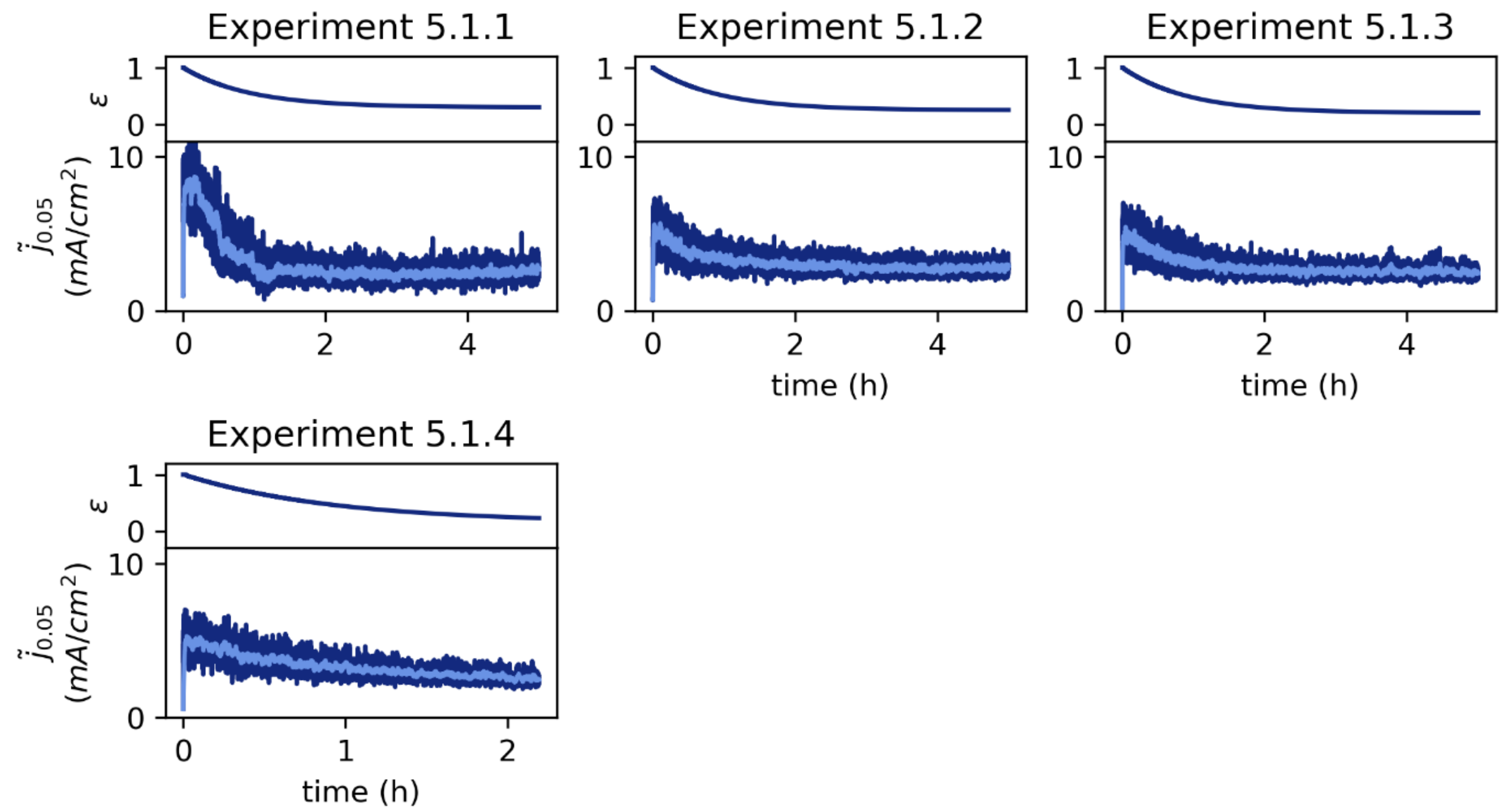

Figure S19. *Summary of data acquired under AI control of Experiment 5.1.*

Experiment 5.2

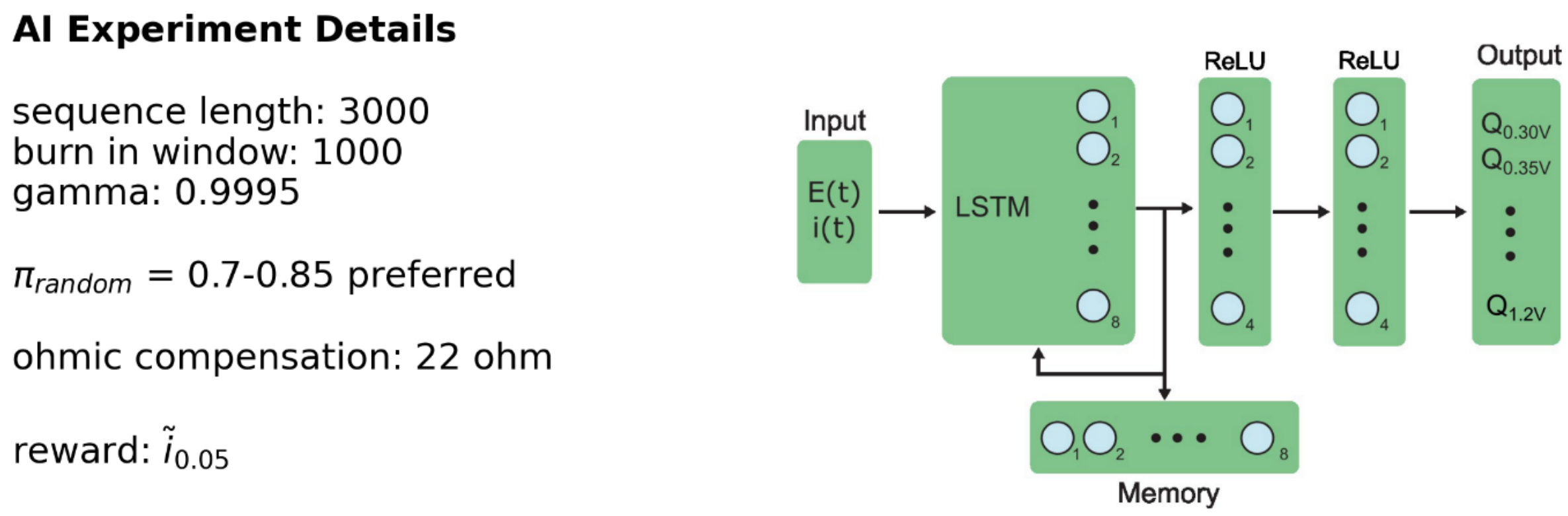

Figure S20. *Changes to the AI controller for Experiment 5.2, which was performed directly after 5.1. The output layer is a linear unit.*

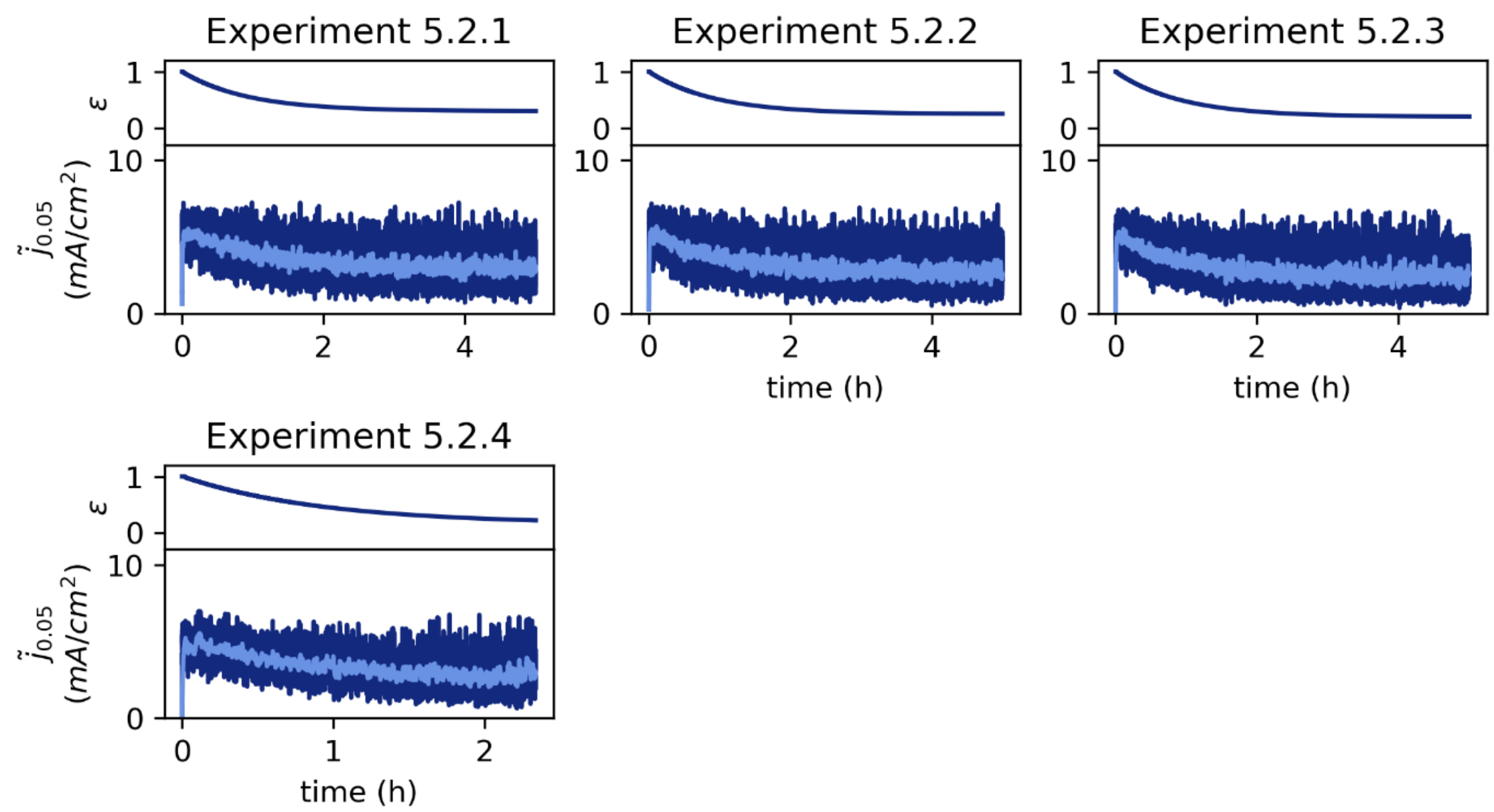

Figure S21. *Summary of data acquired under AI control of Experiment 5.2.*

Experiment 6

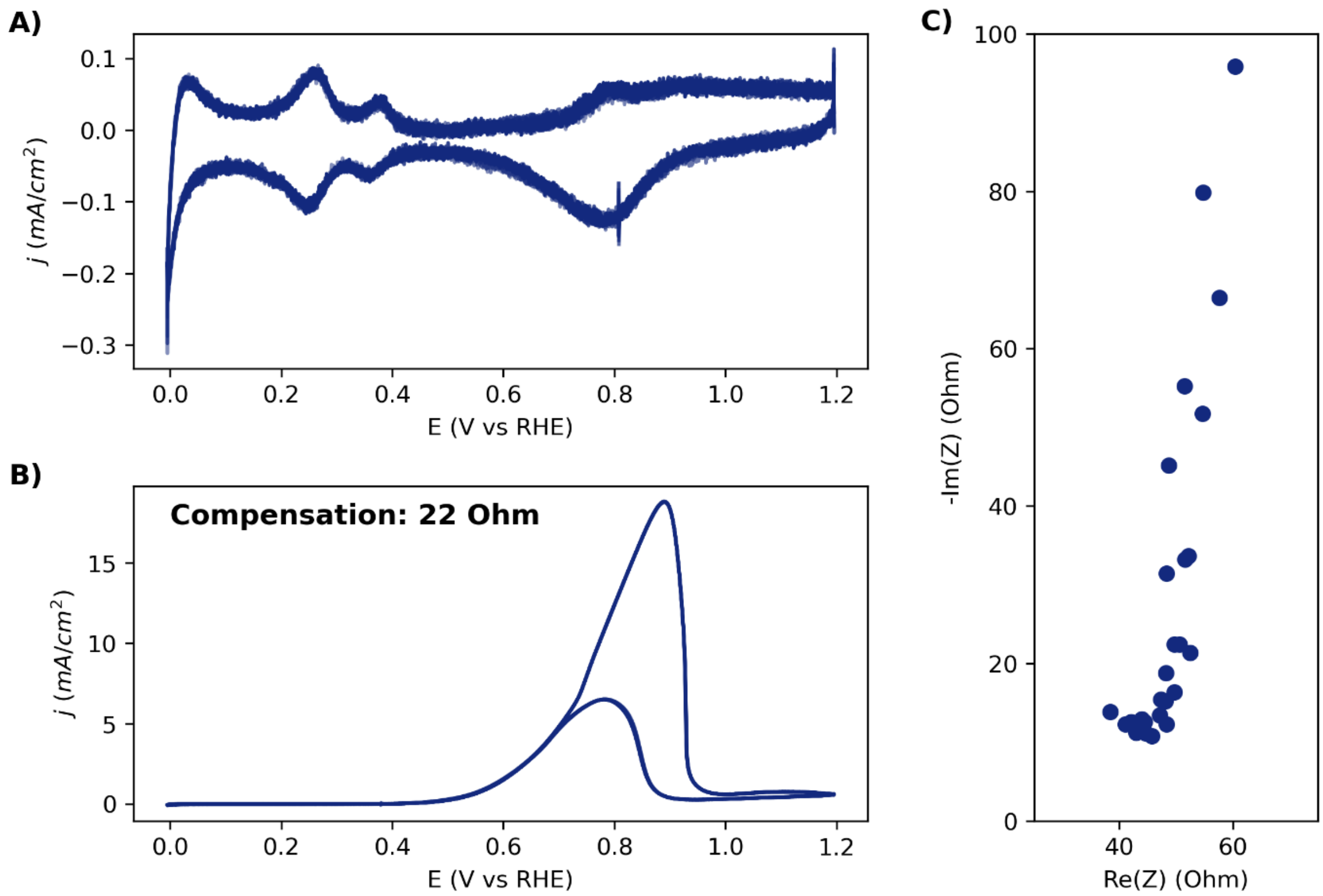

AI Experiment Details

sequence length: 1200
burn in window: 600
gamma: 0.9995

π_{random}: uniform

ohmic compensation: 22 ohm

reward: $\tilde{i}_{0.05} + max(0, (\tilde{i}_{0.05} - 1) * 2))$
penalty: $max(0, min(1, 1 - 0.05 * (action_{repeat} - 10))$
final reward: reward * penalty

D)

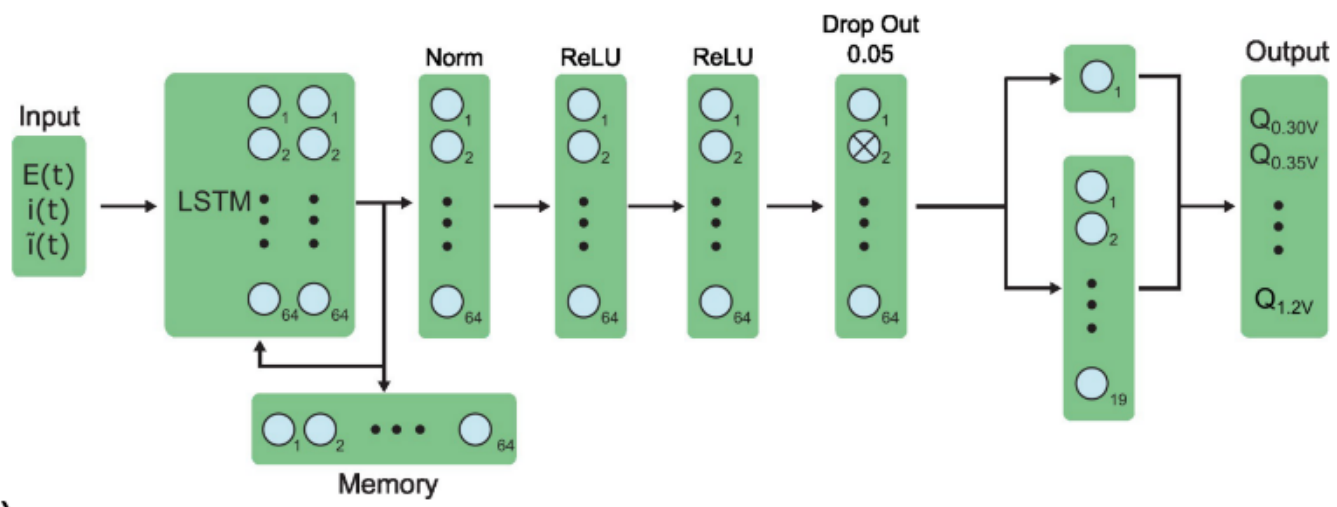

***Figure S22**. Summary of Experiment 6. **A)** Pt characterization CV at 0 rpm, 50 mV s^{-1} in 0.1 M KOH Ar$_{sat}$. **B)** Pt characterization CV at 200 rpm, 50 mV s^{-1} in 0.1 M KOH, 0.1 M glycerol Ar$_{sat}$. **C)** Nyquist plot of Pt at OCV in 0.1 M KOH, 0.1 M glycerol. **D)** AI-controller architecture used in this experiment. The output layer is a linear unit.*

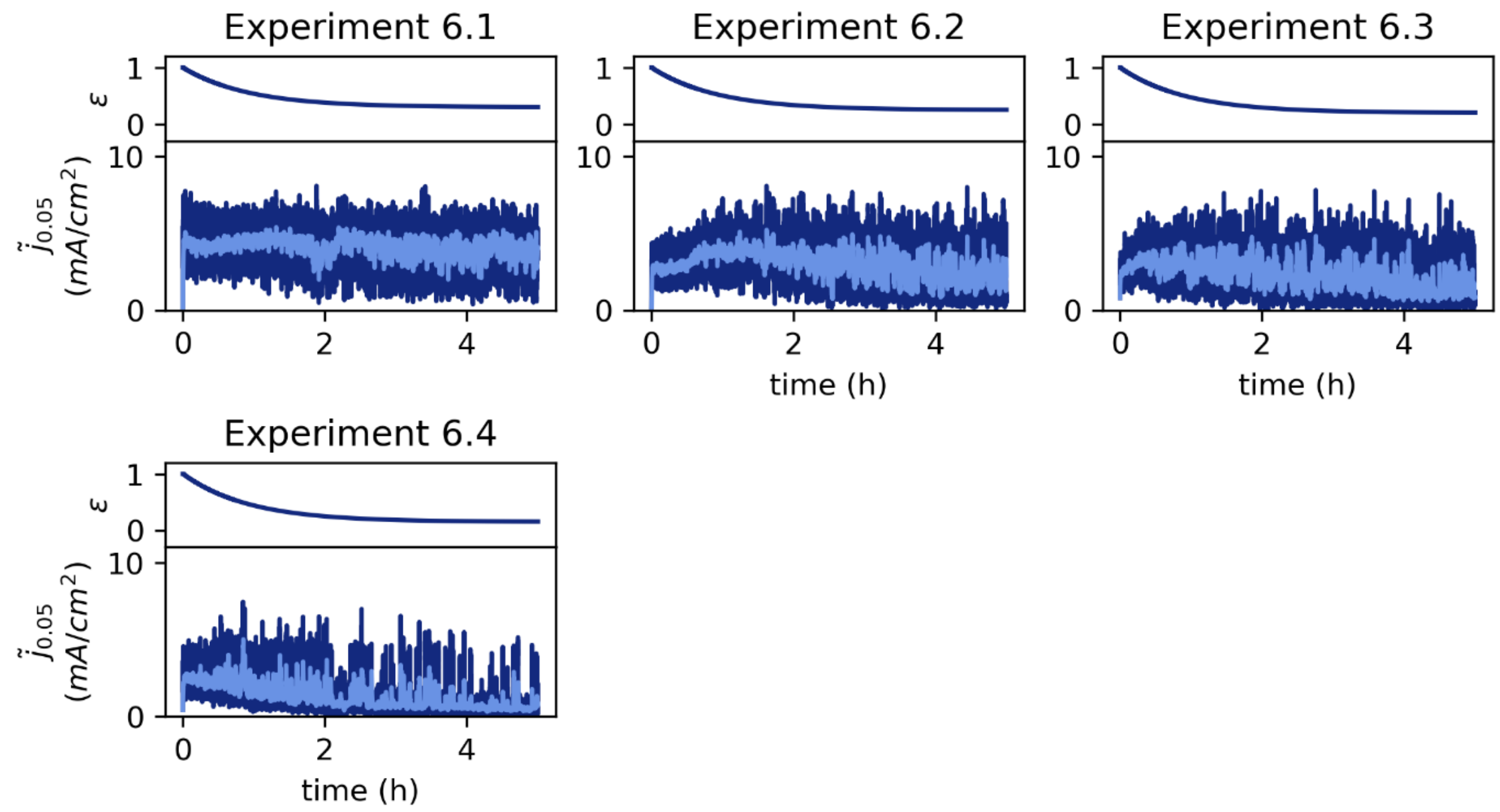

Figure S23. *Summary of data acquired under AI control of Experiment 6.*

Experiment 7

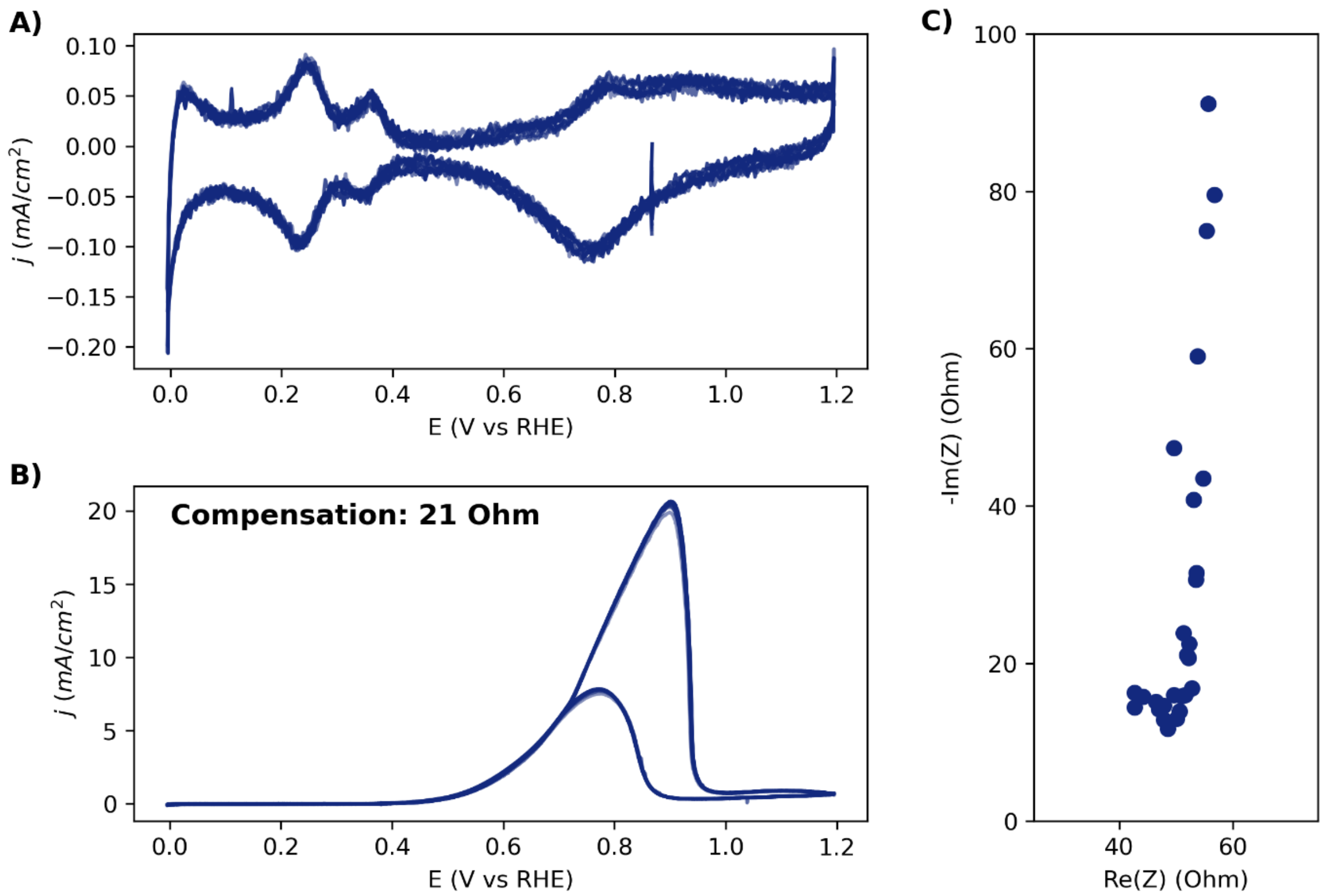

AI Experiment Details

sequence length: 1200
burn in window: 600
gamma: 0.9995

π_{random}: uniform

ohmic compensation: 20 ohm

reward: $\tilde{i}_{0.05} + max(0, (\tilde{i}_{0.05} - 1) * 2))$
penalty: $max(0, min(1, 1 - 0.05 * (action_{repeat} - 10))$
final reward: reward * penalty

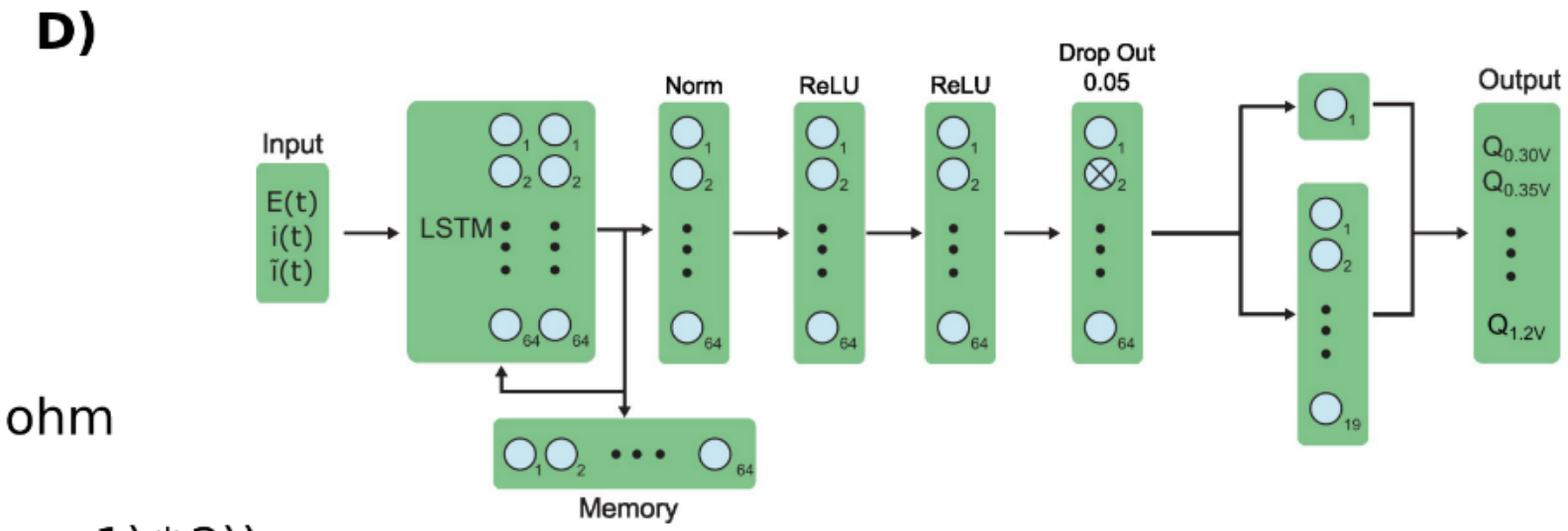

Figure S24. *Summary of Experiment 7.* ***A)*** *Pt characterization CV at 0 rpm, 50 mV s⁻¹ in 0.1 M KOH Ar_{sat}.* ***B)*** *Pt characterization CV at 200 rpm, 50 mV s^{-1} in 0.1 M KOH, 0.1 M glycerol Ar_{sat}.* ***C)*** *Nyquist plot of Pt at OCV in 0.1 M KOH, 0.1 M glycerol.* ***D)*** *AI-controller architecture used in this experiment. The output layer is a linear unit.*

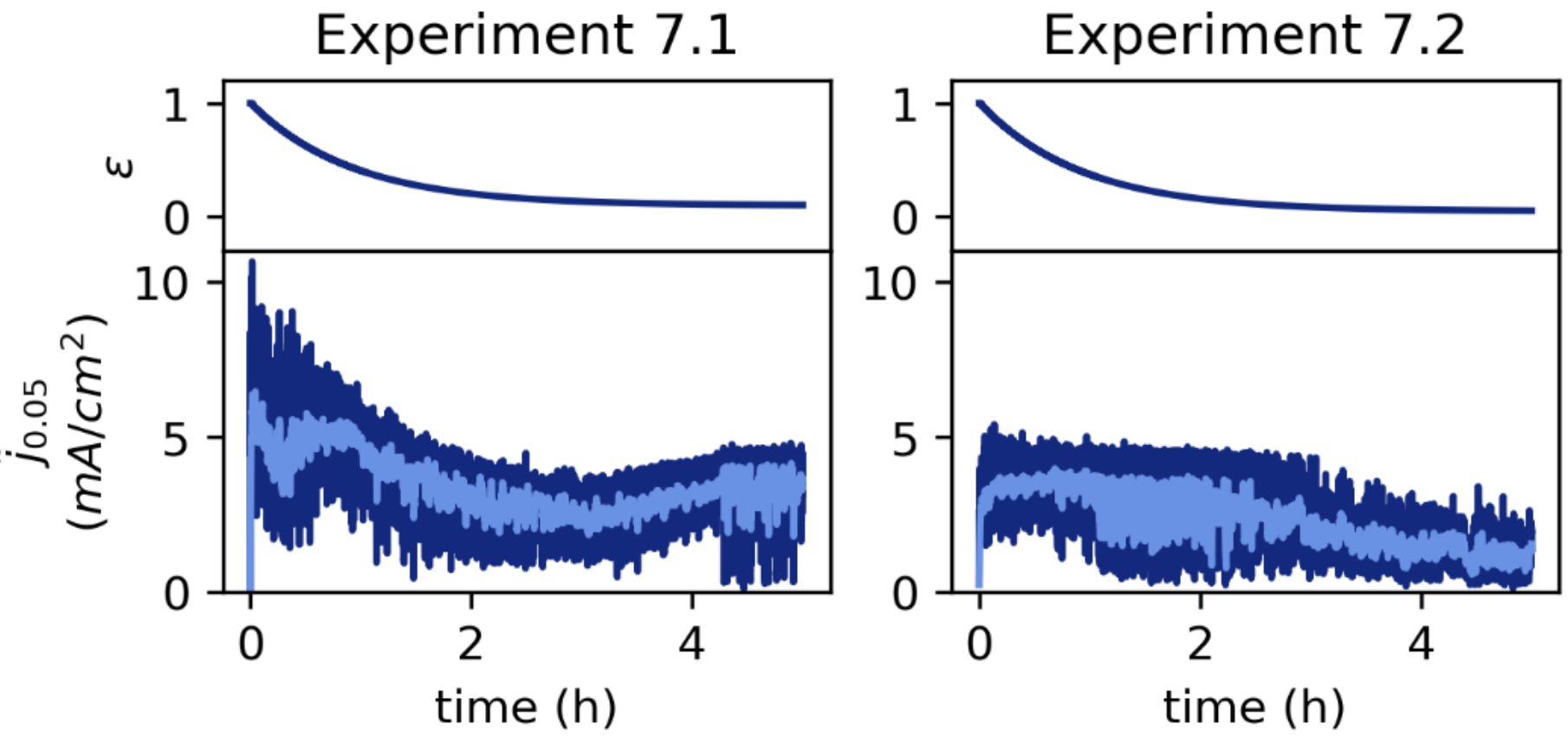

Figure S25. *Summary of data acquired under AI control of Experiment 7*

Experiment 8.1

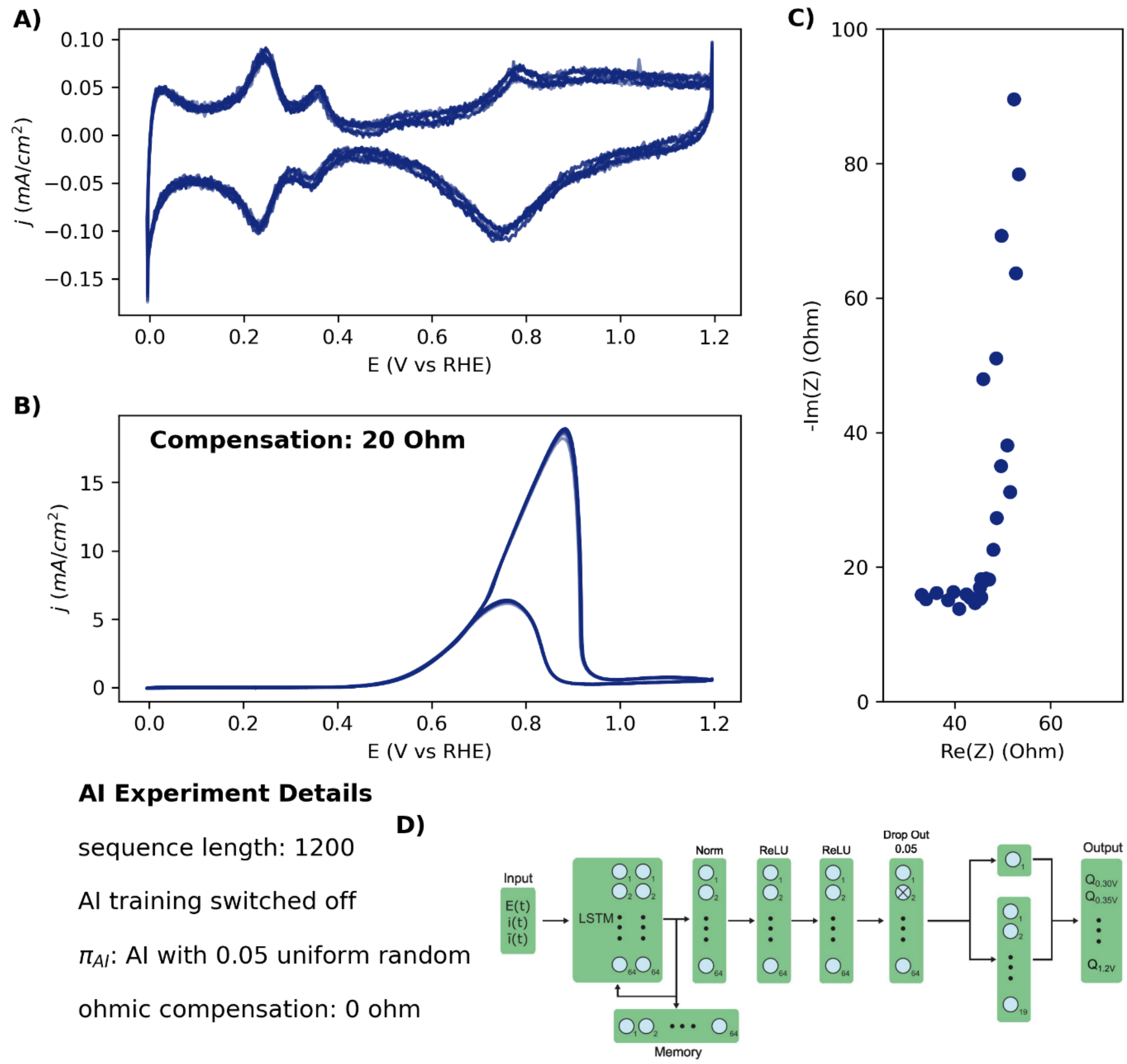

Figure S26. *Summary of Experiment 8.1.* ***A)*** *Pt characterization CV at 0 rpm, 50 mV s⁻¹ in 0.1 M KOH Ar_{sat}.* ***B)*** *Pt characterization CV at 200 rpm, 50 mV s⁻¹ in 0.1 M KOH, 0.1 M glycerol Ar_{sat}.* ***C)*** *Nyquist plot of Pt at OCV in 0.1 M KOH, 0.1 M glycerol.* ***D)*** *AI-controller architecture used in this experiment. The output layer is a linear unit.*

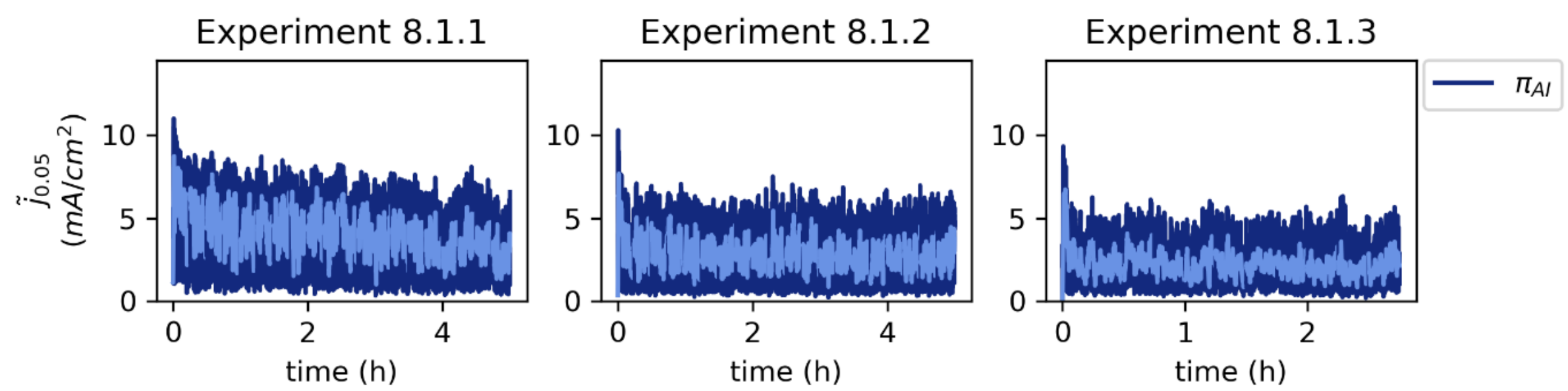

Figure S27. *Summary of data acquired under AI control of Experiment 8.1.*

Experiment 8.2

AI Experiment Details

sequence length: 1200

AI training switched off

π_{AI}: AI with 0.05 uniform random
π_{random}: uniform
$\pi_{random-2}$: 0.7 to 0.85 preferred

ohmic compensation: 0 ohm

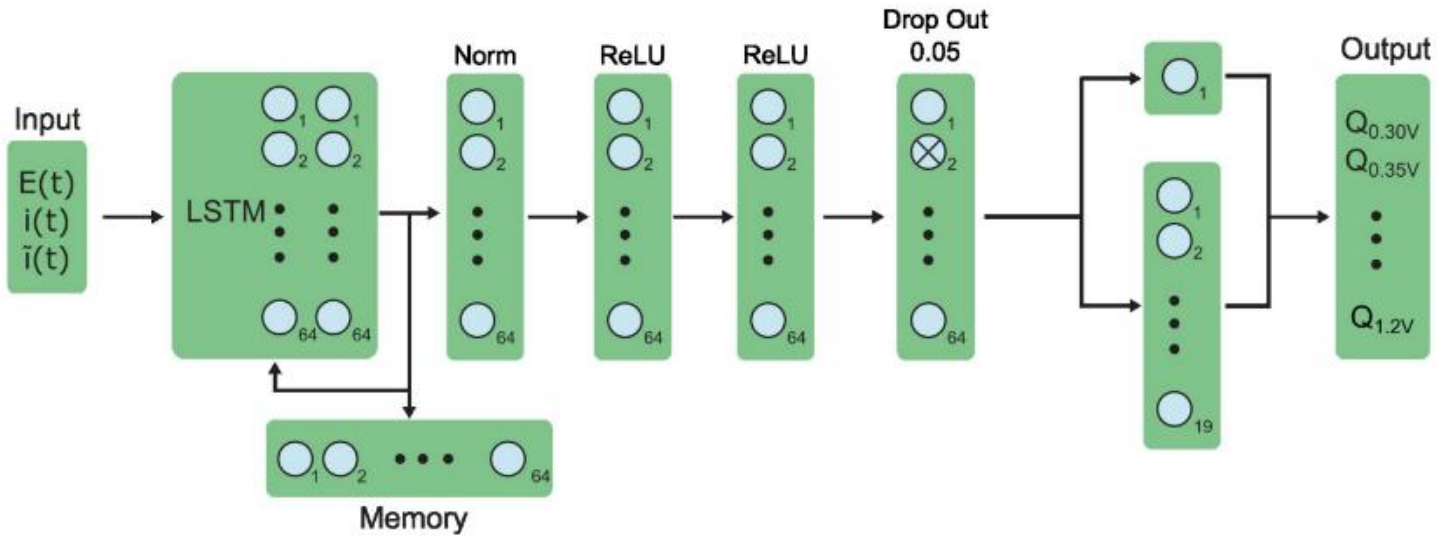

Figure S28. *Changes to the AI controller for Experiment 8.2, which was performed directly after 8.1. The output layer is a linear unit.*

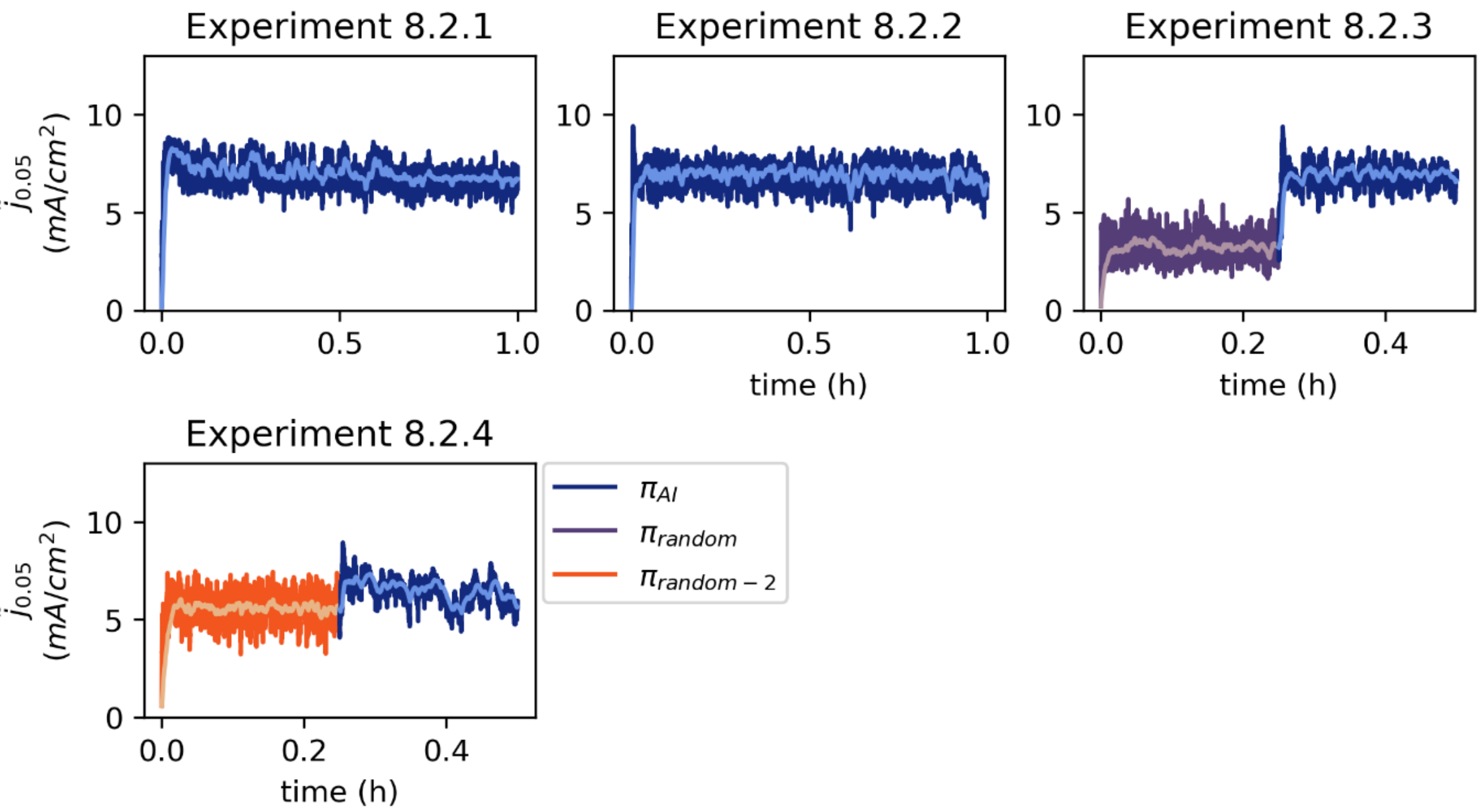

Figure S29. *Summary of data acquired under AI control of Experiment 8.2.*

Experiment 9

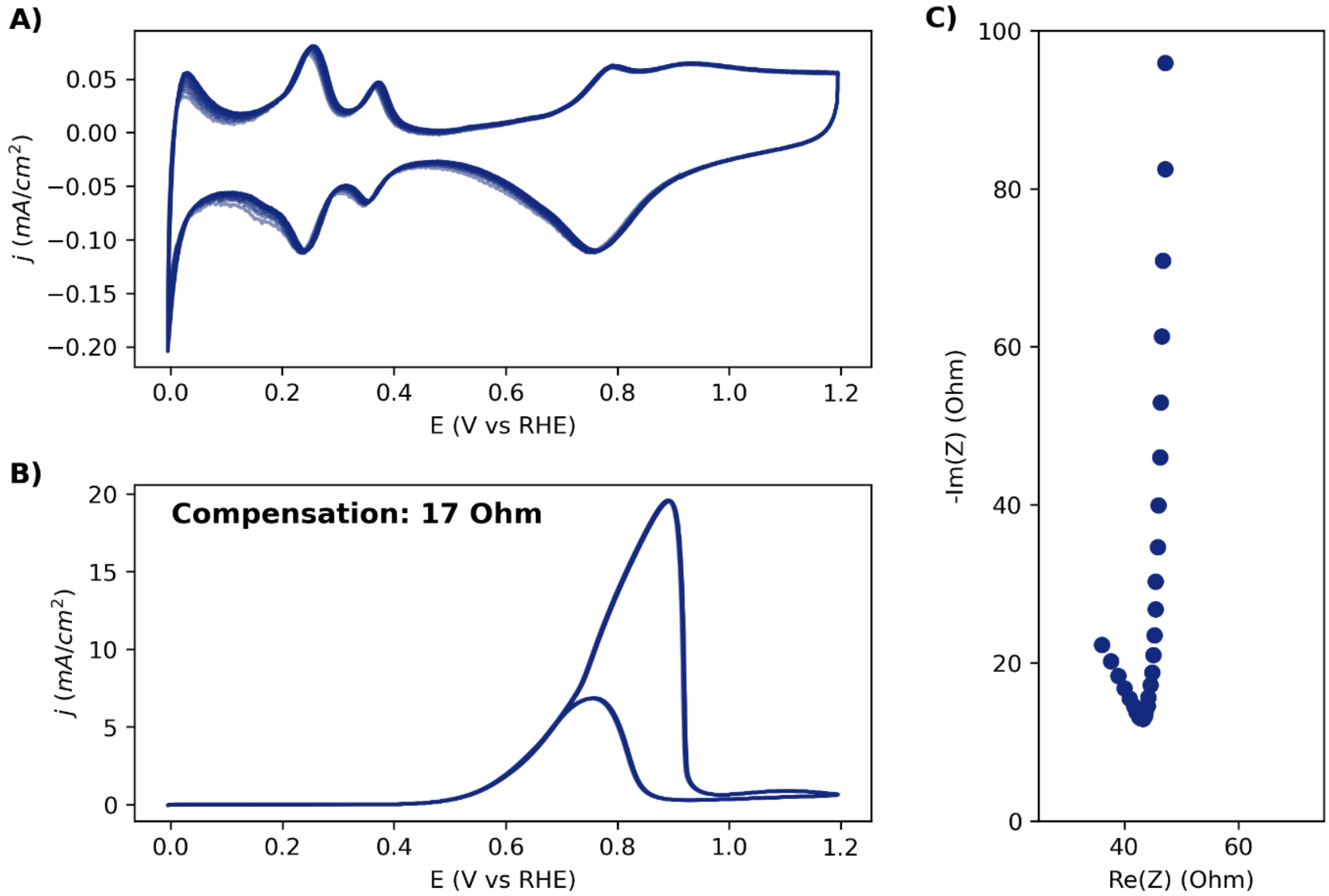

AI Experiment Details

sequence length: 1200

AI training switched off

π_{AI}: AI with 0.05 random
π_{2-step}: 0.3-0.85
π_{3-step}: 0.3-0.85-1.05
π_{random}: uniform
$\pi_{random-2}$: 0.7 to 0.85 preferred

ohmic compensation: 17 ohm

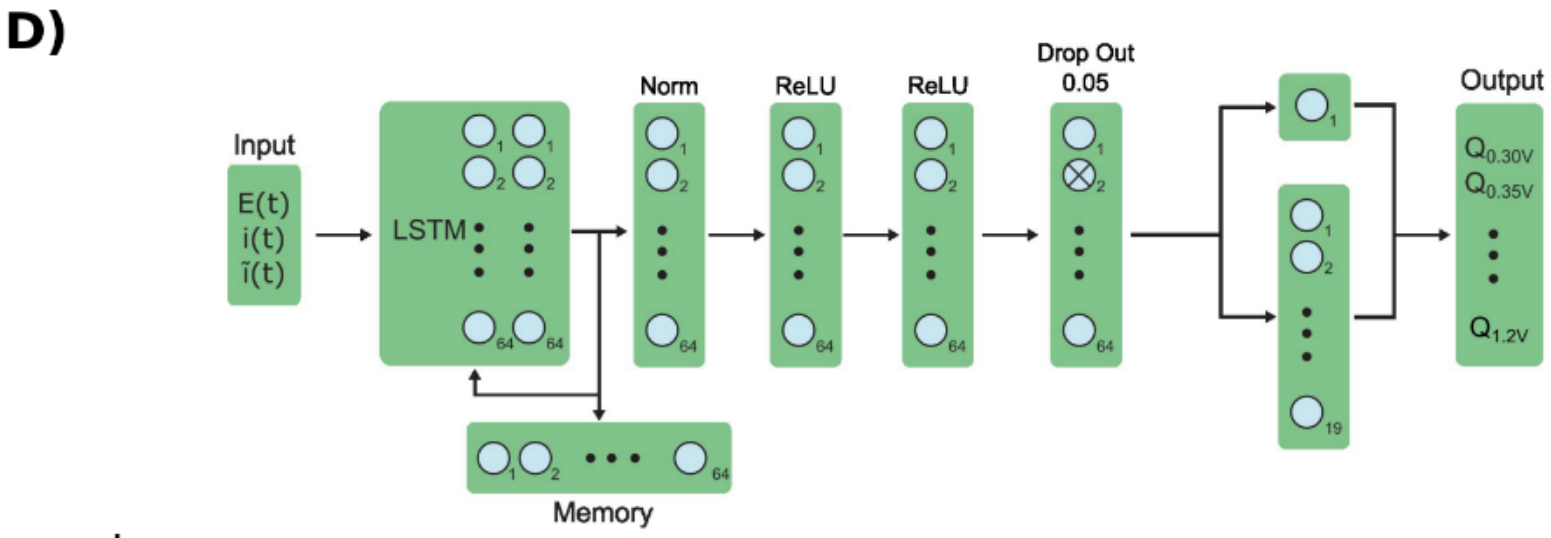

***Figure S30**. Summary of Experiment 9. **A)** Pt characterization CV at 0 rpm, 50 mV s^{-1} in 0.1 M KOH Ar$_{sat}$. **B)** Pt characterization CV at 200 rpm, 50 mV s^{-1} in 0.1 M KOH, 0.1 M glycerol Ar$_{sat}$. **C)** Nyquist plot of Pt at OCV in 0.1 M KOH, 0.1 M glycerol. **D)** AI-controller architecture used in this experiment. The output layer is a linear unit.*

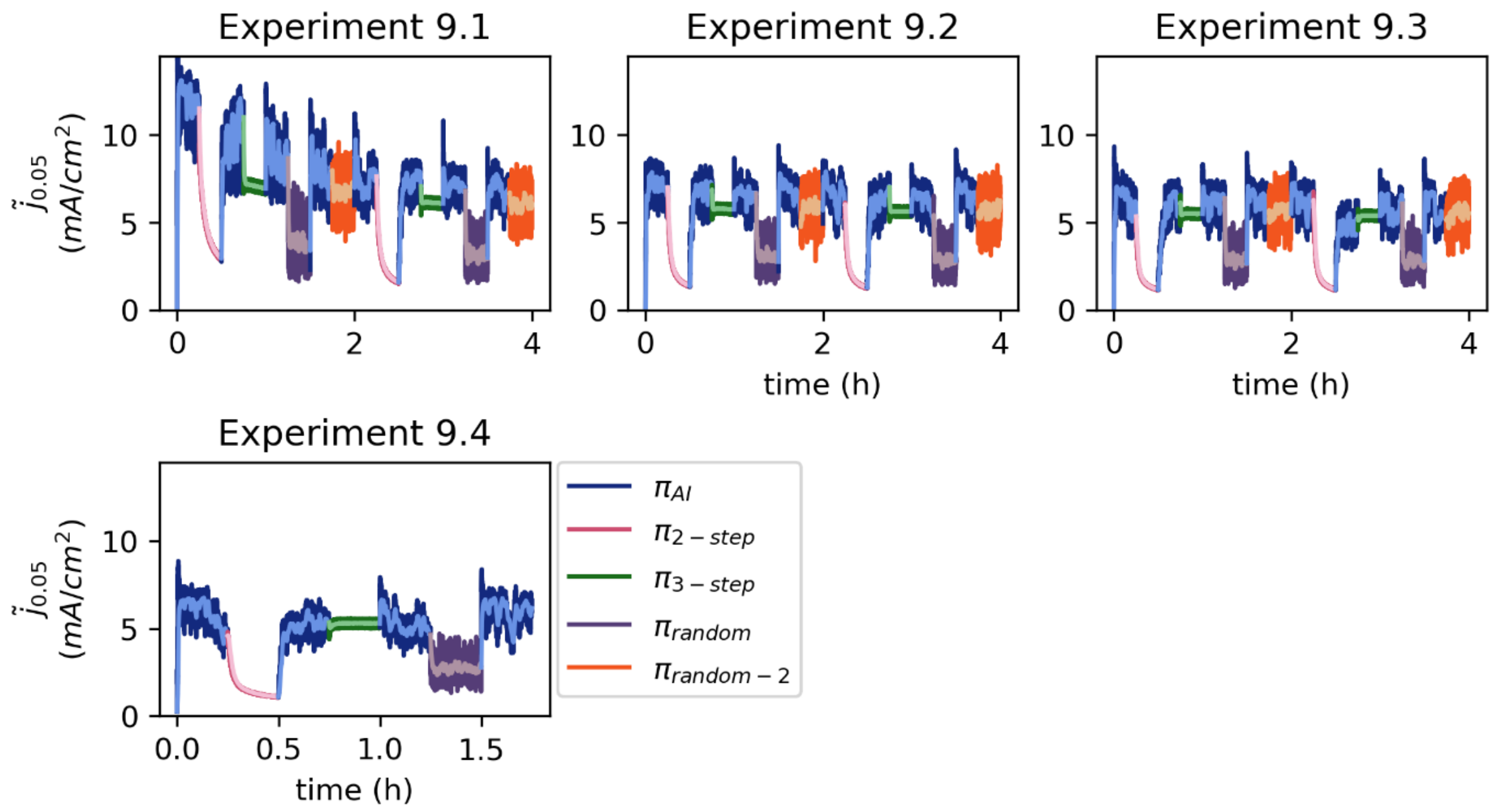

Figure S31. *Summary of data acquired under AI control of Experiment 9.*

Experiment 10

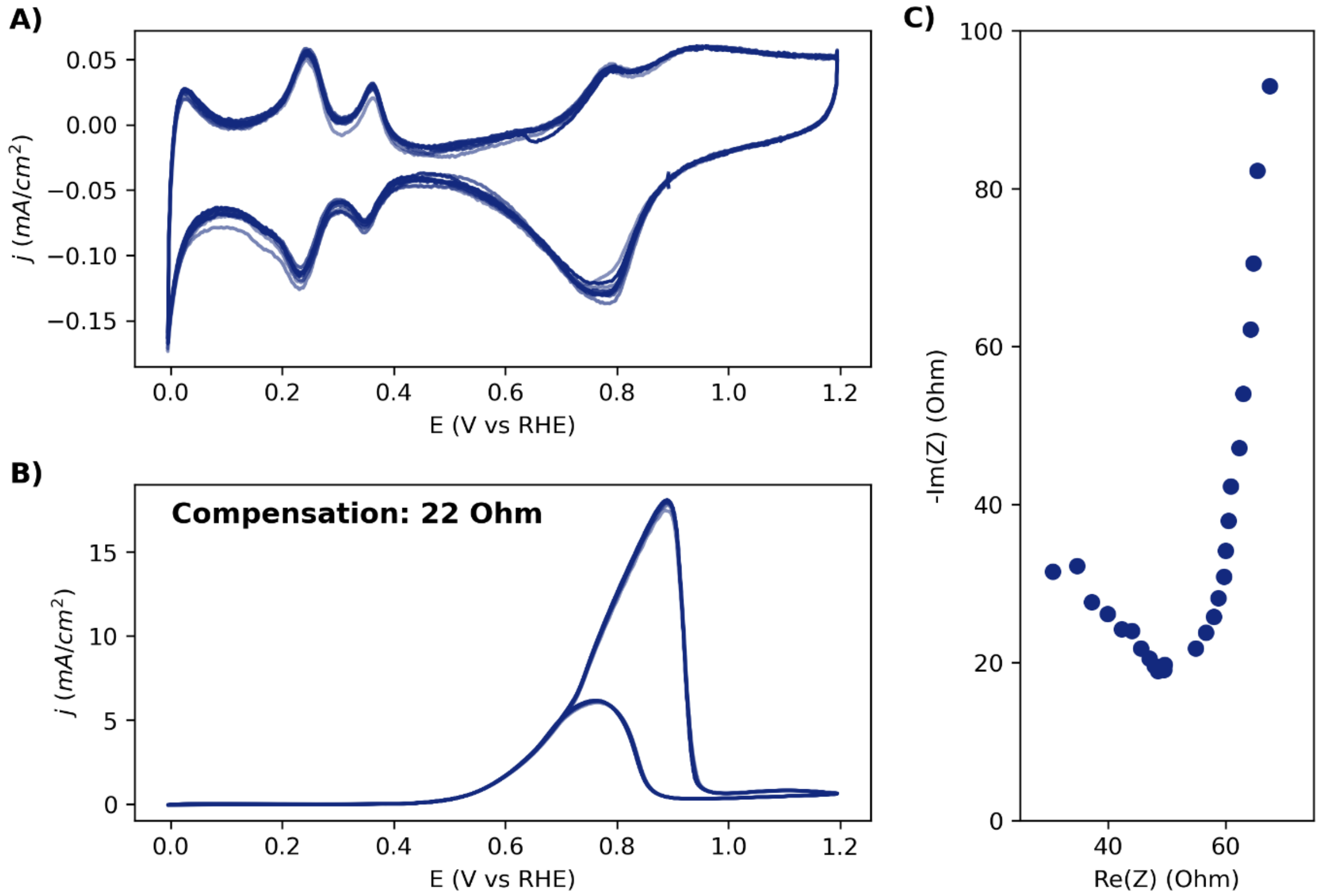

AI Experiment Details

sequence length: 120
burn in window: 60
gamma: 0.9

π_{random}: uniform

ohmic compensation: 0 ohm

reward: $i + 0.2 * \tilde{i}_{0.05}$

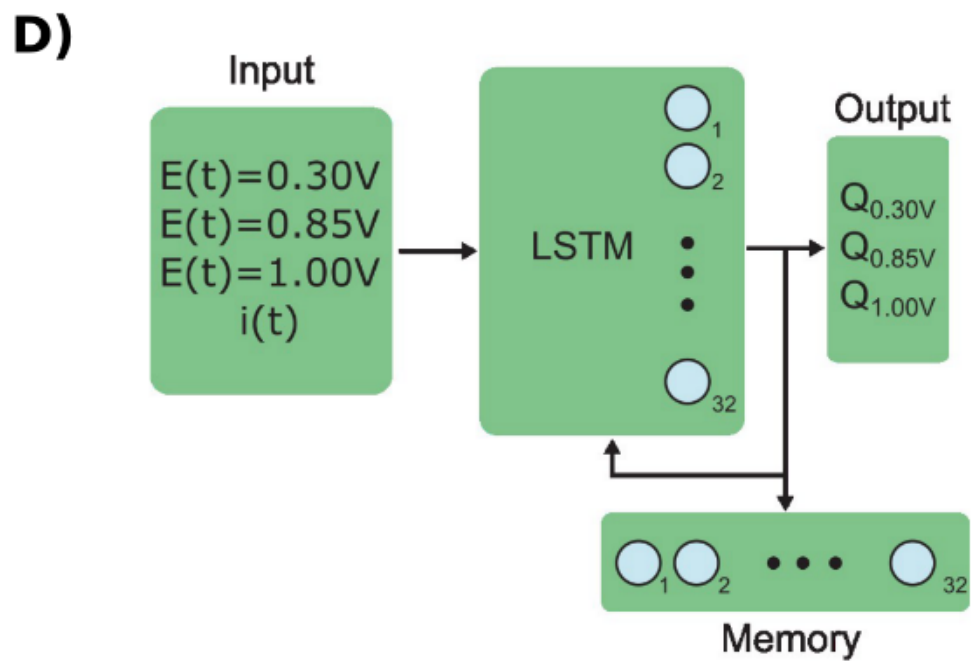

Figure S32. *Summary of Experiment 10.* ***A)*** *Pt characterization CV at 0 rpm, 50 mV s^{-1} in 0.1 M KOH Ar$_{sat}$.* ***B)*** *Pt characterization CV at 200 rpm, 50 mV s^{-1} in 0.1 M KOH, 0.1 M glycerol Ar$_{sat}$.* ***C)*** *Nyquist plot of Pt at OCV in 0.1 M KOH, 0.1 M glycerol.* ***D)*** *AI-controller architecture used in this experiment. The output layer is a linear unit.*

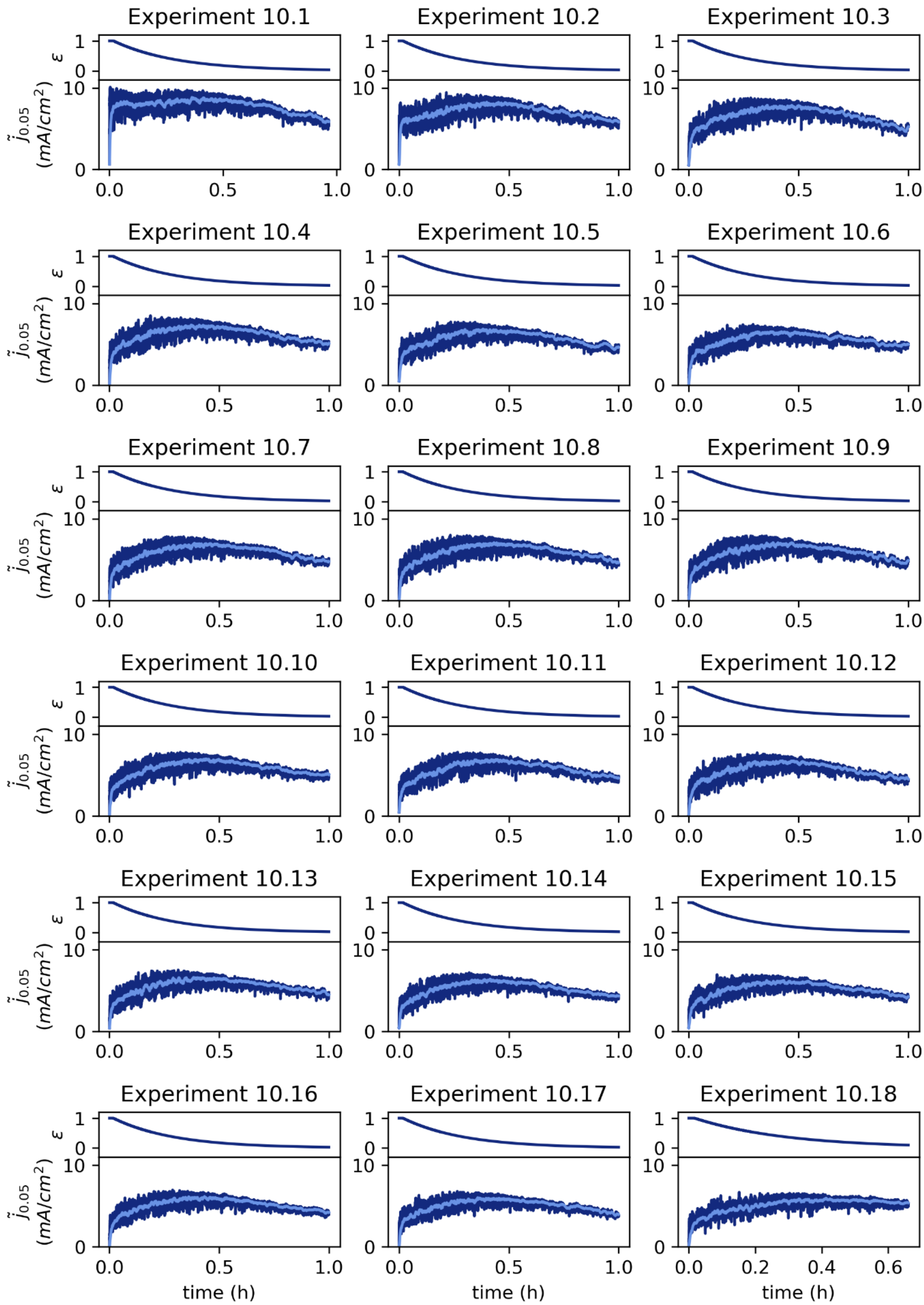

Figure S33. *Summary of data acquired under AI control of Experiment 10.*

Experiment 11.1

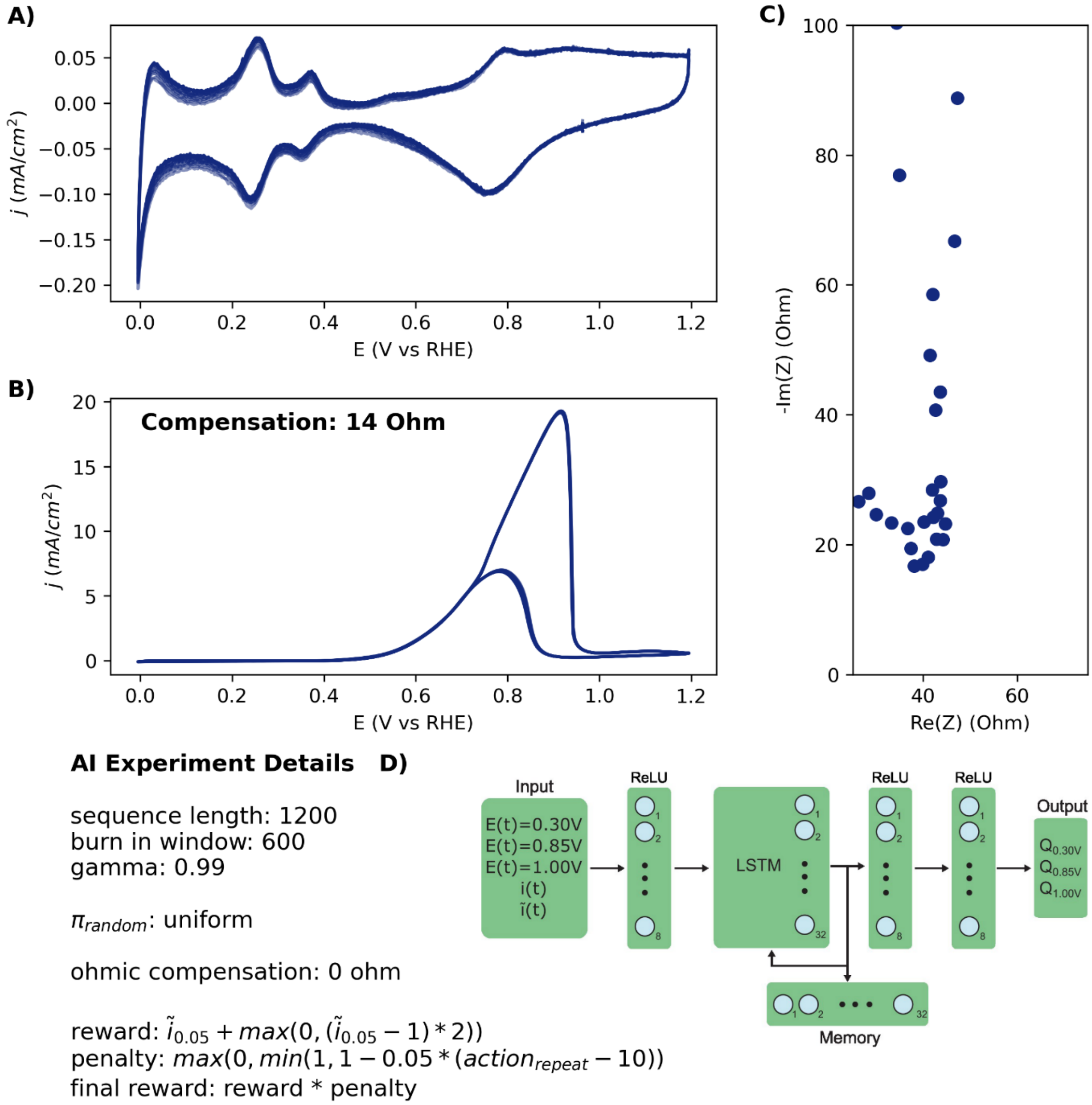

***Figure S34**. Summary of Experiment 11.1. **A)** Pt characterization CV at 0 rpm, 50 mV s^-1 in 0.1 M KOH Ar_{sat}. **B)** Pt characterization CV at 200 rpm, 50 mV s^-1 in 0.1 M KOH, 0.1 M glycerol Ar_{sat}. **C)** Nyquist plot of Pt at OCV in 0.1 M KOH, 0.1 M glycerol. **D)** AI-controller architecture used in this experiment. The output layer is a linear unit.*

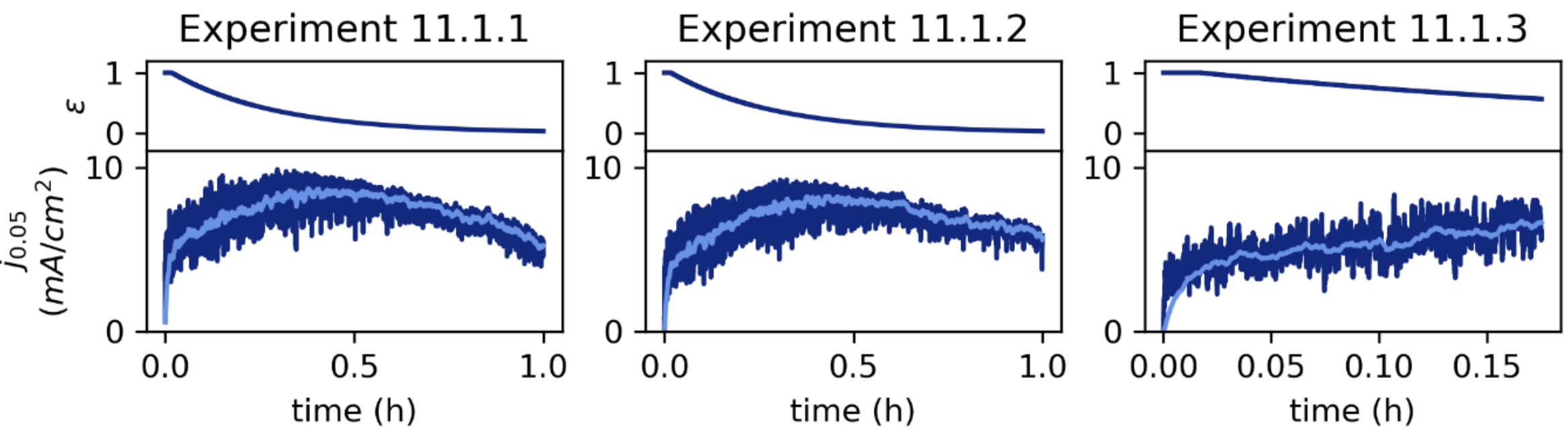

Figure S35. *Summary of data acquired under AI control of Experiment 11.1.*

Experiment 11.2

AI Experiment Details

sequence length: 1200
burn in window: 600
gamma: 0.995

π_{random}: uniform

ohmic compensation: 0 ohm

reward: $\tilde{i}_{0.05} + max(0, (\tilde{i}_{0.05} - 1) * 2))$
penalty: $max(0, min(1, 1 - 0.05 * (action_{repeat} - 10))$
final reward: reward * penalty

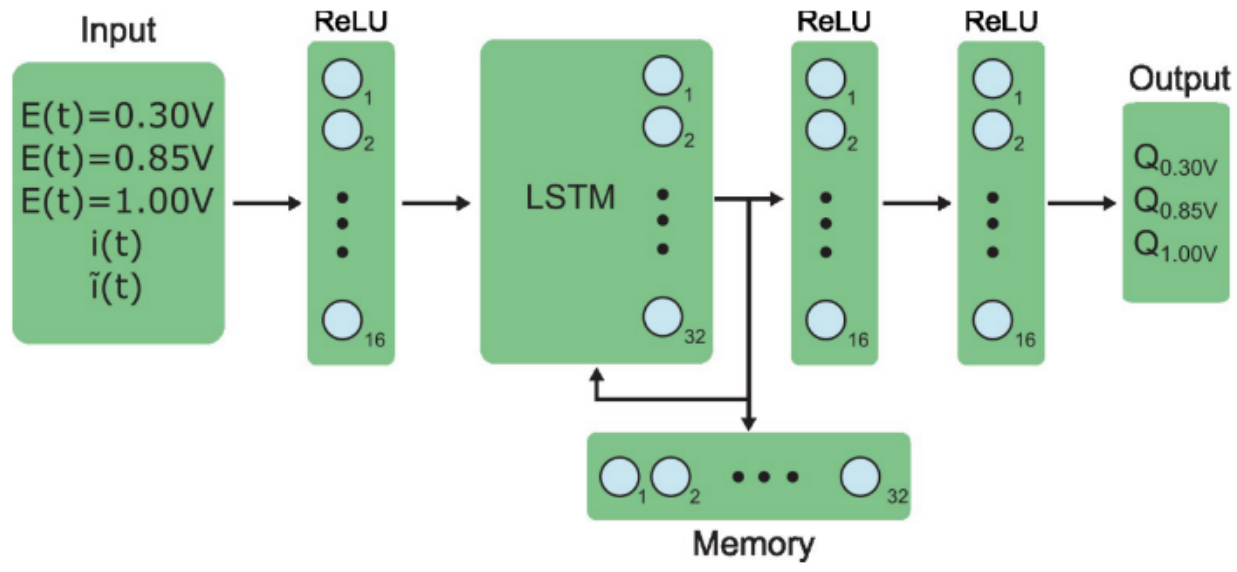

Figure S36. *Changes to the AI controller for Experiment 11.2, which was performed directly after 11.1. The output layer is a linear unit.*

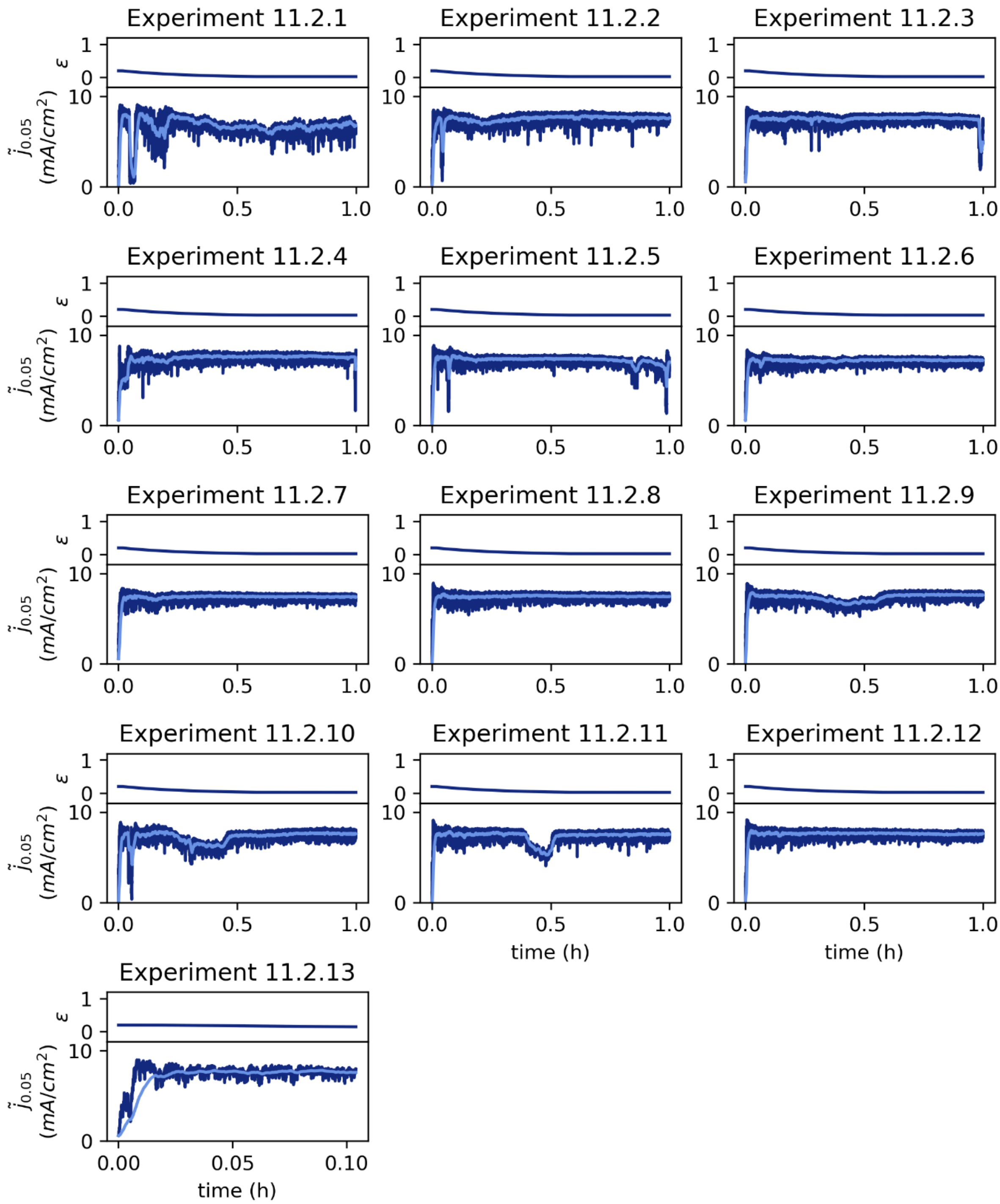

Figure S37. *Summary of data acquired under AI control of Experiment 11.2.*

Experiment 11.3

AI Experiment Details

sequence length: 1200
burn in window: 600
gamma: 0.995

π_{AI}: AI with 0.05 uniform random
π_{3-step}: 0.3-0.85-1.0

ohmic compensation: 0 ohm

reward: $\tilde{i}_{0.05} + max(0, (\tilde{i}_{0.05} - 1) * 2))$
penalty: $max(0, min(1, 1 - 0.05 * (action_{repeat} - 10))$
final reward: reward * penalty

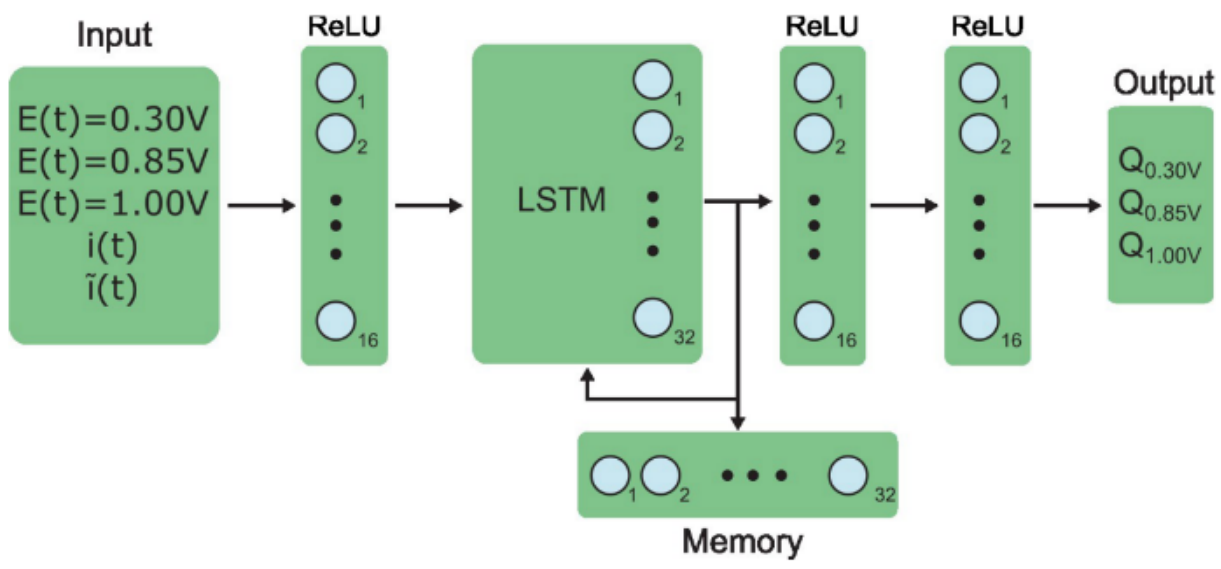

Figure S38. *Changes to the AI controller for Experiment 11.3, which was performed directly after 11.2. The output layer is a linear unit.*

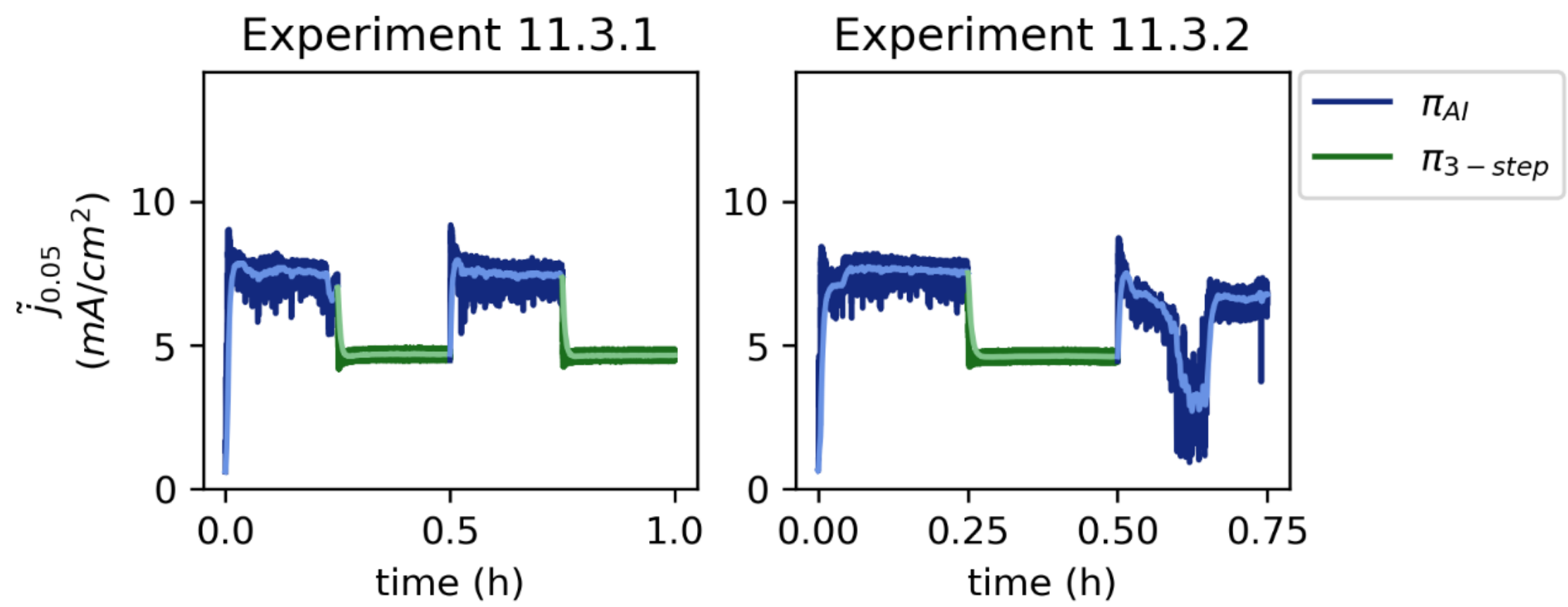

Figure S39. *Summary of data acquired under AI control of Experiment 11.3.*

Experiment 11.4

AI Experiment Details

sequence length: 1800

AI training switched off

π_{AI}: AI with 0.05 uniform random
π_{3-step}: 0.3-0.85-1.05
π_{random}: uniform
$\pi_{random-2}$: 0.7-0.85 preferred

ohmic compensation: 0 ohm

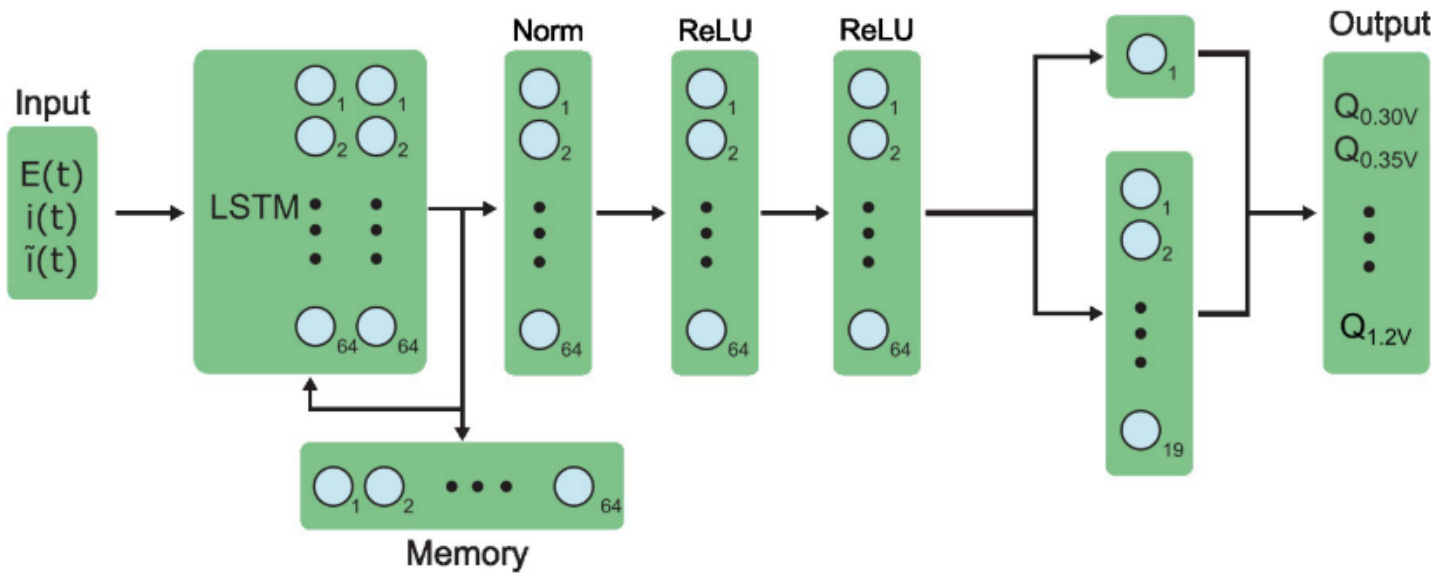

Figure S40. *Changes to the AI controller for Experiment 11.4, which was performed directly after 11.3. The output layer is a linear unit.*

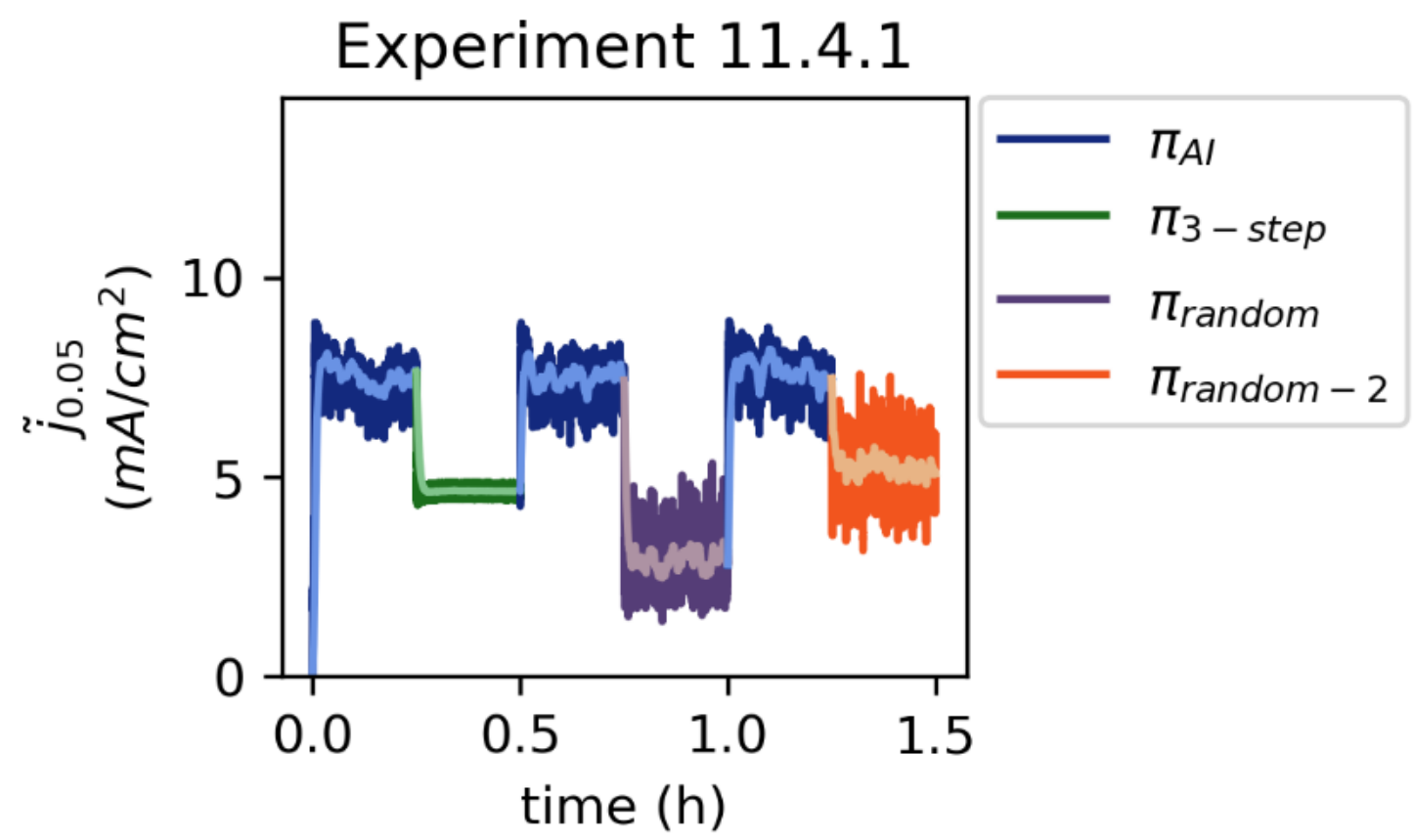

Figure S41. *Summary of data acquired under AI control of Experiment 11.4.*

Experiment 12

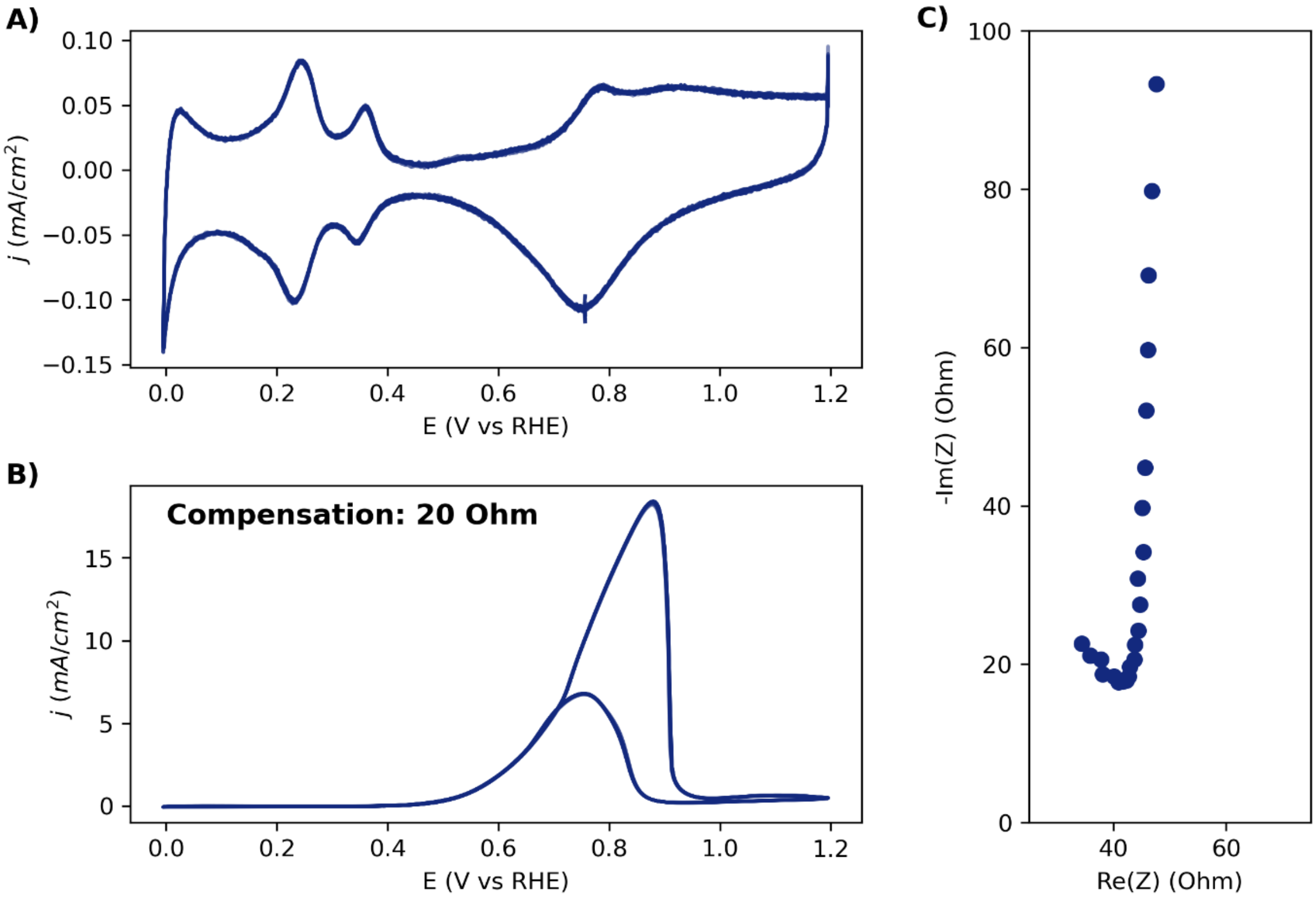

AI Experiment Details

sequence length: 1800

AI training switched off

π_{AI}: AI with 0.05 uniform random
π_{3-step}: 0.3-0.85-1.0
π_{random}: uniform
$\pi_{random-2}$: 0.7 to 0.85 preferred

ohmic compensation: 0 ohm

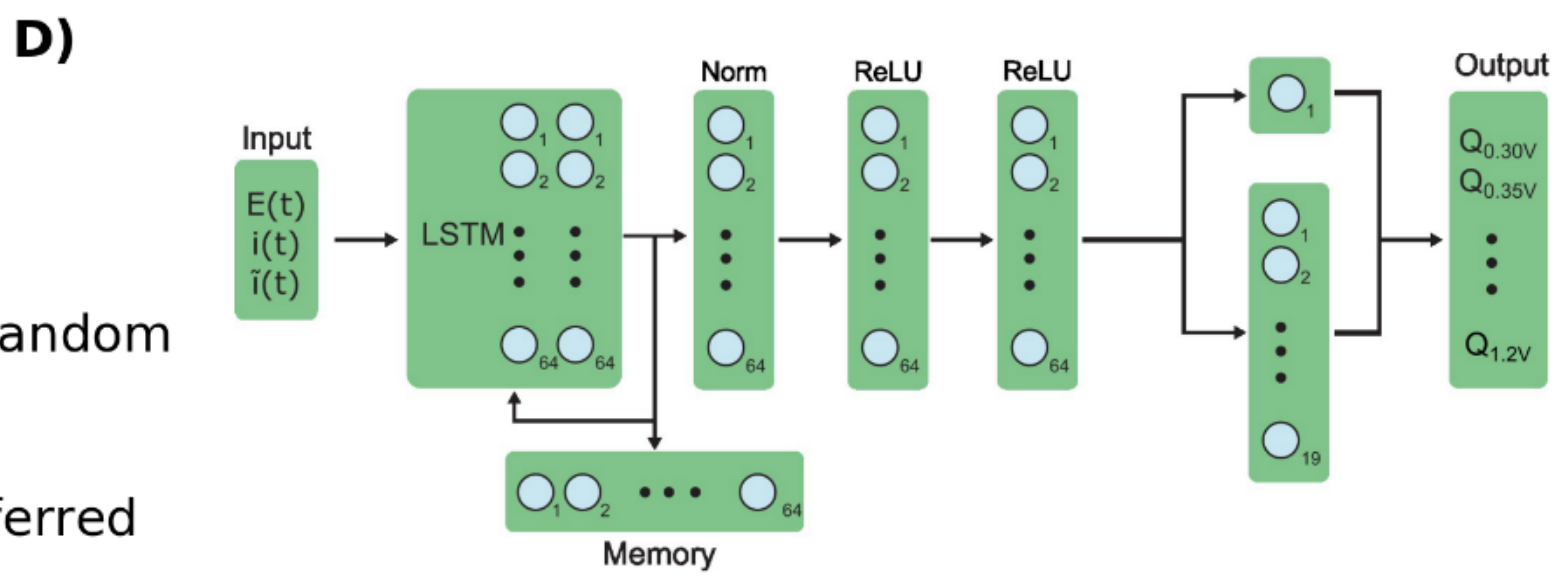

***Figure S42**. Summary of Experiment 12. **A)** Pt characterization CV at 0 rpm, 50 mV s^{-1} in 0.1 M KOH Ar_{sat}. **B)** Pt characterization CV at 200 rpm, 50 mV s^{-1} in 0.1 M KOH, 0.1 M glycerol Ar_{sat}. **C)** Nyquist plot of Pt at OCV in 0.1 M KOH, 0.1 M glycerol. **D)** AI-controller architecture used in this experiment. The output layer is a linear unit.*

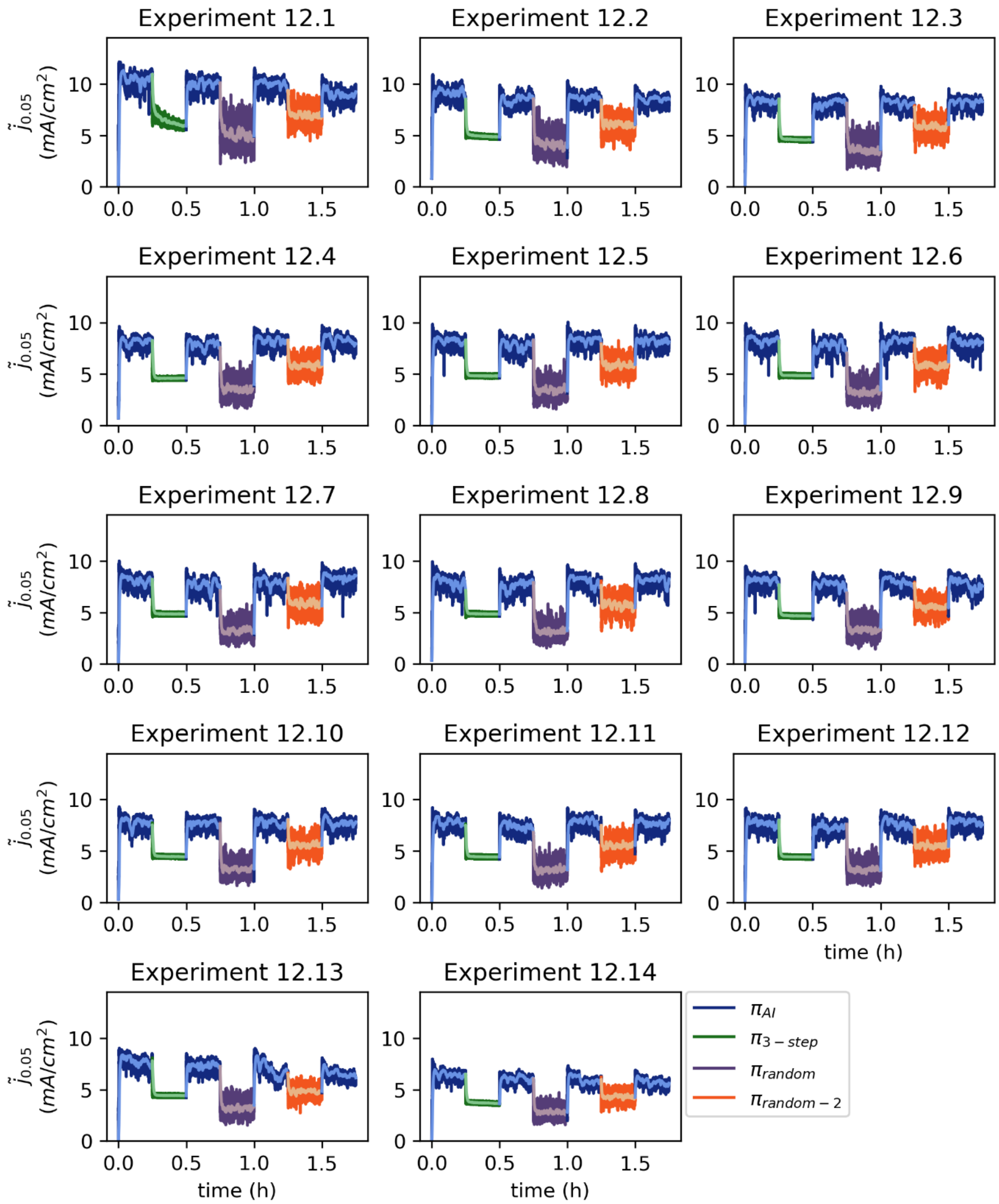

Figure S43. *Summary of data acquired under AI control of Experiment 12.*

VI AI Models

VI.1 AI Model 1.1

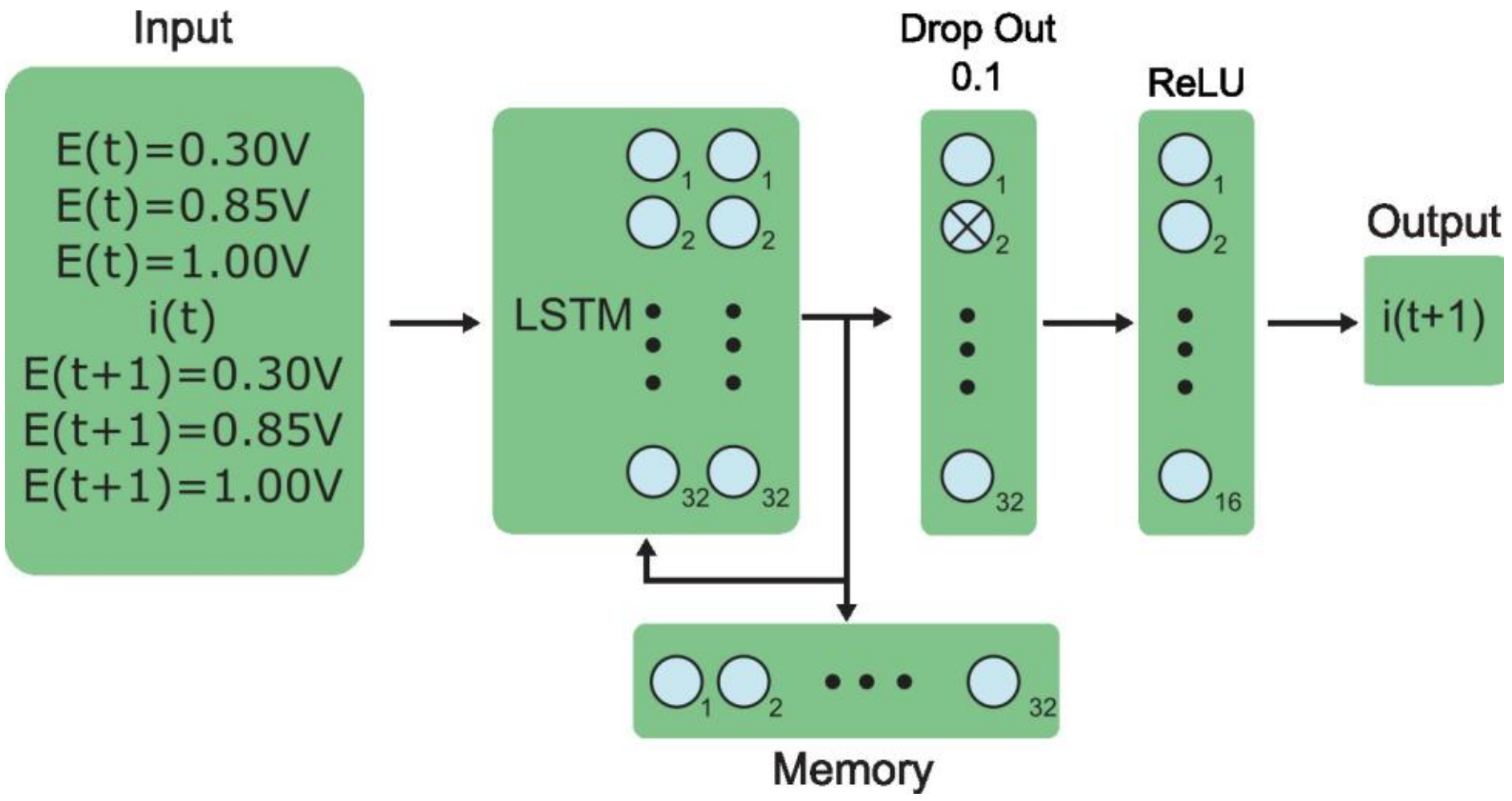

Figure S44. *The structure of AI Model 1.1. The output layer is a fully connected linear unit.*

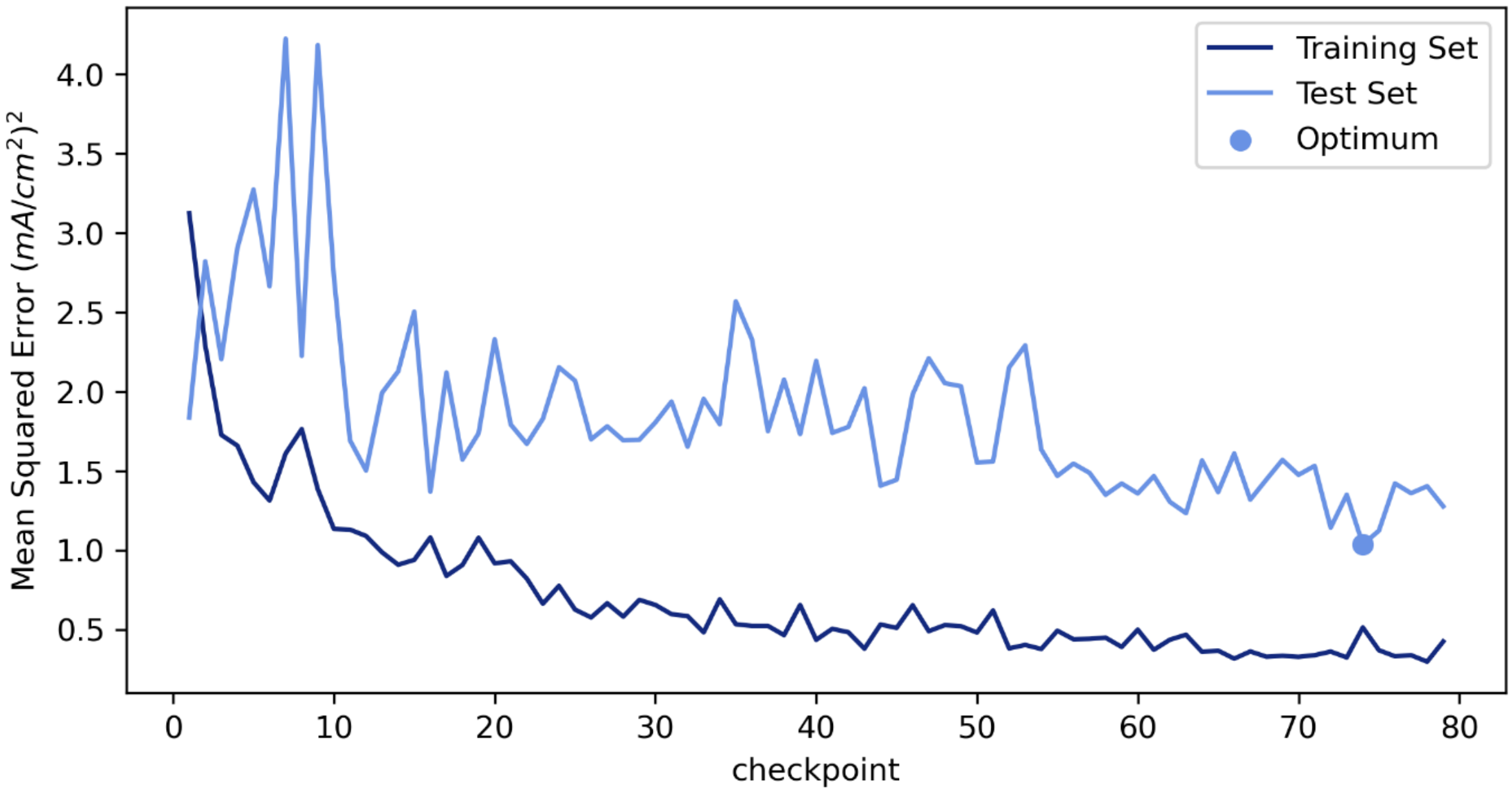

Figure S45. *Cross-validation scores vs. checkpoint number. The training and test datasets were segmented into chunks of 30 000 observations, and any remaining tails containing fewer than 30 000 observations were discarded. For each segment, the first 600 points were used to burn in the hidden state, while the remaining 29 400 points were used to evaluate the checkpoint performance.*

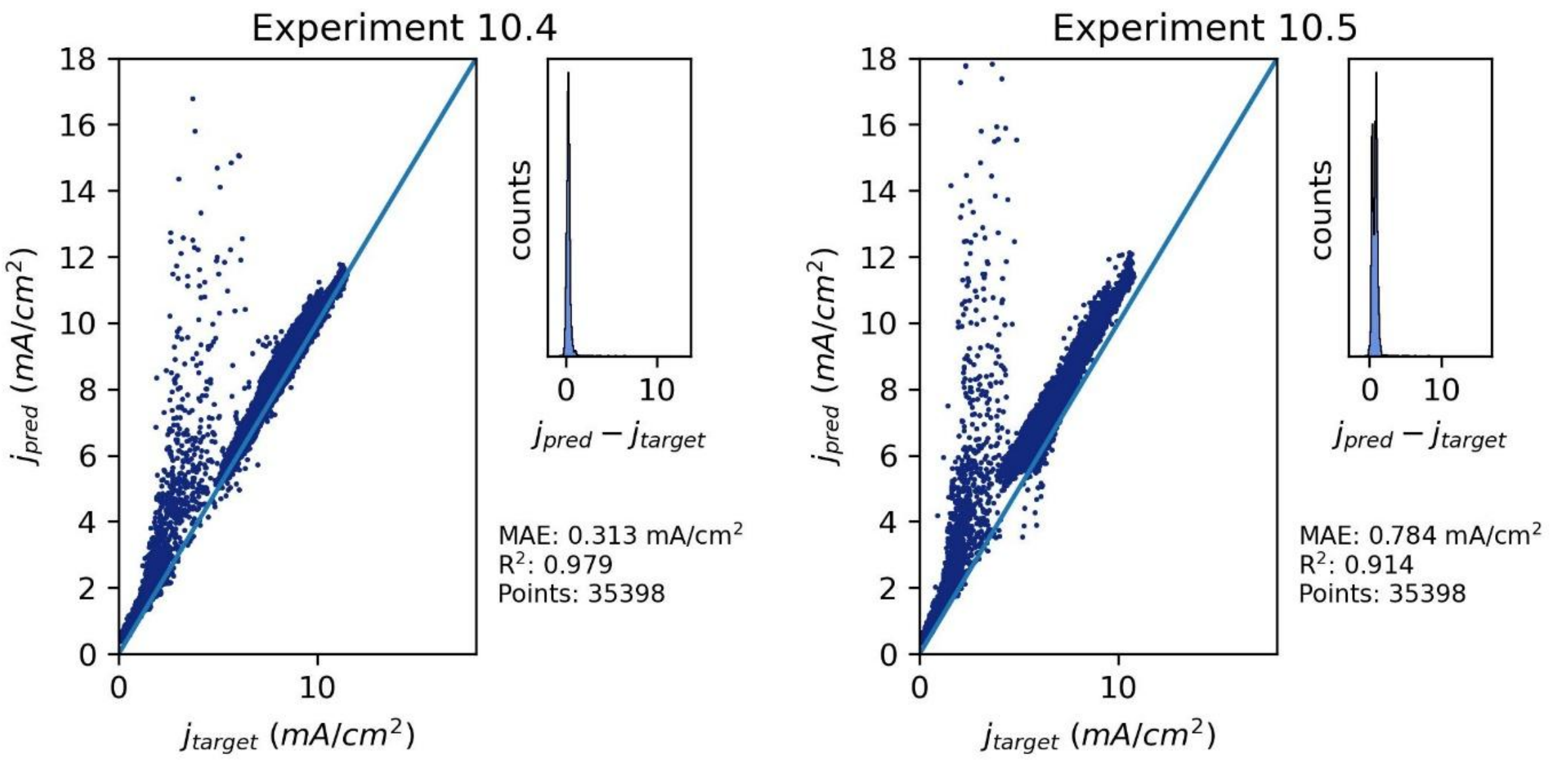

Figure S46. *Parity plots of the test set using a free running simulation with a burn in of 600 steps and the optimal checkpoint.*

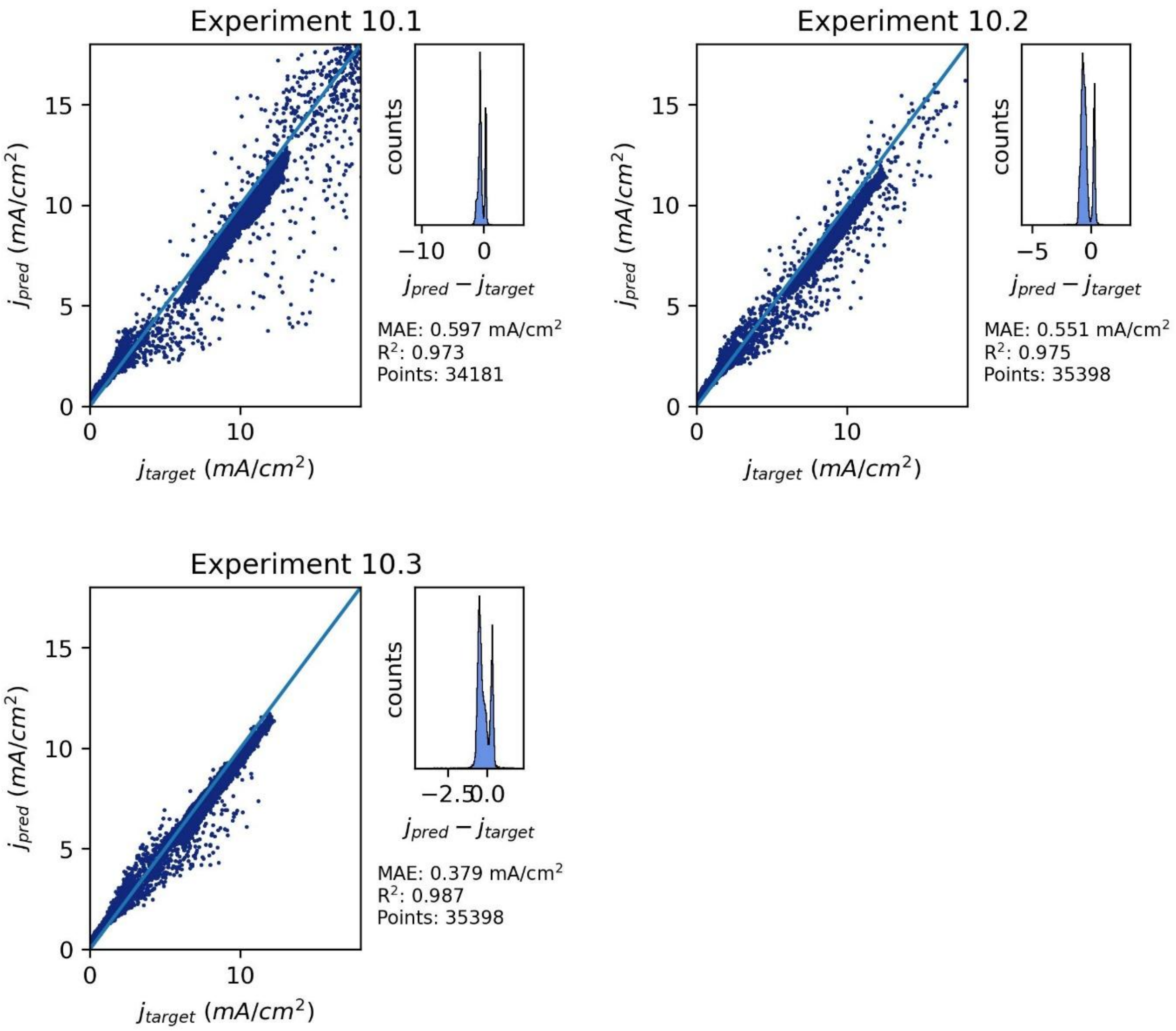

Figure S47. *Parity plots of the training set using a free running simulation with a burn in of 600 steps and the optimal checkpoint.*

VI.2 AI Model 1.2

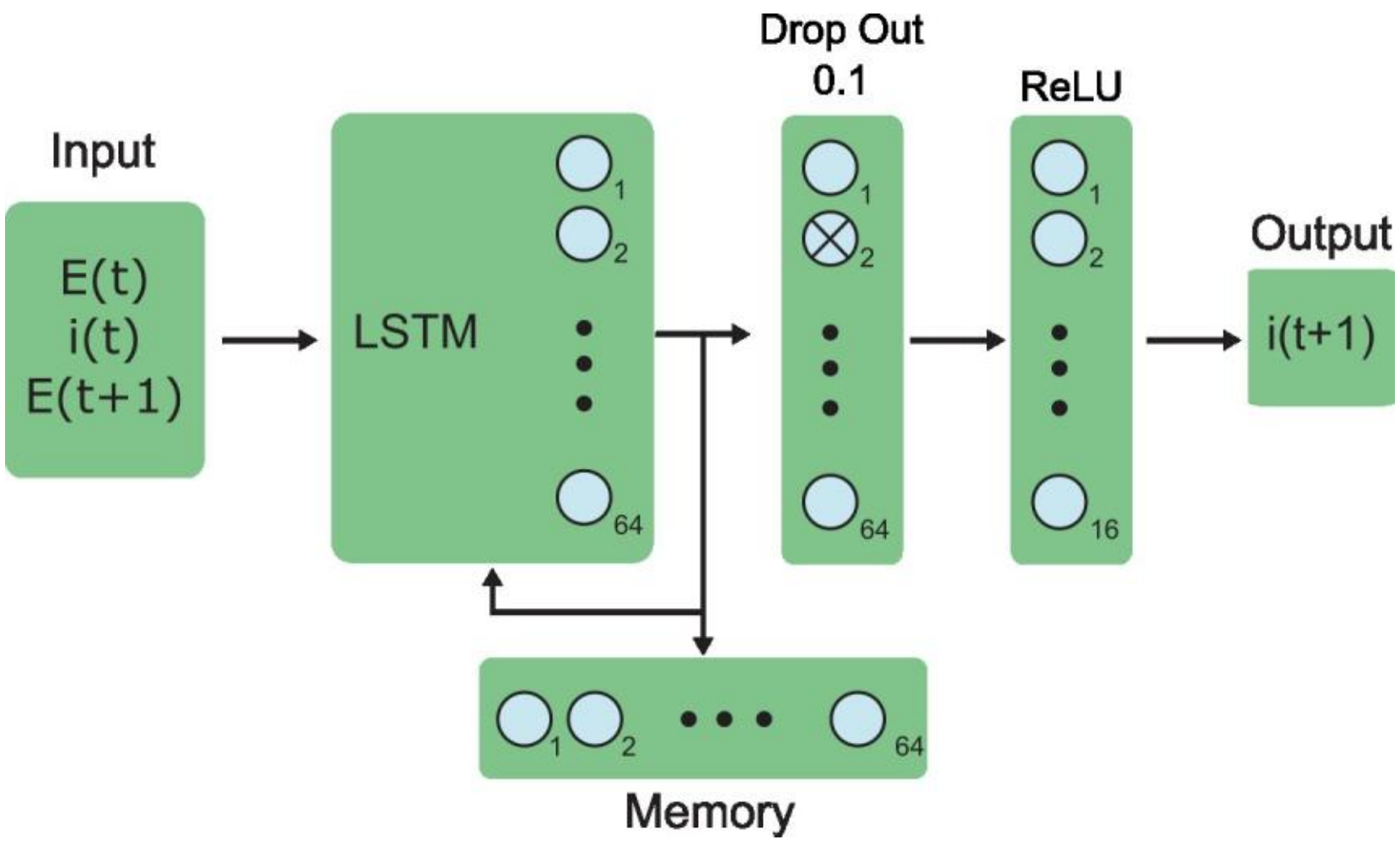

Figure S48. *The structure of AI Model 1.2. The output layer is a fully connected linear unit.*

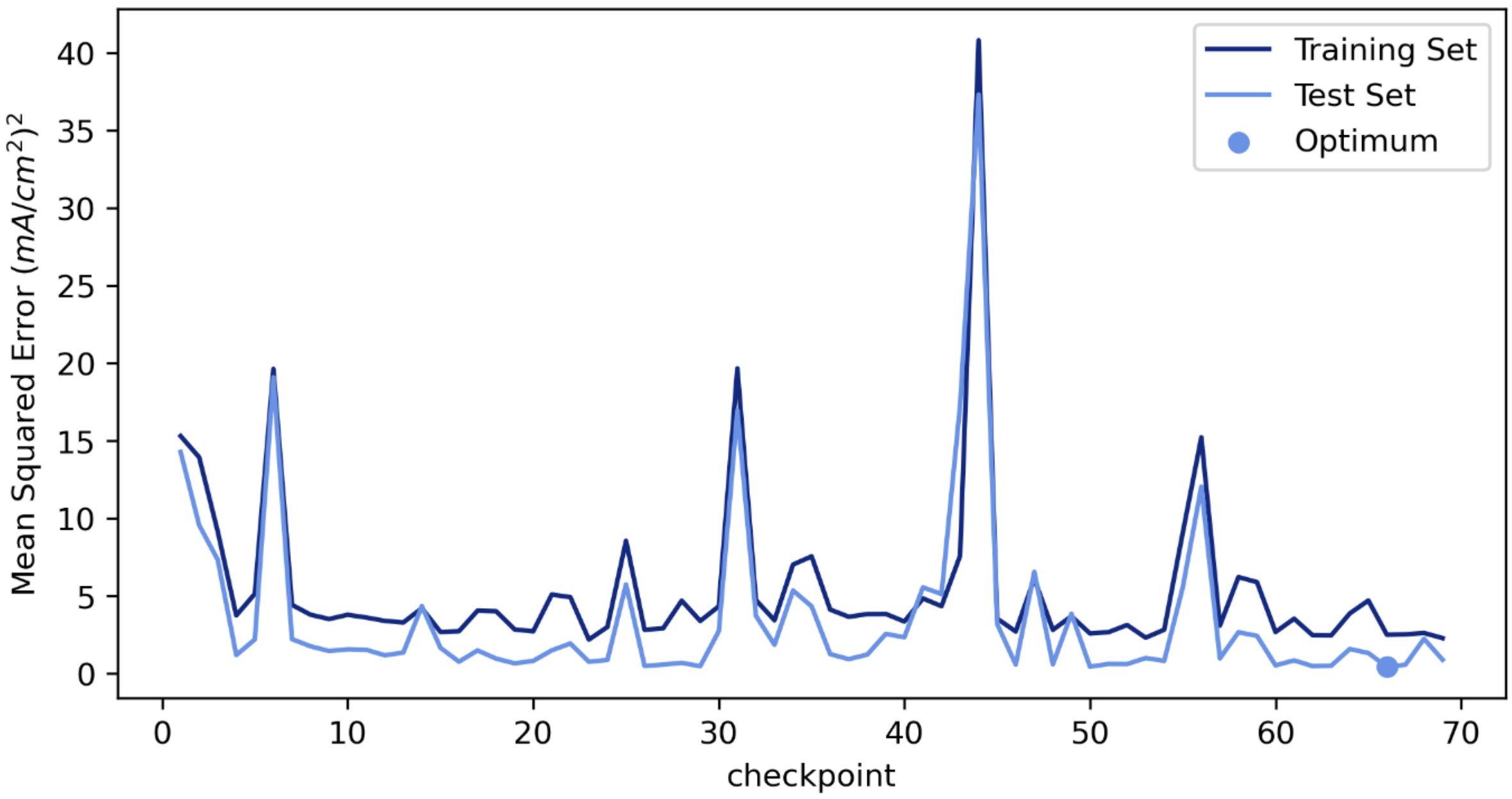

Figure S49. *Cross-validation scores vs. checkpoint number. The training and test datasets were segmented into chunks of 30 000 observations, and any remaining tails containing fewer than 30 000 observations were discarded. For each segment, the first 600 points were used to burn in the hidden state, while the remaining 29 400 points were used to evaluate the checkpoint performance.*

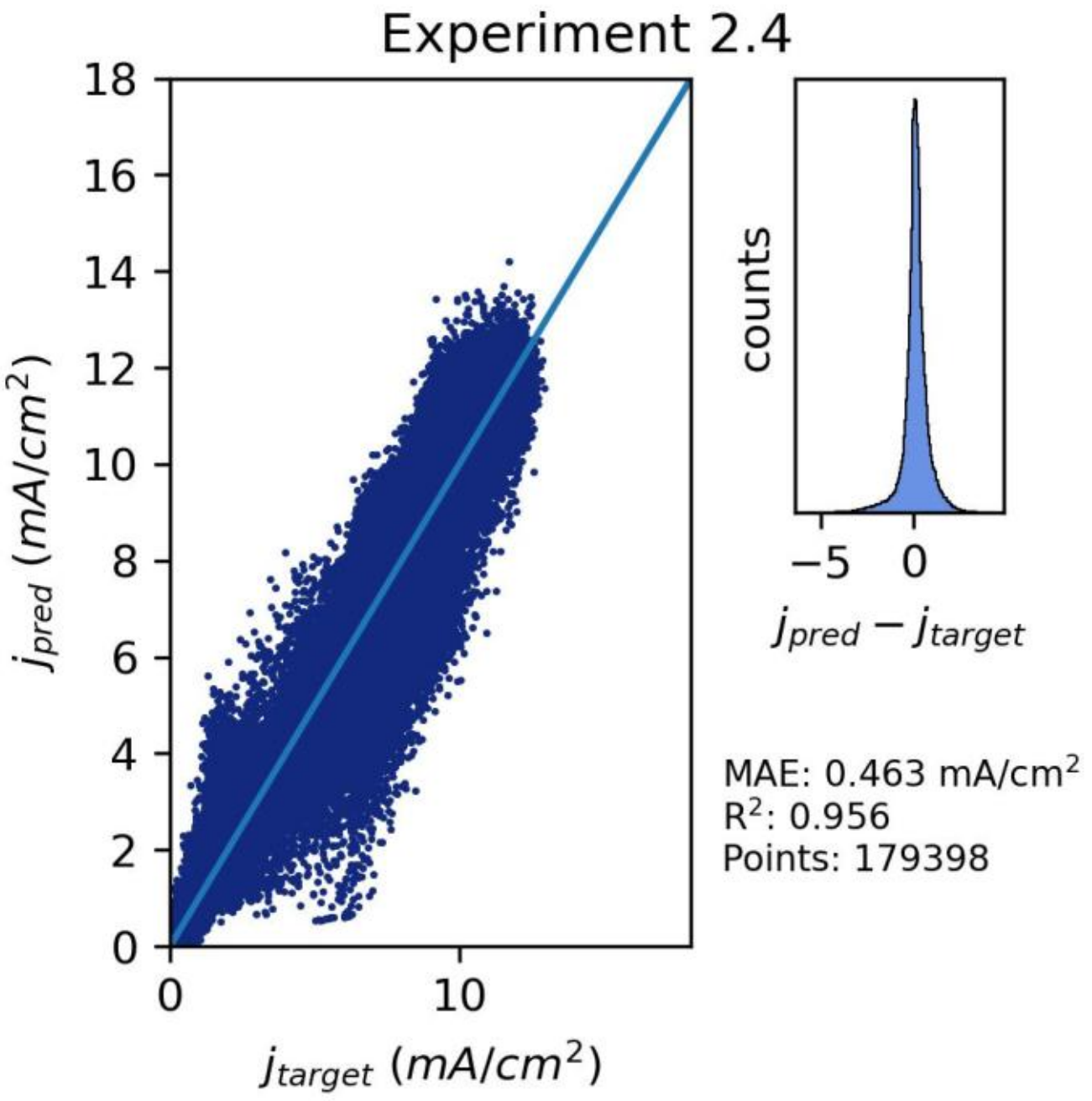

Figure S50. *Parity plots of the test set using a free running simulation with a burn in of 600 steps and the optimal checkpoint.*

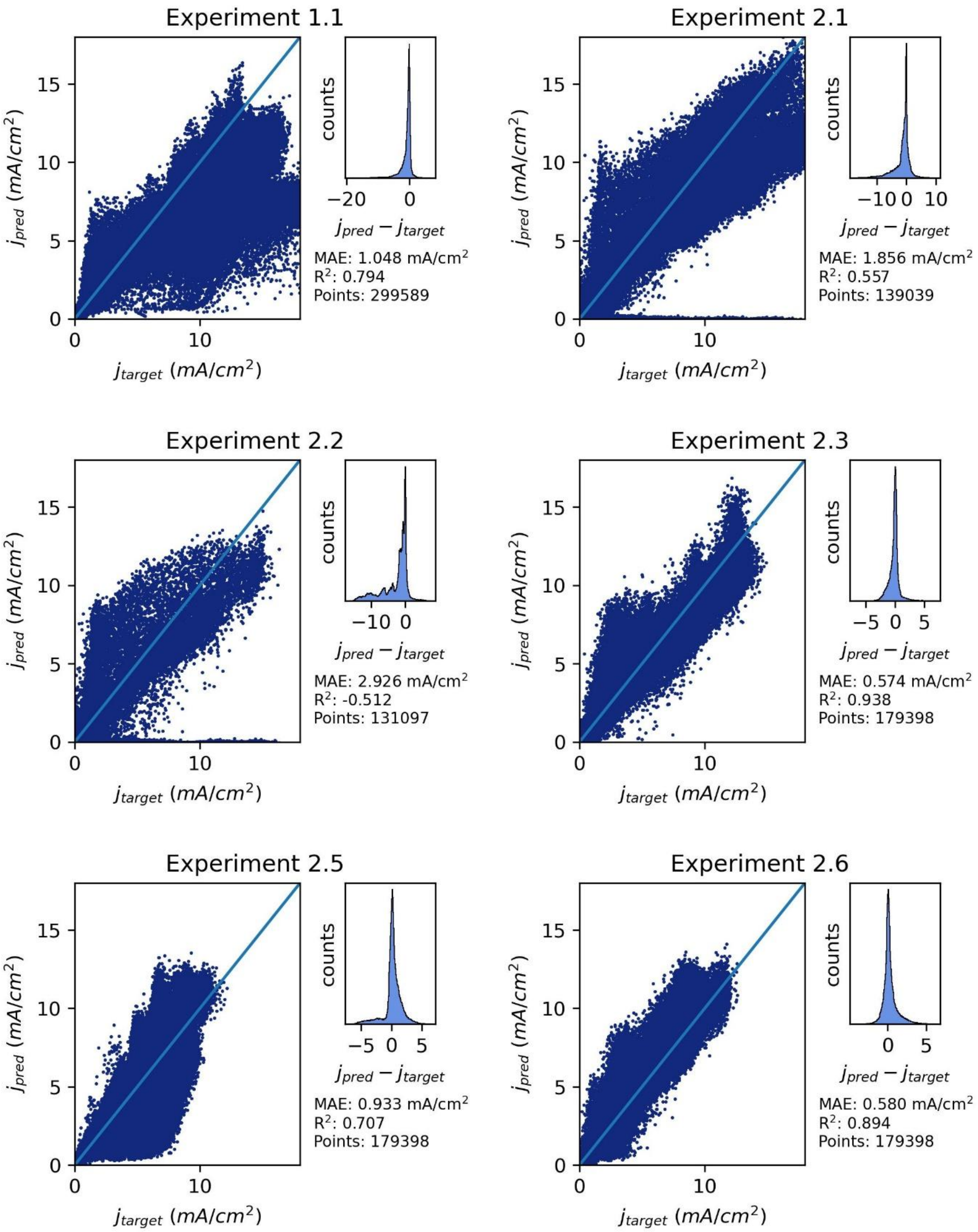

Experiment 1.1
MAE: 1.048 mA/cm2
R2: 0.794
Points: 299589
Experiment 2.1
MAE: 1.856 mA/cm2
R2: 0.557
Points: 139039
Experiment 2.2
MAE: 2.926 mA/cm2
R2: -0.512
Points: 131097
Experiment 2.3
MAE: 0.574 mA/cm2
R2: 0.938
Points: 179398
Experiment 2.5
MAE: 0.933 mA/cm2
R2: 0.707
Points: 179398
Experiment 2.6
MAE: 0.580 mA/cm2
R2: 0.894
Points: 179398
j_pred (mA/cm2)
j_target (mA/cm2)
counts
j_pred − j_target

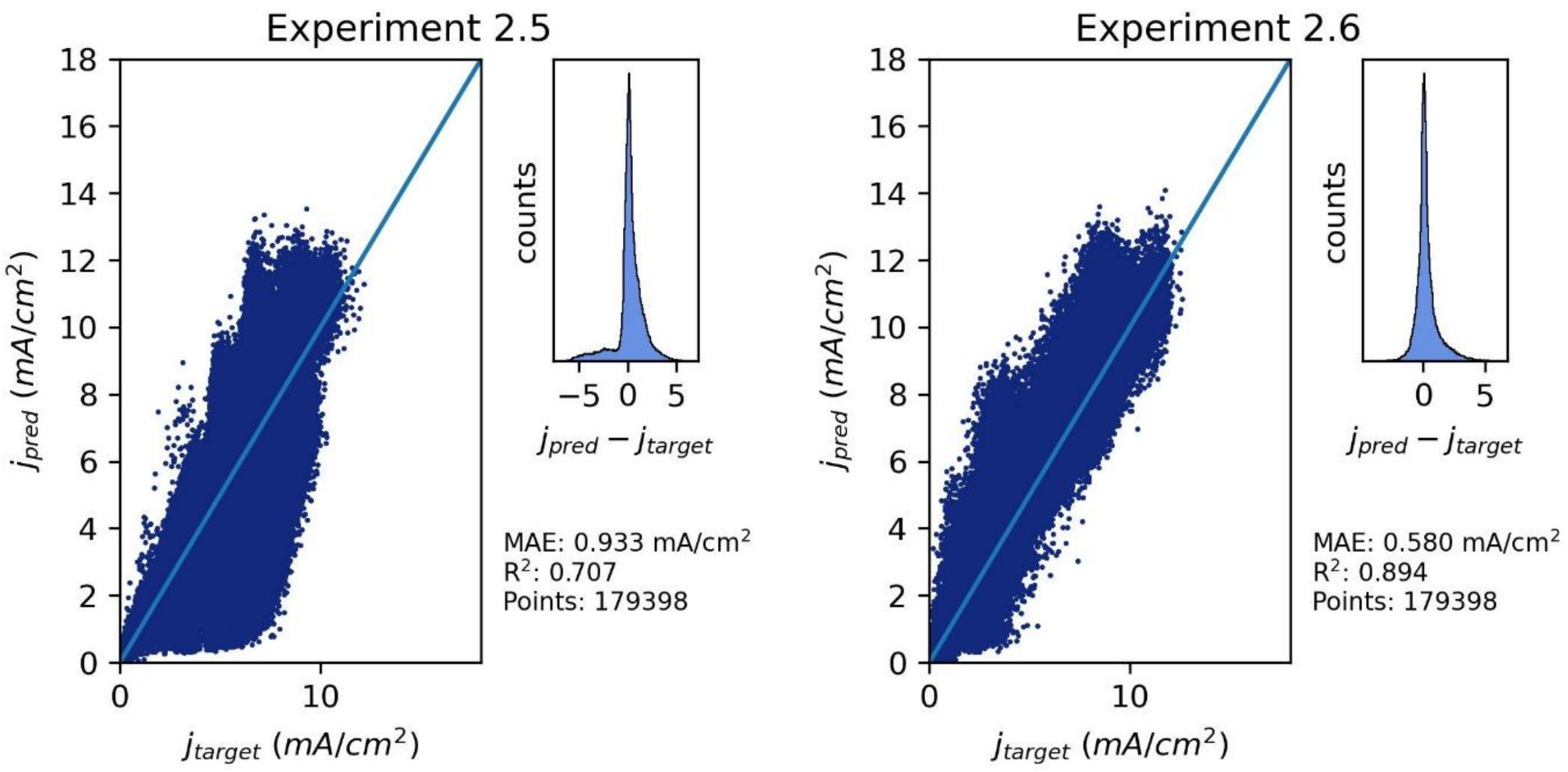

Figure S51. *Parity plots of the training set using a free running simulation with a burn in of 600 steps and the optimal checkpoint.*

VI.3 AI Model 2.1

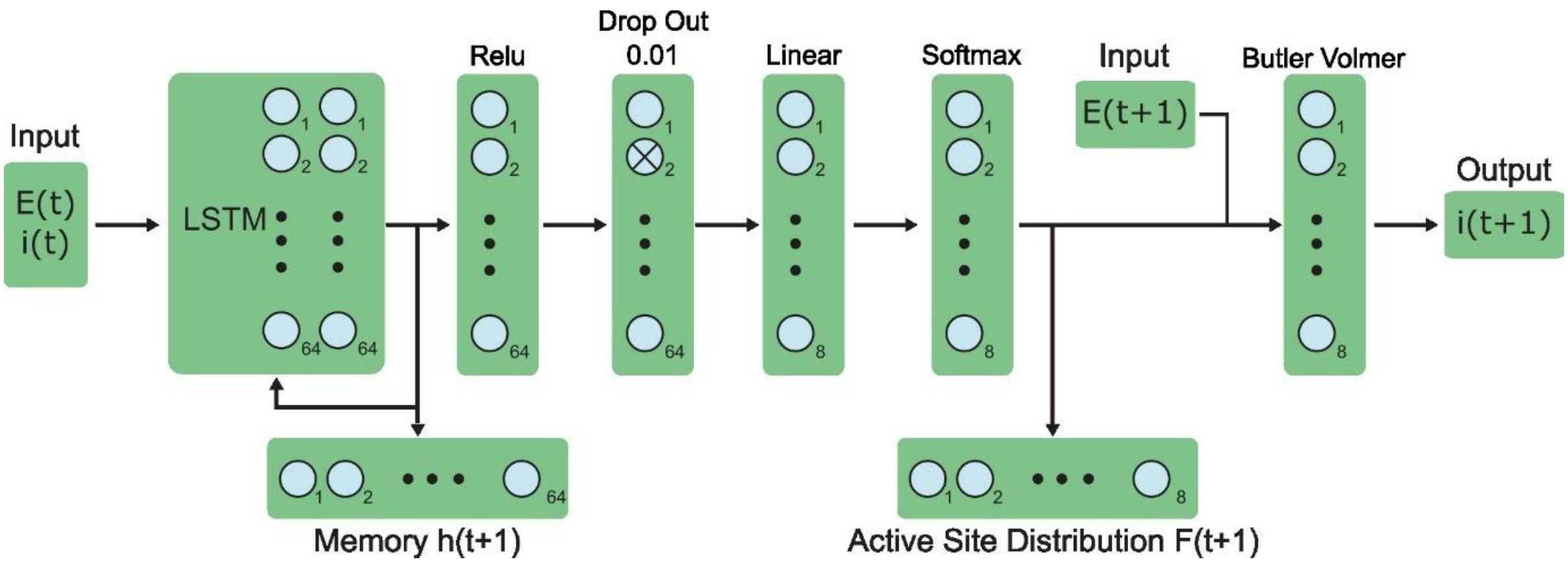

Figure S52. *The structure of AI Model 2.1. The output layer is a sum of all incoming units.*

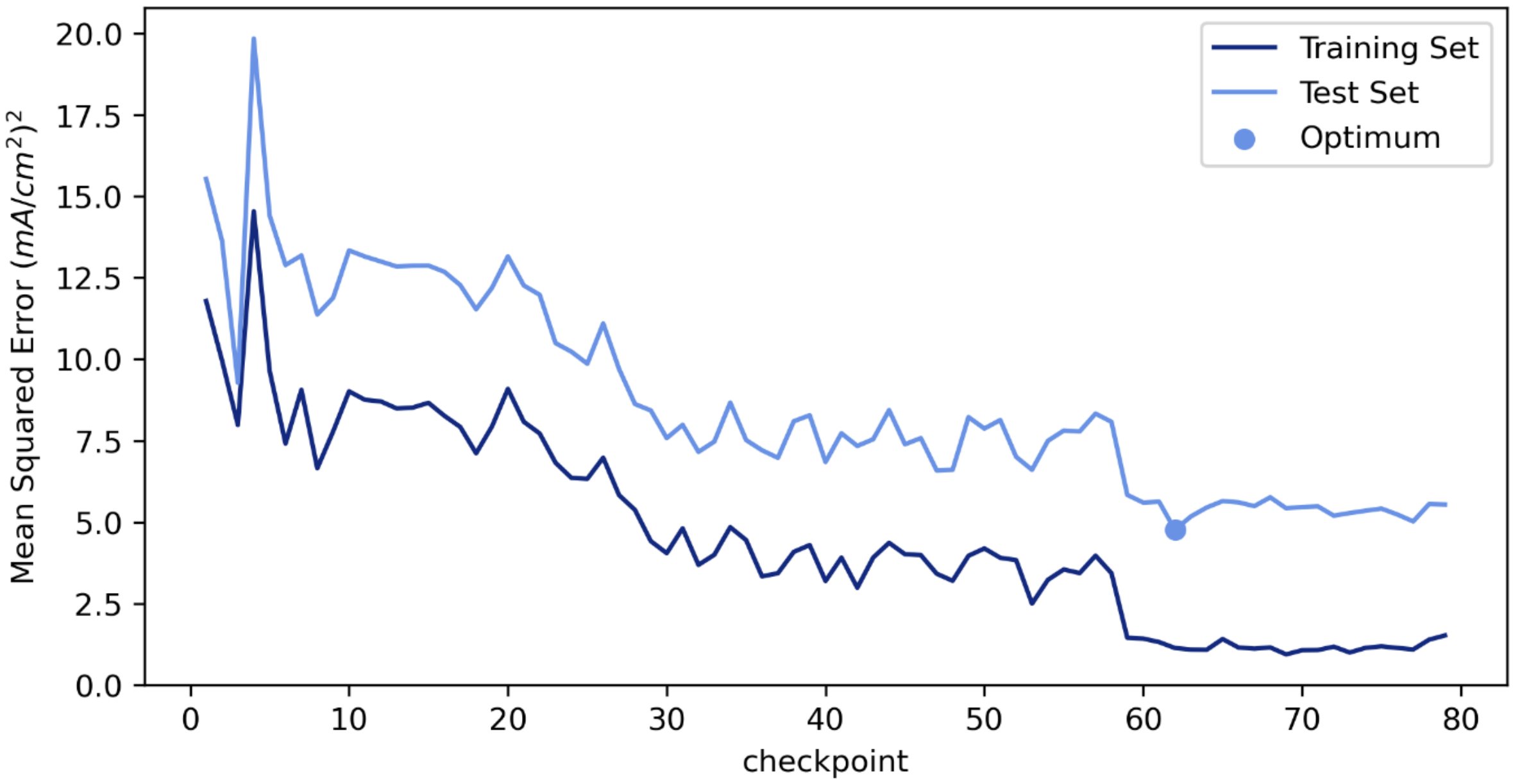

Figure S53. *Cross-validation scores vs. checkpoint number. The training and test datasets were segmented into chunks of 30 000 observations, and any remaining tails containing fewer than 30 000 observations were discarded. For each segment, the first 600 points were used to burn in the hidden state, while the remaining 29 400 points were used to evaluate the checkpoint performance.*

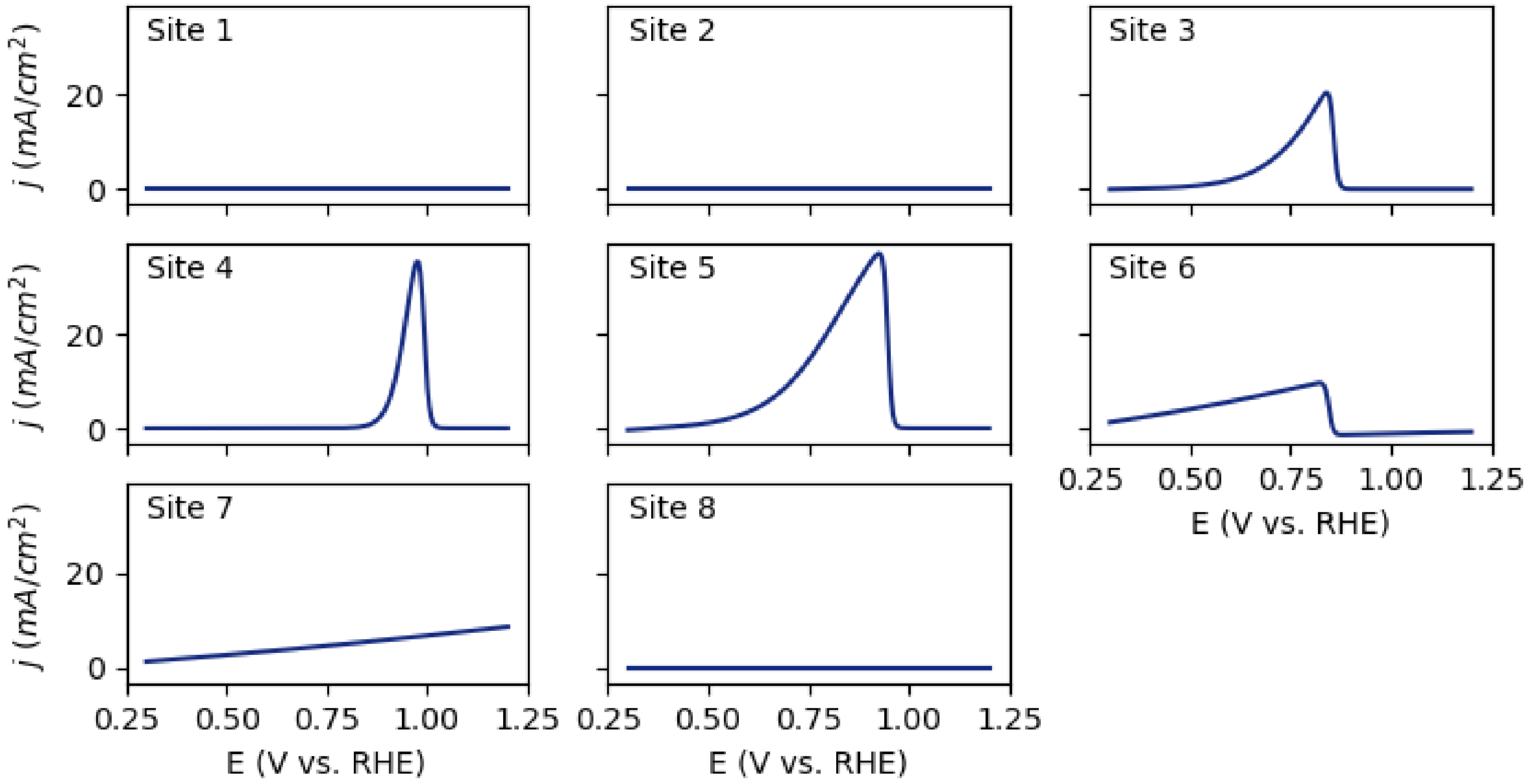

Figure S54. *Visualization of the Butler-Volmer layer for the optimal checkpoint.*

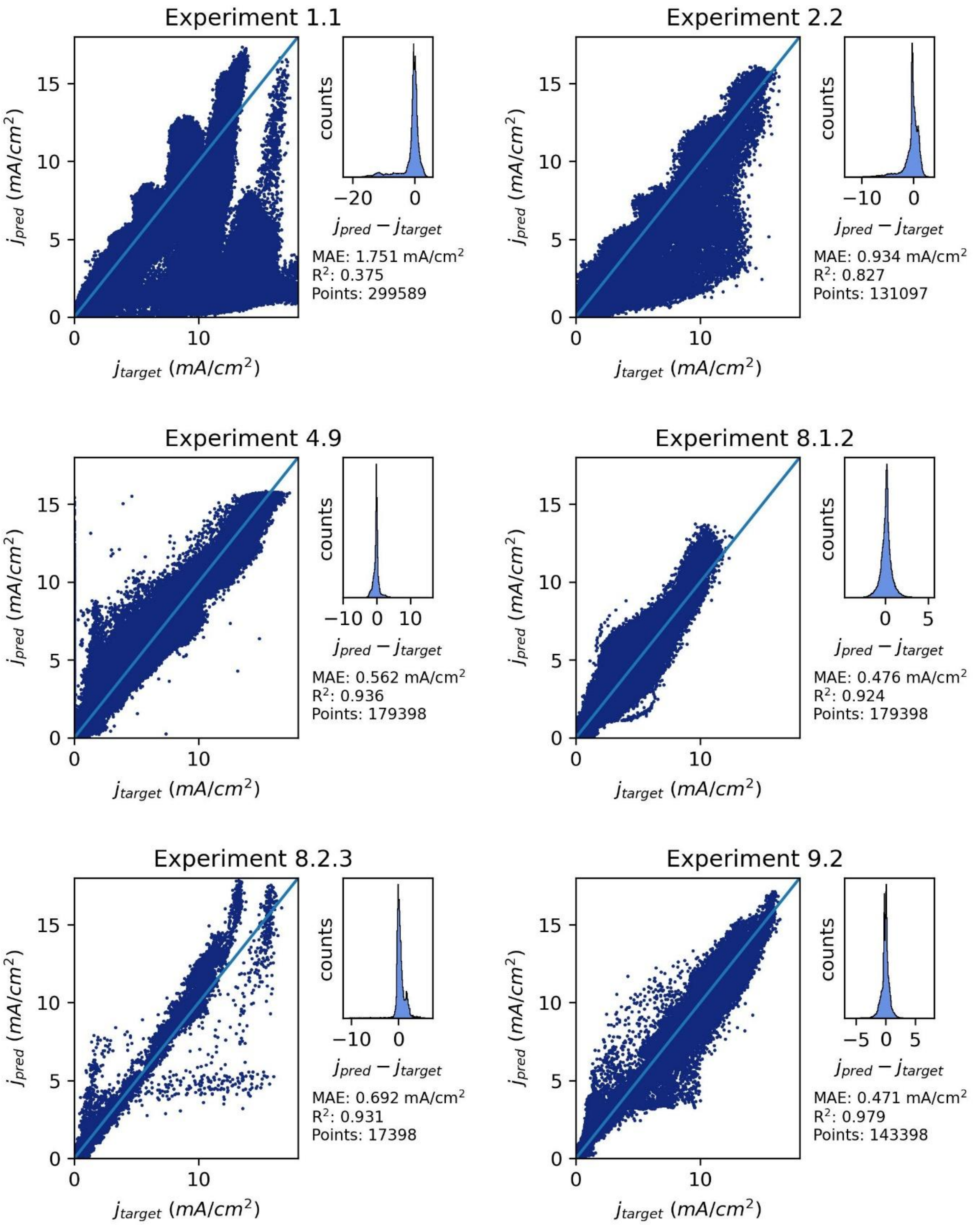

Figure S55. *Parity plots of the test set using a free running simulation with a burn in of 600 steps and the optimal checkpoint.*

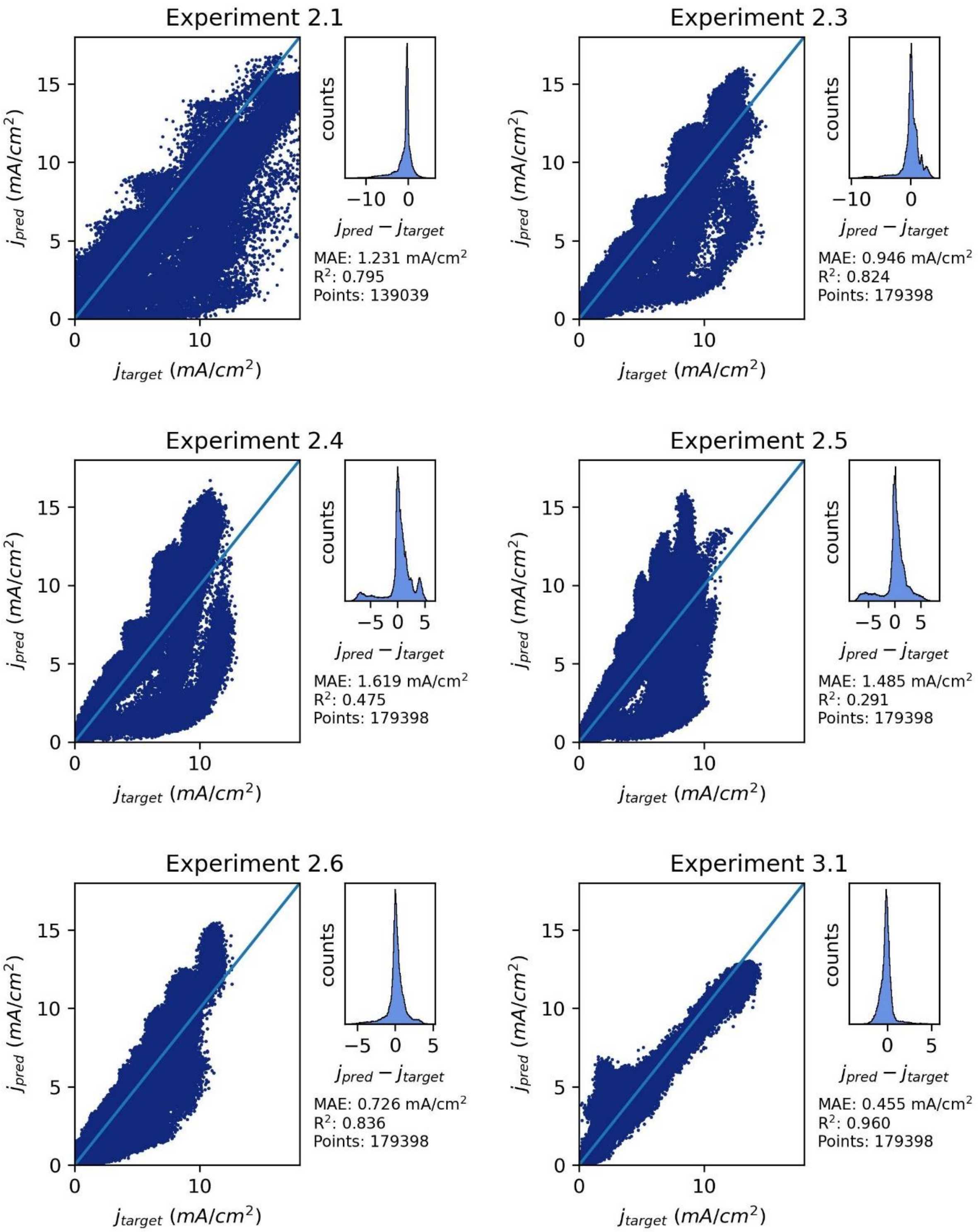
Experiment 2.1
j_{pred} (mA/cm^2)
j_{target} (mA/cm^2)
counts
$j_{pred} - j_{target}$
MAE: 1.231 mA/cm^2
R^2: 0.795
Points: 139039
Experiment 2.3
j_{pred} (mA/cm^2)
j_{target} (mA/cm^2)
counts
$j_{pred} - j_{target}$
MAE: 0.946 mA/cm^2
R^2: 0.824
Points: 179398
Experiment 2.4
j_{pred} (mA/cm^2)
j_{target} (mA/cm^2)
counts
$j_{pred} - j_{target}$
MAE: 1.619 mA/cm^2
R^2: 0.475
Points: 179398
Experiment 2.5
j_{pred} (mA/cm^2)
j_{target} (mA/cm^2)
counts
$j_{pred} - j_{target}$
MAE: 1.485 mA/cm^2
R^2: 0.291
Points: 179398
Experiment 2.6
j_{pred} (mA/cm^2)
j_{target} (mA/cm^2)
counts
$j_{pred} - j_{target}$
MAE: 0.726 mA/cm^2
R^2: 0.836
Points: 179398
Experiment 3.1
j_{pred} (mA/cm^2)
j_{target} (mA/cm^2)
counts
$j_{pred} - j_{target}$
MAE: 0.455 mA/cm^2
R^2: 0.960
Points: 179398

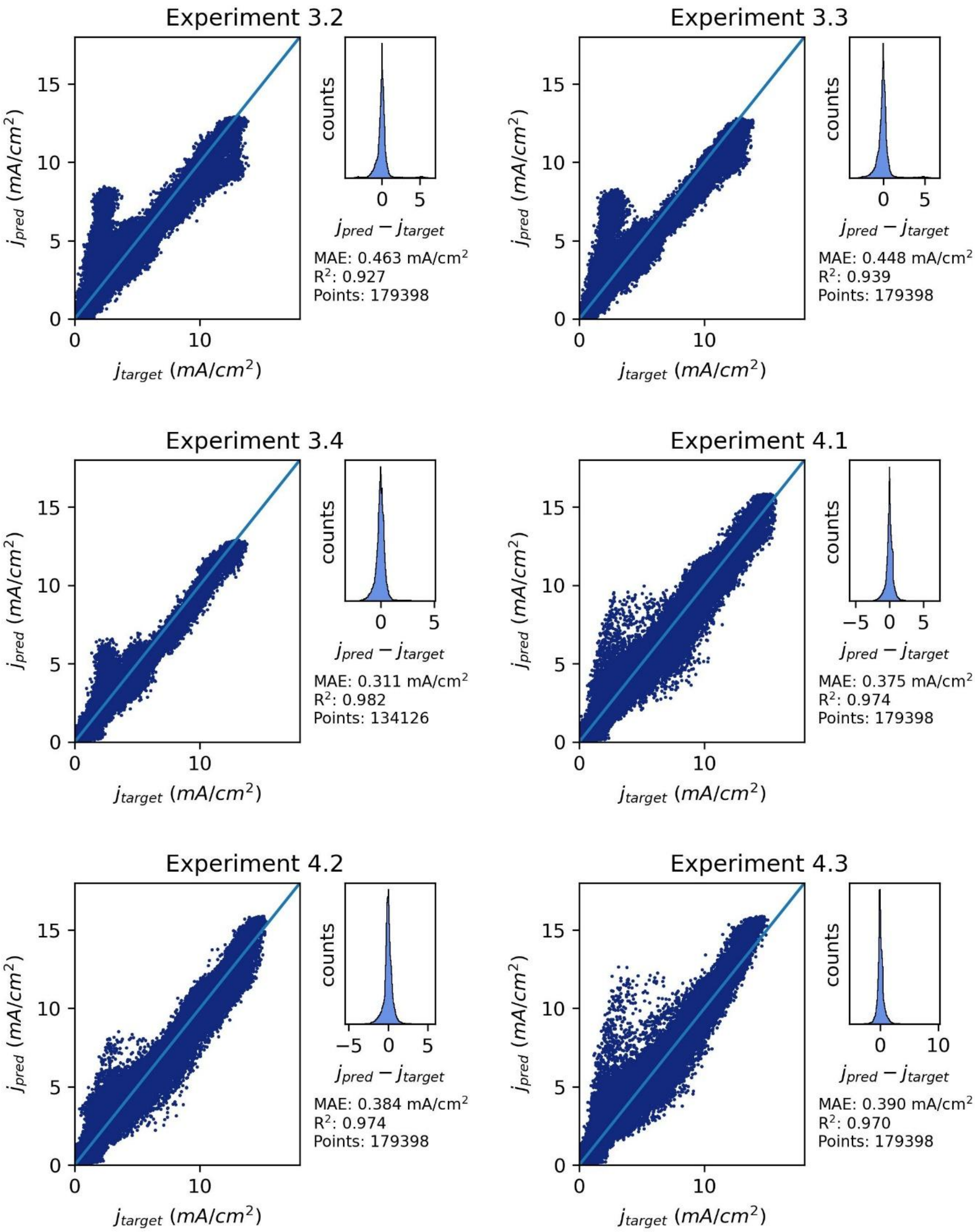

Experiment 3.2
MAE: 0.463 mA/cm^2
R^2: 0.927
Points: 179398
Experiment 3.3
MAE: 0.448 mA/cm^2
R^2: 0.939
Points: 179398
Experiment 3.4
MAE: 0.311 mA/cm^2
R^2: 0.982
Points: 134126
Experiment 4.1
MAE: 0.375 mA/cm^2
R^2: 0.974
Points: 179398
Experiment 4.2
MAE: 0.384 mA/cm^2
R^2: 0.974
Points: 179398
Experiment 4.3
MAE: 0.390 mA/cm^2
R^2: 0.970
Points: 179398
j_{pred} (mA/cm^2)
j_{target} (mA/cm^2)
counts
$j_{pred} - j_{target}$

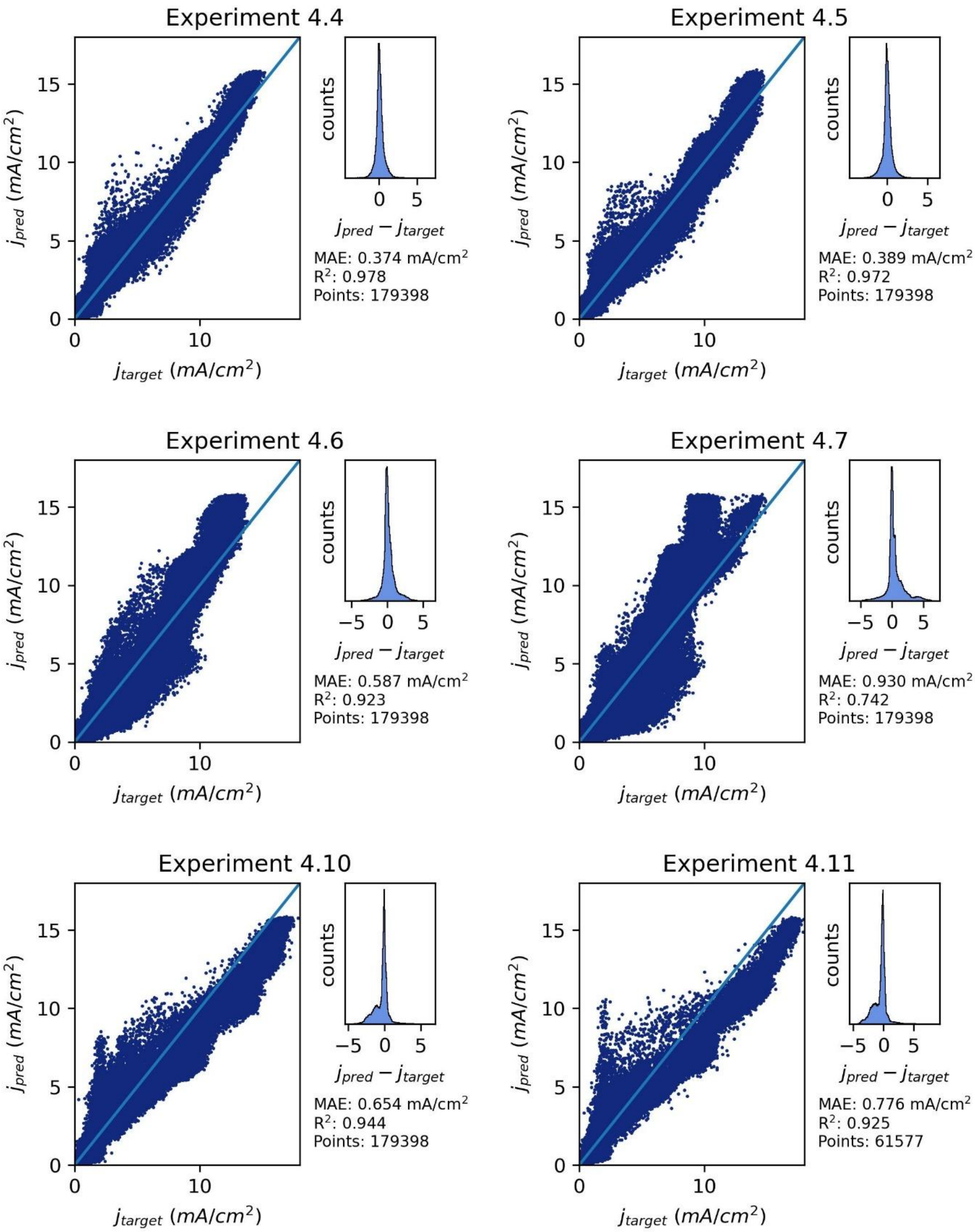

Experiment 4.4
MAE: 0.374 mA/cm2
R2: 0.978
Points: 179398
Experiment 4.5
MAE: 0.389 mA/cm2
R2: 0.972
Points: 179398
Experiment 4.6
MAE: 0.587 mA/cm2
R2: 0.923
Points: 179398
Experiment 4.7
MAE: 0.930 mA/cm2
R2: 0.742
Points: 179398
Experiment 4.10
MAE: 0.654 mA/cm2
R2: 0.944
Points: 179398
Experiment 4.11
MAE: 0.776 mA/cm2
R2: 0.925
Points: 61577
j_pred (mA/cm2)
j_target (mA/cm2)
counts
j_pred − j_target

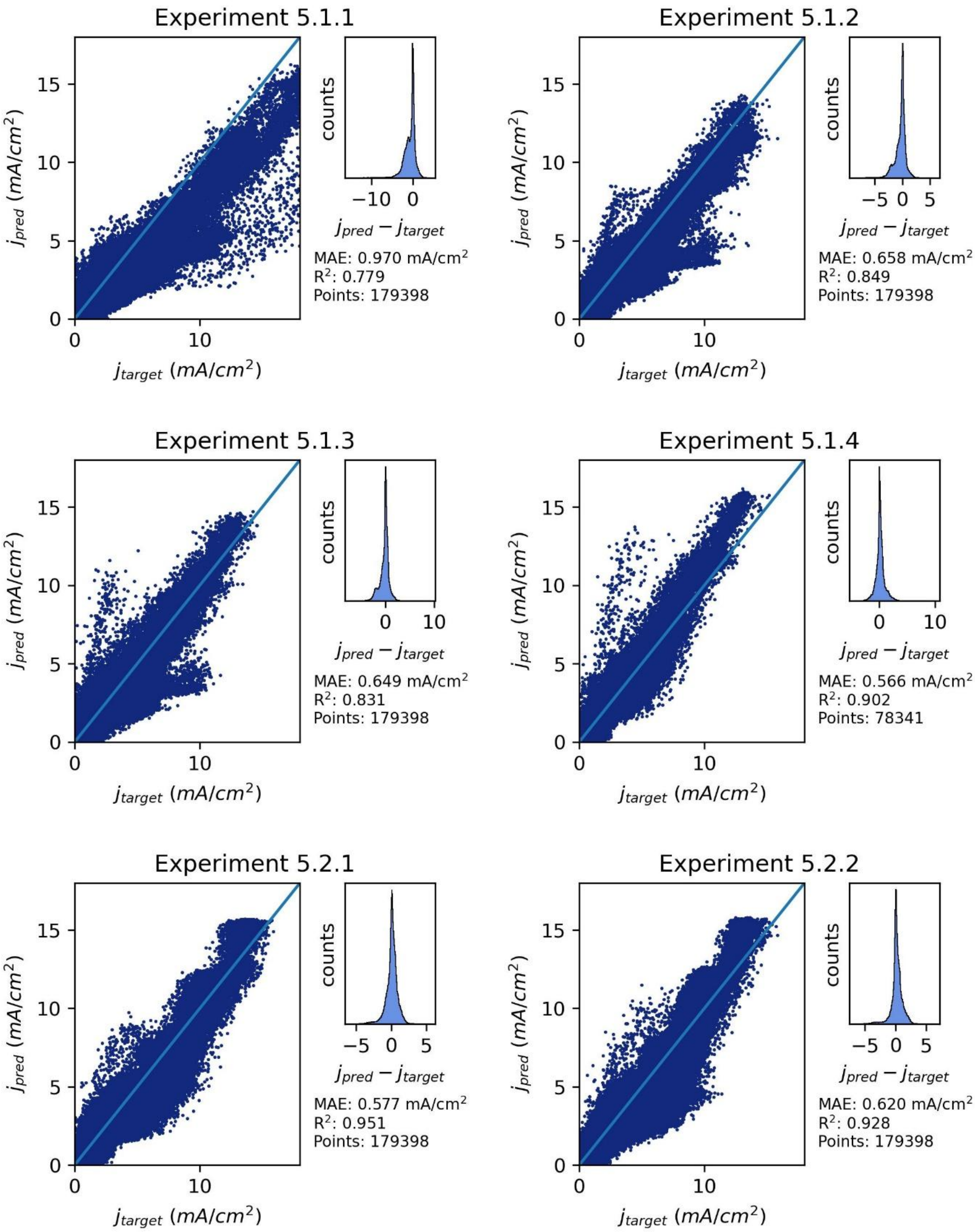
Experiment 5.1.1
MAE: 0.970 mA/cm²
R²: 0.779
Points: 179398
Experiment 5.1.2
MAE: 0.658 mA/cm²
R²: 0.849
Points: 179398
Experiment 5.1.3
MAE: 0.649 mA/cm²
R²: 0.831
Points: 179398
Experiment 5.1.4
MAE: 0.566 mA/cm²
R²: 0.902
Points: 78341
Experiment 5.2.1
MAE: 0.577 mA/cm²
R²: 0.951
Points: 179398
Experiment 5.2.2
MAE: 0.620 mA/cm²
R²: 0.928
Points: 179398
j_pred (mA/cm²)
j_target (mA/cm²)
counts
j_pred − j_target

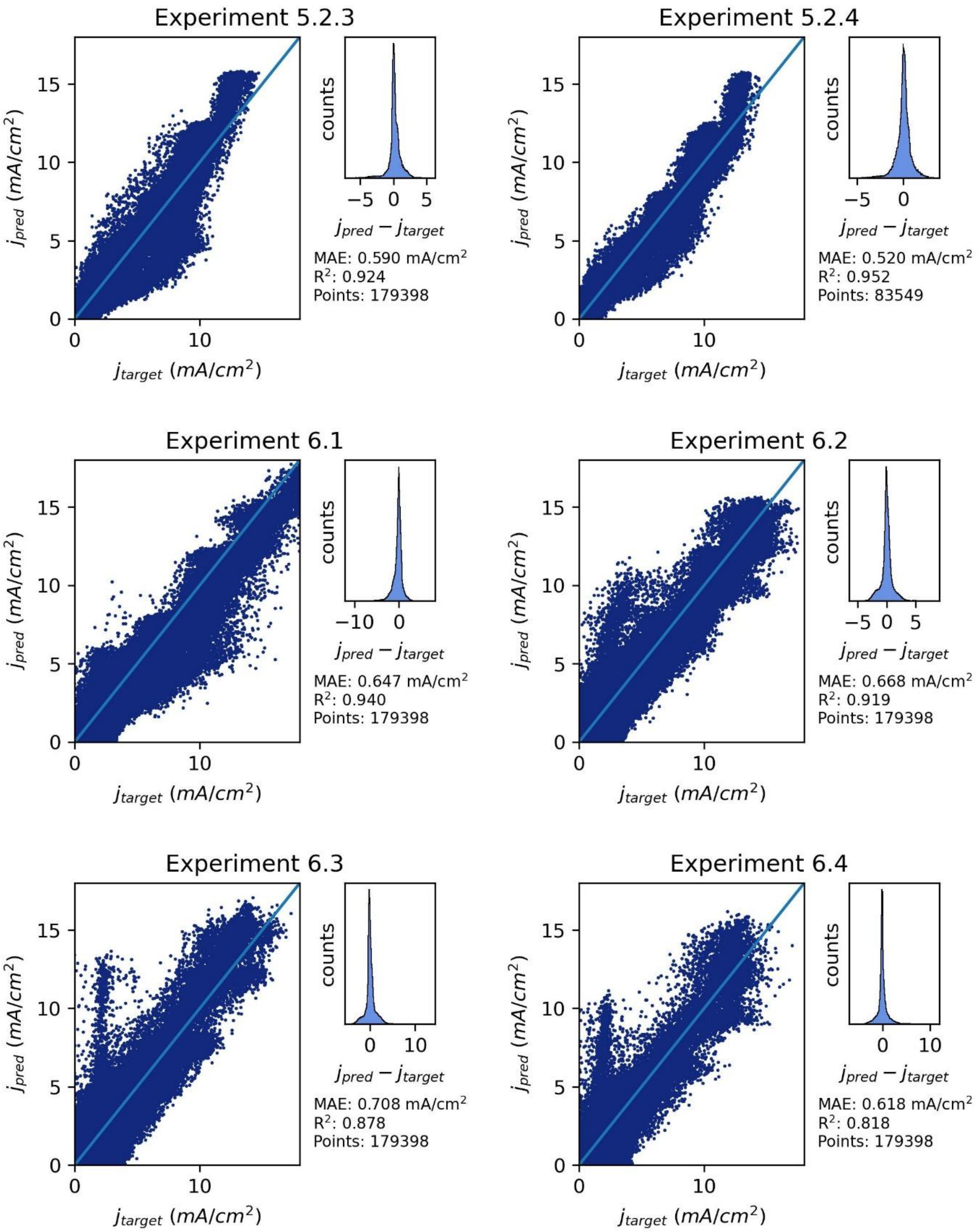
Experiment 5.2.3
j_{pred} (mA/cm^2)
j_{target} (mA/cm^2)
counts
$j_{pred} - j_{target}$
MAE: 0.590 mA/cm^2
R^2: 0.924
Points: 179398
Experiment 5.2.4
j_{pred} (mA/cm^2)
j_{target} (mA/cm^2)
counts
$j_{pred} - j_{target}$
MAE: 0.520 mA/cm^2
R^2: 0.952
Points: 83549
Experiment 6.1
j_{pred} (mA/cm^2)
j_{target} (mA/cm^2)
counts
$j_{pred} - j_{target}$
MAE: 0.647 mA/cm^2
R^2: 0.940
Points: 179398
Experiment 6.2
j_{pred} (mA/cm^2)
j_{target} (mA/cm^2)
counts
$j_{pred} - j_{target}$
MAE: 0.668 mA/cm^2
R^2: 0.919
Points: 179398
Experiment 6.3
j_{pred} (mA/cm^2)
j_{target} (mA/cm^2)
counts
$j_{pred} - j_{target}$
MAE: 0.708 mA/cm^2
R^2: 0.878
Points: 179398
Experiment 6.4
j_{pred} (mA/cm^2)
j_{target} (mA/cm^2)
counts
$j_{pred} - j_{target}$
MAE: 0.618 mA/cm^2
R^2: 0.818
Points: 179398

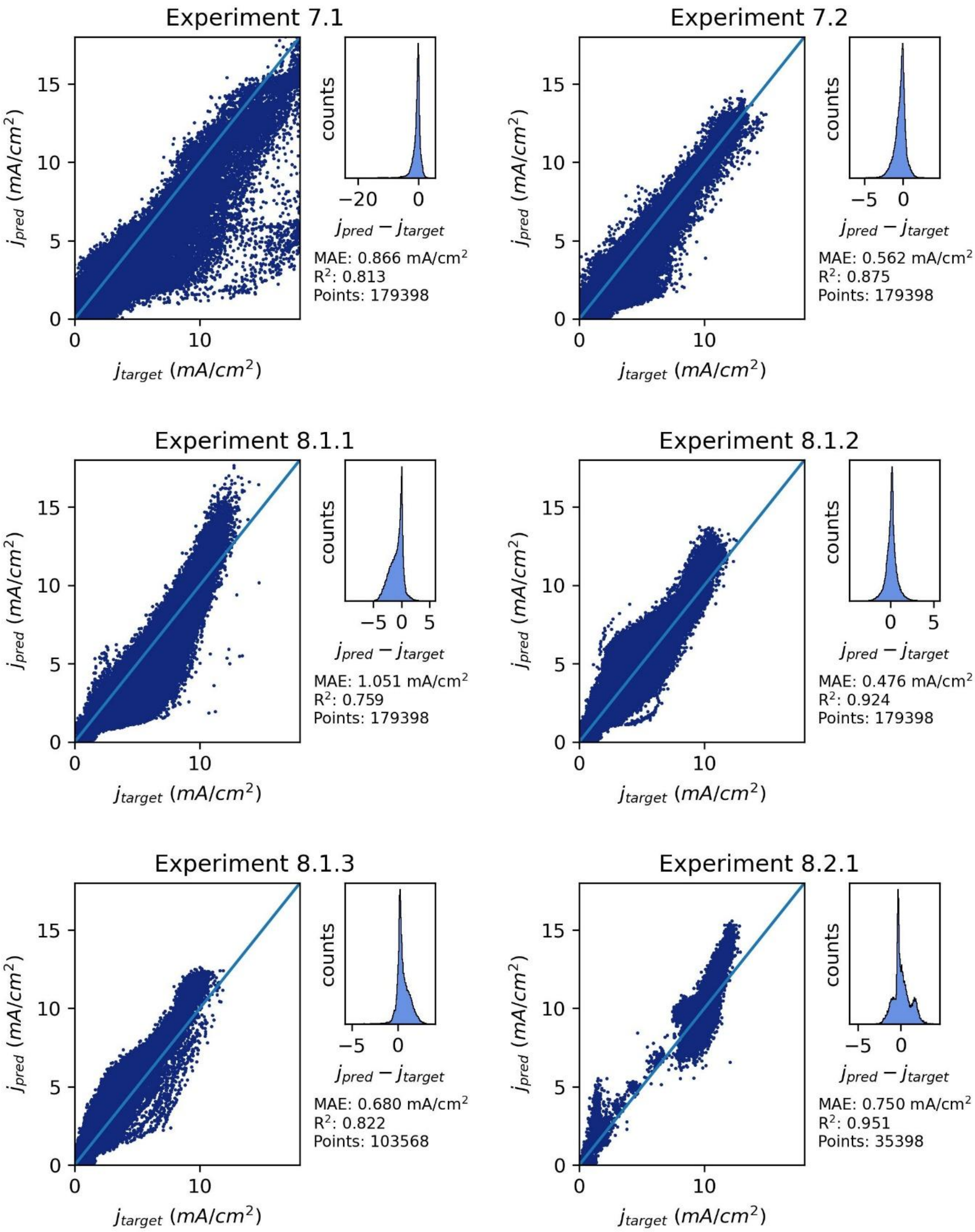

Experiment 7.1
MAE: 0.866 mA/cm2
R2: 0.813
Points: 179398
Experiment 7.2
MAE: 0.562 mA/cm2
R2: 0.875
Points: 179398
Experiment 8.1.1
MAE: 1.051 mA/cm2
R2: 0.759
Points: 179398
Experiment 8.1.2
MAE: 0.476 mA/cm2
R2: 0.924
Points: 179398
Experiment 8.1.3
MAE: 0.680 mA/cm2
R2: 0.822
Points: 103568
Experiment 8.2.1
MAE: 0.750 mA/cm2
R2: 0.951
Points: 35398
j_pred (mA/cm2)
j_target (mA/cm2)
counts
j_pred − j_target

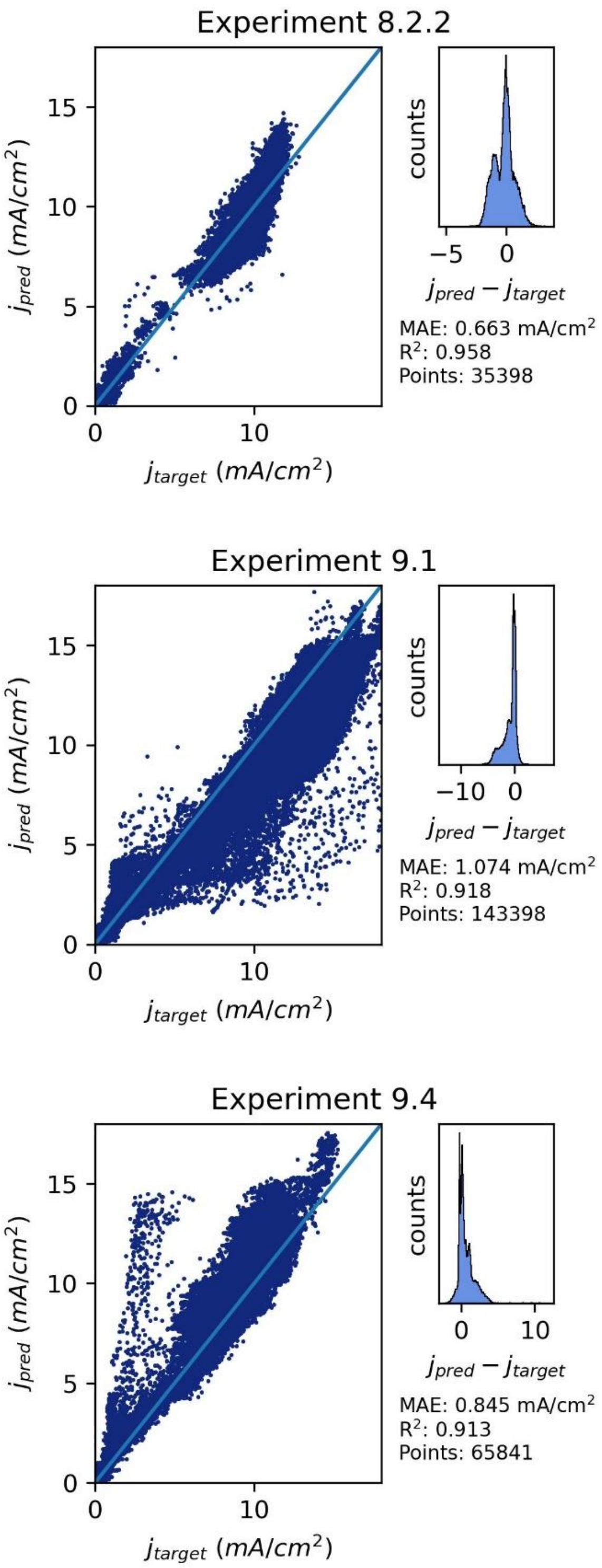

Figure S56. *Parity plots of the training set using a free running simulation with a burn in of 600 steps and the optimal checkpoint.*

VI.4 AI Model 2.2

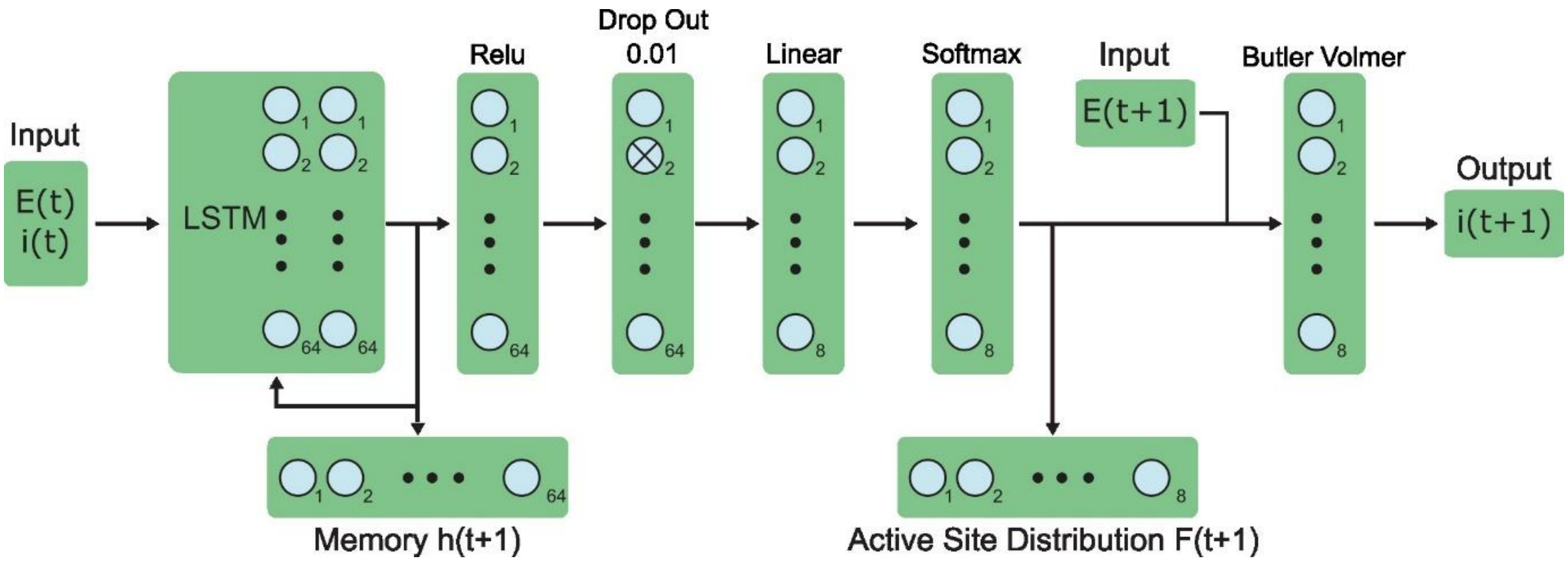

Figure S57. *The structure of AI Model 2.2. The output layer is a sum of all incoming units.*

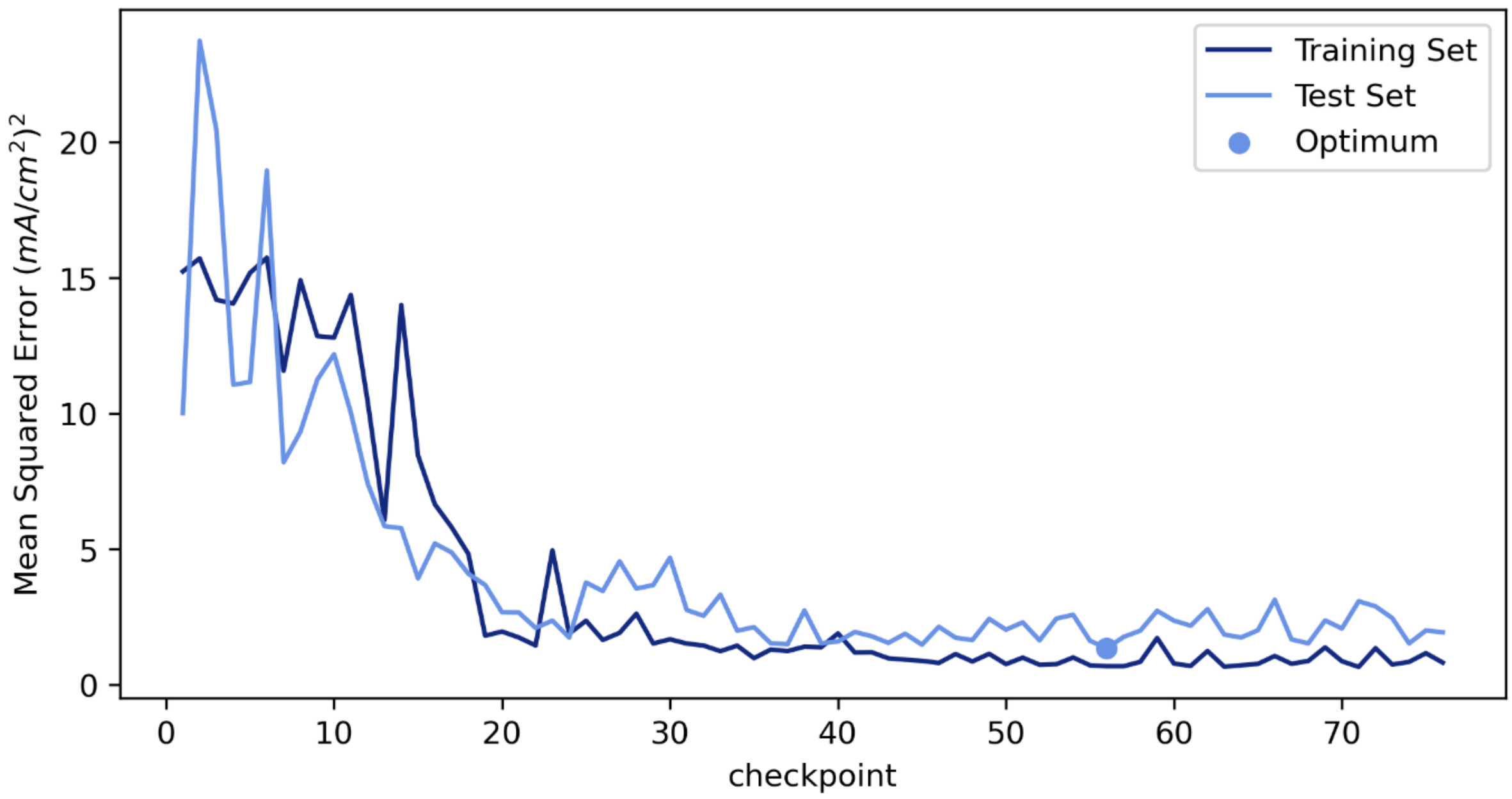

Figure S58. *Cross-validation scores vs. checkpoint number. The training and test datasets were segmented into chunks of 30 000 observations, and any remaining tails containing fewer than 30 000 observations were discarded. For each segment, the first 600 points were used to burn in the hidden state, while the remaining 29 400 points were used to evaluate the checkpoint performance.*

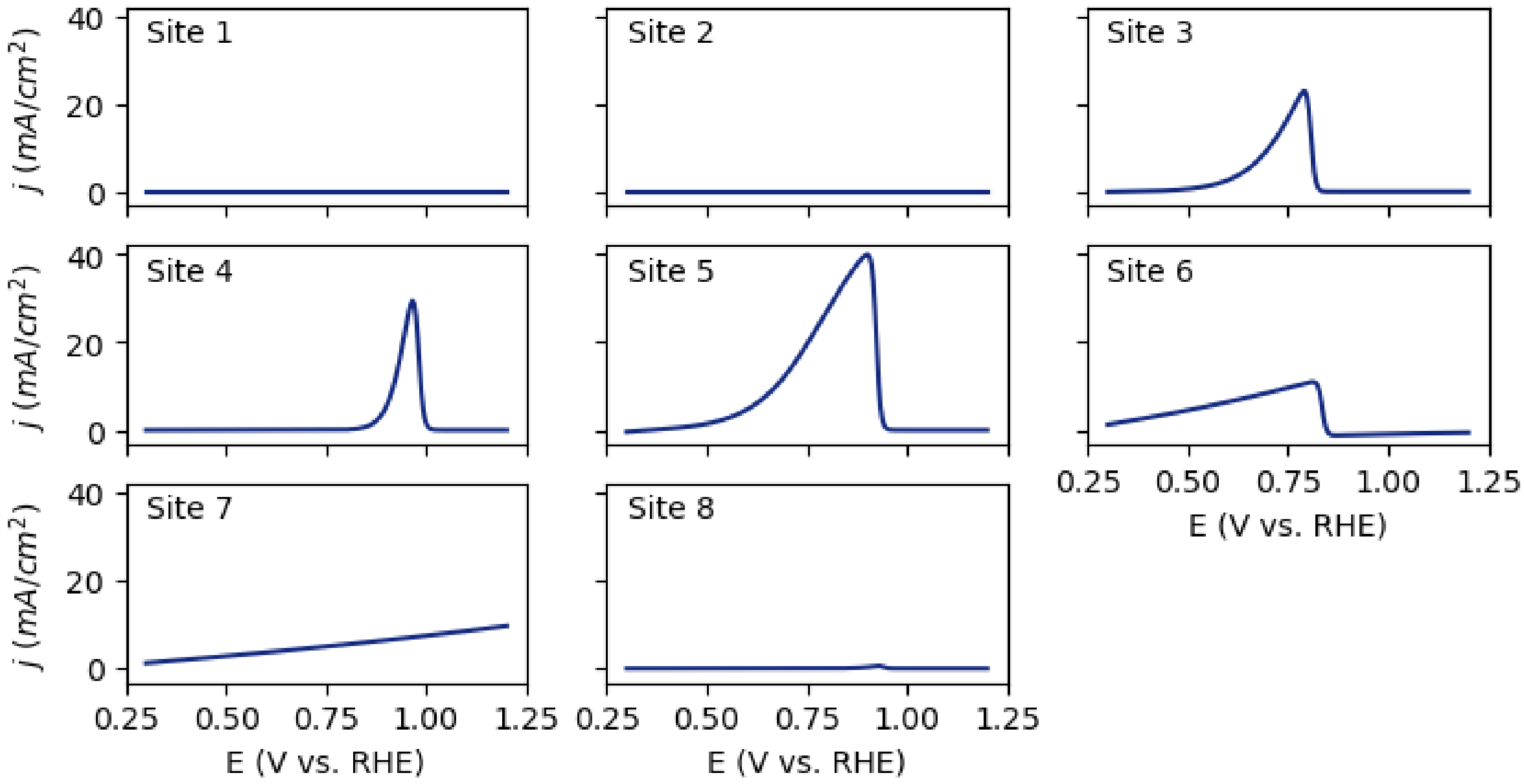

Figure S59. *Visualization of the Butler-Volmer layer for the optimal checkpoint. The curves are plotted assuming that the active site has the maximum coverage of 1.*

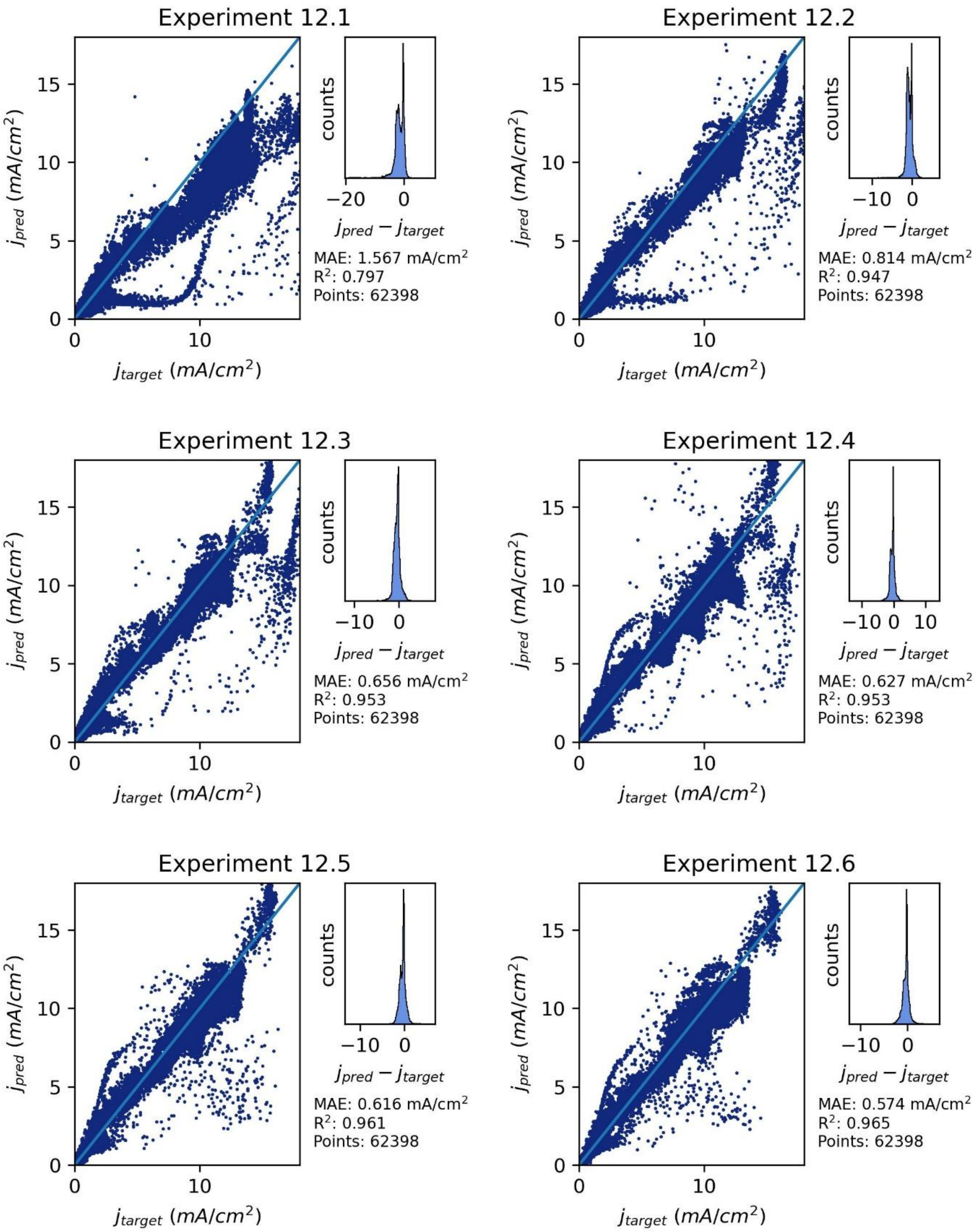
Experiment 12.1
MAE: 1.567 mA/cm2
R2: 0.797
Points: 62398
Experiment 12.2
MAE: 0.814 mA/cm2
R2: 0.947
Points: 62398
Experiment 12.3
MAE: 0.656 mA/cm2
R2: 0.953
Points: 62398
Experiment 12.4
MAE: 0.627 mA/cm2
R2: 0.953
Points: 62398
Experiment 12.5
MAE: 0.616 mA/cm2
R2: 0.961
Points: 62398
Experiment 12.6
MAE: 0.574 mA/cm2
R2: 0.965
Points: 62398
j_pred (mA/cm2)
j_target (mA/cm2)
counts
j_pred − j_target

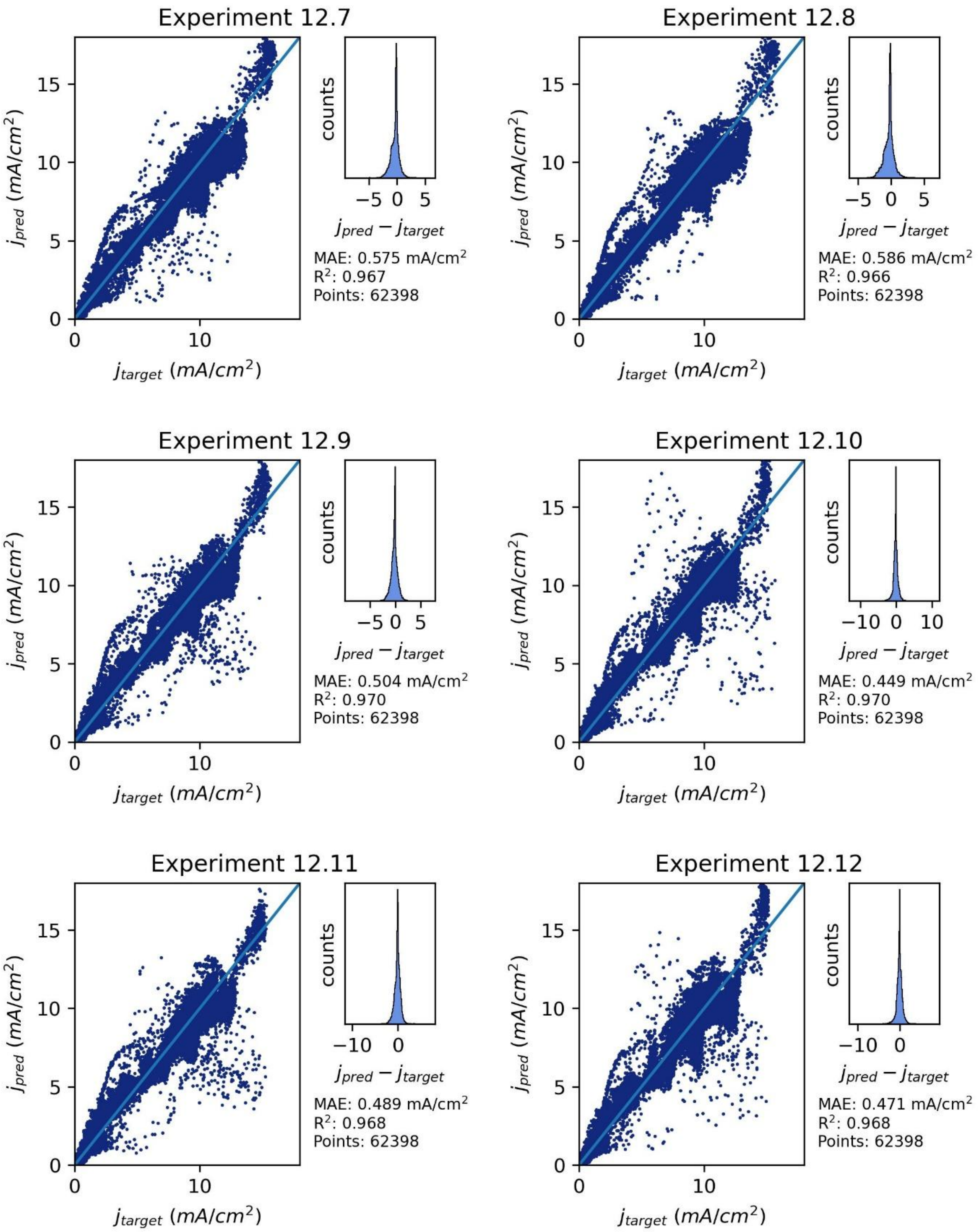
Experiment 12.7
j_{pred} (mA/cm^2)
j_{target} (mA/cm^2)
counts
$j_{pred} - j_{target}$
MAE: 0.575 mA/cm^2
R^2: 0.967
Points: 62398
Experiment 12.8
j_{pred} (mA/cm^2)
j_{target} (mA/cm^2)
counts
$j_{pred} - j_{target}$
MAE: 0.586 mA/cm^2
R^2: 0.966
Points: 62398
Experiment 12.9
j_{pred} (mA/cm^2)
j_{target} (mA/cm^2)
counts
$j_{pred} - j_{target}$
MAE: 0.504 mA/cm^2
R^2: 0.970
Points: 62398
Experiment 12.10
j_{pred} (mA/cm^2)
j_{target} (mA/cm^2)
counts
$j_{pred} - j_{target}$
MAE: 0.449 mA/cm^2
R^2: 0.970
Points: 62398
Experiment 12.11
j_{pred} (mA/cm^2)
j_{target} (mA/cm^2)
counts
$j_{pred} - j_{target}$
MAE: 0.489 mA/cm^2
R^2: 0.968
Points: 62398
Experiment 12.12
j_{pred} (mA/cm^2)
j_{target} (mA/cm^2)
counts
$j_{pred} - j_{target}$
MAE: 0.471 mA/cm^2
R^2: 0.968
Points: 62398

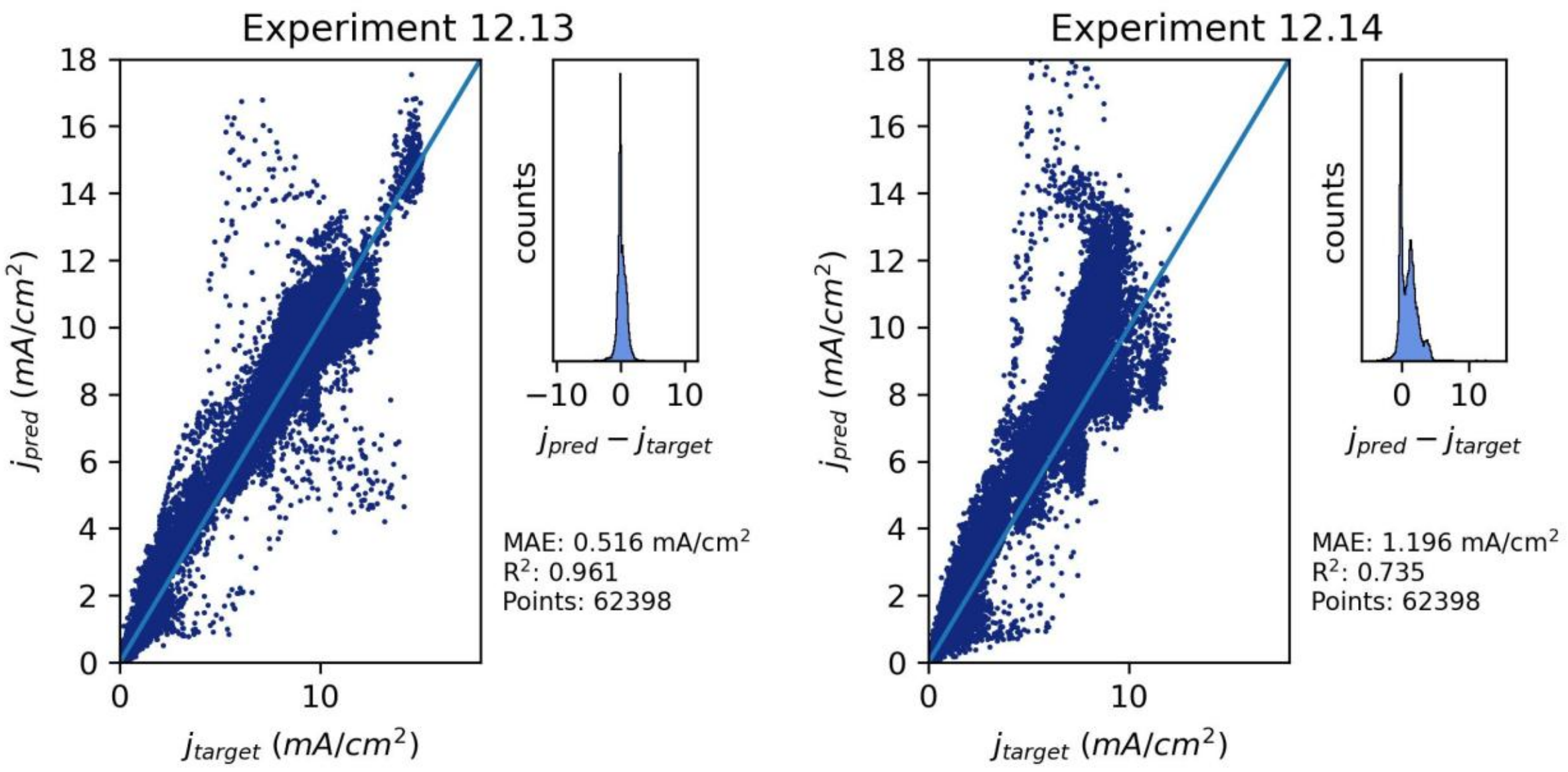

Figure S60. *Parity plots of the test set using a free running simulation with a burn in of 600 steps and the optimal checkpoint.*

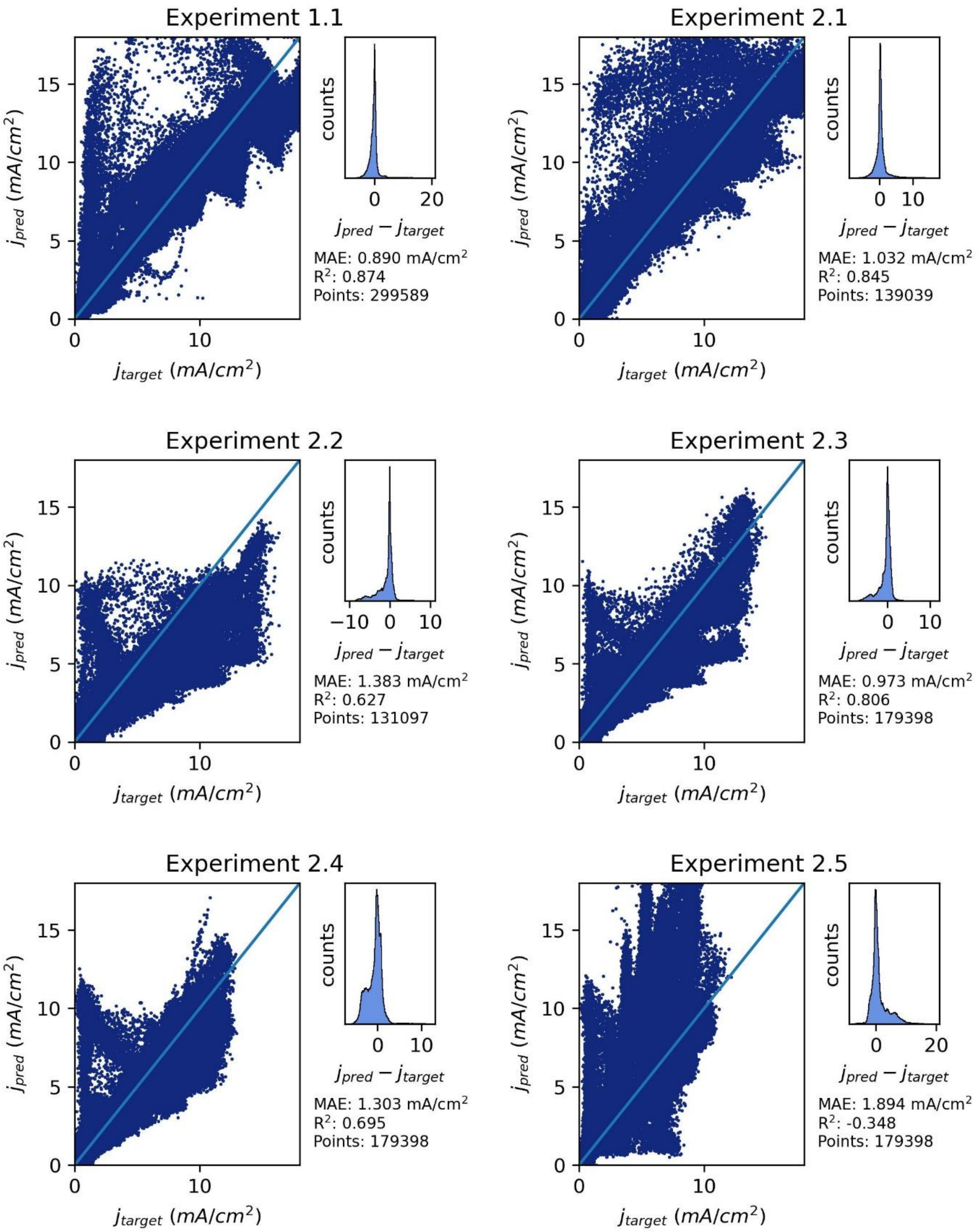
Experiment 1.1
MAE: 0.890 mA/cm^2
R^2: 0.874
Points: 299589
Experiment 2.1
MAE: 1.032 mA/cm^2
R^2: 0.845
Points: 139039
Experiment 2.2
MAE: 1.383 mA/cm^2
R^2: 0.627
Points: 131097
Experiment 2.3
MAE: 0.973 mA/cm^2
R^2: 0.806
Points: 179398
Experiment 2.4
MAE: 1.303 mA/cm^2
R^2: 0.695
Points: 179398
Experiment 2.5
MAE: 1.894 mA/cm^2
R^2: -0.348
Points: 179398
j_{pred} (mA/cm^2)
j_{target} (mA/cm^2)
counts
$j_{pred} - j_{target}$

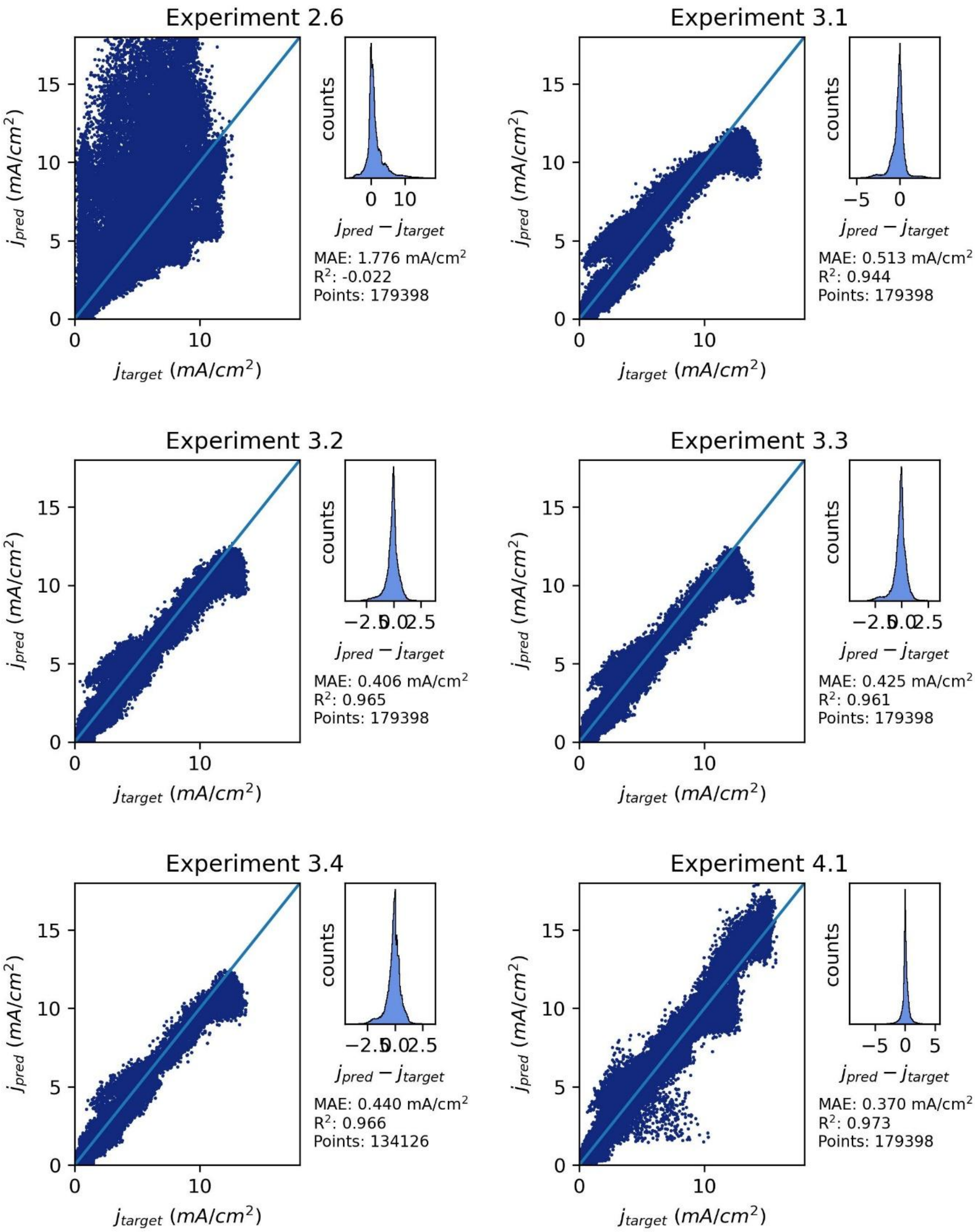

Experiment 2.6
MAE: 1.776 mA/cm^2
R^2: -0.022
Points: 179398
Experiment 3.1
MAE: 0.513 mA/cm^2
R^2: 0.944
Points: 179398
Experiment 3.2
MAE: 0.406 mA/cm^2
R^2: 0.965
Points: 179398
Experiment 3.3
MAE: 0.425 mA/cm^2
R^2: 0.961
Points: 179398
Experiment 3.4
MAE: 0.440 mA/cm^2
R^2: 0.966
Points: 134126
Experiment 4.1
MAE: 0.370 mA/cm^2
R^2: 0.973
Points: 179398
j_{pred} (mA/cm^2)
j_{target} (mA/cm^2)
counts
$j_{pred} - j_{target}$

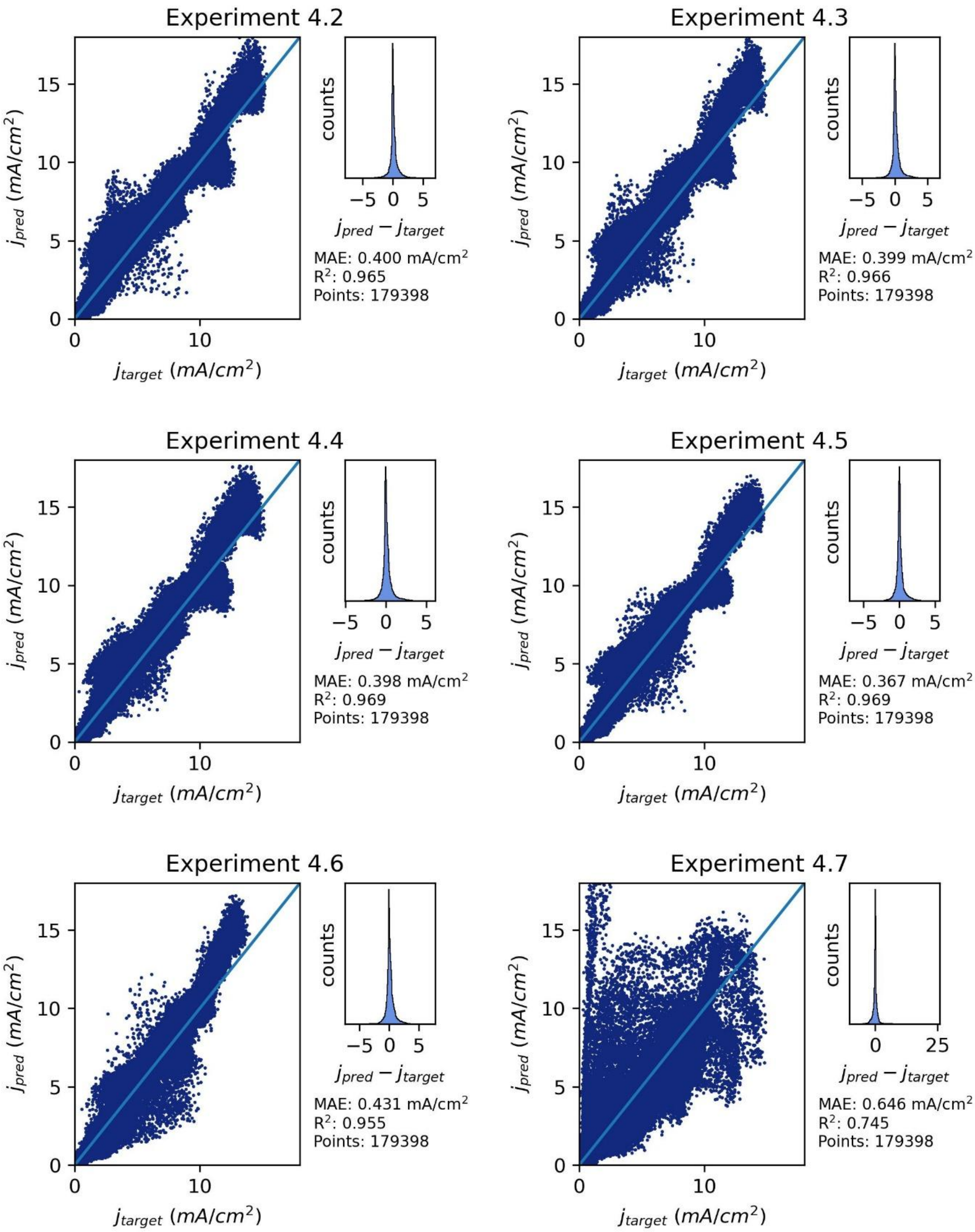
Experiment 4.2
Experiment 4.3
Experiment 4.4
Experiment 4.5
Experiment 4.6
Experiment 4.7
j_{pred} (mA/cm^2)
j_{target} (mA/cm^2)
counts
$j_{pred} - j_{target}$
MAE: 0.400 mA/cm^2
R^2: 0.965
Points: 179398
MAE: 0.399 mA/cm^2
R^2: 0.966
Points: 179398
MAE: 0.398 mA/cm^2
R^2: 0.969
Points: 179398
MAE: 0.367 mA/cm^2
R^2: 0.969
Points: 179398
MAE: 0.431 mA/cm^2
R^2: 0.955
Points: 179398
MAE: 0.646 mA/cm^2
R^2: 0.745
Points: 179398

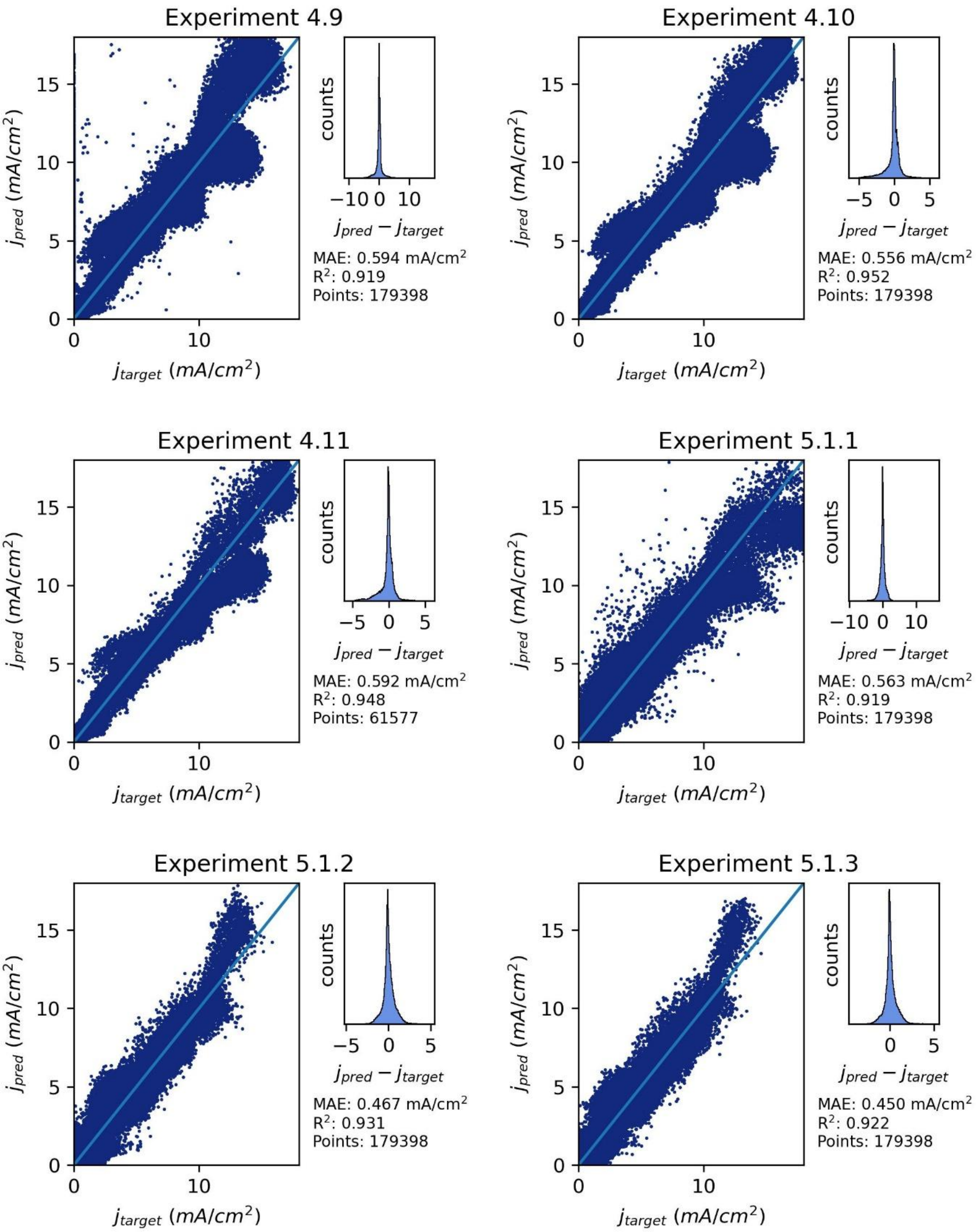
Experiment 4.9
j_{pred} (mA/cm^2)
j_{target} (mA/cm^2)
counts
$j_{pred} - j_{target}$
MAE: 0.594 mA/cm^2
R^2: 0.919
Points: 179398
Experiment 4.10
j_{pred} (mA/cm^2)
j_{target} (mA/cm^2)
counts
$j_{pred} - j_{target}$
MAE: 0.556 mA/cm^2
R^2: 0.952
Points: 179398
Experiment 4.11
j_{pred} (mA/cm^2)
j_{target} (mA/cm^2)
counts
$j_{pred} - j_{target}$
MAE: 0.592 mA/cm^2
R^2: 0.948
Points: 61577
Experiment 5.1.1
j_{pred} (mA/cm^2)
j_{target} (mA/cm^2)
counts
$j_{pred} - j_{target}$
MAE: 0.563 mA/cm^2
R^2: 0.919
Points: 179398
Experiment 5.1.2
j_{pred} (mA/cm^2)
j_{target} (mA/cm^2)
counts
$j_{pred} - j_{target}$
MAE: 0.467 mA/cm^2
R^2: 0.931
Points: 179398
Experiment 5.1.3
j_{pred} (mA/cm^2)
j_{target} (mA/cm^2)
counts
$j_{pred} - j_{target}$
MAE: 0.450 mA/cm^2
R^2: 0.922
Points: 179398

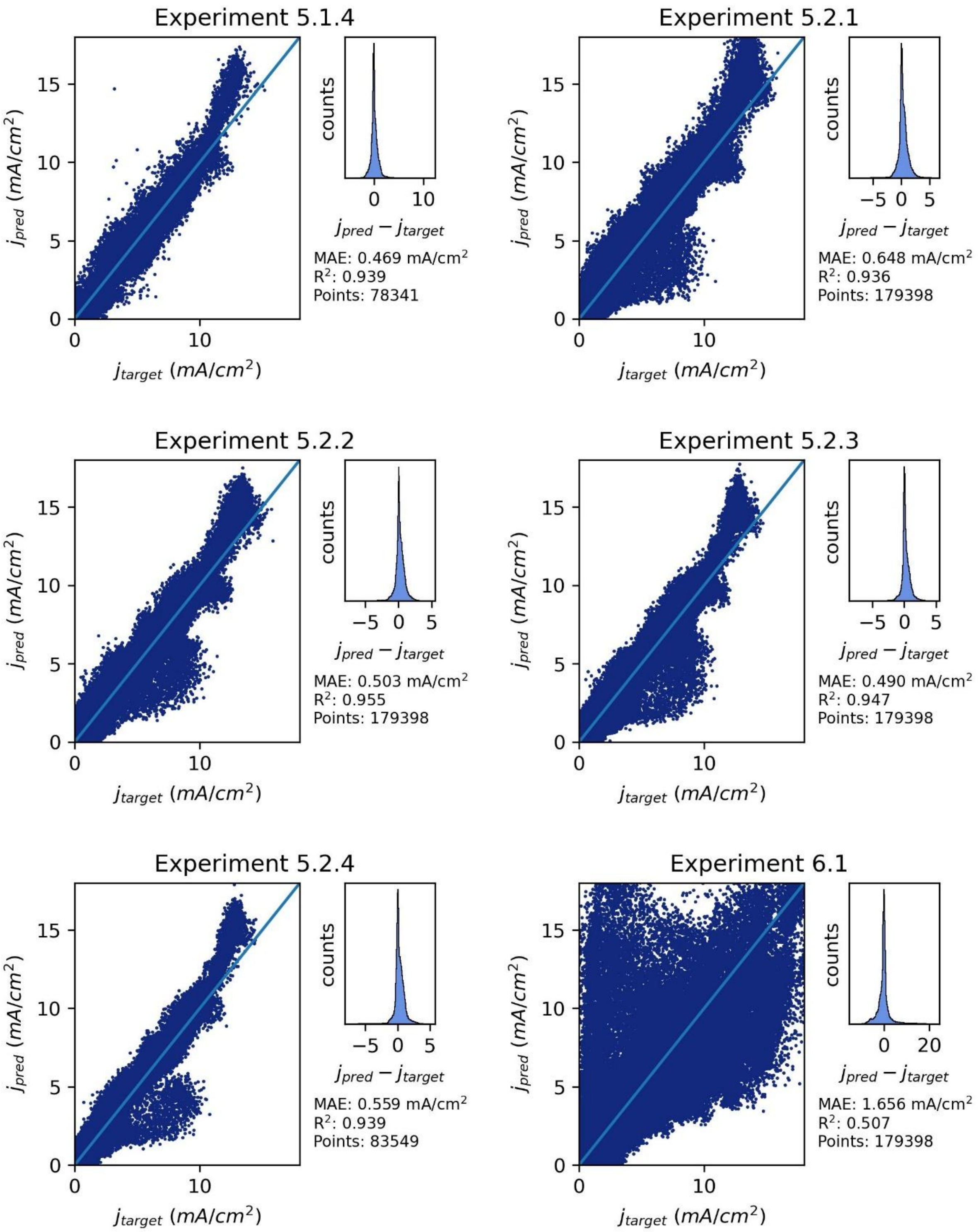

Experiment 5.1.4
j_{pred} (mA/cm^2)
j_{target} (mA/cm^2)
counts
$j_{pred} - j_{target}$
MAE: 0.469 mA/cm^2
R^2: 0.939
Points: 78341
Experiment 5.2.1
MAE: 0.648 mA/cm^2
R^2: 0.936
Points: 179398
Experiment 5.2.2
MAE: 0.503 mA/cm^2
R^2: 0.955
Points: 179398
Experiment 5.2.3
MAE: 0.490 mA/cm^2
R^2: 0.947
Points: 179398
Experiment 5.2.4
MAE: 0.559 mA/cm^2
R^2: 0.939
Points: 83549
Experiment 6.1
MAE: 1.656 mA/cm^2
R^2: 0.507
Points: 179398

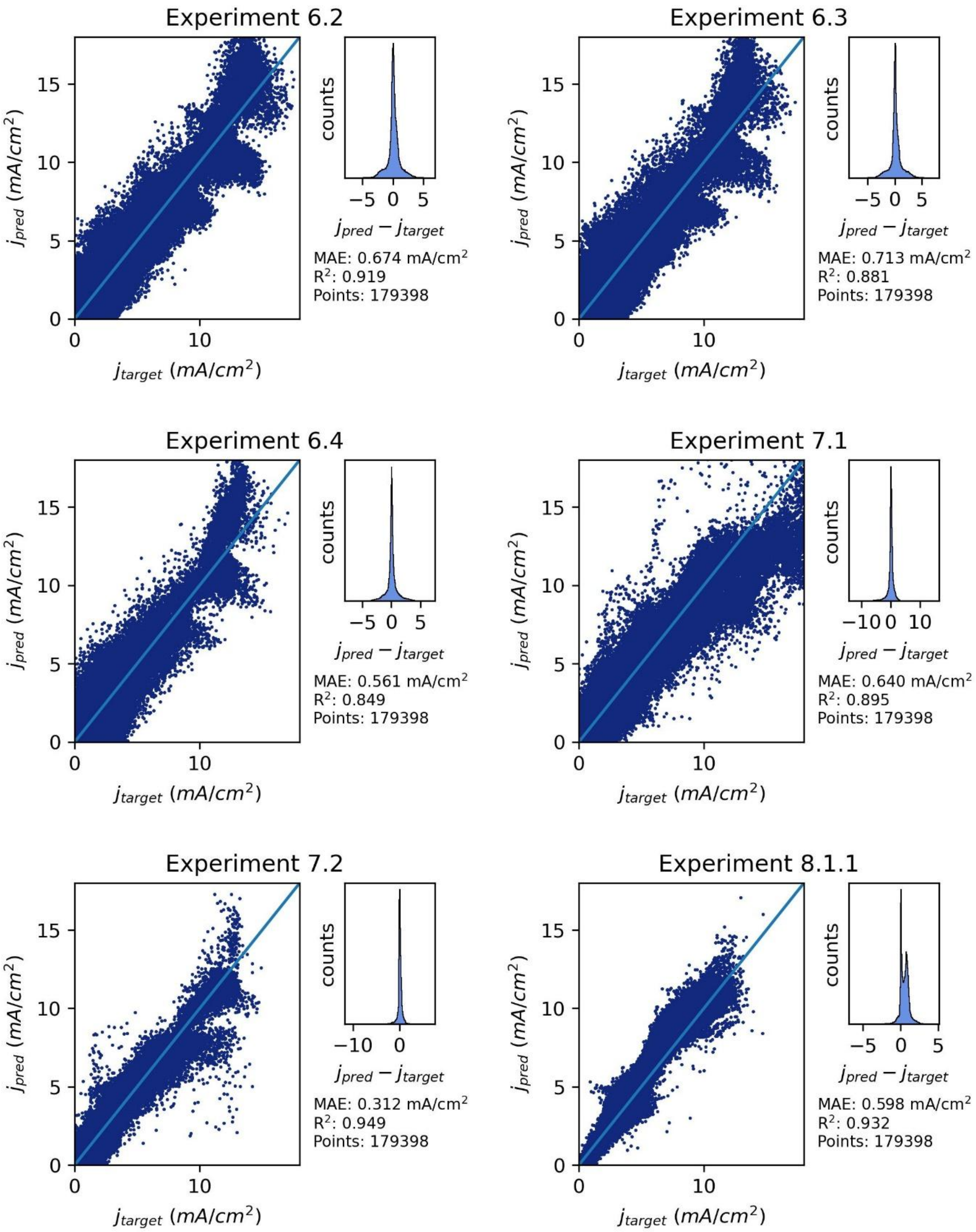
Experiment 6.2
MAE: 0.674 mA/cm2
R2: 0.919
Points: 179398
Experiment 6.3
MAE: 0.713 mA/cm2
R2: 0.881
Points: 179398
Experiment 6.4
MAE: 0.561 mA/cm2
R2: 0.849
Points: 179398
Experiment 7.1
MAE: 0.640 mA/cm2
R2: 0.895
Points: 179398
Experiment 7.2
MAE: 0.312 mA/cm2
R2: 0.949
Points: 179398
Experiment 8.1.1
MAE: 0.598 mA/cm2
R2: 0.932
Points: 179398
j_pred (mA/cm2)
j_target (mA/cm2)
counts
j_pred − j_target

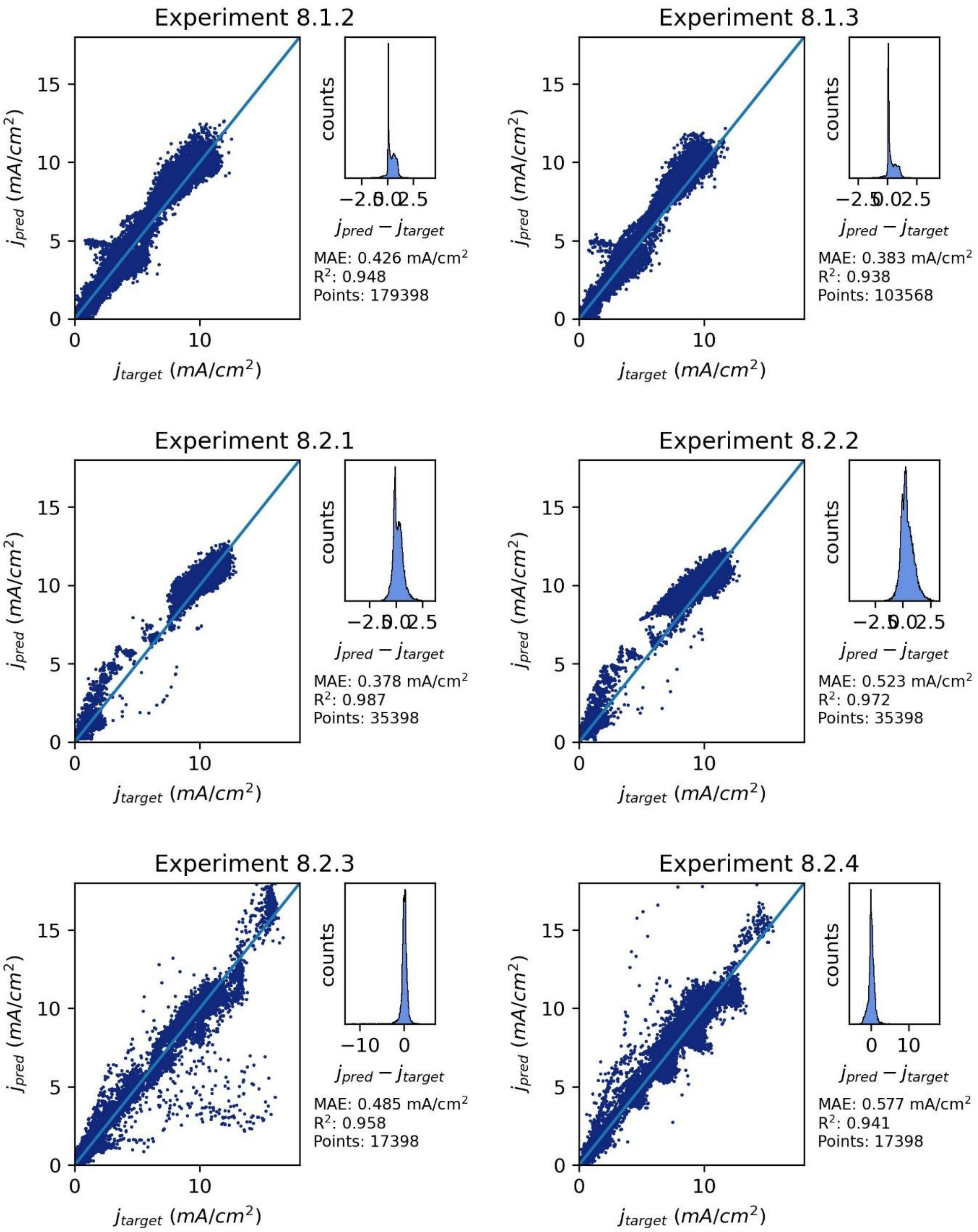
Experiment 8.1.2
j_{pred} (mA/cm^2)
j_{target} (mA/cm^2)
counts
$j_{pred} - j_{target}$
MAE: 0.426 mA/cm^2
R^2: 0.948
Points: 179398
Experiment 8.1.3
j_{pred} (mA/cm^2)
j_{target} (mA/cm^2)
counts
$j_{pred} - j_{target}$
MAE: 0.383 mA/cm^2
R^2: 0.938
Points: 103568
Experiment 8.2.1
j_{pred} (mA/cm^2)
j_{target} (mA/cm^2)
counts
$j_{pred} - j_{target}$
MAE: 0.378 mA/cm^2
R^2: 0.987
Points: 35398
Experiment 8.2.2
j_{pred} (mA/cm^2)
j_{target} (mA/cm^2)
counts
$j_{pred} - j_{target}$
MAE: 0.523 mA/cm^2
R^2: 0.972
Points: 35398
Experiment 8.2.3
j_{pred} (mA/cm^2)
j_{target} (mA/cm^2)
counts
$j_{pred} - j_{target}$
MAE: 0.485 mA/cm^2
R^2: 0.958
Points: 17398
Experiment 8.2.4
j_{pred} (mA/cm^2)
j_{target} (mA/cm^2)
counts
$j_{pred} - j_{target}$
MAE: 0.577 mA/cm^2
R^2: 0.941
Points: 17398

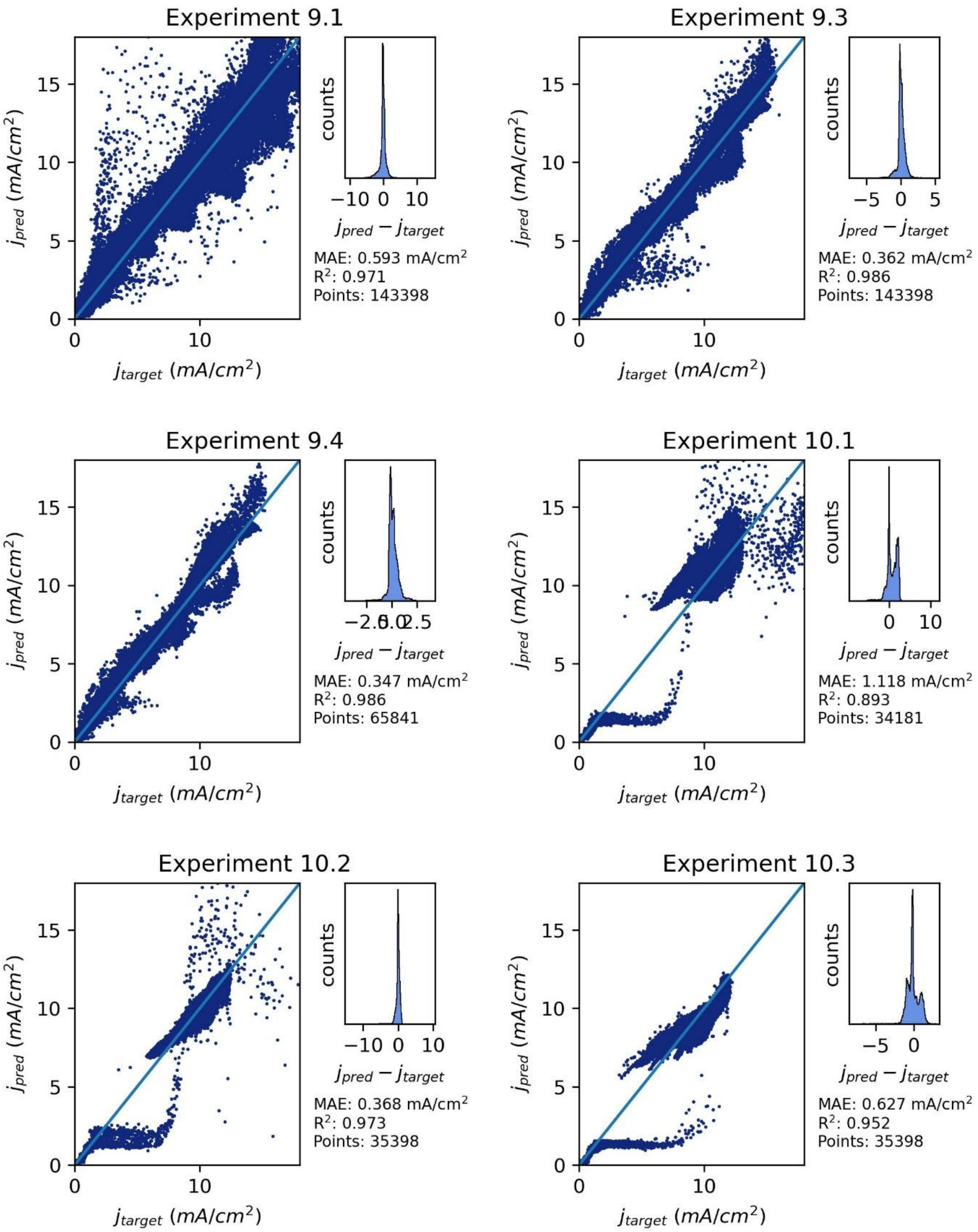
Experiment 9.1
MAE: 0.593 mA/cm2
R2: 0.971
Points: 143398
Experiment 9.3
MAE: 0.362 mA/cm2
R2: 0.986
Points: 143398
Experiment 9.4
MAE: 0.347 mA/cm2
R2: 0.986
Points: 65841
Experiment 10.1
MAE: 1.118 mA/cm2
R2: 0.893
Points: 34181
Experiment 10.2
MAE: 0.368 mA/cm2
R2: 0.973
Points: 35398
Experiment 10.3
MAE: 0.627 mA/cm2
R2: 0.952
Points: 35398
jpred (mA/cm2)
jtarget (mA/cm2)
counts
jpred − jtarget

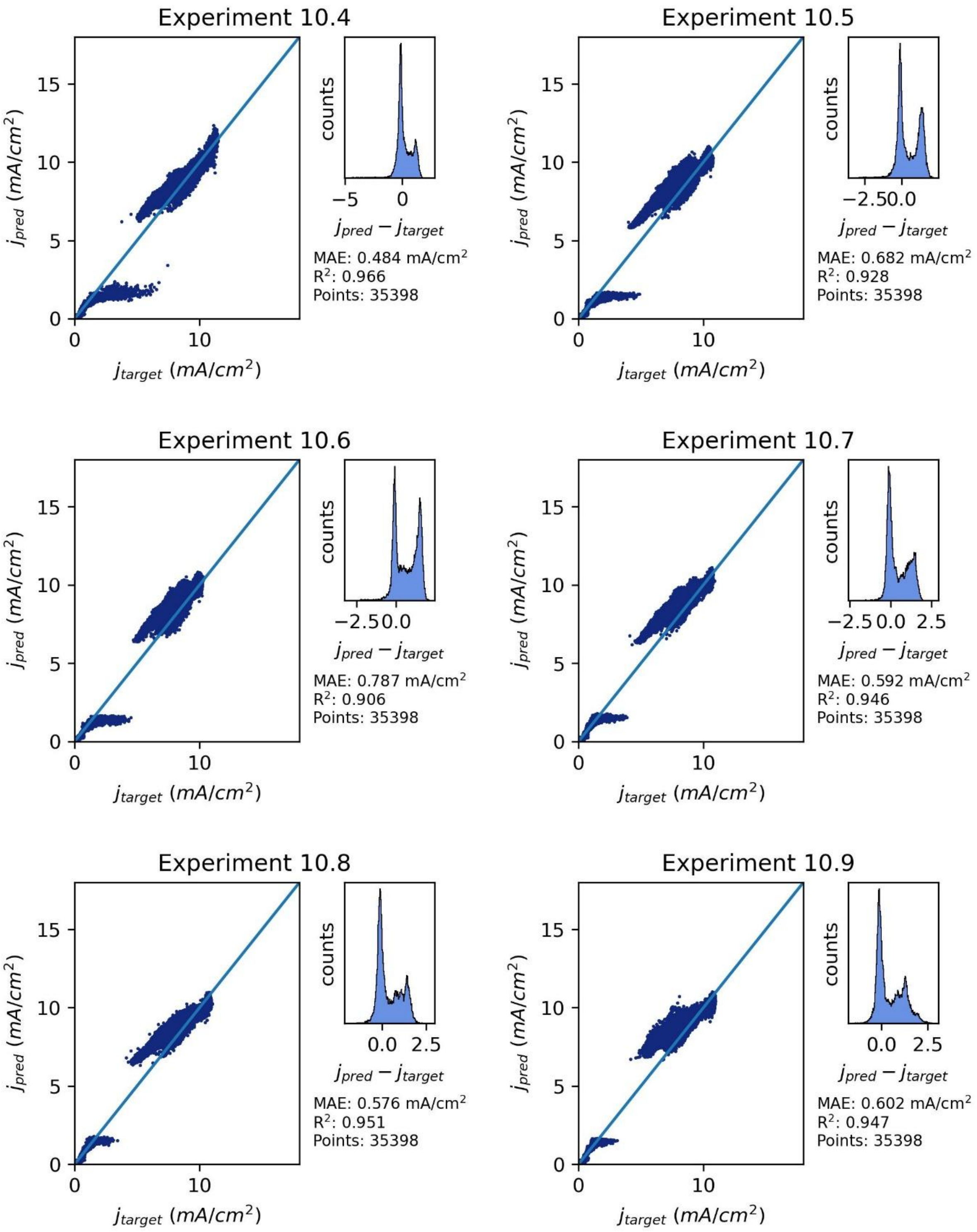

Experiment 10.4
MAE: 0.484 mA/cm^2
R^2: 0.966
Points: 35398
Experiment 10.5
MAE: 0.682 mA/cm^2
R^2: 0.928
Points: 35398
Experiment 10.6
MAE: 0.787 mA/cm^2
R^2: 0.906
Points: 35398
Experiment 10.7
MAE: 0.592 mA/cm^2
R^2: 0.946
Points: 35398
Experiment 10.8
MAE: 0.576 mA/cm^2
R^2: 0.951
Points: 35398
Experiment 10.9
MAE: 0.602 mA/cm^2
R^2: 0.947
Points: 35398
j_{pred} (mA/cm^2)
j_{target} (mA/cm^2)
counts
$j_{pred} - j_{target}$

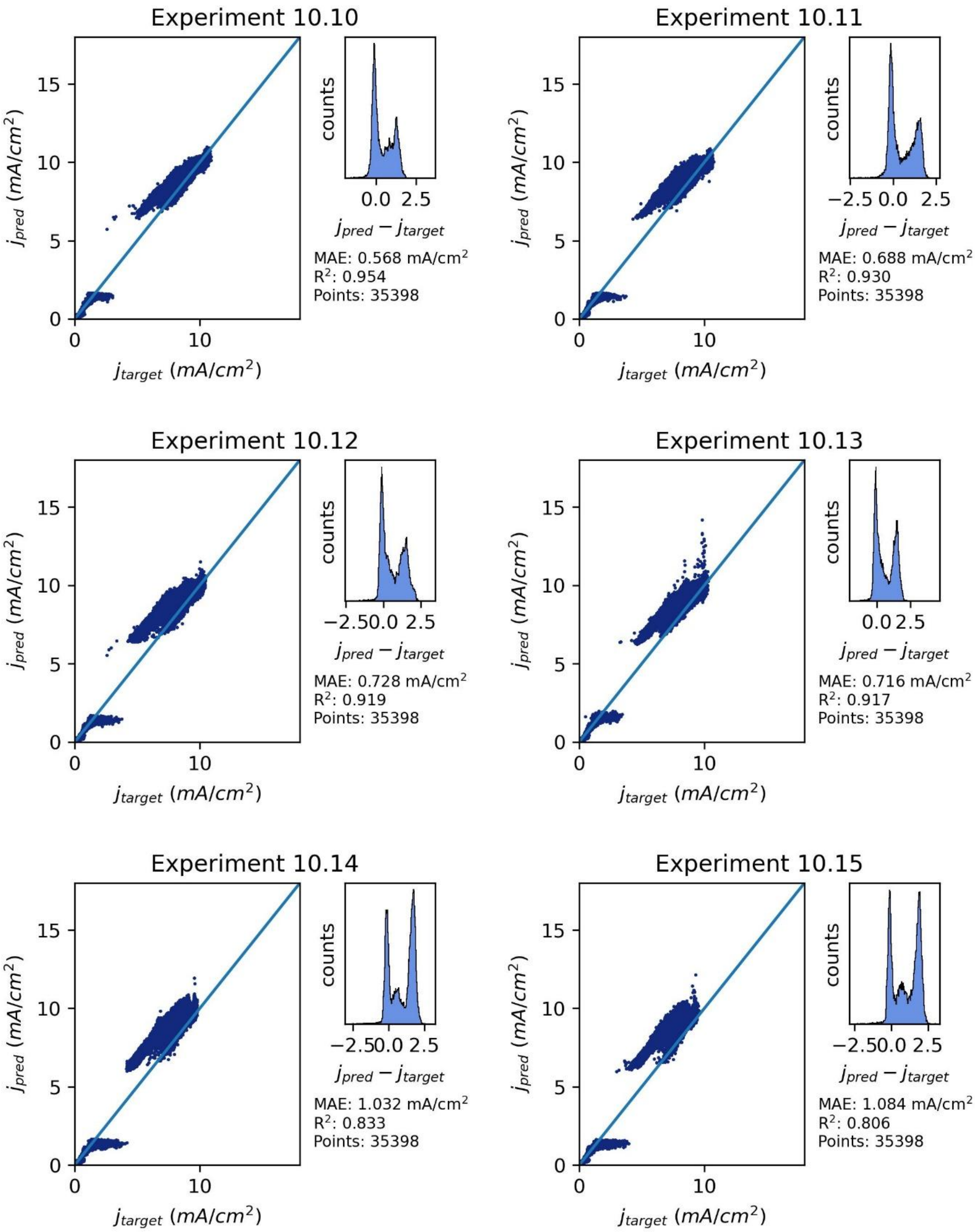
Experiment 10.10
MAE: 0.568 mA/cm^2
R^2: 0.954
Points: 35398
Experiment 10.11
MAE: 0.688 mA/cm^2
R^2: 0.930
Points: 35398
Experiment 10.12
MAE: 0.728 mA/cm^2
R^2: 0.919
Points: 35398
Experiment 10.13
MAE: 0.716 mA/cm^2
R^2: 0.917
Points: 35398
Experiment 10.14
MAE: 1.032 mA/cm^2
R^2: 0.833
Points: 35398
Experiment 10.15
MAE: 1.084 mA/cm^2
R^2: 0.806
Points: 35398
j_{pred} (mA/cm^2)
j_{target} (mA/cm^2)
counts
$j_{pred} - j_{target}$

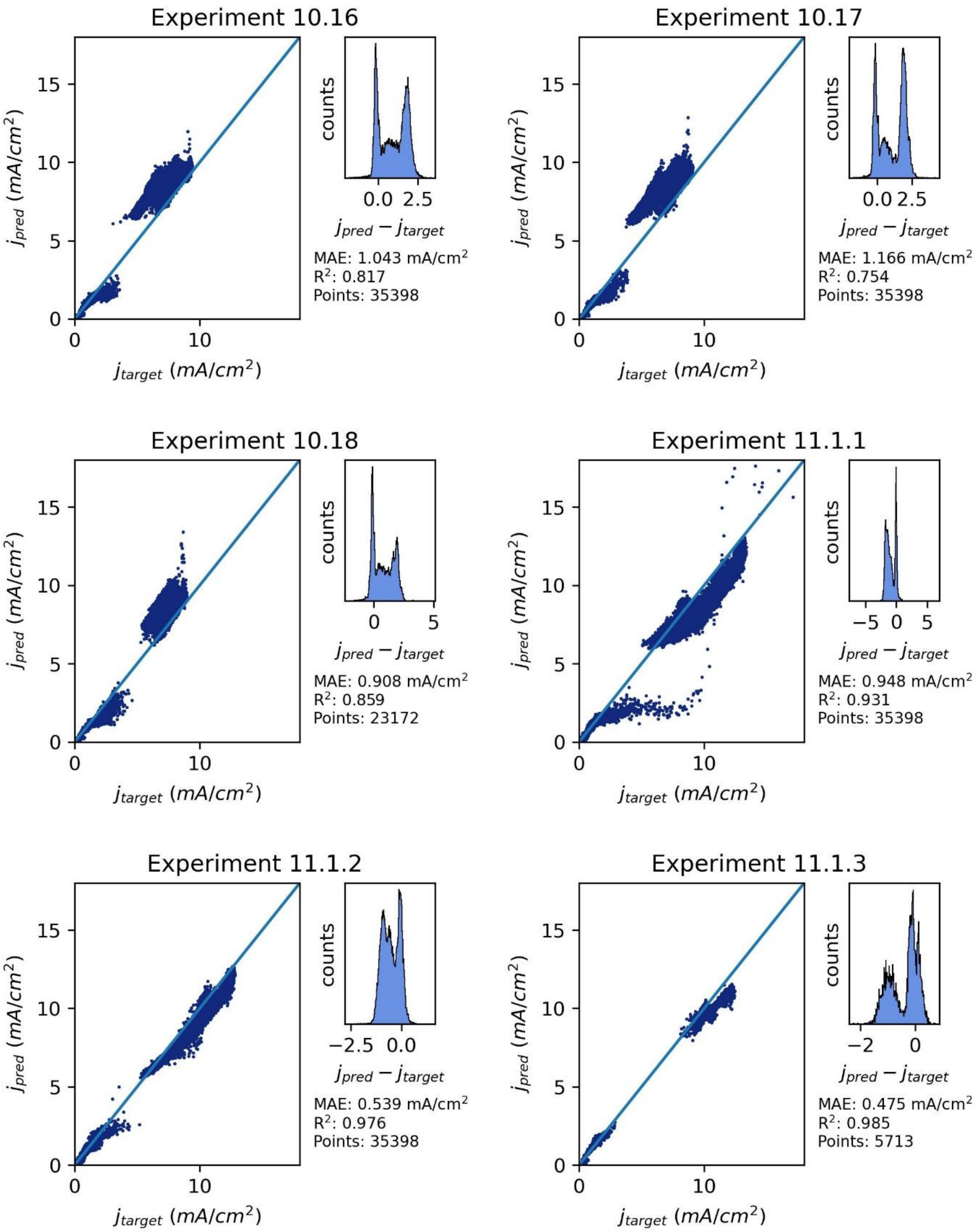
Experiment 10.16
j_pred (mA/cm^2)
j_target (mA/cm^2)
counts
j_pred − j_target
MAE: 1.043 mA/cm^2
R^2: 0.817
Points: 35398
Experiment 10.17
MAE: 1.166 mA/cm^2
R^2: 0.754
Points: 35398
Experiment 10.18
MAE: 0.908 mA/cm^2
R^2: 0.859
Points: 23172
Experiment 11.1.1
MAE: 0.948 mA/cm^2
R^2: 0.931
Points: 35398
Experiment 11.1.2
MAE: 0.539 mA/cm^2
R^2: 0.976
Points: 35398
Experiment 11.1.3
MAE: 0.475 mA/cm^2
R^2: 0.985
Points: 5713

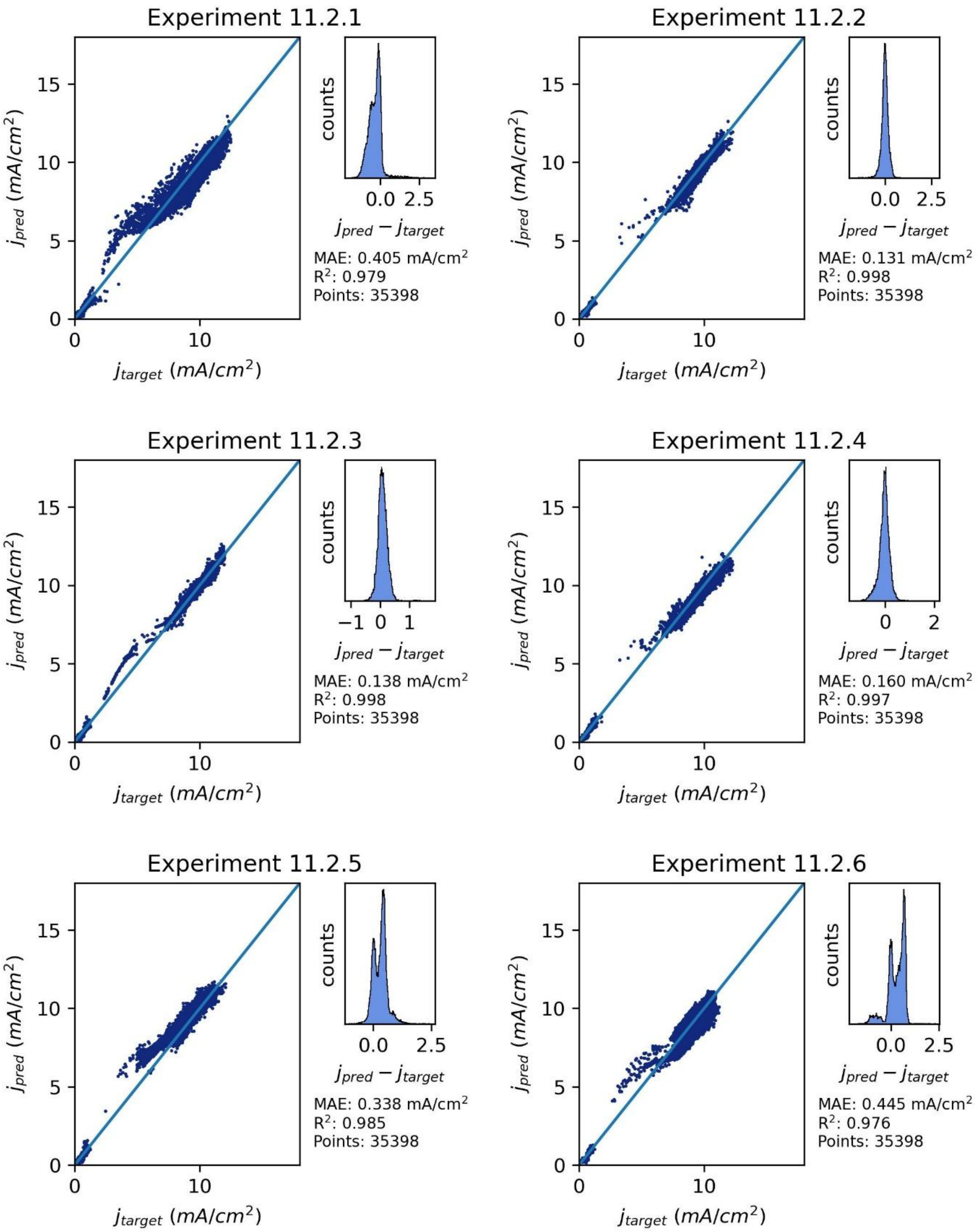
Experiment 11.2.1
MAE: 0.405 mA/cm^2
R^2: 0.979
Points: 35398
Experiment 11.2.2
MAE: 0.131 mA/cm^2
R^2: 0.998
Points: 35398
Experiment 11.2.3
MAE: 0.138 mA/cm^2
R^2: 0.998
Points: 35398
Experiment 11.2.4
MAE: 0.160 mA/cm^2
R^2: 0.997
Points: 35398
Experiment 11.2.5
MAE: 0.338 mA/cm^2
R^2: 0.985
Points: 35398
Experiment 11.2.6
MAE: 0.445 mA/cm^2
R^2: 0.976
Points: 35398
j_{pred} (mA/cm^2)
j_{target} (mA/cm^2)
counts
$j_{pred} - j_{target}$

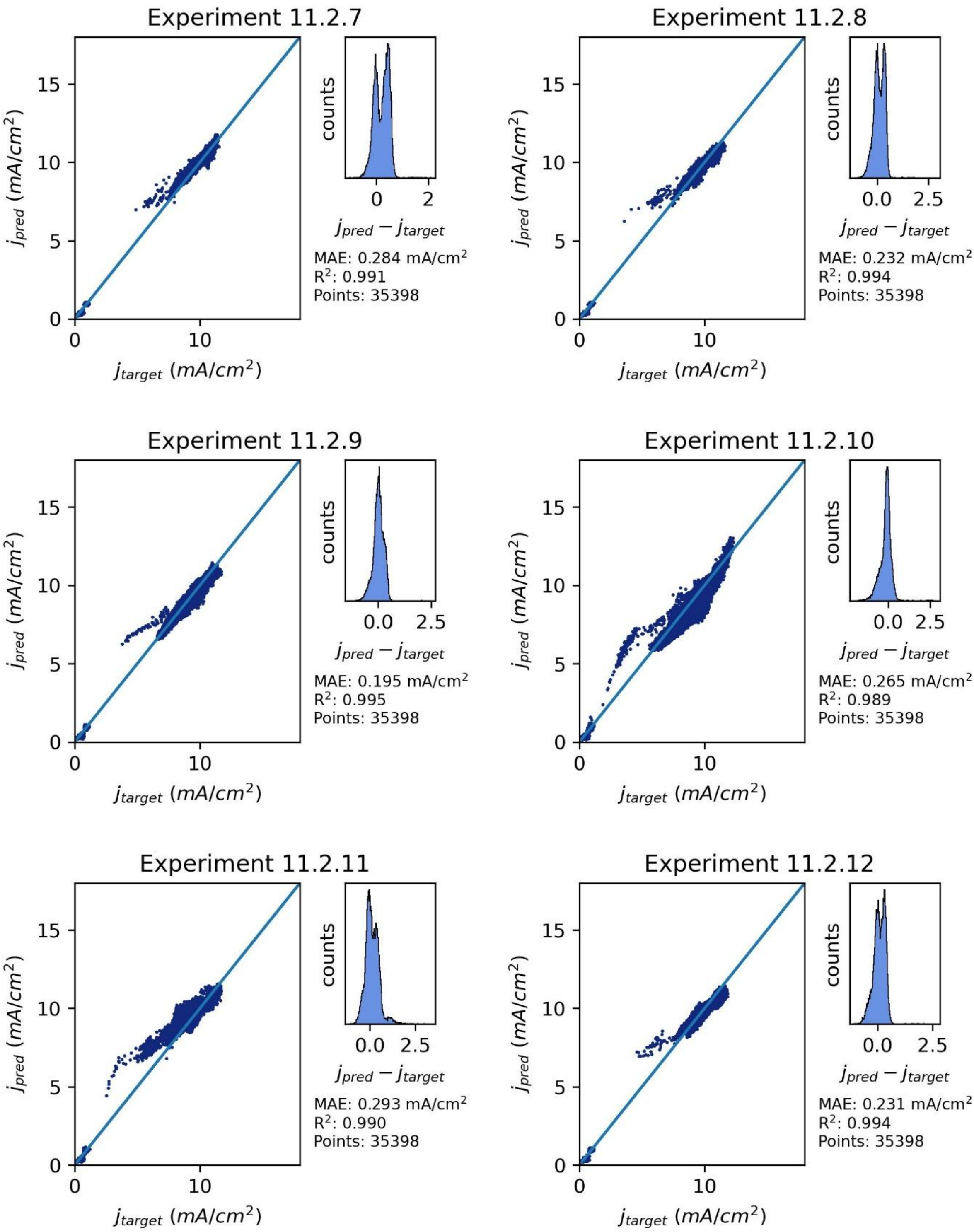

Experiment 11.2.7
MAE: 0.284 mA/cm^2
R^2: 0.991
Points: 35398
Experiment 11.2.8
MAE: 0.232 mA/cm^2
R^2: 0.994
Points: 35398
Experiment 11.2.9
MAE: 0.195 mA/cm^2
R^2: 0.995
Points: 35398
Experiment 11.2.10
MAE: 0.265 mA/cm^2
R^2: 0.989
Points: 35398
Experiment 11.2.11
MAE: 0.293 mA/cm^2
R^2: 0.990
Points: 35398
Experiment 11.2.12
MAE: 0.231 mA/cm^2
R^2: 0.994
Points: 35398
j_{pred} (mA/cm^2)
j_{target} (mA/cm^2)
counts
$j_{pred} - j_{target}$

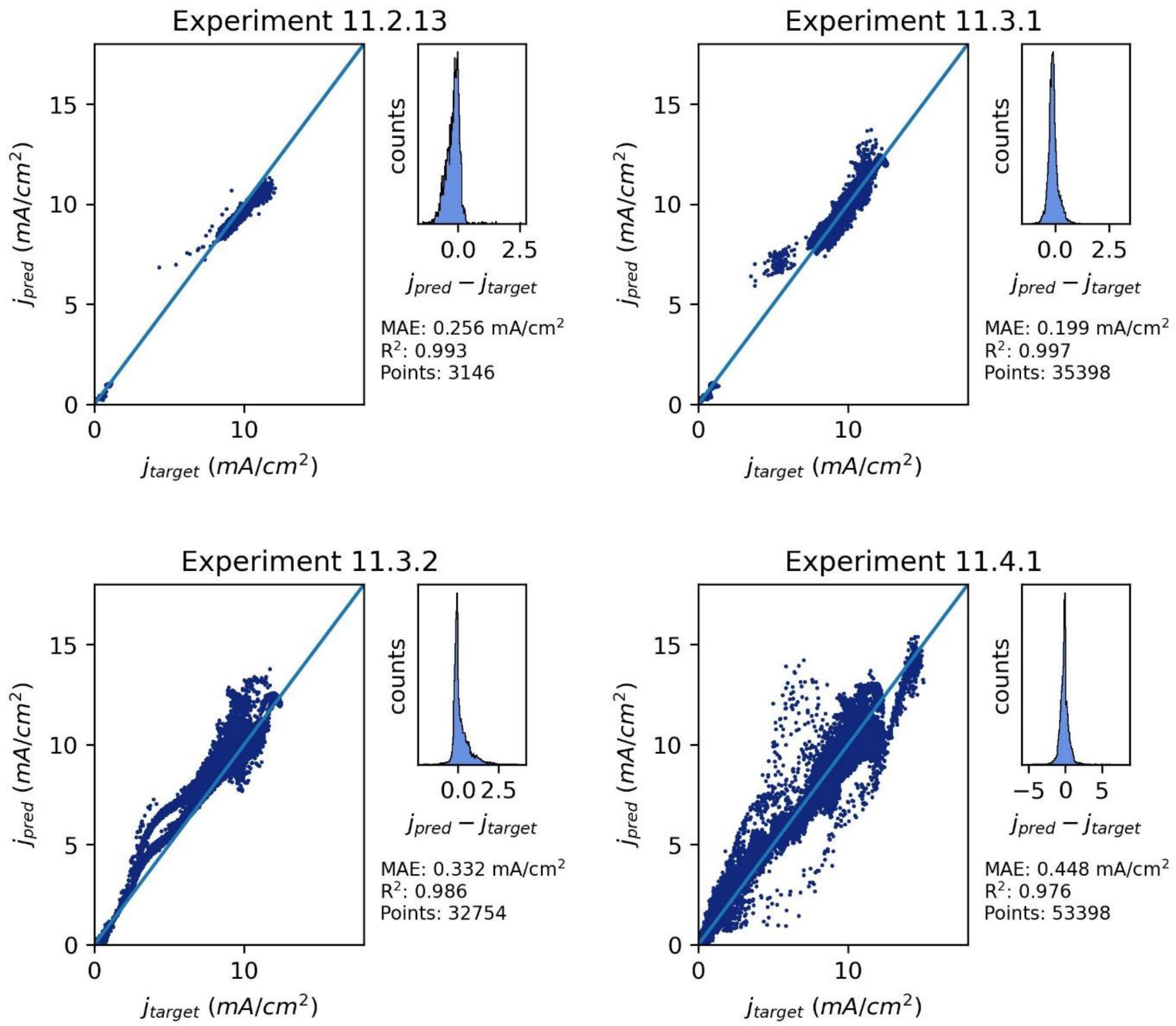

Figure S61. *Parity plots of the training set using a free running simulation with a burn in of 600 steps and the optimal checkpoint.*

VI.5 AI Model 2.2 – Ablated

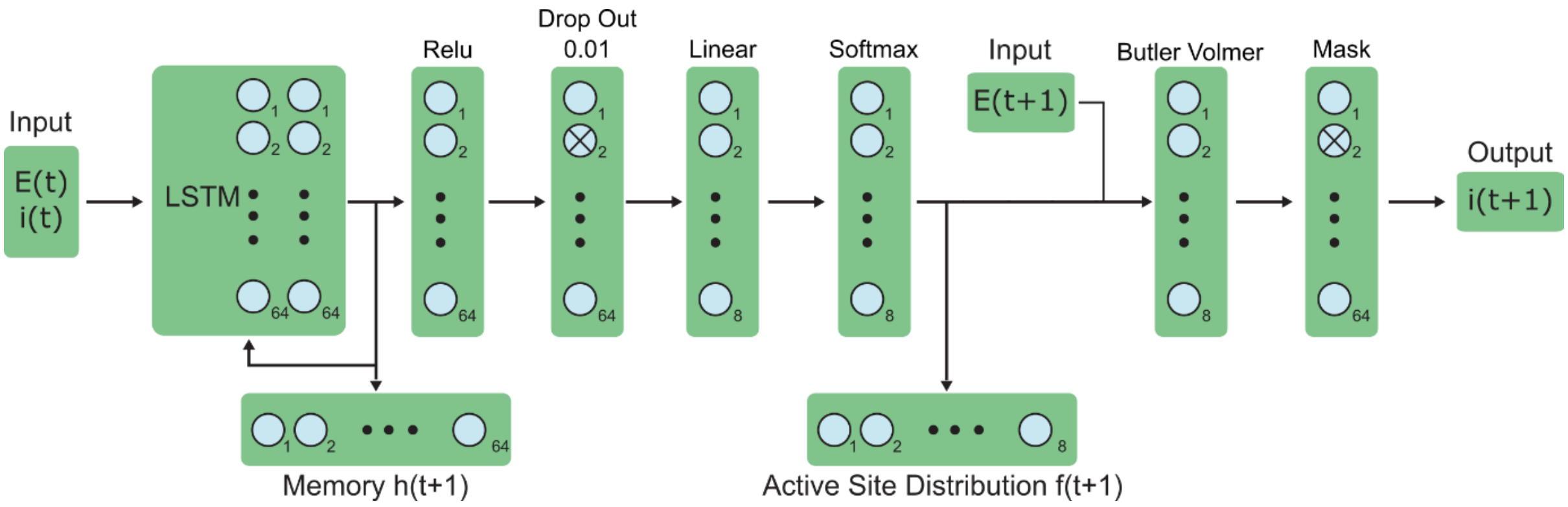

Figure S62. *The structure of the ablated AI Model 2.2. The mask shut sets the sites A, B, C, F, G to 0. The output layer is a sum of all incoming units.*

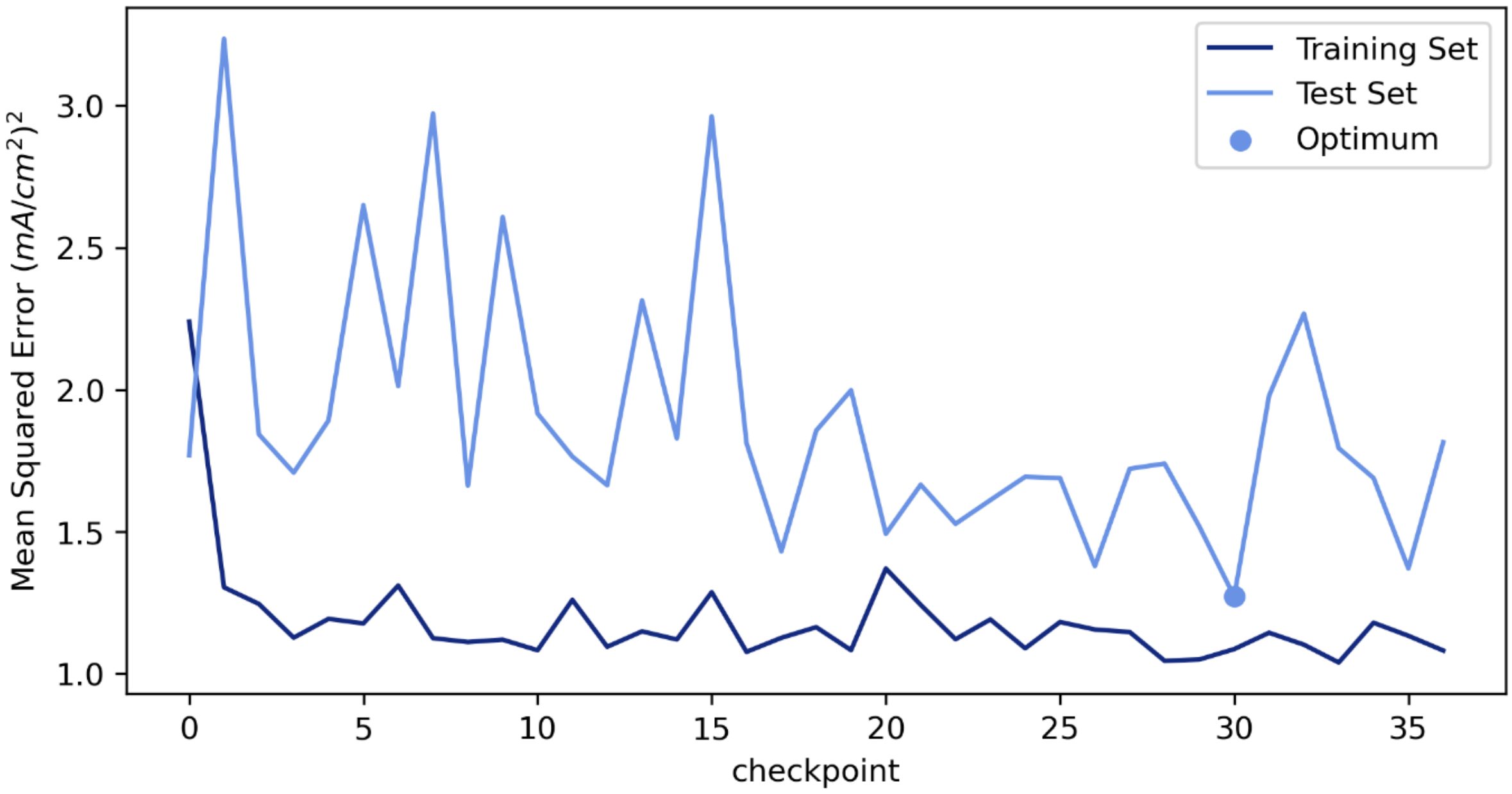

Figure S63. *Cross-validation scores vs. checkpoint number. The training and test datasets were segmented into chunks of 30 000 observations, and any remaining tails containing fewer than 30 000 observations were discarded. For each segment, the first 600 points were used to burn in the hidden state, while the remaining 29 400 points were used to evaluate the checkpoint performance.*

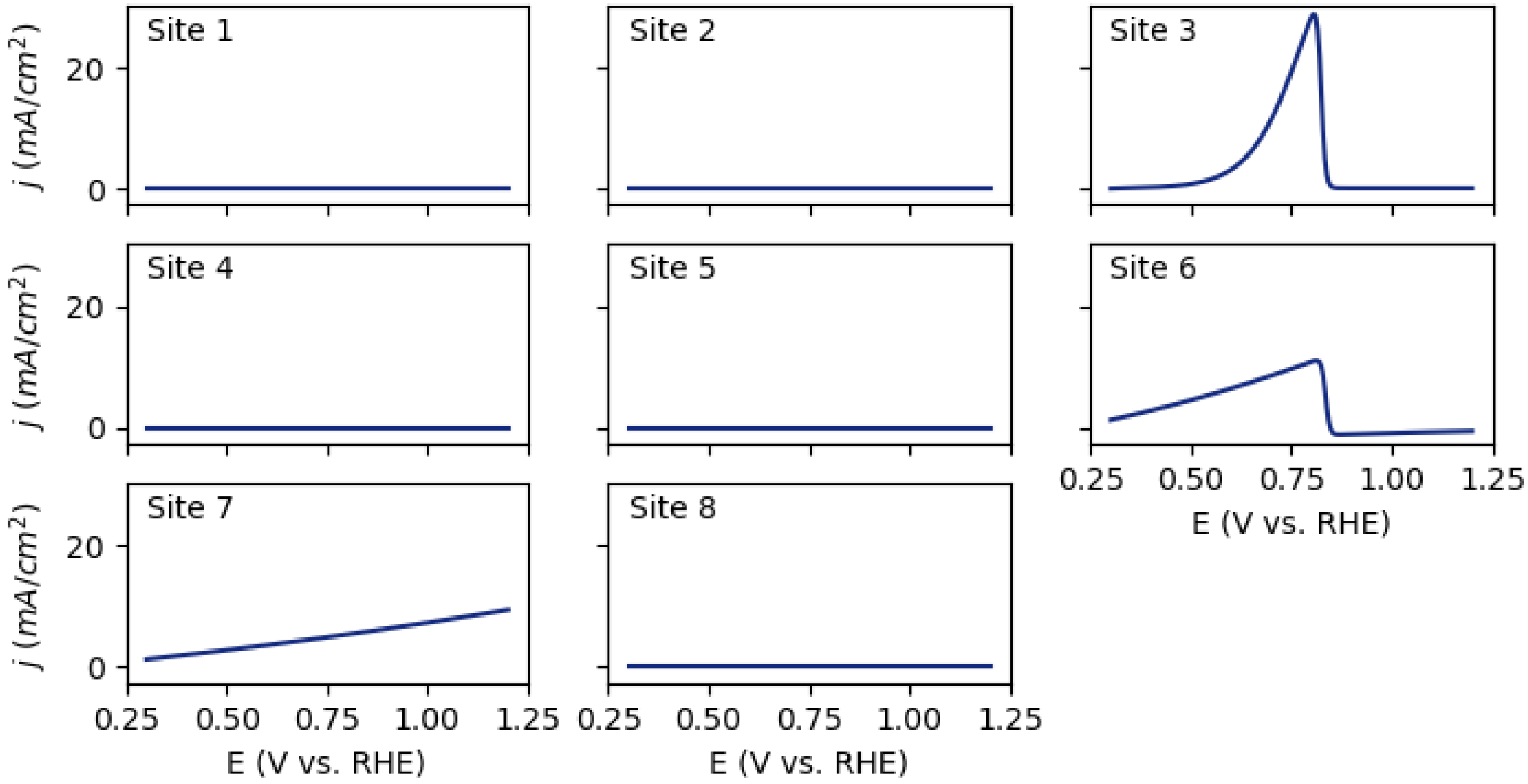

Figure S64. *Visualization of the Butler-Volmer layer for the optimal checkpoint. The curves are plotted assuming that the active site has the maximum coverage of 1.*

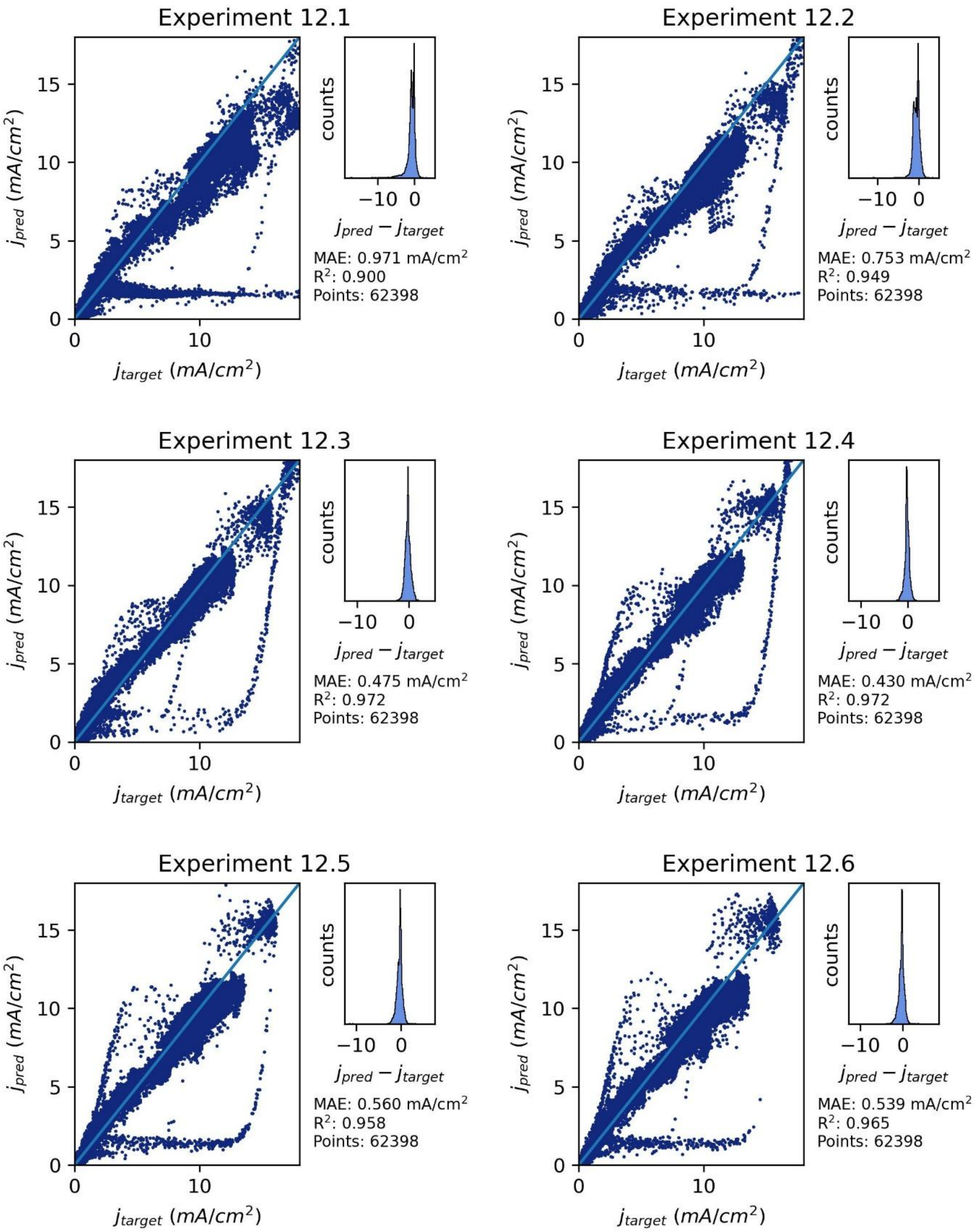
Experiment 12.1
j_{pred} (mA/cm^2)
j_{target} (mA/cm^2)
counts
$j_{pred} - j_{target}$
MAE: 0.971 mA/cm^2
R^2: 0.900
Points: 62398
Experiment 12.2
MAE: 0.753 mA/cm^2
R^2: 0.949
Points: 62398
Experiment 12.3
MAE: 0.475 mA/cm^2
R^2: 0.972
Points: 62398
Experiment 12.4
MAE: 0.430 mA/cm^2
R^2: 0.972
Points: 62398
Experiment 12.5
MAE: 0.560 mA/cm^2
R^2: 0.958
Points: 62398
Experiment 12.6
MAE: 0.539 mA/cm^2
R^2: 0.965
Points: 62398

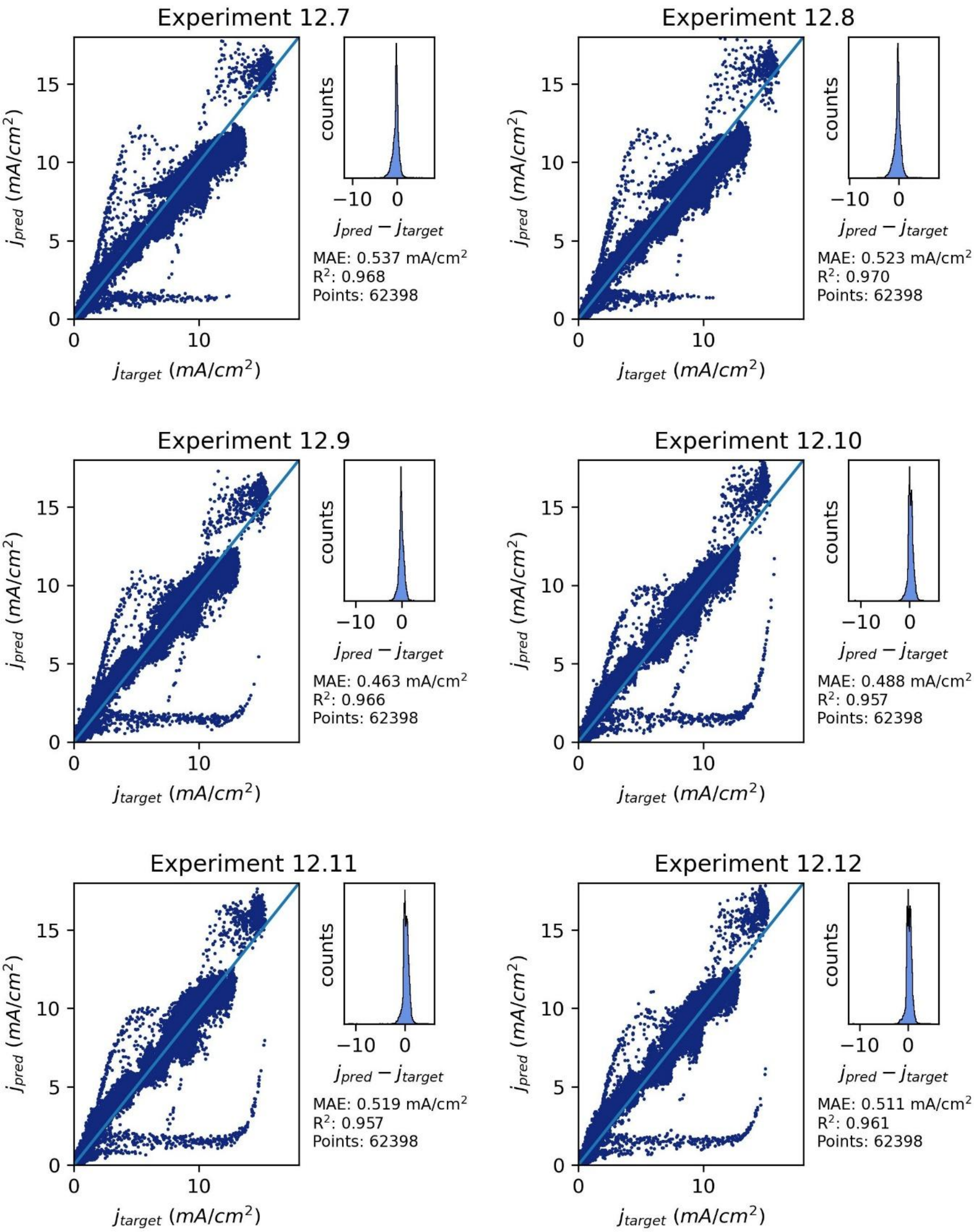
Experiment 12.7
j_{pred} (mA/cm^2)
j_{target} (mA/cm^2)
counts
$j_{pred} - j_{target}$
MAE: 0.537 mA/cm^2
R^2: 0.968
Points: 62398
Experiment 12.8
j_{pred} (mA/cm^2)
j_{target} (mA/cm^2)
counts
$j_{pred} - j_{target}$
MAE: 0.523 mA/cm^2
R^2: 0.970
Points: 62398
Experiment 12.9
j_{pred} (mA/cm^2)
j_{target} (mA/cm^2)
counts
$j_{pred} - j_{target}$
MAE: 0.463 mA/cm^2
R^2: 0.966
Points: 62398
Experiment 12.10
j_{pred} (mA/cm^2)
j_{target} (mA/cm^2)
counts
$j_{pred} - j_{target}$
MAE: 0.488 mA/cm^2
R^2: 0.957
Points: 62398
Experiment 12.11
j_{pred} (mA/cm^2)
j_{target} (mA/cm^2)
counts
$j_{pred} - j_{target}$
MAE: 0.519 mA/cm^2
R^2: 0.957
Points: 62398
Experiment 12.12
j_{pred} (mA/cm^2)
j_{target} (mA/cm^2)
counts
$j_{pred} - j_{target}$
MAE: 0.511 mA/cm^2
R^2: 0.961
Points: 62398

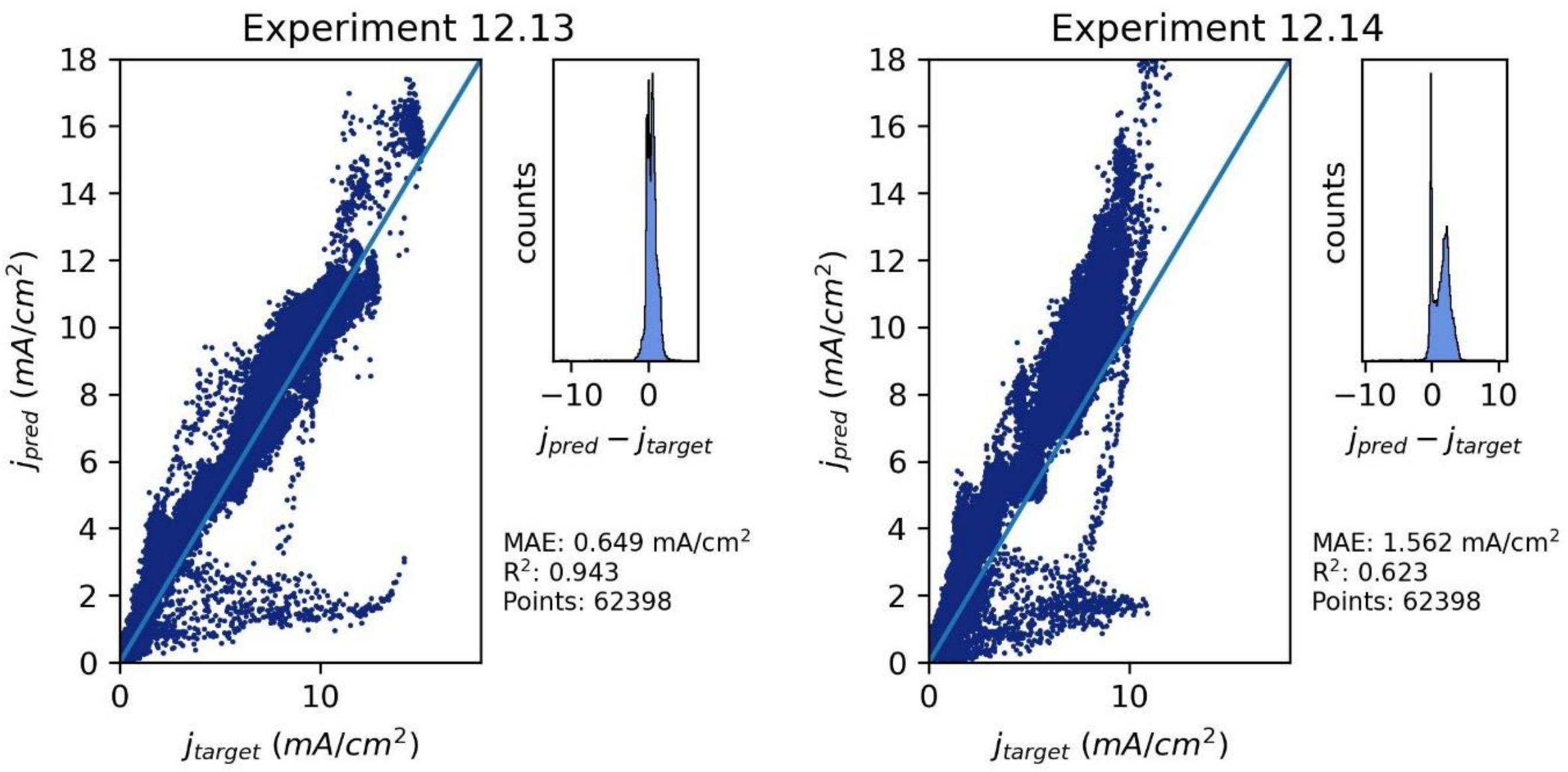

Figure S65. *Parity plots of the test set using a free running simulation with a burn in of 600 steps and the optimal checkpoint.*

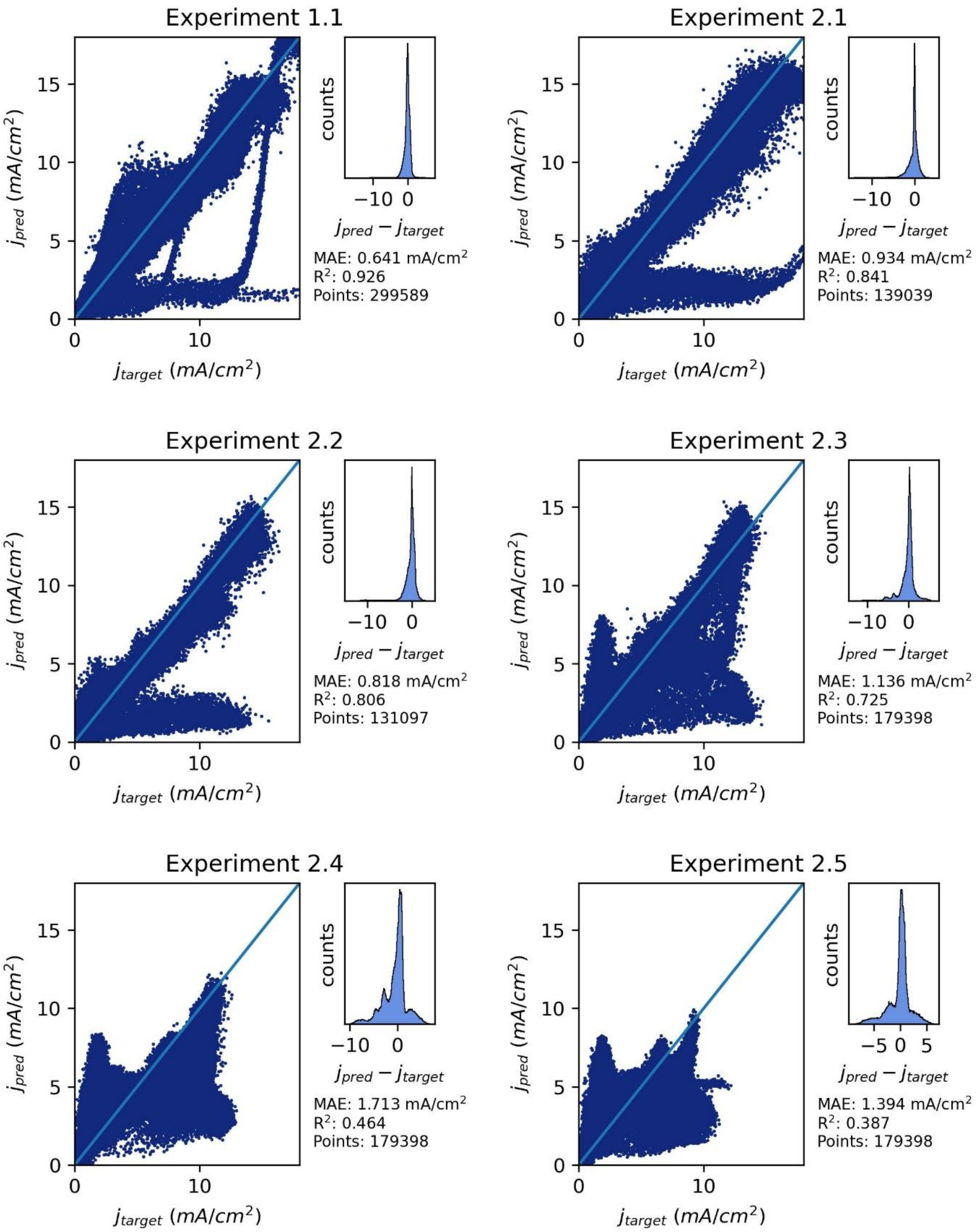
Experiment 1.1
MAE: 0.641 mA/cm^2
R^2: 0.926
Points: 299589
Experiment 2.1
MAE: 0.934 mA/cm^2
R^2: 0.841
Points: 139039
Experiment 2.2
MAE: 0.818 mA/cm^2
R^2: 0.806
Points: 131097
Experiment 2.3
MAE: 1.136 mA/cm^2
R^2: 0.725
Points: 179398
Experiment 2.4
MAE: 1.713 mA/cm^2
R^2: 0.464
Points: 179398
Experiment 2.5
MAE: 1.394 mA/cm^2
R^2: 0.387
Points: 179398
j_{pred} (mA/cm^2)
j_{target} (mA/cm^2)
counts
$j_{pred} - j_{target}$

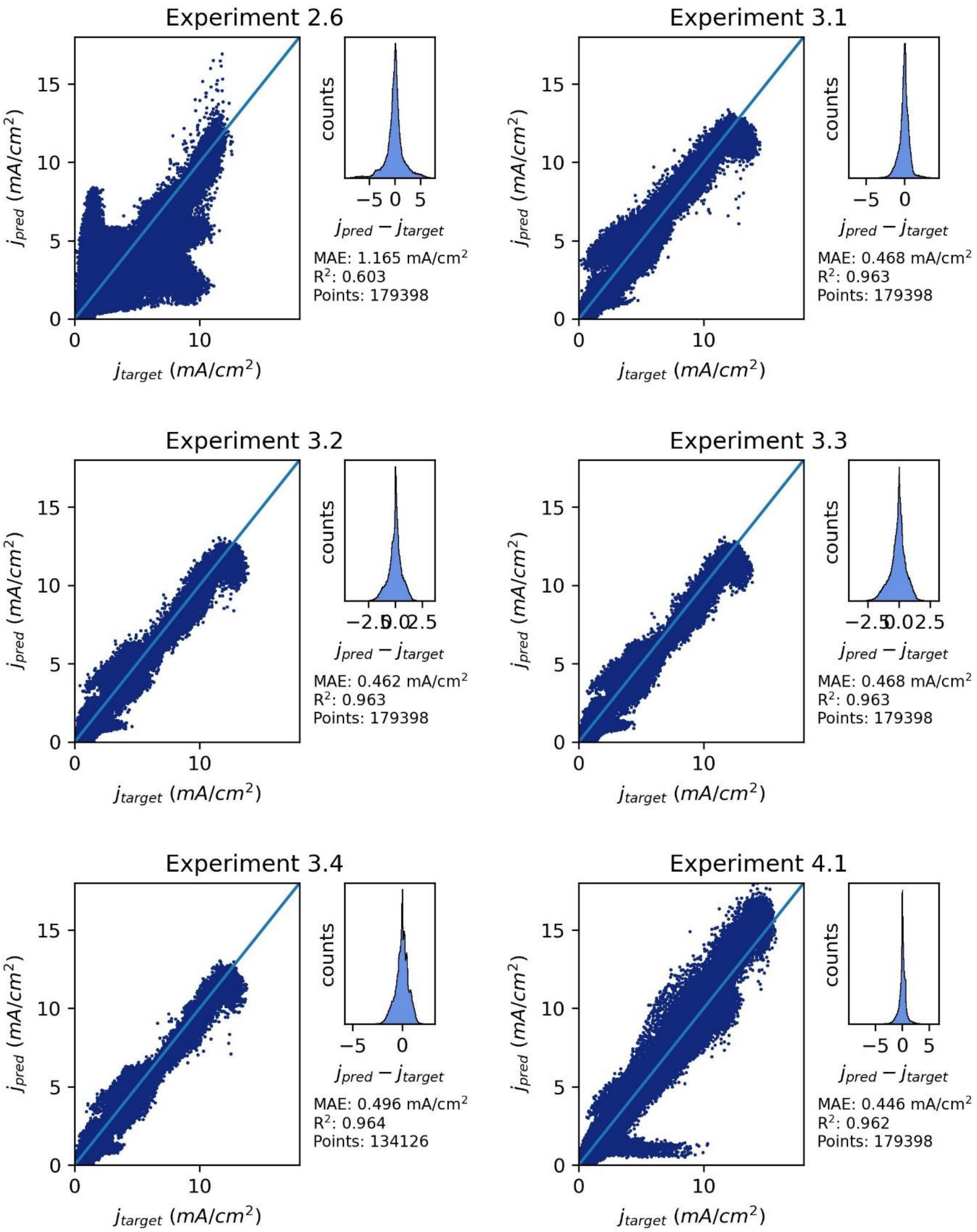

Experiment 2.6
MAE: 1.165 mA/cm²
R²: 0.603
Points: 179398
Experiment 3.1
MAE: 0.468 mA/cm²
R²: 0.963
Points: 179398
Experiment 3.2
MAE: 0.462 mA/cm²
R²: 0.963
Points: 179398
Experiment 3.3
MAE: 0.468 mA/cm²
R²: 0.963
Points: 179398
Experiment 3.4
MAE: 0.496 mA/cm²
R²: 0.964
Points: 134126
Experiment 4.1
MAE: 0.446 mA/cm²
R²: 0.962
Points: 179398
j_pred (mA/cm²)
j_target (mA/cm²)
counts
j_pred − j_target

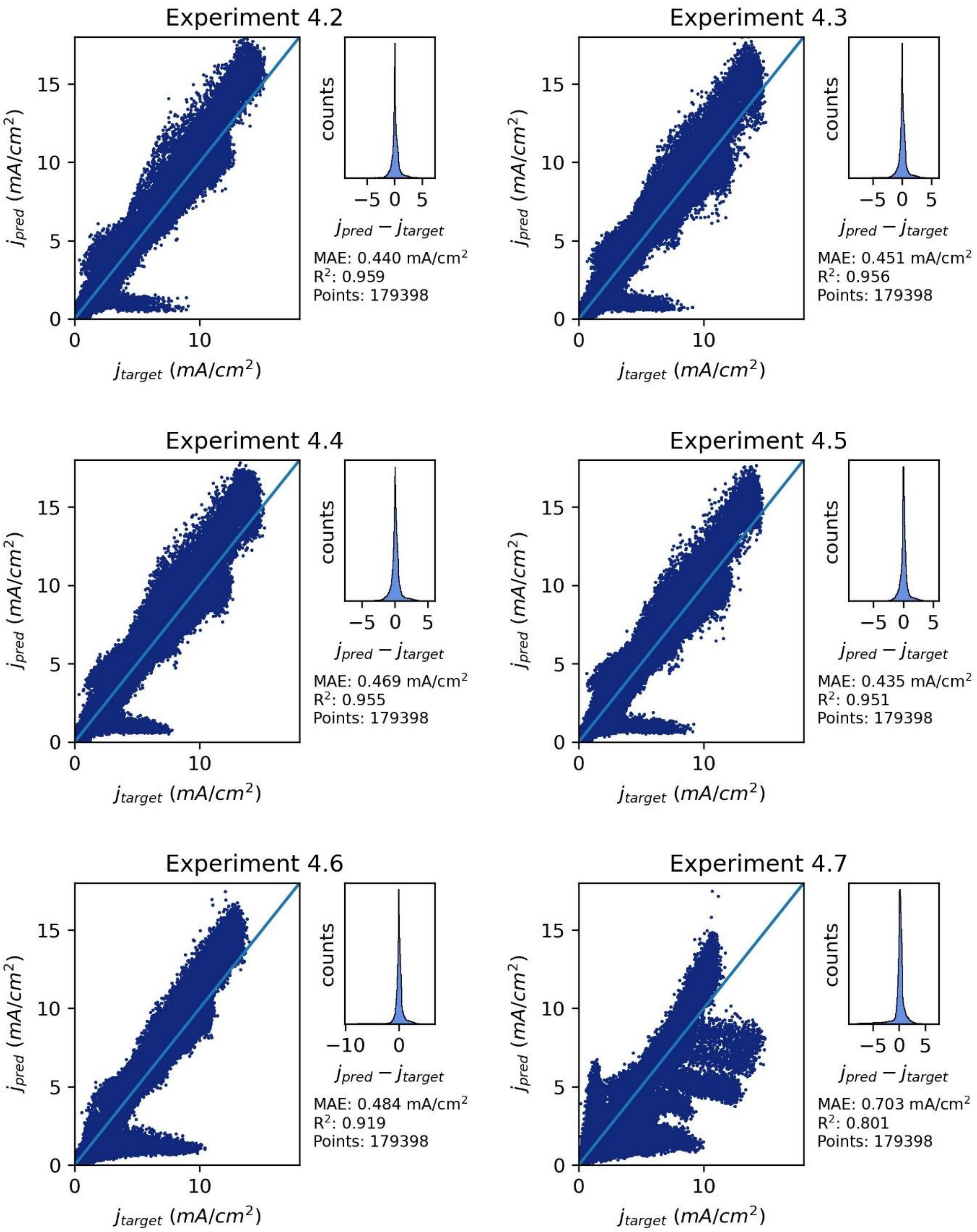
Experiment 4.2
MAE: 0.440 mA/cm^2
R^2: 0.959
Points: 179398
Experiment 4.3
MAE: 0.451 mA/cm^2
R^2: 0.956
Points: 179398
Experiment 4.4
MAE: 0.469 mA/cm^2
R^2: 0.955
Points: 179398
Experiment 4.5
MAE: 0.435 mA/cm^2
R^2: 0.951
Points: 179398
Experiment 4.6
MAE: 0.484 mA/cm^2
R^2: 0.919
Points: 179398
Experiment 4.7
MAE: 0.703 mA/cm^2
R^2: 0.801
Points: 179398
j_{pred} (mA/cm^2)
j_{target} (mA/cm^2)
counts
$j_{pred} - j_{target}$

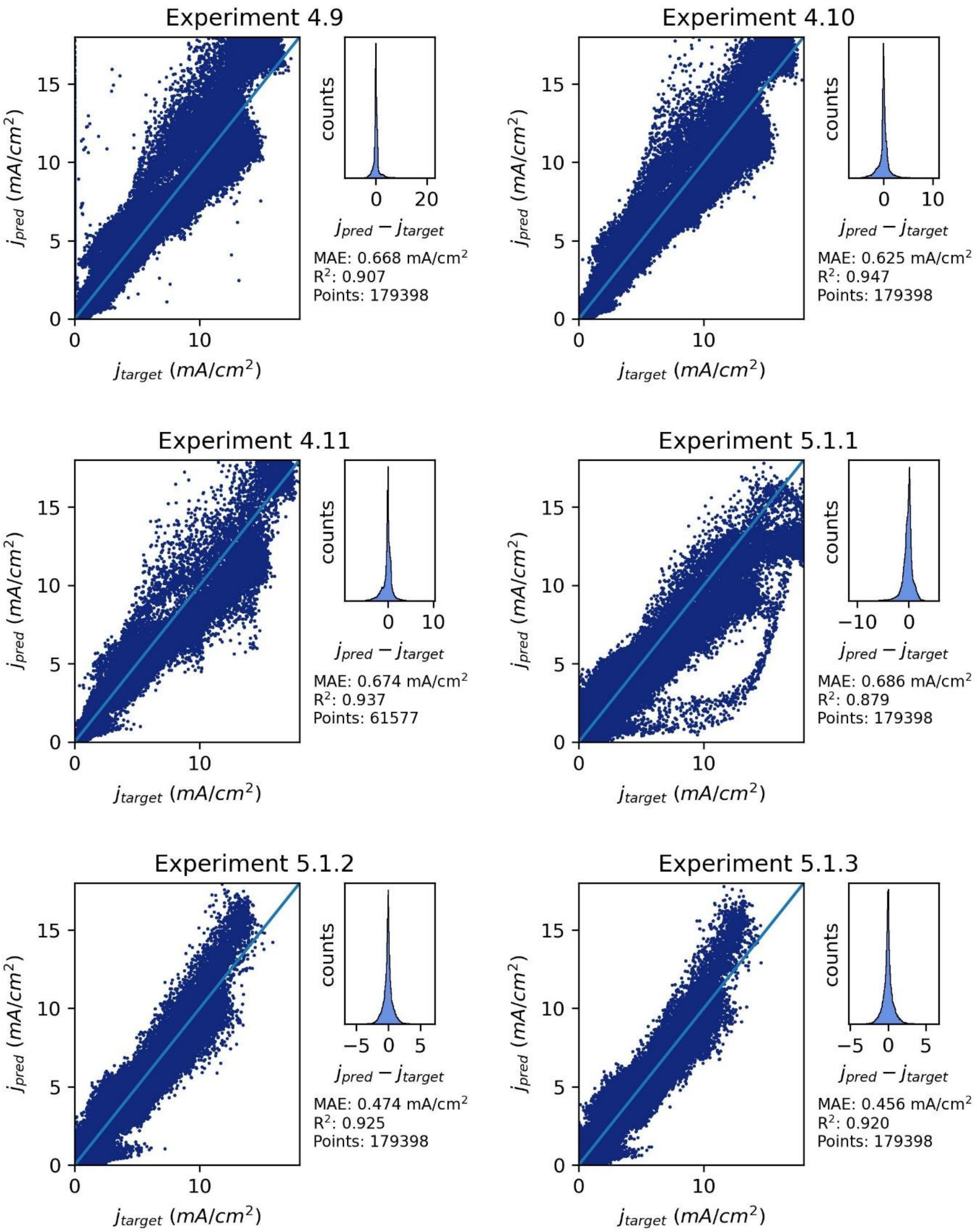
Experiment 4.9
MAE: 0.668 mA/cm2
R2: 0.907
Points: 179398
Experiment 4.10
MAE: 0.625 mA/cm2
R2: 0.947
Points: 179398
Experiment 4.11
MAE: 0.674 mA/cm2
R2: 0.937
Points: 61577
Experiment 5.1.1
MAE: 0.686 mA/cm2
R2: 0.879
Points: 179398
Experiment 5.1.2
MAE: 0.474 mA/cm2
R2: 0.925
Points: 179398
Experiment 5.1.3
MAE: 0.456 mA/cm2
R2: 0.920
Points: 179398
j_pred (mA/cm2)
j_target (mA/cm2)
counts
j_pred − j_target

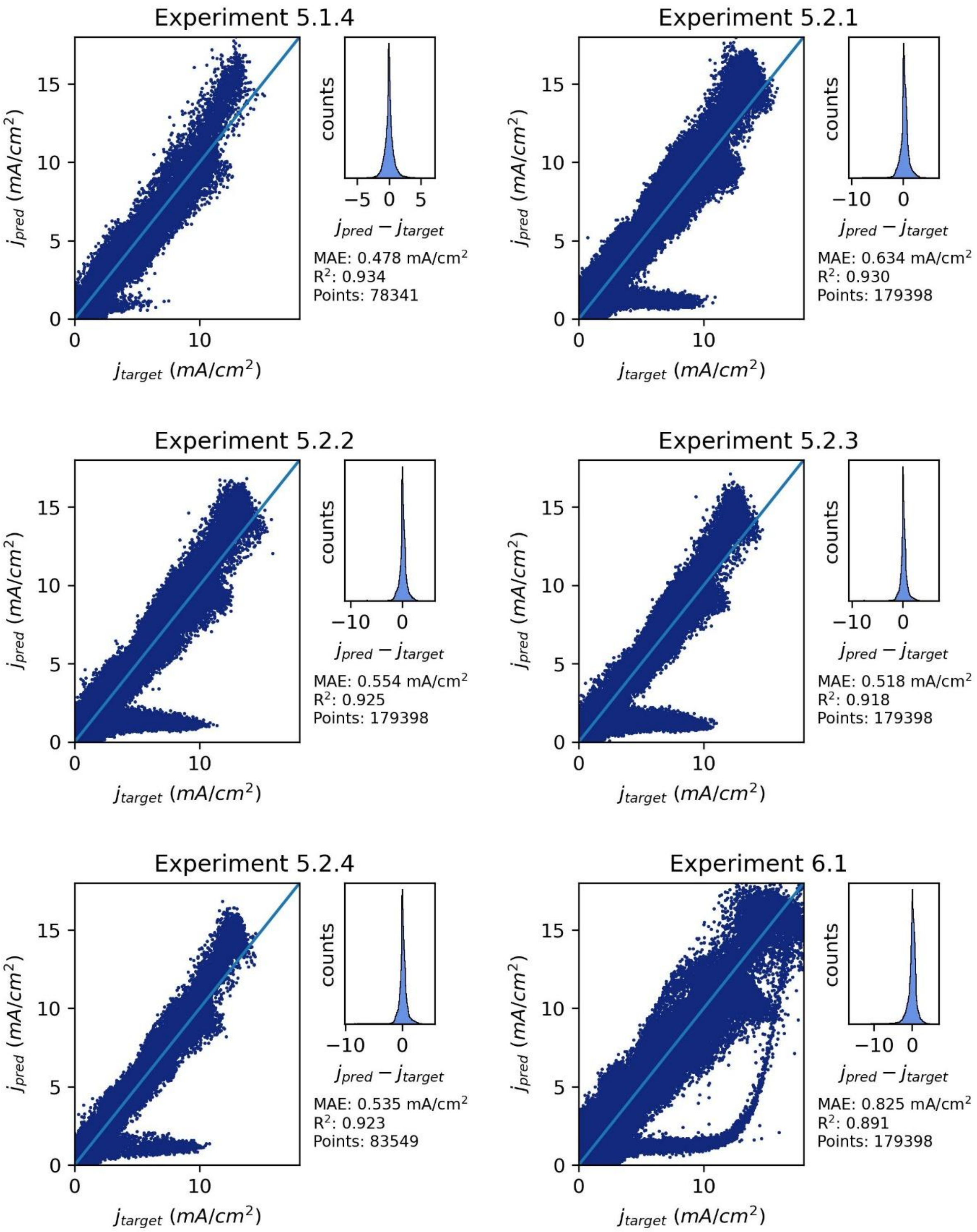

Experiment 5.1.4
MAE: 0.478 mA/cm^2
R^2: 0.934
Points: 78341
Experiment 5.2.1
MAE: 0.634 mA/cm^2
R^2: 0.930
Points: 179398
Experiment 5.2.2
MAE: 0.554 mA/cm^2
R^2: 0.925
Points: 179398
Experiment 5.2.3
MAE: 0.518 mA/cm^2
R^2: 0.918
Points: 179398
Experiment 5.2.4
MAE: 0.535 mA/cm^2
R^2: 0.923
Points: 83549
Experiment 6.1
MAE: 0.825 mA/cm^2
R^2: 0.891
Points: 179398
j_{pred} (mA/cm^2)
j_{target} (mA/cm^2)
counts
$j_{pred} - j_{target}$

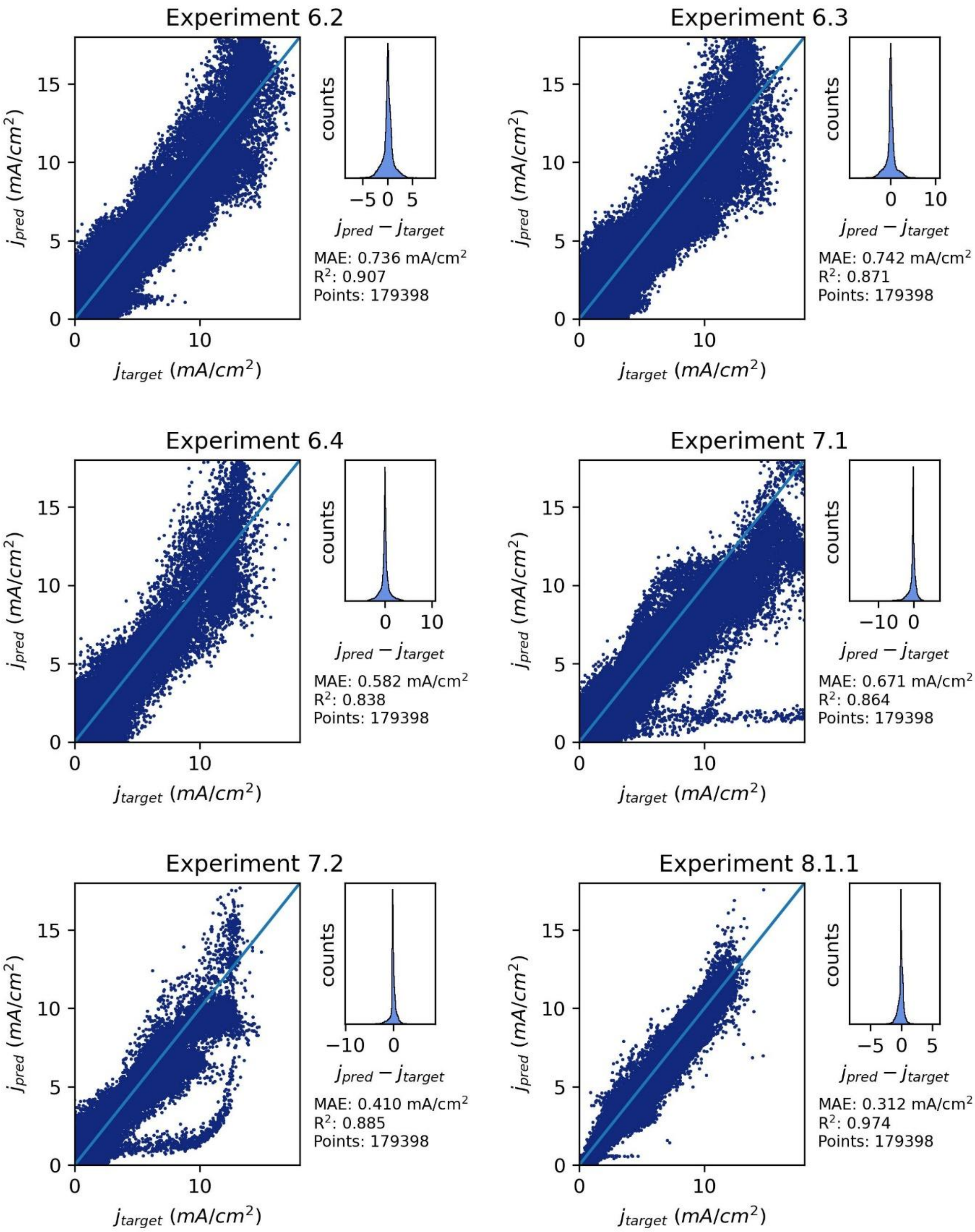

Experiment 6.2
MAE: 0.736 mA/cm²
R²: 0.907
Points: 179398
Experiment 6.3
MAE: 0.742 mA/cm²
R²: 0.871
Points: 179398
Experiment 6.4
MAE: 0.582 mA/cm²
R²: 0.838
Points: 179398
Experiment 7.1
MAE: 0.671 mA/cm²
R²: 0.864
Points: 179398
Experiment 7.2
MAE: 0.410 mA/cm²
R²: 0.885
Points: 179398
Experiment 8.1.1
MAE: 0.312 mA/cm²
R²: 0.974
Points: 179398
j_pred (mA/cm²)
j_target (mA/cm²)
counts
j_pred − j_target

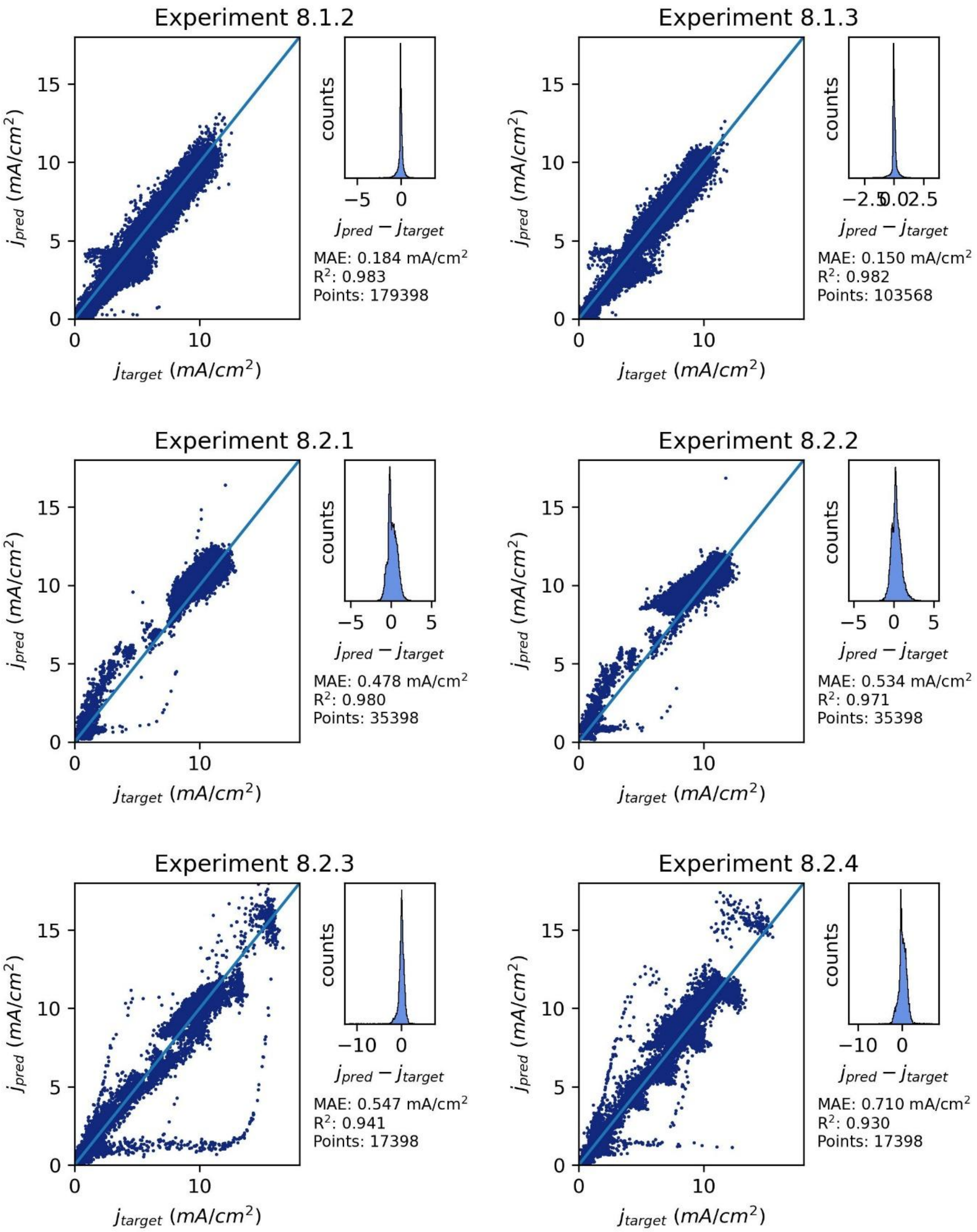

Experiment 8.1.2
j_{pred} (mA/cm^2)
j_{target} (mA/cm^2)
counts
$j_{pred} - j_{target}$
MAE: 0.184 mA/cm^2
R^2: 0.983
Points: 179398
Experiment 8.1.3
j_{pred} (mA/cm^2)
j_{target} (mA/cm^2)
counts
$j_{pred} - j_{target}$
MAE: 0.150 mA/cm^2
R^2: 0.982
Points: 103568
Experiment 8.2.1
j_{pred} (mA/cm^2)
j_{target} (mA/cm^2)
counts
$j_{pred} - j_{target}$
MAE: 0.478 mA/cm^2
R^2: 0.980
Points: 35398
Experiment 8.2.2
j_{pred} (mA/cm^2)
j_{target} (mA/cm^2)
counts
$j_{pred} - j_{target}$
MAE: 0.534 mA/cm^2
R^2: 0.971
Points: 35398
Experiment 8.2.3
j_{pred} (mA/cm^2)
j_{target} (mA/cm^2)
counts
$j_{pred} - j_{target}$
MAE: 0.547 mA/cm^2
R^2: 0.941
Points: 17398
Experiment 8.2.4
j_{pred} (mA/cm^2)
j_{target} (mA/cm^2)
counts
$j_{pred} - j_{target}$
MAE: 0.710 mA/cm^2
R^2: 0.930
Points: 17398

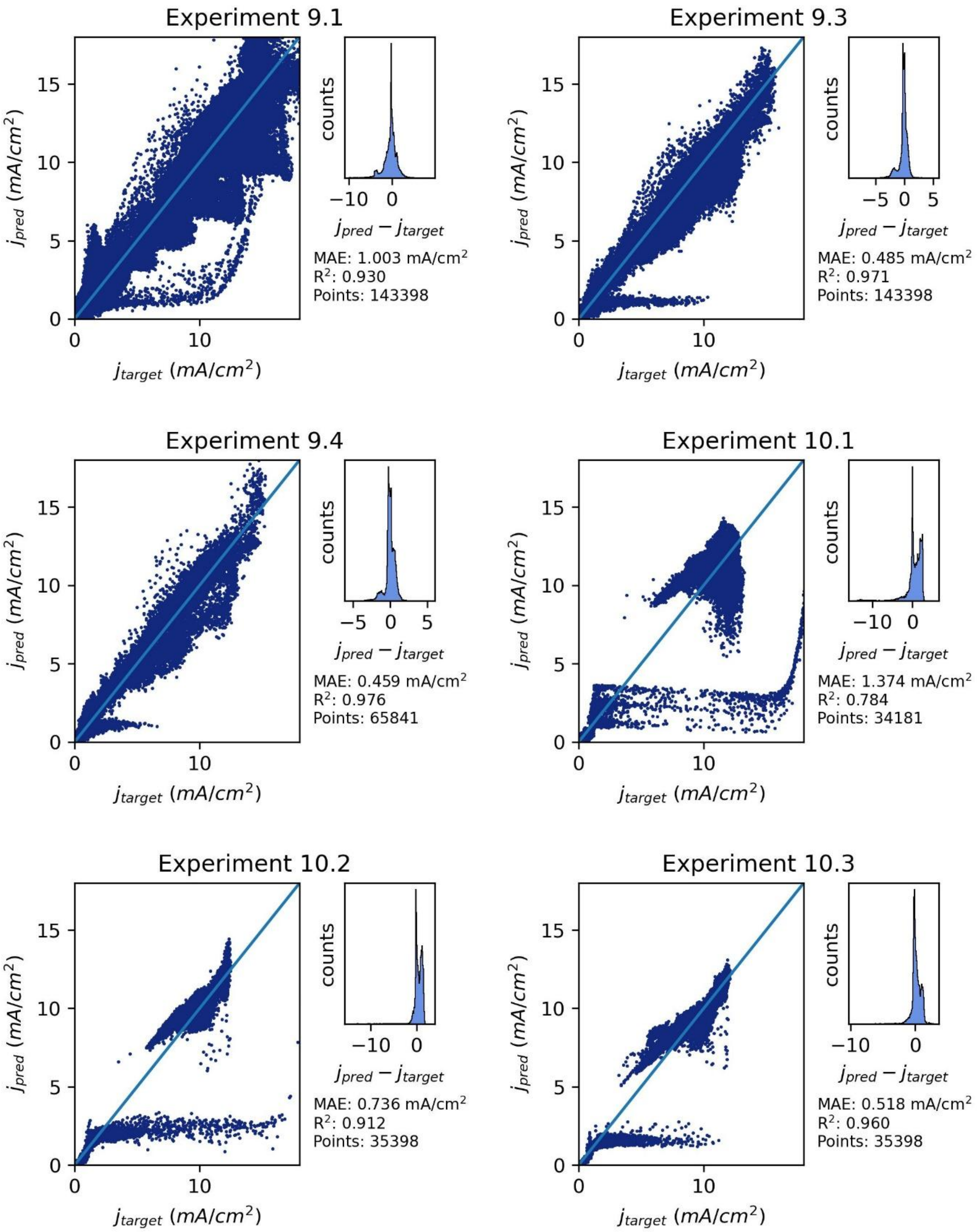
Experiment 9.1
MAE: 1.003 mA/cm^2
R^2: 0.930
Points: 143398
Experiment 9.3
MAE: 0.485 mA/cm^2
R^2: 0.971
Points: 143398
Experiment 9.4
MAE: 0.459 mA/cm^2
R^2: 0.976
Points: 65841
Experiment 10.1
MAE: 1.374 mA/cm^2
R^2: 0.784
Points: 34181
Experiment 10.2
MAE: 0.736 mA/cm^2
R^2: 0.912
Points: 35398
Experiment 10.3
MAE: 0.518 mA/cm^2
R^2: 0.960
Points: 35398
j_{pred} (mA/cm^2)
j_{target} (mA/cm^2)
counts
$j_{pred} - j_{target}$

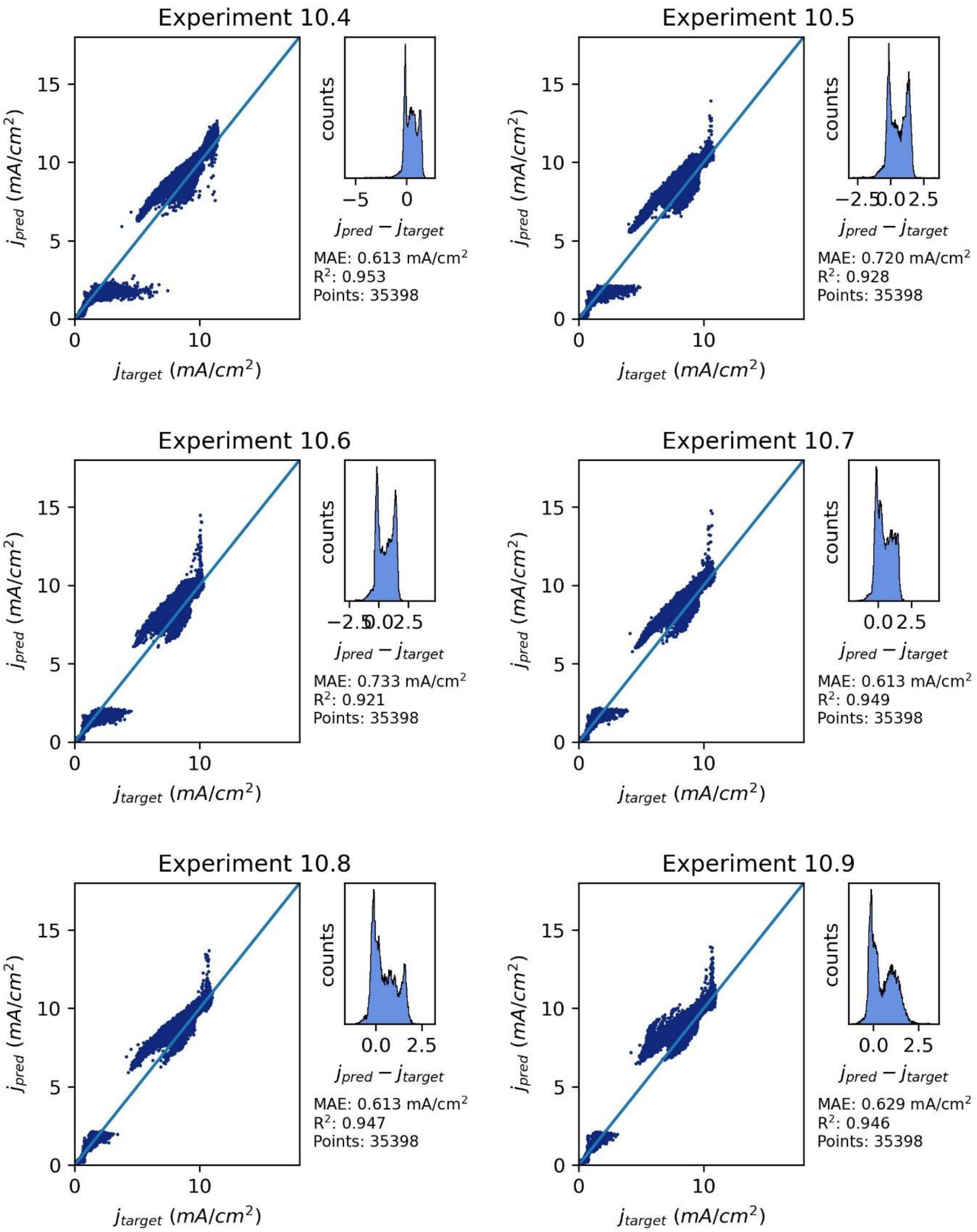
Experiment 10.4
j_{pred} (mA/cm^2)
j_{target} (mA/cm^2)
counts
$j_{pred} - j_{target}$
MAE: 0.613 mA/cm^2
R^2: 0.953
Points: 35398
Experiment 10.5
j_{pred} (mA/cm^2)
j_{target} (mA/cm^2)
counts
$j_{pred} - j_{target}$
MAE: 0.720 mA/cm^2
R^2: 0.928
Points: 35398
Experiment 10.6
j_{pred} (mA/cm^2)
j_{target} (mA/cm^2)
counts
$j_{pred} - j_{target}$
MAE: 0.733 mA/cm^2
R^2: 0.921
Points: 35398
Experiment 10.7
j_{pred} (mA/cm^2)
j_{target} (mA/cm^2)
counts
$j_{pred} - j_{target}$
MAE: 0.613 mA/cm^2
R^2: 0.949
Points: 35398
Experiment 10.8
j_{pred} (mA/cm^2)
j_{target} (mA/cm^2)
counts
$j_{pred} - j_{target}$
MAE: 0.613 mA/cm^2
R^2: 0.947
Points: 35398
Experiment 10.9
j_{pred} (mA/cm^2)
j_{target} (mA/cm^2)
counts
$j_{pred} - j_{target}$
MAE: 0.629 mA/cm^2
R^2: 0.946
Points: 35398

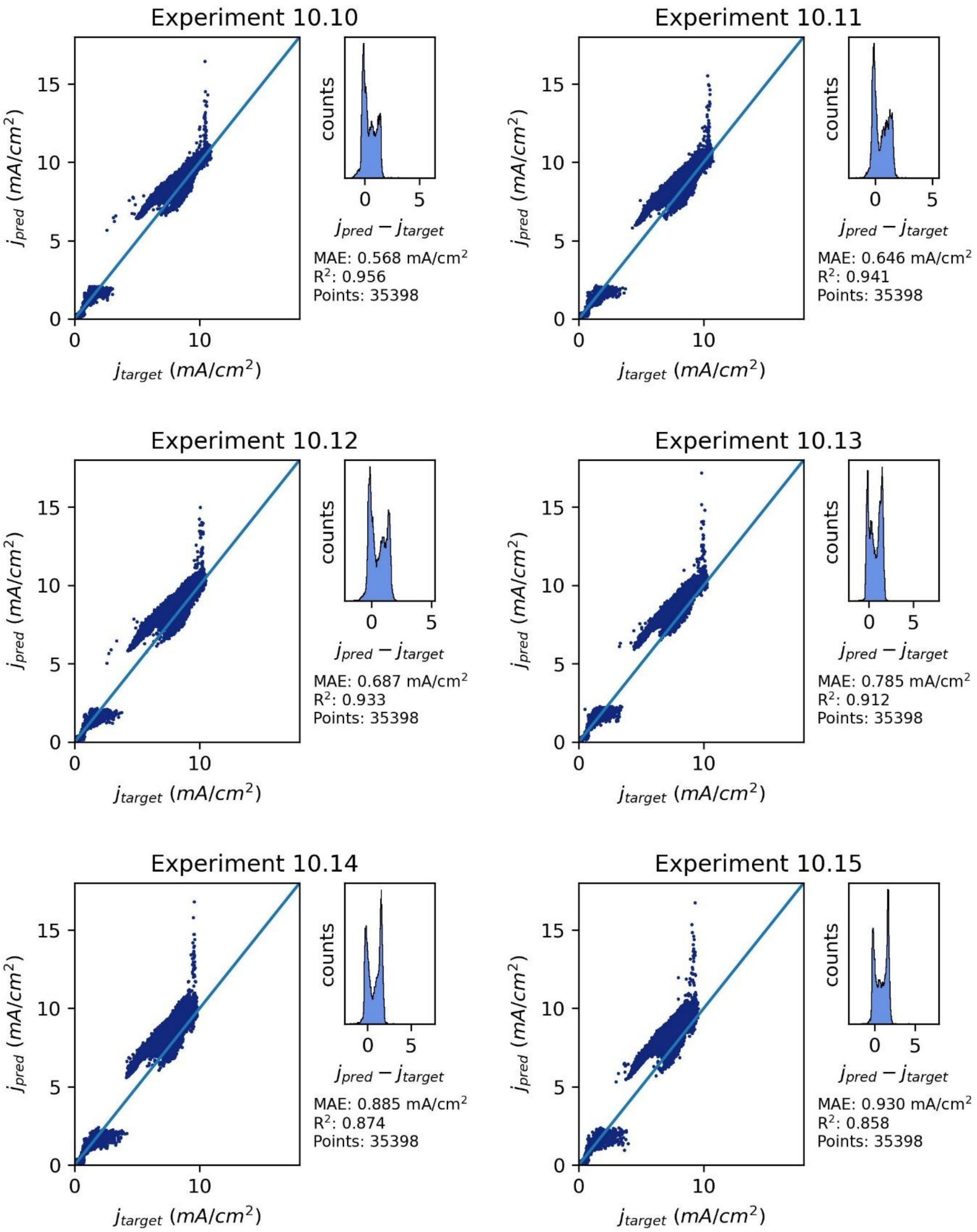
Experiment 10.10
MAE: 0.568 mA/cm^2
R^2: 0.956
Points: 35398
Experiment 10.11
MAE: 0.646 mA/cm^2
R^2: 0.941
Points: 35398
Experiment 10.12
MAE: 0.687 mA/cm^2
R^2: 0.933
Points: 35398
Experiment 10.13
MAE: 0.785 mA/cm^2
R^2: 0.912
Points: 35398
Experiment 10.14
MAE: 0.885 mA/cm^2
R^2: 0.874
Points: 35398
Experiment 10.15
MAE: 0.930 mA/cm^2
R^2: 0.858
Points: 35398
j_{pred} (mA/cm^2)
j_{target} (mA/cm^2)
counts
$j_{pred} - j_{target}$

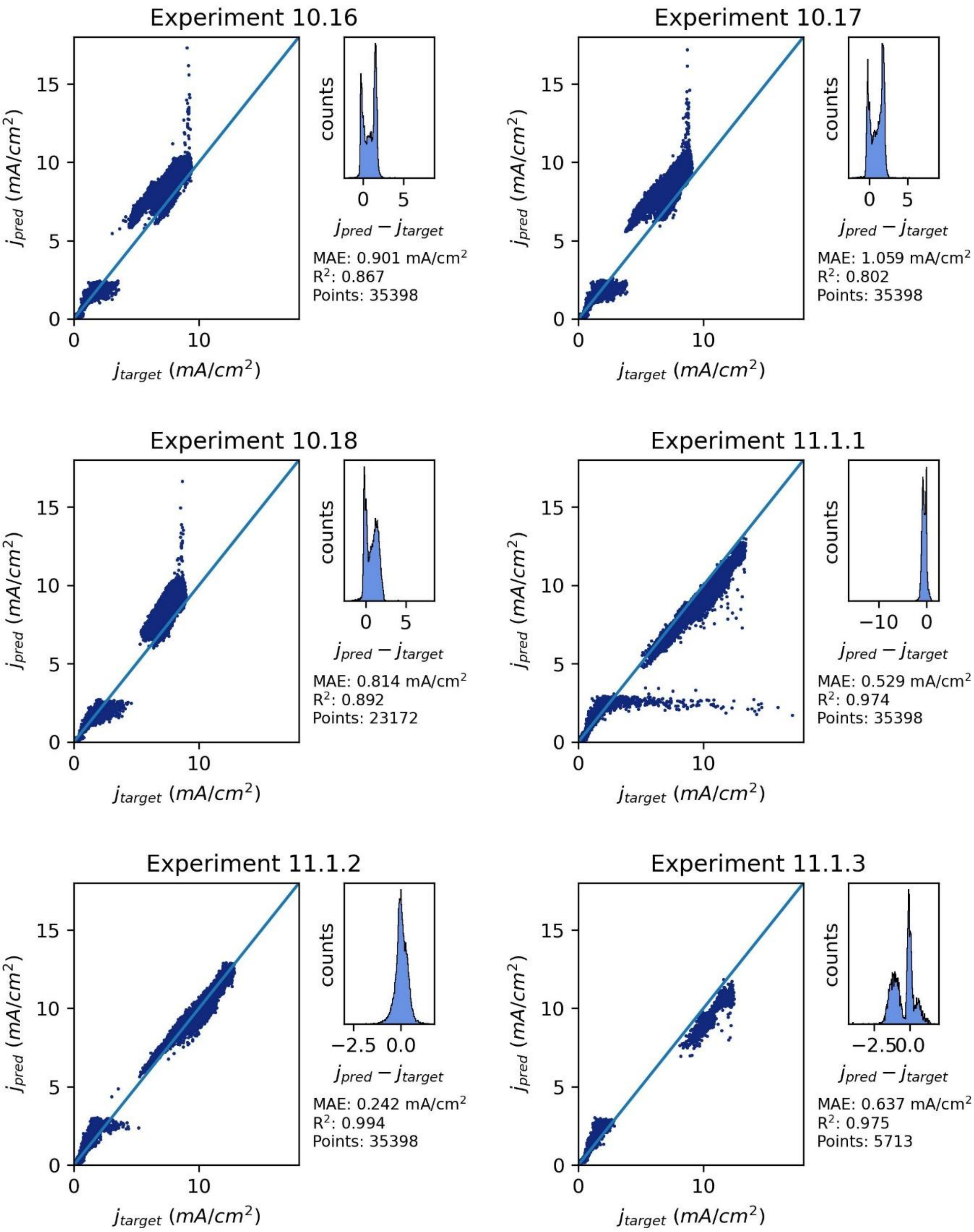
Experiment 10.16
MAE: 0.901 mA/cm²
R²: 0.867
Points: 35398
Experiment 10.17
MAE: 1.059 mA/cm²
R²: 0.802
Points: 35398
Experiment 10.18
MAE: 0.814 mA/cm²
R²: 0.892
Points: 23172
Experiment 11.1.1
MAE: 0.529 mA/cm²
R²: 0.974
Points: 35398
Experiment 11.1.2
MAE: 0.242 mA/cm²
R²: 0.994
Points: 35398
Experiment 11.1.3
MAE: 0.637 mA/cm²
R²: 0.975
Points: 5713
j_pred (mA/cm²)
j_target (mA/cm²)
counts
j_pred − j_target

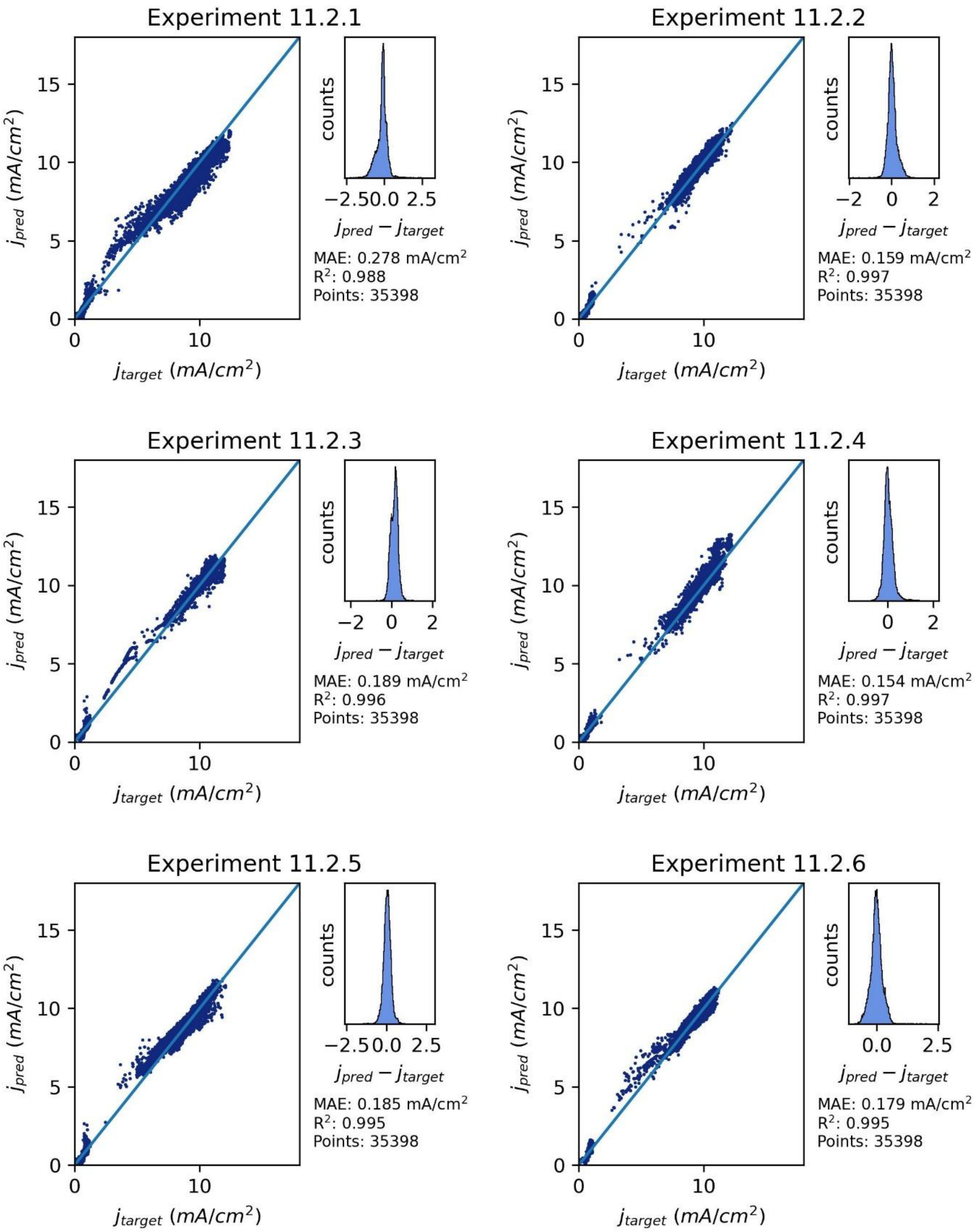
Experiment 11.2.1
MAE: 0.278 mA/cm2
R2: 0.988
Points: 35398
Experiment 11.2.2
MAE: 0.159 mA/cm2
R2: 0.997
Points: 35398
Experiment 11.2.3
MAE: 0.189 mA/cm2
R2: 0.996
Points: 35398
Experiment 11.2.4
MAE: 0.154 mA/cm2
R2: 0.997
Points: 35398
Experiment 11.2.5
MAE: 0.185 mA/cm2
R2: 0.995
Points: 35398
Experiment 11.2.6
MAE: 0.179 mA/cm2
R2: 0.995
Points: 35398
counts
j_pred − j_target
j_pred (mA/cm2)
j_target (mA/cm2)

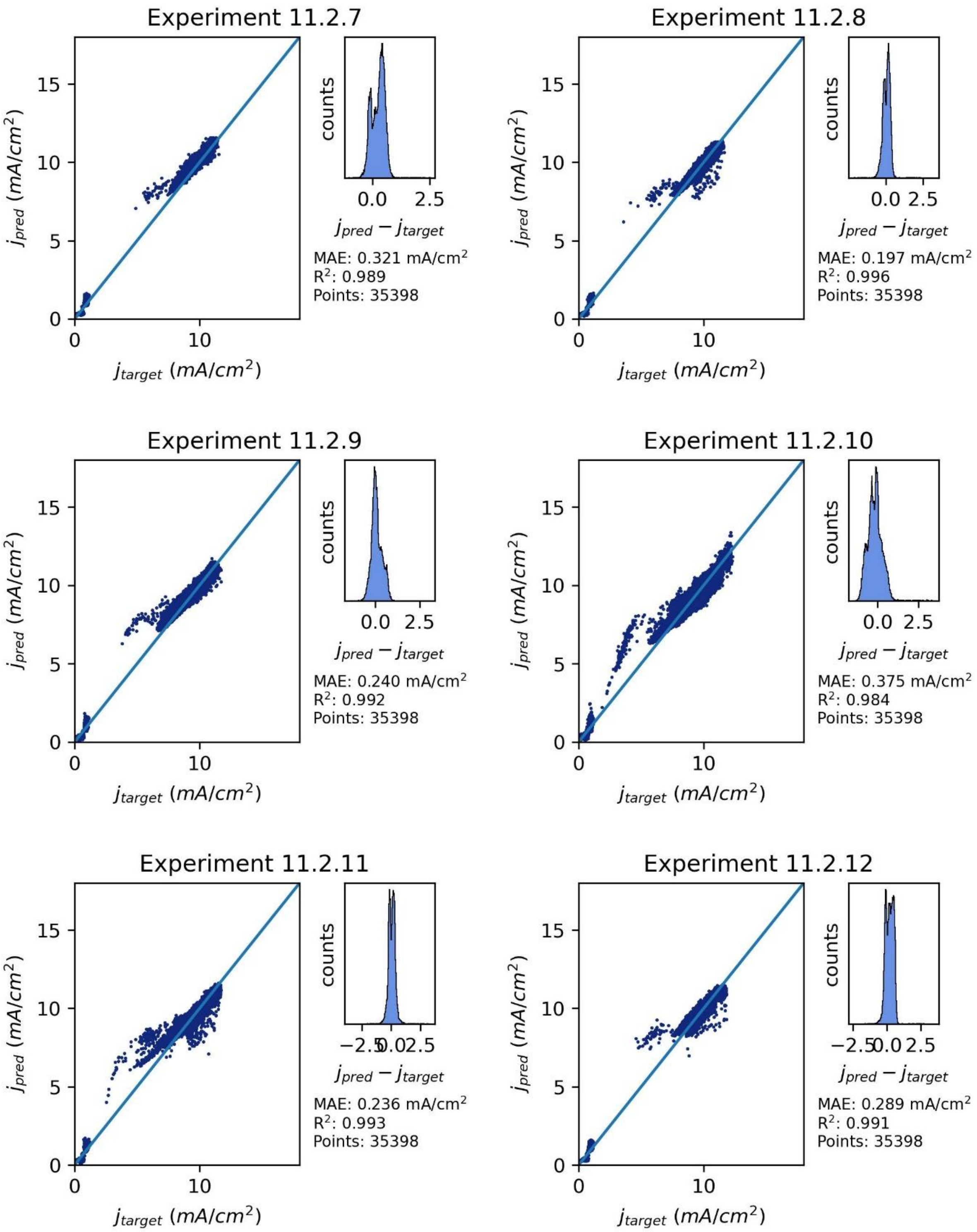
Experiment 11.2.7
MAE: 0.321 mA/cm^2
R^2: 0.989
Points: 35398
Experiment 11.2.8
MAE: 0.197 mA/cm^2
R^2: 0.996
Points: 35398
Experiment 11.2.9
MAE: 0.240 mA/cm^2
R^2: 0.992
Points: 35398
Experiment 11.2.10
MAE: 0.375 mA/cm^2
R^2: 0.984
Points: 35398
Experiment 11.2.11
MAE: 0.236 mA/cm^2
R^2: 0.993
Points: 35398
Experiment 11.2.12
MAE: 0.289 mA/cm^2
R^2: 0.991
Points: 35398
j_{pred} (mA/cm^2)
j_{target} (mA/cm^2)
counts
$j_{pred} - j_{target}$

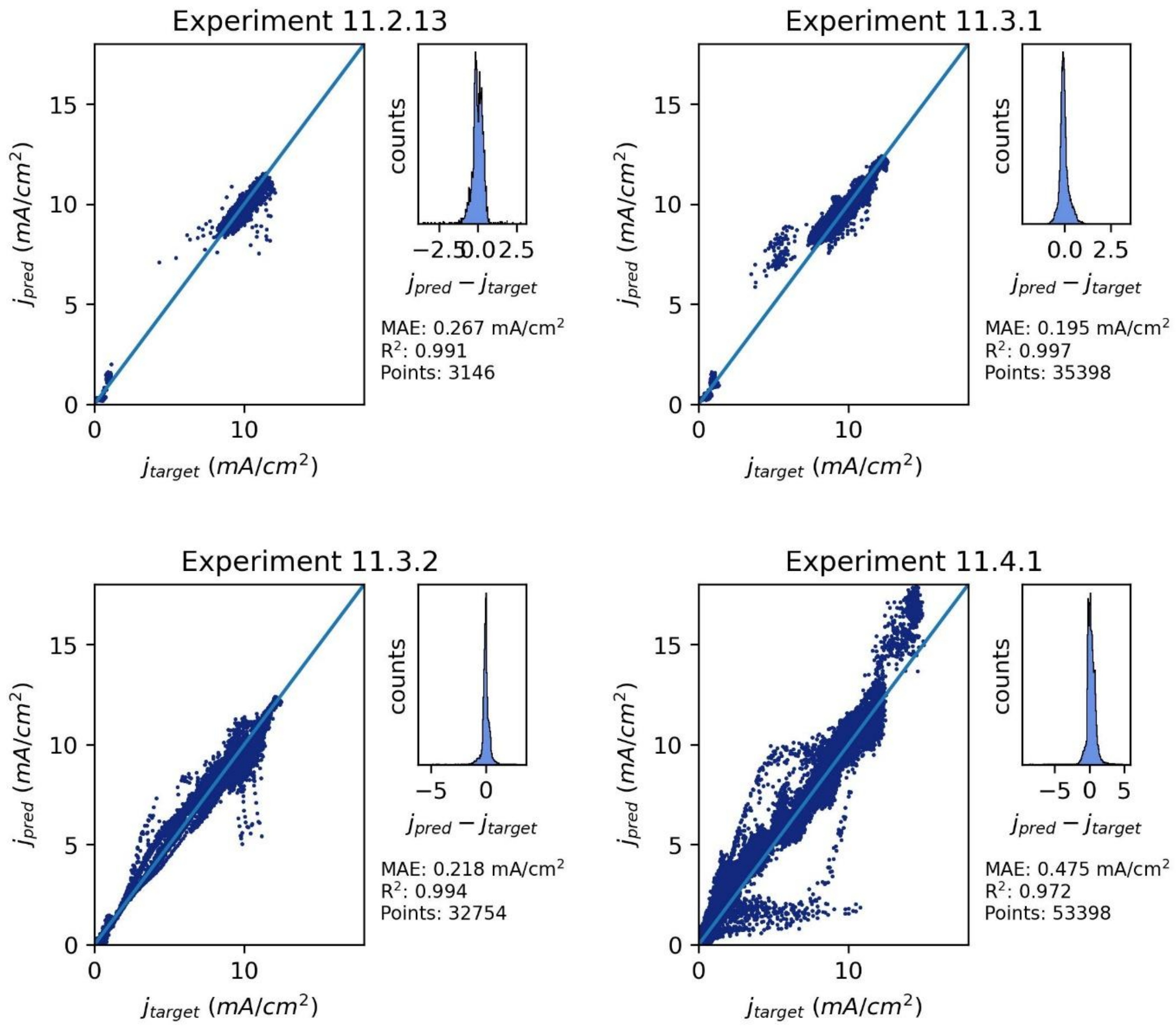

Figure S66. *Parity plots of the training set using a free running simulation with a burn in of 600 steps and the optimal checkpoint.*

VII Reference List

1. Hausknecht, M. & Stone, P. Deep Recurrent Q-Learning for Partially Observable MDPs. http://arxiv.org/abs/1507.06527 (2015).

2. Ansel, J., Yang, E., He, H., Gimelshein, N., Jain, A., Voznesensky, M., Bao, B., Bell, P., Berard, D., Burovski, E., Chauhan, G., Chourdia, A., Constable, W., Desmaison, A., DeVito, Z., Ellison, E., Feng, W., Gong, J., Gschwind, M., Hirsh, B., Huang, S., Kalambarkar, K., Kirsch, L., Lazos, M., Lezcano, M., Liang, Y., Liang, J., Lu, Y., Luk, C. K., Maher, B., Pan, Y., Puhrsch, C., Reso, M., Saroufim, M., Siraichi, M. Y., Suk, H., Zhang, S., Suo, M., Tillet, P., Zhao, X., Wang, E., Zhou, K., Zou, R., Wang, X., Mathews, A., Wen, W., Chanan, G., Wu, P. & Chintala, S. PyTorch 2: Faster Machine Learning Through Dynamic Python Bytecode Transformation and Graph Compilation. in *Proceedings of the 29th ACM International Conference on Architectural Support for Programming Languages and Operating Systems, Volume 2* 929–947 (ACM, New York, NY, USA, 2024). doi:10.1145/3620665.3640366.

3. van Hasselt, H., Guez, A. & Silver, D. Deep Reinforcement Learning with Double Q-Learning. *Proceedings of the AAAI Conference on Artificial Intelligence* **30**, (2016).

4. Kobayashi, T. & Ilboudo, W. E. L. t-soft update of target network for deep reinforcement learning. *Neural Networks* **136**, 63–71 (2021).

5. Jayasankar, B. & Karan, K. O2 electrochemistry on Pt: A unified multi-step model for oxygen reduction and oxide growth. *Electrochim. Acta* **273**, 367–378 (2018).

6. Plub-in, K., Ye, H., Morales-Guio, C. G., Titirici, M. M., Stephens, I. E. L. & Hankin, A. Coupling dynamic catalyst regeneration with convective transport enables stable and selective glycerol electrooxidation on platinum. *EES Catal.* https://doi.org/10.1039/D6EY00090H (2026).

7. Kumar, A., Zhou, A., Tucker, G. & Levine, S. Conservative Q-Learning for Offline Reinforcement Learning. http://arxiv.org/abs/2006.04779 (2020).

8. Mints, V. A., Pedersen, J. K., Olsen, J. C., Plenge, M. K., Arenz, M. & Rossmeisl, J. Unraveling the Mixing Entropy-Activity Relationship in High Entropy Alloy Catalysts: The More, The Better? *J. Am. Chem. Soc.* **148**, 4815–4825 (2026).
